# PP2A‐based triple‐strike therapy overcomes mitochondrial apoptosis resistance in brain cancer cells

**DOI:** 10.1002/1878-0261.13488

**Published:** 2023-07-26

**Authors:** Oxana V. Denisova, Joni Merisaari, Riikka Huhtaniemi, Xi Qiao, Laxman Yetukuri, Mikael Jumppanen, Amanpreet Kaur, Mirva Pääkkönen, Сarina von Schantz‐Fant, Michael Ohlmeyer, Krister Wennerberg, Otto Kauko, Raphael Koch, Tero Aittokallio, Mikko Taipale, Jukka Westermarck

**Affiliations:** ^1^ Turku Bioscience Centre University of Turku and Åbo Akademi University Finland; ^2^ Institute of Biomedicine University of Turku Finland; ^3^ Institute for Molecular Medicine Finland (FIMM), HiLIFE University of Helsinki Finland; ^4^ Centre for Biostatistics and Epidemiology (OCBE) University of Oslo Norway; ^5^ Icahn School of Medicine at Mount Sinai New York NY USA; ^6^ Atux Iskay LLC Plainsboro NJ USA; ^7^ Biotech Research & Innovation Centre University of Copenhagen Denmark; ^8^ University Medical Center Göttingen Germany; ^9^ Institute for Cancer Research Oslo University Hospital Norway; ^10^ Donnelly Centre for Cellular and Biomolecular Research University of Toronto Canada

**Keywords:** AKT, apoptosis, glioblastoma, mitochondria, PDK, PP2A

## Abstract

Mitochondrial glycolysis and hyperactivity of the phosphatidylinositol 3‐kinase–protein kinase B (AKT) pathway are hallmarks of malignant brain tumors. However, kinase inhibitors targeting AKT (AKTi) or the glycolysis master regulator pyruvate dehydrogenase kinase (PDKi) have failed to provide clinical benefits for brain tumor patients. Here, we demonstrate that heterogeneous glioblastoma (GB) and medulloblastoma (MB) cell lines display only cytostatic responses to combined AKT and PDK targeting. Biochemically, the combined AKT and PDK inhibition resulted in the shutdown of both target pathways and priming to mitochondrial apoptosis but failed to induce apoptosis. In contrast, all tested brain tumor cell models were sensitive to a triplet therapy, in which AKT and PDK inhibition was combined with the pharmacological reactivation of protein phosphatase 2A (PP2A) by NZ‐8‐061 (also known as DT‐061), DBK‐1154, and DBK‐1160. We also provide proof‐of‐principle evidence for *in vivo* efficacy in the intracranial GB and MB models by the brain‐penetrant triplet therapy (AKTi + PDKi + PP2A reactivator). Mechanistically, PP2A reactivation converted the cytostatic AKTi + PDKi response to cytotoxic apoptosis, through PP2A‐elicited shutdown of compensatory mitochondrial oxidative phosphorylation and by increased proton leakage. These results encourage the development of triple‐strike strategies targeting mitochondrial metabolism to overcome therapy tolerance in brain tumors.

## Graphical abstract

The authors demonstrate that heterogenous glioblastoma and medulloblastoma cell lines display cytostatic responses to double combinations targeting mitochondrial glycolysis, the phosphatidylinositol 3‐kinase/protein kinase B pathway, or protein phosphatase 2A. However, all tested brain tumor cell models were sensitive to a triplet kinase/phosphatase therapy in which all three compounds were combined. The triplet kinase/phosphatase therapy had *in vivo* efficacy in independent intracranial brain tumor models.

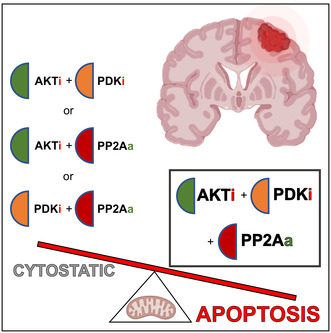

**Abbreviations**
2‐DG: 2‐deoxy‐d‐glucose
AKT: protein kinase B
AKTi: AKT inhibitor
AToMI: actionable targets of multikinase inhibitors screening platform
BBB: blood–brain barrier
CIP2A: cancerous inhibitor of PP2A
DCA: sodium salt of dichloroacetate
ECAR: extracellular acidification rate
ERK: extracellular signal‐regulated kinases
FCCP: carbonyl cyanide‐4 (trifluoromethoxy) phenylhydrazone
GB: glioblastoma
GSC: glioblastoma stem‐like cells
i.c.: intracranial inoculation
MB: medulloblastoma
mTOR: mammalian target of rapamycin
OCR: oxygen consumption rate
OXPHOS: oxidative phosphorylation
PDHE1α: pyruvate dehydrogenase E1 subunit alpha 1
PDK: pyruvate dehydrogenase kinase
PDKi: PDK inhibitor
PDPK1: 3‐phosphoinositide dependent protein kinase 1
PI3K: phosphatidylinositol 3‐kinase
PME‐1: protein phosphatase methylesterase‐1
PP2A: protein phosphatase 2A
s.c.: subcutaneous inoculation
SET: SET nuclear proto‐oncogene
SMAP: small‐molecule activators of PP2A
SV40st: simian virus 40 small‐t antigen

## 1 Introduction

The concept of targeted cancer therapies involves the identification of cancer type‐specific driver mechanisms, followed by targeted inhibition of these mechanisms, with an assumption that this leads to cancer cell death. Protein kinases have been identified as major drivers of many cancers, and effective kinase inhibitors are clinically available. However, most tumors acquire resistance to kinase inhibitors and their combinations [1, 2]. Clinically observed kinase inhibitor resistance is a mechanistic enigma, especially in cancer types genetically driven by the hyperactivation of kinase pathways, such as human glioblastoma (GB) [3, 4, 5, 6]. Acquired therapy resistance develops via two phases: first, through the adaptive development of a drug‐tolerant cellular state, and later, stable resistance that often occurs through the acquisition of genetic mutations [7]. Emerging evidence strongly indicates that drug tolerance is initiated rapidly after drug exposure via nonmutational signaling rewiring, which is often mediated by phosphorylation‐dependent signaling pathways [8, 9]. Therefore, further understanding of the kinase/phosphatase networks controlling nongenetic therapy tolerance can provide novel approaches for targeting brain tumors [10].

Glioblastoma is the most common primary brain tumor in adults associated with a high degree of resistance to therapy, tumor recurrence, and mortality [5, 11]. Extensive genome‐wide profiling studies have established phosphatidylinositol 3‐kinase (PI3K)‐protein kinase B (AKT) signaling as one of the driver pathways contributing to GB disease progression [3, 6, 12]. In addition to its prosurvival effects, the AKT pathway fuels aerobic glycolysis [13], and GB cells are notorious for employing aerobic glycolysis for energy production and survival [14, 15]. Mitochondrial pyruvate dehydrogenase kinase (PDK1‐4; not related to AKT upstream kinase 3‐phosphoinositide dependent protein kinase 1 (PDPK1) also cited as PDK1 frequently in the literature) are hyperactive in GB and function as gatekeepers to direct mitochondrial metabolism toward glycolysis instead of oxidative phosphorylation (OXPHOS), via inactivation of the pyruvate dehydrogenase complex [15, 16]. A recent study demonstrated that AKT activates PDK1 in hypoxic tumors and promotes tumorigenesis, providing a rationale for the combined targeting of AKT and PDK1 in cancer [17]. However, targeting the deregulated AKT and mitochondrial metabolism pathways, even with combination therapies, has achieved dismal clinical response rates in GB [4, 17, 18]. This is consistent with the broad‐range kinase inhibitor tolerance in GB. Mechanistically, kinase inhibitor tolerance has been linked to phosphorylation‐dependent rewiring mechanisms [18] and general apoptosis resistance of glioblastoma stem‐like cells (GSCs) [11]. In addition, intratumoral heterogeneity of GB constitutes a significant therapeutic challenge as the therapies should be effective across cells with different lineages and differentiation statuses [11, 19]. Although vastly different in its origin and patient characteristics, the most common children's brain tumor medulloblastoma (MB) share many features with GB in relation to their clinical resistance to kinase targeting [20].

The Serine/Threonine protein phosphatase 2A (PP2A) broadly regulates phosphorylation‐dependent signaling including several established cancer driver pathways [21]. PP2A inhibition by either genetic mechanisms or overexpression of its endogenous inhibitor proteins promotes tumorigenesis, and this is associated with resistance to cancer therapy in different cancer types [21, 22]. PP2A is frequently inactivated in GB by the overexpression of endogenous PP2A inhibitor proteins, such as protein phosphatase methylesterase 1 (PME‐1), Cancerous Inhibitor of PP2A (CIP2A), SET nuclear proto‐oncogene (SET), and ARPP19 [23, 24, 25]. Particularly, PME‐1 is associated with GB progression [24, 26]. Previous results indicated that the reversal of PME‐1‐mediated PP2A inhibition sensitizes GB cells to cell killing by several multikinase inhibitors such as staurosporine and its analogs [26]. However, the translational impact of these results remains questionable, as neither the siRNA therapies for brain tumors are sufficiently advanced, nor the PME‐1 interaction with PP2A is druggable. Furthermore, staurosporine derivative UCN‐01 (7‐hydroxystaurosporine) does not cross the blood–brain barrier (BBB), and it is currently unclear which of the approximately 50 kinases that are inhibited by UCN‐01 at nanomolar concentrations [27, 28] are the therapeutically relevant target kinases.

Here, we demonstrate widespread tolerance of GB cells to combined AKT and PDK kinase inhibition. However, this widespread GB therapy tolerance can be overcome either by PME‐1 inhibition, or by target‐engaging low micromolar concentrations of pharmacological PP2A reactivators. Mechanistically, we reveal novel impact of pharmacological PP2A reactivation on mitochondrial metabolism, especially in preventing OXPHOS induction that GB cells use as a mitigation strategy to resist the shutdown of glycolysis [15]. We also provide proof‐of‐principle evidence for the translatability of the results by demonstrating significant *in vivo* intracranial tumor growth inhibition by orally dosed triplet therapy combining AKT and PDK inhibition with PP2A reactivation. Collectively, these results reveal that PP2A inhibition constitutes a brain tumor vulnerability by promoting apoptosis resistance. In general, the results provide additional indication that triplet targeting strategies involving mitochondrial targeting could provide significant translational advances in therapies for different cancer types [29].

## 2 Materials and methods

### 2.1 Cell culture and reagents

The established GB cell lines T98G (RRID:CVCL_0556), A172 (RRID:CVCL_0131), and U118MG (RRID:CVCL_0633) were obtained from VTT Technical Research Centre (Turku, Finland), U87MG (RRID:CVCL_0022), U251MG (RRID:CVCL_0021), patient‐derived GSCs, BT3‐CD133^+^ and BT12 [25, 30], and murine immortalized HIF knockout (HIFko) astrocytes [31] from Pirjo Laakkonen (University of Helsinki, Finland), E98‐Fluc‐mCherry (E98) [32] from William Leenders (Radboud University Medical Center, the Netherlands), and immortalized human fibroblasts from Johanna Ivaska (Turku Bioscience, Finland). MB cell lines DAOY (RRID:CVCL_1167) and D283‐Med (RRID:CVCL_1155) were purchased from ATCC (LGC Standards GmbH, Wesel, Germany). The cell lines T98G, U118MG, U251MG, DAOY and D283‐Med were maintained in Eagle MEM (Sigma‐Aldrich, Steinheim, Germany), and U87MG, A172, E98, and human fibroblasts in DMEM (Sigma‐Aldrich). All growth media were supplemented with 10% heat‐inactivated FBS (Biowest, Nuaille, France; 20% FBS for human fibroblasts), 2 mm l‐glutamine, and 50 units·mL^−1^ penicillin/streptomycin. The glioma patient samples were obtained from surgeries at the Kuopio University Hospital (Finland) during the years 2010–2011. Written informed consent was obtained from all subjects, and the experiments were approved by the Research Ethics Committee of North Savo Hospital District (license 53/2009) and conformed to the principles set out in the WMA Declaration of Helsinki and the Department of Health and Human Services Belmont Report [30]. The patient‐derived GSC, BT3‐CD133^+^ and BT12, were maintained in DMEM/F12 (Fisher Scientiﬁc, Vantaa, Finland) containing 1× B27 serum free supplement (Fisher Scientific), 15 mm HEPES (BioNordika, Herlev, Denmark), 2 mm l‐glutamine, 50 units·mL^−1^ penicillin/streptomycin, 50 ng·mL^−1^ fungizone (Invitrogen, Darmstadt, Germany), 10 ng·mL^−1^ hFGF (Peprotech), and 10 ng·mL^−1^ EGF (Peprotech, Hamburg, Germany). The murine immortalized HIFko astrocytes were maintained in BME (Gibco) containing 5% heat‐inactivated FBS, 10 mm HEPES, 1 mm sodium pyruvate (Gibco) and 6 g·L^−1^ glucose, 50 units·mL^−1^ penicillin/streptomycin. The established GB and MB cell lines were authenticated by short tandem repeat profiling (Eurofins Genomics, Ebersberg, Germany). All cell lines were grown in a humidified atmosphere of 5% CO_2_ at 37 °C and were mycoplasma free.

T98G PME‐1 knockout cells were generated using CRISPR/Cas9 as described previously [33]. T98G cells expressing simian virus 40 small‐t (SV40st) antigen were generated using the piggyBac plasmid with SV40st (a gift from V. Gorbunova, University of Rochester, USA) using the nucleofection method [34].

UCN‐01, AKT1/2 inhibitor, and the sodium salt of dichloroacetate (DCA) were purchased from Sigma‐Aldrich, and MK‐2206 from MedChemExpress (Monmouth Junction, NJ, USA). The SMAPs (NZ‐8‐061, DBK‐794, DBK‐1154, and DBK‐1160) were kindly provided by M. Ohlmeyer. All compounds were dissolved in DMSO, except DCA, which was dissolved in mQ and stored as described previously [33]. For *in vivo* experiments, DBK‐1160 was dissolved in 10% *N*,*N*‐dimethylacetamide (Sigma‐Aldrich), 10% Kolliphor HS 15 (Sigma‐Aldrich), and 80% sterile water [25], kept at room temperature, protected from light and administered 100 mg·kg^−1^ orally (p.o.) by oral gavage twice a day. MK‐2206 was dissolved in 30% Captisol (A Ligand Technology, San Diego, CA, USA) and 70% sterile water and administered at a dose of 100 mg·kg^−1^ p.o. every second day [35]. Dichloroacetate was dissolved in PBS (Biowest) and administered at 100 mg·kg^−1^ p.o. twice a day [36].

### 2.2 RNAi‐based knockdown

T98G cells (2 × 10^5^ cells in six‐well plates) were reverse transfected with siRNAs (PME‐1: 5′GGAAGUGAGUCUAUAAGCA3′ and SCR: 5′CGUACGCGGAAUACUUCGA3′) using Lipofectamine RNAiMAX (Invitrogen) according to the manufacturer's instructions. All siRNAs were used at a final concentration of 10 nm. The next day, the optimized numbers of cells were replated into either 96‐ or 12‐well plates and used for cell viability or colony formation assays.

### 2.3 Cell viability assay

Cells (2.5 × 10^3^ or 10 × 10^3^) were plated in 96‐well plates. After adhesion, the cells were treated with vehicle (DMSO) or the compounds at indicated concentrations. In experiments with murine astrocytes and human fibroblasts, concentration of FBS was adjusted to 10%. After 72 h, cell viability was measured using the CellTiter‐Glo assay (Promega, Madison, WI, USA) according to the manufacturer's instructions using a BioTek Synergy H1 plate reader (BioTek, Winooski, VT, USA).

### 2.4 Colony formation assay

Colony formation assays were performed as described previously [25]. For assays requiring adherent cells, BT3‐CD133^+^ and BT12 cells were cultured on matrigel‐coated plates (Becton Dickinson, Franklin Lakes, NJ, USA). Colony formation assays under hypoxic conditions were performed in an InvivO2 400 incubator (Ruskinn Technology, Bridgend, UK) with the following conditions: 1% O_2_, 5% CO_2,_ and 90% humidity.

### 2.5 Caspase‐3 and ‐7 activity assay

T98G cells (2.5 × 10^3^) were plated in 96‐well plates and allowed to adhere. After 24 h, the cells were treated with the indicated drugs in combination with the pan‐caspase inhibitor, Z‐VAD‐FMK (10 mm; Promega). After 24 h, caspase‐3 and ‐7 activities were measured using the Caspase‐Glo 3/7 assay (Promega) according to the manufacturer's instructions.

### 2.6 Long‐term growth assay

E98 cells (3 × 10^3^) were seeded into a 96‐well plate. The next day, the cells were treated with DMSO, MK‐2206 (7 μm), DCA (20 mm), or NZ‐8‐061 (10 μm) alone or in their doublet or triplet combinations (6–12 wells/condition). Every 3–4 days, medium was replaced with fresh medium with or without drugs. The confluency of the wells was determined daily using an IncuCyte ZOOM live‐cell analysis system (Essen Bioscience, Royston Hertfordshire, UK).

### 2.7 Immunoblotting and immunohistochemical staining

Immunoblotting was performed as described previously [25]. Primary antibodies against AKT (Cell Signaling; 9272S, 1 : 1000, Frankfurt, Germany), phospho‐Akt S473 (Cell Signaling; 9271, 1 : 1000), PME‐1 (Santa Cruz Biotechnology; sc‐20086, 1 : 1000, Heidelberg, Germany), phospho‐PDHE1α S300 (Merck Millipore; ABS194, 1 : 1000, Darmstadt, Germany), cleaved PARP1 (Abcam; ab32064, 1 : 1000, Cambridge, UK), SV40 T Ag (Pab 108) (Santa Cruz Biotechnology; sc‐148, 1 : 1000), β‐actin (Sigma‐Aldrich; A1978, 1 : 10 000), and GAPDH (HyTest; 5G4cc, 1 : 10 000, Turku, Finland). Secondary antibodies were purchased from LI‐COR Biotechnology, Bad Homburg, Germany or Dako (Agilent Technologies, Winooski, VT, USA. The histological methods were performed by the Histology core facility of the Institute of Biomedicine, University of Turku, Finland. Brain sections were stained with phospho‐ERK1/2 (Thr202/Tyr204) (Cell Signaling; #9101, 1 : 500) and Ki67 (Dako; M7240, 1 : 500). Slides were scanned using a Pannoramic 250 Flash scanner (3DHISTECH, Budapest, Hungary), and images were acquired using a slideviewer v2.6 (3DHISTECH).

### 2.8 Metabolic assays

Oxygen consumption rate (OCR) and extracellular acidification rate (ECAR) were determined using Seahorse XF Mitochondria Stress and Glycolysis Stress tests (Agilent Technologies), respectively. T98G cells (1 × 10^4^) were plated in XFe96 microplates. The next day, the cells were treated with DMSO, DCA (10 mm), MK‐2206 (7 μm), or NZ‐8‐061 (10 μm) alone or in combination for 24 h. On the day of analysis, cells were washed twice in PBS and 175 μL nonbuffered DMEM supplemented with 10 mm glucose, 1 mm sodium pyruvate, and 2 mm glutamine was added for mitochondria stress test or 2 mm glutamine for glycolysis stress test. Cells were incubated at 37 °C in a non‐CO_2_ incubator for 1 h. The mitochondria stress tests were run by sequential injections of 1.5 μm oligomycin, 1 μm carbonyl cyanide‐4 (trifluoromethoxy) phenylhydrazone (FCCP), and 0.5 μm rotenone/antimycin A and glycolysis stress test: 10 mm glucose, 1 μm oligomycin, and 50 mm 2‐deoxy‐d‐glucose (2‐DG). OCR and ECAR were measured using a Seahorse XFe96 analyzer (Agilent Technologies) and calculated in wave software v2.6.153 (Agilent Technologies). The data were normalized to the total protein per well using BCA assay (Thermo Fisher Scientific).

### 2.9 *In vivo* studies

All animal experiments were approved by the National Animal Experiment Board of Finland (ESAVI/9241/2018 license), and the studies were performed according to the instructions provided by the Institutional Animal Care and Use Committees, University of Turku, Finland. Female athymic nude mice (Hsd:Athymic Nude‐Foxn1^nu^; Envigo, Gannat, France) weighing between 17.7 and 25.5 g were used at 5–6 weeks of age. The mice were housed in individually ventilated cages (IVC, Techniplast; up to five mouse/cage), under controlled conditions of light (12‐h light/12‐h dark), temperature (21 ± 3 °C), and humidity (55 ± 15%) in specific pathogen‐free conditions at the Central Animal Laboratory, University of Turku (Turku, Finland). The mice were given irradiated soy protein‐free diet (2920X; Envigo—Teklad Diets) and autoclaved tap water *ad libitum*.

For intracranial inoculation (i.c.), E98 (1.5 × 10^5^ cells) and DAOY cells (1 × 10^5^ cells) suspended in 5 μL of PBS were injected into the mouse brain as previously described [25]. Coordinates for the injection site from bregma: 1 mm posterior, 2 mm to right, 3 mm depth from the skull. Mice with E98 i.c. xenografts were randomized into equal groups based on the bioluminescence signal [37] and mice with DAOY i.c. xenografts based on animal weights using the same web‐based program. For monitoring tumor growth, bioluminescence imaging for E98‐xenografts was performed using the Xenogen IVIS Spectrum (Caliper Life Sciences, Hopkinton, MA, USA). Drug treatments were initiated after the tumors were visible with bioluminescence 10 days after inoculation and continued for 32 days. The tumor size of the DAOY xenografts could not be measured; however, the well‐being of the animals was closely monitored, and dosing was started 15 days after inoculation and continued for 34 days. Animals were weighed at least twice a week and euthanized when symptoms of terminal tumor burden, such as greater than 15% weight loss, hunched posture, cranial protrusion, and significant neurologic symptoms, were observed.

Subcutaneous (s.c.) xenografts were generated by engrafting 2 × 10^6^ DAOY cells suspended in a 150 μL solution of PBS/Matrigel (1 : 1 v/v). Tumor growth of DAOY s.c. xenografts was monitored three times a week using caliper measurements, and the animals were weighed once a week. The volume of the s.c. tumors was calculated according to the following formula: *W* ^2^ × *L*/2 (*W*—shorter diameter, *L*—longer diameter of the tumor). DAOY tumors were grown for 30 days until the mean tumor volume reached 125 mm^3^, after which drug treatment was initiated for 30 following days. Mice were sacrificed when the tumors reached their maximum size (longer diameter reached 15 mm), and tumors were collected for further use.

### 2.10 Proteome integral solubility alteration assay

For *in cellulo* target engagement of NZ‐08‐61 with subunits of PP2A, we used the Proteome Integral Solubility Alteration assay, as described previously [38]. For this purpose, T98G cells were treated with increasing doses of NZ‐08‐61 for 3 h, after which cellular lysates were collected and analyzed by mass spectrometry.

### 2.11 BH3 profiling

BH3 profiling was performed as previously described [39, 40]. Briefly, T98G cells were pretreated with DMSO, MK‐2206 (5 μm), DCA (20 mm), or NZ‐8‐061 (8 μm), alone or in combination. The next day, cells were permeabilized with 0.002% digitonin and treated with a library of synthetic peptides. The peptides used were BIM (0.01 μm), BAD, HRK, and MS1 (10 μm each). A pore‐forming peptide, alamethicin (positive control), or DMSO (negative control) served as a control. The cells were incubated with peptides for half an hour at 25 °C and subsequently fixed with 4% paraformaldehyde for 10 min. Finally, intracellular cytochrome *c* was stained with an immunofluorescence‐labeled antibody (BioLegend Alexa Fluor 647 anti‐Cytochrome *c* Antibody, clone 6H2.B4). Relative cytochrome *c* release was assessed using formula 1 − [(sample − pos.ctrl.)/(neg.ctrl. − pos.ctrl.)].

### 2.12 Proteomics analysis

LC‐ESI‐MS/MS analyses were performed on an Orbitrap Fusion Lumos mass spectrometer (Thermo Fisher Scientific) equipped with a nano‐electrospray ionization source and FAIMS interface. Compensation voltages of −40, −60, and −80 V were used. Orbitrap Fusion Lumos MS data were acquired automatically using Thermo xcalibur v4.4 software (Thermo Fisher Scientific). The DDA method consisted of an Orbitrap MS survey scan with a mass range of 350–1750 *m/z*, followed by HCD fragmentation for the most intense peptide ions in the top‐speed mode with a cycle time of 1 s for each compensation voltage.

### 2.13 Statistical analyses

At least three biological replicates were performed for cell culture experiments. Data are presented as mean ± SD from three independent experiments, unless otherwise specified. For quantification with two groups, a two‐tailed Student's *t*‐test assuming unequal variances was used. For quantification with more than two groups, one‐way ANOVA analysis (data with normal distribution) or Kruskal–Wallis test (data with non‐normal distribution) was used to assess statistical significance with graphpad prism 9 (GraphPad Software, Boston, MA, USA). Lonk‐rank (Mantel‐Cox) test was used in survival analysis. Statistical significance was set at *P* < 0.05.

## 3 Results

### 3.1 Pharmacological reactivation of PP2A synergizes with a multikinase inhibitor UCN‐01

**Fig. 1:**
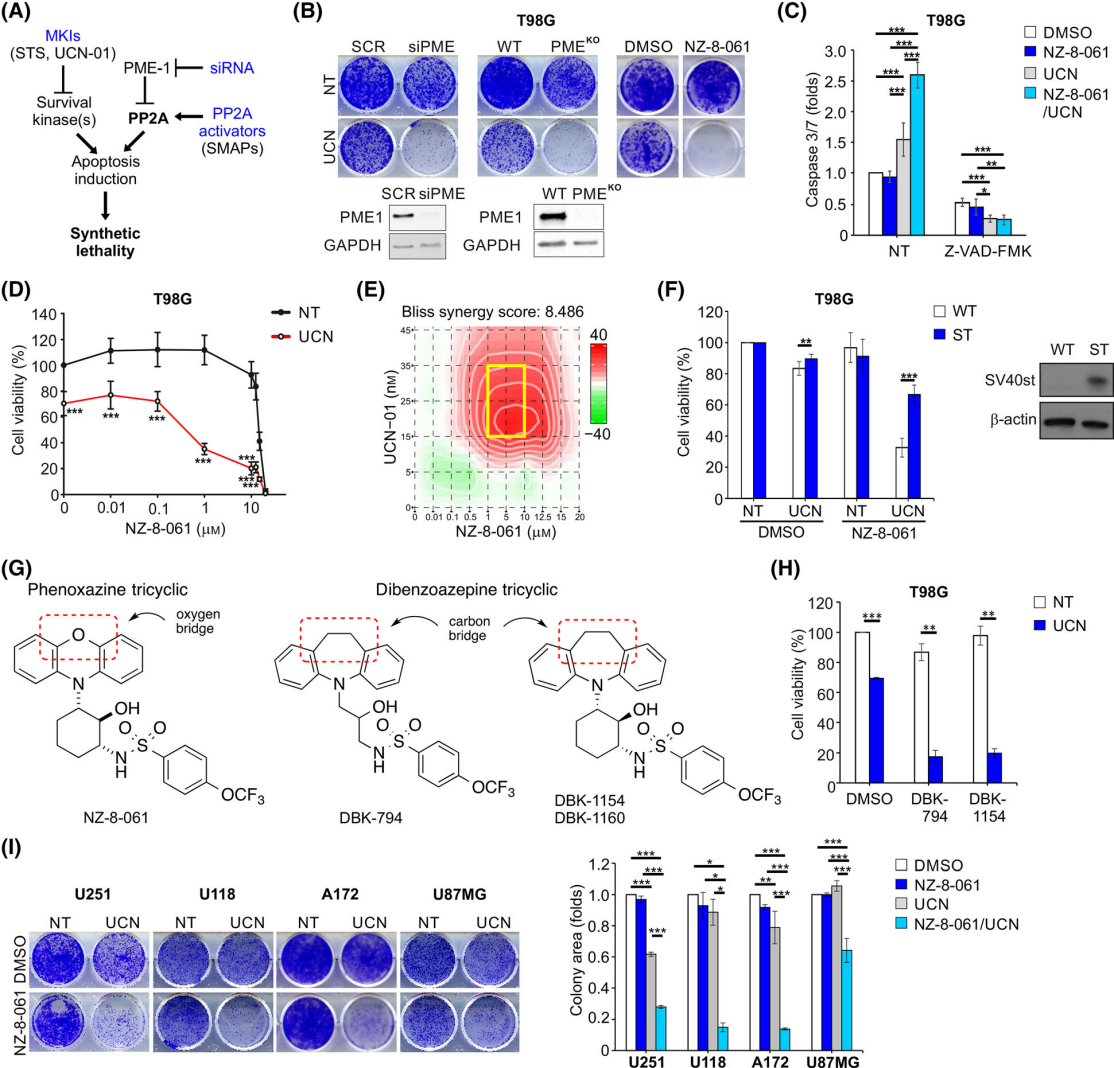
Pharmacological PP2A reactivation and UCN‐01 exert a synergistic effect in glioblastoma cells. (A) Scheme illustrating rationale of testing pharmacological PP2A reactivators in combination with multikinase inhibitions in glioblastoma. MKIs, multi‐kinase inhibitors; SMAPs, small‐molecule activators of PP2A; STS, staurosporine. (B) Colony formation assay in T98G cells under PME‐1 deletion (siRNA or CRISPR/Cas9) or 8 μm NZ‐8‐061 treatment. Cells were treated with 25 nm UCN‐01 (UCN) or left untreated (NT). Immunoblot analysis of PME‐1 (lower panel). These experiments were performed three times, and representative images are presented. (C) Caspase 3/7 activity in T98G cells treated with 8 μm NZ‐8‐061 alone or in combination with 25 nm UCN‐01 under caspase inhibitor Z‐VAD‐FMK (20 μm) for 24 h. Mean ± SD from three independent experiments. *P < 0.05, **P < 0.01, ***P < 0.001, one‐way ANOVA. (D) Viability of T98G cells treated with increasing concentration of NZ‐8‐061 either alone or in combination with 25 nm UCN‐01 for 72 h. Mean ± SD from three independent experiments. ***P < 0.001, Student's *t*‐test. (E) Synergy plot showing the most synergistic area (yellow box) between NZ‐8‐061 and UCN‐01 in T98G cells. The Bliss synergy score is calculated over the whole dose–response matrix using synergyfinder 2.0 web application [63]. (F) Viability of control and simian virus 40 small‐t antigen (SV40st)‐expressing T98G cells treated with 25 nm UCN‐01 and 8 μm NZ‐8‐061, alone or in combination for 72 h. Immunoblot analysis of SV40st (right panel). Mean ± SD from three independent experiments. **P < 0.01, ***P < 0.001, Student's *t*‐test. (G) Structures of two different classes of SMAPs. (H) Viability of T98G cells treated with SMAPs (10 μm DBK‐794 or 5 μm DBK‐1154) alone or in combination with 25 nm UCN‐01 for 72 h. Mean ± SD from three independent experiments. **P < 0.01, ***P < 0.001, Student's *t*‐test. (I) Representative images (left) and quantified data (right) of colony formation assay in U251, U118, A172 and U87MG cells treated with 8 μm NZ‐8‐061 alone or in combination with UCN‐01 (200, 25, 50 and 500 nm, respectively). After 72 h of drug‐treatment, medium was replaced with nondrug medium and the cells were left for another 72 h or until the control well was confluent (T98G, U118, and U87MG: (3) + 3 days, and A172: (3) + 3 + 3 days). Mean ± SD from two independent experiments. *P < 0.05, **P < 0.01, ***P < 0.001, one‐way ANOVA.

To address the significant translational caveats related to experimental evidence that PP2A reactivation by PME‐1 inhibition sensitizes GB cells to staurosporine [26] (Fig. 1A), we first examined whether the recently developed BBB‐permeable SMAPs [21, 25, 41] could be used as pharmacological agents to induce synthetic lethality [42] with clinically used staurosporine derivative UCN‐01. To test this hypothesis, we directly compared the impact of PP2A reactivation resulting from either PME‐1 depletion, or SMAP (NZ‐8‐061) treatment, on the colony growth potential of T98G cells. As shown in Fig. 1B, neither PME‐1 depletion (siRNA), or deletion (CRISPR/Cas9), nor treatment with NZ‐8‐061 induced significant growth defect themselves but induced remarkable synthetic lethality with UCN‐01. The induction of caspase 3/7 activity indicated that the mode of cell death induced by the SMAP+UCN‐01 combination was apoptosis (Fig. 1C; Fig. S1A).

The interaction between NZ‐8‐061 and UCN‐01 was dose‐dependent, and synergy was observed using both compounds at concentrations that showed negligible monotherapeutic activity (Fig. 1D,E). Validating the particular potential of PP2A reactivation in multikinase inhibitor sensitization, NZ‐8‐061 displayed synergistic activity at low concentrations (0.5–2 μm, Fig. 1D,E), which is approximately 10‐fold lower than the concentrations that have been previously shown to be required for monotherapy effects for the compound [25, 41]. NZ‐8‐061 has been shown in number of publications to directly interact with, and impact PP2A complex composition both *in vitro* and *in cellulo* [41, 43]. Consistent with these results, we confirmed *in cellulo* target engagement of NZ‐08‐61 with the B56 subunits of PP2A in T98G cells treated with 2 μm NZ‐08‐61 for 3 h (Fig. S1B). Furthermore, consistent with published data demonstrating the rescue of NZ‐8‐061 effects by overexpression of the selective PP2A inhibitor protein, SV40st antigen [41, 44], the interaction between UCN‐01 and NZ‐8‐061 was abrogated in SV40st expressing T98G cells (Fig. 1F; Fig. S1C). To further rule out the possibility that the synergy between NZ‐8‐061 and UCN‐01 would be mediated by potential non‐selective targets of NZ‐8‐061, we used the SMAPs DBK‐794 and DBK‐1154 derived from the dibenzoapine tricyclic family, which is chemically different from NZ‐8‐061 (Fig. 1G). Both DBK‐794 and DBK‐1154 were originally used to demonstrate the direct interaction between SMAPs and PP2A and to map their interaction regions [41]. Importantly, these chemically diverse SMAPs showed identical drug interactions with UCN‐01 (Fig. 1H; Fig. S1D). Collectively, our evidence mitigates concerns that SMAP effects are related to potential nonselective effects reported using toxic (15–30 μm) concentrations of NZ‐8‐061 (a.k.a. DT‐061) [45]. Additionally, drug interactions were validated across heterogenous GB cell lines (Fig. 1I; Fig. S1D). Importantly, synergy between UCN‐01 and NZ‐8‐061 was not observed in noncancerous fibroblasts and murine astrocytes by cell viability assay nevertheless presented in long‐term colony grow assay (Fig. S1E,F). Synergistic drug interactions in GB cells were also observed under hypoxia, which induces PDK‐mediated glycolysis, a common resistance mechanism in GB (Fig. S1G) [16]. Together these results show that SMAPs, when used at the target‐engaging low micromolar concentrations, have remarkable potential to overcome multikinase therapy tolerance in GB.

### 3.2 Triplet combination of AKTi + PDKi + SMAP induces cytotoxic cell killing of GB tumor cells

We recently developed a generalizable Actionable Targets of Multi‐kinase Inhibitors (AToMI) screening platform, aimed to identify UCN‐01 target kinases that are specifically involved in the synthetic lethality phenotype in GB. The PI3K/AKT/ mammalian target of rapamycin (mTOR) pathway (PIKCA, AKT1, and AKT3), and mitochondrial pyruvate dehydrogenase kinases (PDK1 and PDK4) were revealed as strongest candidates, and several AKT and PDK inhibitors were validated [33]. To understand whether inhibition of either AKT or PDK alone was sufficient for synergistic interaction with PP2A reactivation, a range of GB cells were treated with either AKT inhibitor (AKTi; MK‐2206) or PDK inhibitor (PDKi; DCA) in combination with SMAP. Similar to what was observed in the AToMI study, the maximal response to combination of SMAP and either AKTi or PDKi was only approximately 50% inhibition in cell viability (Fig. S2A). Notably, cell models used in this study have constitutive but highly heterogeneous AKT and PDK activity (Fig. S2B). Moreover, GB cells showed heterogeneity regarding whether the combination of either AKTi or PDKi with SMAP resulted in the maximal response. This indicates that unlike observed in other cancer types [46], PP2A reactivation combined with only one kinase inhibitor cannot be used as a strategy to kill heterogeneous GB cells.

**Fig. 2:**
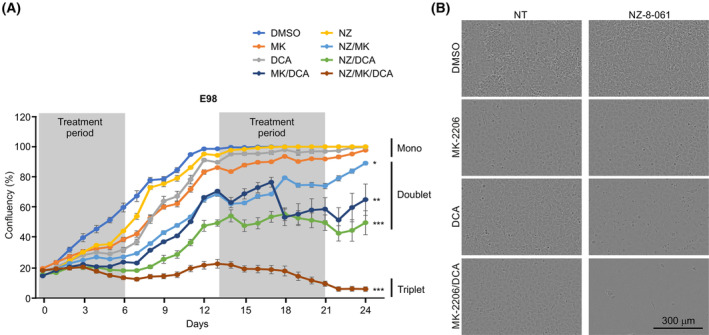
Triplet combination of AKTi + PDKi + SMAP induces cytotoxic cell killing of glioblastoma cells. (A) Incucyte analysis of proliferation of E98 cells treated with DMSO, 7 μm MK‐2206 (MK), 20 mm DCA, 10 μm NZ‐8‐061 (NZ) alone or in doublet or triplet combinations. The cells were treated with drugs twice per week, with a one‐week drug holiday (6 wells per condition). Mean ± SEM. *P < 0.05, **P < 0.01, ***P < 0.001, Kruskal–Wallis test. (B) Representative pictures of E98 cells at day 24. Scale bar, 300 μm.

To better profile the GB driver kinase inhibition and PP2A reactivation responses over time, we performed a long‐term growth assay in E98 cells (Fig. 2A). Based on the inefficiency of combined targeting of one kinase and PP2A (Fig. S2A) [33], we also profiled the triplet combination of AKT and PDK inhibition and PP2A reactivation. The rationale behind the triplet combination is that nongenetic signaling rewiring induced by single and doublet therapies could be avoided by simultaneously targeting several signaling nodes [8, 9]. Consistent with the results of the short‐term viability assays (Fig. S2A) [33], E98 cells displayed cytostatic responses to each monotherapy during the first 6‐day dosing period (Fig. 2A). However, long‐term data confirmed that E98 cells fully escaped all these monotherapy effects. Furthermore, although doublet combinations were found to be more efficient than monotherapies, the cells regained their proliferation after drug washout, indicating only cytostatic effects also with doublet combinations (Fig. 2A, see Days 6–13 and 21–24). However, the triplet therapy‐treated cells were unable to escape the therapy during follow‐up and showed clear signs of a cytotoxic response after the initiation of the second dosing period (Fig. 2A,B).

Collectively, these results demonstrated that long‐term cultured E98 cells can escape any doublet combinations but not triplet targeting of AKT, PDK, and PP2A.

### 3.3 Triplet kinase/phosphatase therapy is effective across heterogeneous GB tumor cells

**Fig. 3:**
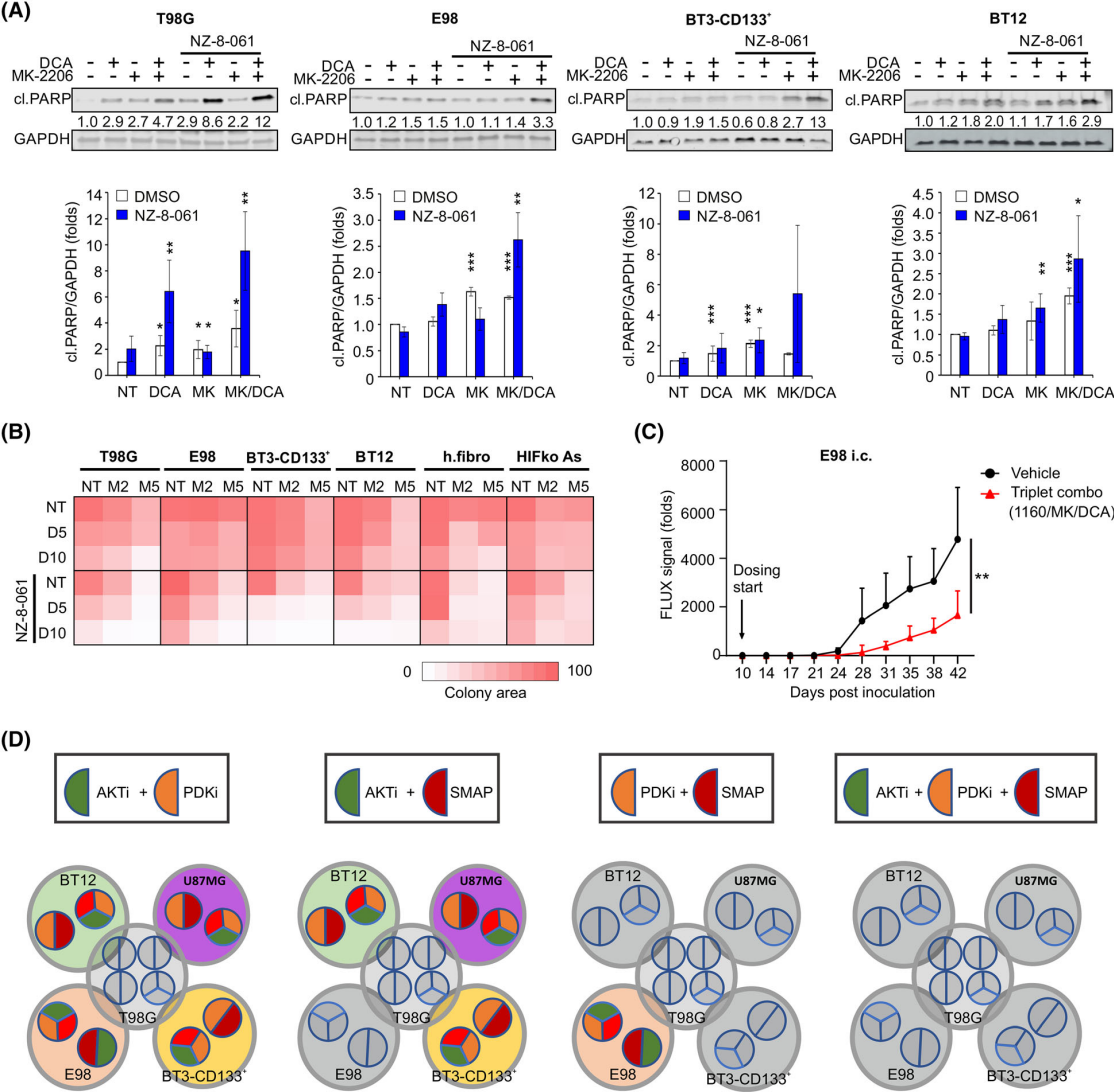
Triplet kinase/phosphatase therapy in molecularly heterogeneous glioblastoma cell lines. (A) Immunoblot assessment (top) and normalized quantifications (bottom) of cleaved PARP in the indicated cell lines treated with 20 mm DCA, 7 μm MK‐2206 (MK) or 10 μm NZ‐8‐061 alone or in doublet or triplet combination for 24 h. Numerical values for protein band intensities are shown below the gels. The same loading controls (GAPDH) have been used for the Western Blots shown in Fig. 5A. Mean ± SD from three independent experiments. *P < 0.05, **P < 0.01, ***P < 0.001, Student's *t*‐test vs DMSO. (B) Heat map representation of quantified colony growth assay data in the indicated cell lines treated with MK‐2206 (M, 2.5 and 5 μm), DCA (D, 5 and 10 mm) or NZ‐8‐061 alone or in doublet or triplet combination. Human fibroblasts and murine astrocytes were set as normal controls. Mean data from two independent experiments. (C) Bioluminescence follow up of E98 i.c. xenografts comparing the vehicle and triplet kinase/phosphatase therapy (DBK‐1160 (100 mg·kg^−1^ twice a day) + MK‐2206 (100 mg·kg^−1^ every second day) + DCA (100 mg·kg^−1^ twice a day)). Each group had *n* = 9 mice, mean ± SEM. **P < 0.01, Student's *t*‐test. (D) Summary scheme of effectiveness of doublet and triplet combination in heterogeneous glioblastoma models. Cell lines sensitive to the indicated doublet (AKTi + PDKi, AKTi + SMAP and PDKi + SMAP) or triplet (AKTi + PDKi + SMAP) therapies are displayed as larger gray circles, whereas inside each larger cycle the color‐coded smaller circles indicate the drug combinations that induced cytotoxicity in the corresponding cell line. The only combination that uniformly eradicated all cell lines was the triplet (AKTi + PDKi + SMAP) therapy. These data are summarized from all cell viability and colony growth experiments throughout the study.

Cellular heterogeneity and the presence of GSCs are significant causes of therapeutic tolerance of GB. Therefore, we first revealed the impact of PP2A reactivation on converting the cytostatic kinase inhibitor responses to cytotoxic in heterogeneous GB cell lines, including patient‐derived GSCs. The used GB models are genetically heterogeneous and display significant kinase inhibitor resistance [25]. Western blot analysis of cleaved PARP confirmed that PP2A reactivation forced cells to apoptosis (Fig. 3A). The highest level of cleaved PARP was found in all cell lines from samples treated with the triplet therapy. Apoptosis sensitization was confirmed by analysis of mitochondrial cytochrome *c* release in T98G cells (Fig. S2C). Furthermore, PP2A reactivation as the mechanism inducing synergistic drug interaction in the context of triplet therapy was validated by genetic PME‐1 inhibition (Fig. S2D).

We then validated our results on the long‐term colony growth of heterogeneous GB cell lines, including patient‐derived GSCs. Notably, consistent with the Incucyte results, all cell lines, except for T98G, were resistant to combined AKT and PDK inhibition (DCA + MK‐2206) (Fig. 3B; see Fig. S2E for raw data). In contrast, the triplet therapy (NZ‐8‐061 + DCA + MK‐2206) showed its efficiency across all GB and GSC lines, with moderate effects on noncancerous human fibroblasts and murine astrocytes (Fig. 3B; Fig. S2E). Eventually, the effect of orally administered triplet therapy on orthotopic intracranial GB tumor growth was tested using E98 cells. The oral administration of triplet therapy was initiated upon the appearance of detectable tumors on Day 10. Despite significant inhibition of intracranial tumor growth (Fig. 3C) and target engagement by the decreased intensity of phosphorylated extracellular signal‐regulated kinases (pERK1/2) staining in the triplet therapy‐treated tumors (Fig. S3E), the therapy did not result in survival benefits (Fig. S3A). This may be due to the highly invasive nature of E98 tumors and their frequent invasion to brain ventricles regardless of the tumor size. This causes neurological symptoms that force culling of the mice prematurely. No therapy‐limiting toxicities were observed during the triplet therapy treatment period (Fig. S3B–D). However, as expected, SMAP treatment resulted in a reversible increase in liver weight, as previously reported [41].

Collectively, our data show that among the tested GB cell lines, the cells are vulnerable to either AKTi + SMAP or PDKi + SMAP combinations, but none of the doublet combinations can effectively kill all cell lines (Fig. 3G). However, the triplet kinase/phosphatase combination strategy (AKTi + PDKi + SMAP) is effective across heterogeneous GB cell lines and should be considered to prevent development of therapy tolerance.

### 3.4 Impact of triplet kinase/phosphatase therapy on medulloblastoma

**Fig. 4:**
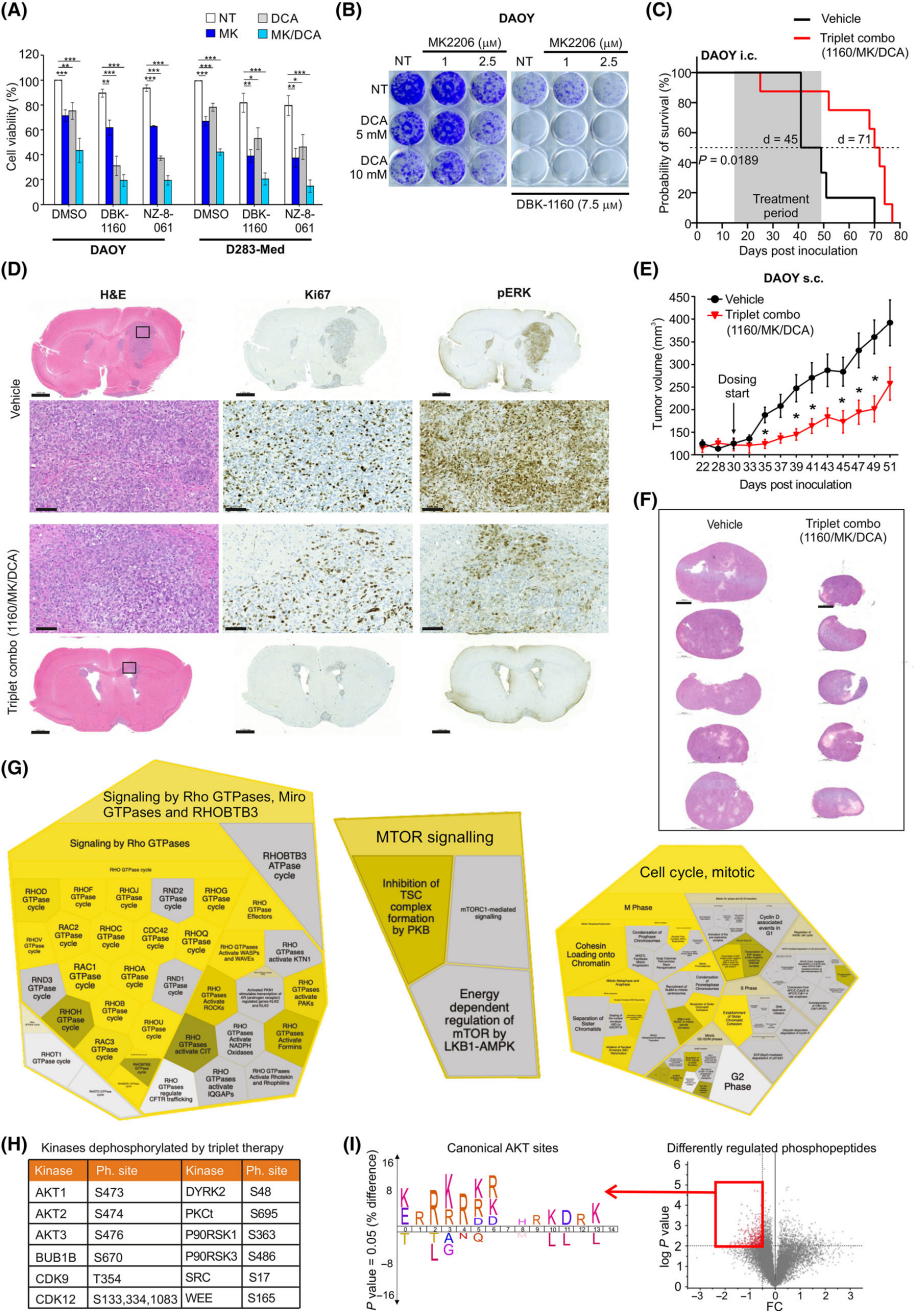
Triplet kinase/phosphatase therapy demonstrates efficacy in MB *in vitro* and *in vivo*. (A) Cell viability in MB DAOY and D283‐Med cells treated with DMSO, 8 μm DBK‐1160 or 10 μm NZ‐8‐061 alone or in combination with 5 μm MK‐2206 (MK), 20 mm DCA, or MK + DCA for 72 h. Mean ± SD from three independent experiments. *P < 0.05, **P < 0.01, ***P < 0.001, one‐way ANOVA, Kruskal–Wallis test. (B) Colony growth assay in DAOY cells under the triplet combination as indicated. These experiments were performed three times, and a representative image is presented. (C) Kaplan–Meier survival analysis of xenograft orthotopic DAOY model treated with triplet combination (DBK‐1160 (100 mg·kg^−1^ twice a day) + MK‐2206 (100 mg·kg^−1^ every second day) + DCA (100 mg·kg^−1^ twice a day)). Vehicle control (*n* = 6) has a median survival of 45 days, and triplet combo (*n* = 8) median survival is 71 days (*P* = 0.0189, Mantel‐Cox test). (D) Brain sections stained with H&E, Ki67, and pERK1/2 from DAOY i.c. xenografts in the end of treatment period as indicated in (C). Vehicle control mouse (*n* = 1, day 49) and Triplet combo mouse (*n* = 1, Day 52). Scale bars, 2000 μm (whole brain section) and 100 μm (scaled area). (E) Quantification of tumor volume from DAOY s.c. tumors in mice treated with DBK‐1160 (100 mg·kg^−1^ twice a day) + MK‐2206 (100 mg·kg^−1^ every second day) + DCA (100 mg·kg^−1^ twice a day), or vehicle control. Each group had *n* = 10 mice. *P < 0.05, Student's *t*‐test. (F) H&E staining of DAOY s.c. tumor samples (*n* = 5 per each group). Scale bar, 1000 μm. (G) Examples of Reactome processes based on significantly regulated phosphopeptides from the triplet therapy‐treated DAOY s.c. xenograft samples in (F). The entire Reactome analysis is shown in Fig. S4 and Table S2. (H) Selected kinases dephosphorylated by the triplet therapy‐treated DAOY s.c. xenograft samples from (F). (I) Volcano plot showing differentially regulated phosphopeptides from (F). Icelogo kinase motif enrichment analysis from the dephosphorylated peptides (cut off *P* ≤ 0.01, log2FC ≤ −0.5, red rectangle) revealed enrichment of canonical AKT sites (R‐x‐R‐x‐x‐S/T and R‐x‐x‐S/T).

To further expand the model that PP2A activity is required for the apoptosis sensitivity of kinase inhibitor‐treated brain tumor cells, we used MB cell lines with heterogeneous AKT and PDK basal activity (Fig. S2B). MB is a pediatric brain cancer type for which AKT and PDK kinase inhibitors have proven to be clinically ineffective [20]. Reassuringly, when tested on two MB cell models, DAOY (SHH subtype) and D283‐Med (Group 3) [47], we observed similar synergistic drug interaction between MK‐2206, DCA and SMAPs (NZ‐8‐061 and DBK‐1160), as across the GB cell lines (Fig. 4A). The inefficiency of the AKTi + PDKi combination, but the efficacy of the triplet kinase/phosphatase therapy in long‐term MB cultures, was confirmed by colony growth assay in DAOY cells (Fig. 4B). For intracranial xenografts with DAOY cells, we relied on mouse survival as the endpoint measurement of the therapeutic effect because no tumor growth visualization approaches were available for these tumors. We saw remarkable *in vivo* activity during the dosing period as more than 50% of the vehicle‐treated mice died during this period, whereas only one mouse from the triplet therapy group had to be sacrificed due to neurological symptoms (Fig. 4C). After cessation of therapy on Day 30, due to local regulations, we still observed a very significant increase in mouse survival in the triplet therapy group, associated with a 26‐day prolongation of the median probability of survival (Fig. 4C; Fig. S3F). The target engagement by the triplet therapy was confirmed by decreased pERK1/2 staining (Fig. 4D).

### 3.5 *In vivo* phosphoproteomics analysis confirms widespread shutdown of kinase signaling with the triplet kinase/phosphatase therapy

In parallel to these experiments, we engrafted s.c. DAOY xenografts and treated with the vehicle or the triplet therapy (Fig. 4E; Fig. S3F,G). We next used MS‐phosphoproteomics analysis to molecularly profile the triplet therapy effect in s.c. MB tumors. All downstream analysis was made based on phosphopeptides that were quantifiable from at least three tumors per group (Fig. 4F) and had an FDR of 5% for significance of the difference in phosphopeptide expression between the groups (Table S1). The Reactome pathway analysis validated the impact of triplet therapy on both apoptosis and the cell cycle (Fig. 4G; Fig. S4, Table S2). Furthermore, fully consistent with our model that efficient therapy response in brain tumors requires widespread kinase inhibition, we found inhibition of phosphorylation of several kinases in triplet therapy‐treated tumors (Table S1). Notably, the inhibition of phosphorylation of the activation loop of AKT1, 2, and 3, together with the enrichment of mTOR signaling based on phosphopeptide data (Fig. 4H; Table S1), provides validation of *in vivo* target engagement. Inhibition of AKT signaling was also evident based on kinase target motif enrichment analysis, where canonical AKT target motifs (R‐x‐R‐x‐x‐S/T and R‐x‐x‐S/T) were clearly enriched in phosphopeptides downregulated by triplet therapy (Fig. 4I). In addition to AKT, the dephosphorylated kinases included, for example, transcriptional elongation‐promoting kinase CDK9, which is essential for brain tumor‐initiating cells [48] and a synergistic drug target with SMAPs [49].

### 3.6 Triplet kinase/phosphatase therapy induces apoptosis by combined action of BH3 priming, OXPHOS inhibition, and mitochondrial proton leak

**Fig. 5:**
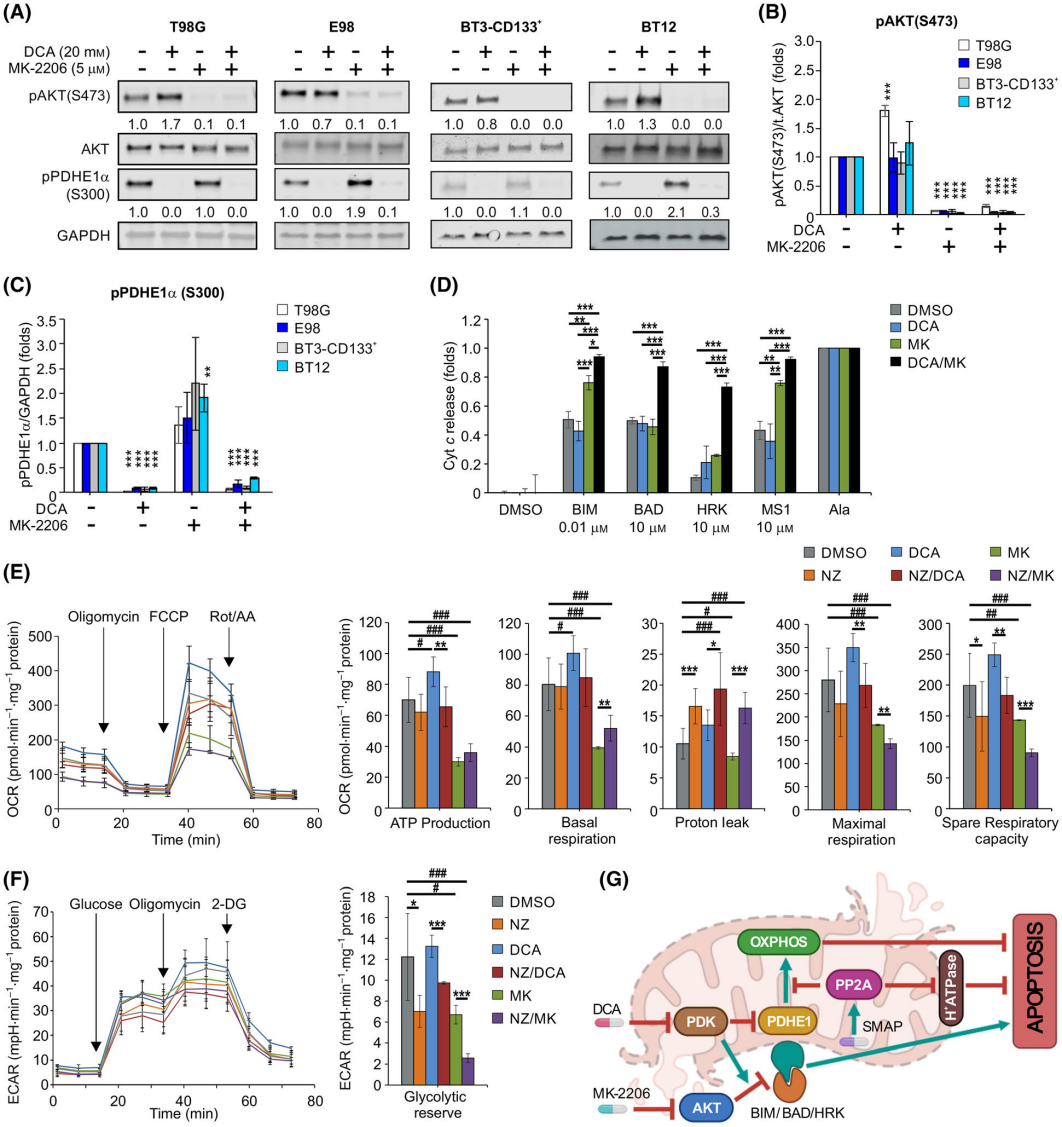
Triplet kinase/phosphatase therapy inhibits mitochondrial OXPHOS and primes BH3 protein‐mediated apoptosis. (A) Immunoblot assessment and normalized quantifications of (B) phosphorylated AKT (S473) and (C) phosphorylated pyruvate dehydrogenase E1 subunit alpha 1 (PDHE1α, S300) in the indicated glioblastoma cells treated with DCA, MK‐2206 or its combination for 24 h. Numerical values for protein band intensities are shown below the gels. The same loading controls (GAPDH) have been used for the Western Blots shown in Fig. 3A. Mean ± SD from three independent experiments. **P < 0.01, ***P < 0.001, Student's *t*‐test to DMSO controls. (D) Priming of T98G cells to apoptosis induction by indicated BH3 peptides. T98G cells treated with 5 μm MK‐2206 (MK), 20 mm DCA alone or its combination for 24 h. Mean ± SD from three independent experiments. *P < 0.05, **P < 0.01, ***P < 0.001, one‐way ANOVA. (E) Mitochondrial stress test and (F) glycolysis stress test profiles and their key parameters in T98G cells treated with 10 mm DCA or 7 μm MK‐2206 (MK) alone or in combination with 10 μm NZ‐8‐061 (NZ) for 24 h. Mean ± SD from three independent experiments. Student's t‐test *P < 0.05, **P < 0.01, ***P < 0.001 to NZ, ^#^ *P* < 0.05, ^##^ *P* < 0.01, ^###^ *P* < 0.001 to DMSO. (G) Schematic illustration of mitochondrial mechanisms for the triplet therapy‐induced apoptosis. PDK and AKT inhibition synergizes on BH3‐mediated apoptosis priming but this is not sufficient for apoptosis execution. Inhibition of PDK also induces compensatory OXPHOS and this is blunted by small‐molecule activators of PP2A (SMAP)‐elicited PP2A re‐activation treatment which additionally induces mitochondrial membrane proton leakage. These PP2A‐mediated mechanisms convert the apoptosis priming by treatment with AKTi + PDKi to terminal apoptosis induction.

To mechanistically understand why the combination of AKT and PDK inhibition was not sufficient for efficient apoptosis induction, we first studied whether the phosphoprotein targets of MK‐2206 and DCA were efficiently inhibited by the combination treatment. Phosphorylation of AKT at S473 was completely abrogated by MK‐2206 treatment (Fig. 5A), whereas DCA completely abolished the phosphorylation of the direct PDK target PDHE1α (pyruvate dehydrogenase E1 subunit alpha 1) [16]. AKT inhibition somewhat enhanced the phosphorylation of PDHE1α in all cell lines, but the combination of MK‐2206 and DCA downregulated the phosphorylation of both target proteins across all cell lines (Fig. 5A–C). Therefore, the inability of the combination of AKT and PDK inhibition to induce apoptosis was not due to the lack of phosphoprotein target inhibition by the drugs.

AKT inhibition is known to directly affect the function of pro‐ and anti‐apoptotic BH3 proteins in the mitochondrial outer membrane, thereby priming cancer cells to induce apoptosis [50]. To study whether the lack of apoptosis induction by AKT and PDK targeting was due to inefficient apoptosis priming, we used dynamic BH3 profiling [39, 40]. Notably, BH3 profiling revealed a marked increase in cell susceptibility to BIM‐, HRK‐, and MS1‐mediated cytochrome *c* release when AKT was inhibited (Fig. 5D). Furthermore, directly indicative of synergism between AKT and PDK, there was a clear enhancement and broadening of BH3‐mediated apoptosis priming when AKT and PDK were co‐inhibited (Fig. 5D). Therefore, combination of AKT and PDK targeting did inhibit their molecular targets (Fig. 5A–C), did prime the mitochondria for BH3 protein‐mediated apoptosis (Fig. 5D), but this was not sufficient for apoptosis induction across heterogeneous GB cell models (Fig. 3A).

Considering the potential mechanisms by which PP2A inhibition prevents apoptosis in GB cells with primed mitochondria (i.e., AKTi + PDKi treated cells), we hypothesized that this could be due to PP2As' impact on mitochondrial metabolism. Recent studies have indicated that cancer cells can use increased OXPHOS as a rescue mechanism against BH3 protein‐mediated apoptosis induction [51], whereas DCA‐elicited inhibition of glycolysis is known to induce compensatory OXPHOS [15]. Therefore, if PP2A reactivation by SMAPs inhibited OXPHOS induction in DCA‐treated cells, the cells would lose their mitigation strategy and commit apoptosis. To assess the impact of PP2A on the mitochondrial metabolism and glycolysis in AKTi or PDKi‐treated GB cells, T98G cells were exposed to either MK‐2206, DCA, or NZ‐8‐061 alone or in combination, and the Seahorse Mito Stress and Glycolysis stress tests were performed. As expected, DCA treatment activated OXPHOS (ATP production), which was also reflected in its positive effect on maximal and spare respiratory activities (Fig. 5E). In contrast, MK‐2206 reduced ATP production and mitochondria‐linked respiration (Fig. 5E). Interestingly, NZ‐8‐061 had a broad‐spectrum effect on mitochondrial metabolism. NZ‐08‐061 completely blocked DCA‐induced OXPHOS and respiration activity (Fig. 5E), demonstrating that PP2A reactivation prevented compensatory mitochondrial survival mechanism. Furthermore, NZ‐8‐061 alone, and especially in combination with DCA, profoundly increased proton leakage, indicating that mitochondrial membrane damage is an additional mechanism of apoptosis induction by the triplet therapy combination (Fig. 5E). The analysis of glycolysis revealed that NZ‐8‐061 decreased the ability of the cells to switch their metabolic dependencies to glycolysis (Fig. 5F; Fig. S5). These data demonstrate that PP2A reactivation prevents several compensatory mitochondrial survival mechanisms. Importantly, DCA failed to reduce glycolysis in T98G cells. At the same time, DCA enhanced the phosphorylation of AKT in T98G cells (Fig. 5A,B), which could compensate for glycolysis inhibition.

All together, these data revealed the mechanistic basis for the high apoptotic activity of triplet kinase/phosphatase therapy. We conclude that, whereas MK‐2206 and DCA synergize in BH3 priming, PP2A reactivation inhibits DCA‐elicited compensatory OXPHOS, induces inner mitochondrial membrane proton leakage, and shut down glycolysis, collectively leading BH3‐primed cells to succumb to apoptosis (Fig. 5G).

## 4 Discussion

Resistance to kinase inhibitors in brain tumors is a notable unmet clinical challenge [4, 18]. Considering that hyperactivated kinase signaling is a hallmark of GB [3, 11], the clinical resistance of GB to kinase inhibitors constitutes a clear mechanistic enigma. Indeed, our results demonstrated that heterogeneous GB cell lines have an astonishing capacity to escape combined inhibition of two kinases with clearly demonstrated driver roles in GB. However, based on our novel results, this kinase inhibitor tolerance can be overcome in both GB and MB cells by simultaneous targeting of three phosphorylation‐dependent signaling nodes: AKT, PDK, and PP2A. Although the use of triplet therapy combinations might be clinically challenging due to the risk of high side effects, many current cancer therapies combine more than two therapeutic agents and have an acceptable side effect profile [52, 53]. As current clinical evidence clearly shows that none of the thus far tested phosphorylation targeting mono‐ or doublet combination therapies have changed the outcome of GB patients [4, 17, 18], we postulate that future research should focus on the concept of triplet therapies, or even higher‐degree combinations, in GB and other brain tumors [29].

Multikinase inhibitors provide an attractive approach to simultaneously inhibit several oncogenic kinases, and some multikinase inhibitors (e.g., sunitinib and PKC412) are clinically used as cancer therapies [54]. However, similar to the more selective kinase inhibitors, all tested multikinase inhibitors have failed in GB clinical trials [4]. To better understand GB‐relevant staurosporine target kinases, we developed the AToMI screening approach and identified several kinases that synergized with PP2A reactivation by either PME‐1 inhibition or SMAPs [33]. Notably, the UCN‐01 target kinases that best synergized with PP2A reactivation were AKT and PDK1‐4, which represent the commonly hyperactivated pathways in GB. For example, the AKT pathway is one of the most dysregulated pathways in GB, whereas PDK kinases play a critical role in GB mitochondrial glycolysis. However, regardless of the importance of the AKT‐PDK axis in GB tumor growth [55], our results challenge the concept that targeting the AKT‐PDK axis would be sufficient for GB therapy alone. Consistent with this, AKT‐ and PDK‐targeting monotherapies have failed to demonstrate significant survival effects in clinical trials for GB [16, 56, 57, 58]. Therefore, multikinase inhibition has not yet provided a solution to overcome brain cancer therapy tolerance. On the contrary, there is a strong theoretical basis for the synergistic activities of simultaneous kinase inhibition and phosphatase activation in phosphorylation‐dependent cancers [10, 21]; however, the therapeutic impact of such a combinatorial approach in brain cancers remains unclear. Unlike other cancer types studied thus far [10, 27, 46, 59], brain cancer cells were here found to be unique in the sense that they are tolerant even to a combination of PP2A reactivation and inhibition of one driver kinase. This could be at least partly explained mechanistically, as in other cancer types, the combinatorial effect of PP2A reactivation has been linked to more efficient shutdown of the kinase pathway, whereas in GB cells, AKT or PDK inhibitors were alone sufficient to completely block the, respectively, signaling pathways. Instead, we identified a novel convergence point for AKT, PDK, and PP2A signaling in the regulation of mitochondrial metabolism and apoptosis sensitivity. Finally, previous studies have shown the monotherapy toxicity of SMAPs in several cancer cell models, including GB cells [25, 46, 60]. However, all these studies used high double‐digit micromolar concentrations of SMAPs, and based on recent evidence, some of these monotherapy effects might be due to off‐targeting [45]. Here, we provide compelling evidence that SMAPs, when used in 10‐fold lower concentrations, are nontoxic, but induce very potent synergy with a combination of either UCN‐01 or AKTi + PDKi. Further evidence for the PP2A‐dependency of these effects was provided by the identical synergy observed by genetic PP2A reactivation [26], the use of SMAPs with different chemistries, and target engagement data and was rescued by SV40st overexpression. Therefore, the role of future PP2A reactivators in brain cancer therapies would rather be as apoptosis sensitizers in higher‐degree combination therapies, where the beneficial therapeutic effects can be unleashed at selective drug concentrations. Furthermore, our results generally indicate that targeting of mitochondrial metabolism could provide a novel approach to overcome tolerance to GB cell kinase inhibitors. Future research is, however, needed to understand how PP2A reactivation blunts compensatory OXPHOS induction in DCA‐treated cells. One interesting future question is whether using direct mitochondrial complex I inhibitors, such as IACS‐010759 [61], would result in the conversion of cytostasis to cytotoxicity in cells treated with a combination of AKT and PDK inhibitors. Moreover, separate investigations focused on T‐cell immunity and tumor microenvironment in response to the triple strike therapy in preclinical syngeneic mouse tumor models are needed.

## 5 Conclusion

Collectively, our data identified the triplet kinase/phosphatase therapy strategy for killing heterogeneous brain cancer cells based on the targeting of critical signaling nodes PP2A, AKT, and PDK. Notably, our results are relevant across heterogeneous GB models, including patient‐derived GSCs, and MB. Based on our results, the uniform kinase inhibitor resistance observed in GB clinical trials [4] could be to significant extent contribute to nongenetic PP2A inhibition by PME‐1 [62]. Clinically, our results strongly indicate that rapidly developing PP2A reactivation therapies [21] will constitute an attractive future therapeutic option for brain tumors when combined with multikinase inhibition. In addition, the results encourage the diagnostic evaluation of PME‐1 and other PP2A‐related biomarkers to identify tumors with high intrinsic PP2A activity for stratification into clinical trials with combinations of clinically tested kinase inhibitors, such as AKT and PDK inhibitors [17, 56, 58].

## Conflict of interest

JW is a consultant and scientific advisory member for Anavo Therapeutics BV.

## Author contributions

OVD, JM, AK, and JW conceptualized and designed the study. OVD, JM, RH, XQ, MT, MP, CS‐F, KW, OK, and RK performed the experiments. MJ, LY, and OK performed bioinformatic analysis. OVD, JM, and RH performed *in vivo* work. OVD, MO, and OK performed data visualization. All authors participated in data interpretation and manuscript writing, review, and editing. All authors read and approved the final manuscript.

### Peer review

The peer review history for this article is available at https://www.webofscience.com/api/gateway/wos/peer‐review/10.1002/1878‐0261.13488.

## Supporting information


**Fig. S1.** Small‐molecule activators of PP2A exert synthetic lethality in heterogeneous glioblastoma cell lines.
**Fig. S2.** Triplet combination of AKTi+PDKi+SMAP induces cytotoxic killing of heterogeneous glioblastoma cell lines.
**Fig. S3.** Mice toxicity data.
**Fig. S4.** Full foamtree presentation of the enriched Reactome processes based on significantly regulated phosphopeptides (p < 0.05) from triplet therapy‐treated DAOY s.c. tumor xenografts.
**Fig. S5.** Glycolytic function parameters.

**Table S1.** Significantly regulated phosphopeptides (p < 0.05) from triplet therapy‐treated DAOY s.c. xenografts.

**Table S2.** Enriched Reactome pathways based on phosphopeptides regulation.

## Data Availability

All data associated with this study are presented in the paper or in the [Link], [Link], [Link].
