# Supplementary material for: PP2A‐based triple‐strike therapy overcomes mitochondrial apoptosis resistance in brain cancer cells
Source: Mol Oncol. 2023 Jul 26;17(9):1803–20. doi: 10.1002/1878-0261.13488 (PMC10483611; doi:10.1002/1878-0261.13488)
Supplement: Supplementary file 1 — Fig. S1. Small‐molecule activators of PP2A exert synthetic lethality in heterogeneous glioblastoma cell lines. Fig. S2. Triplet combination of AKTi+PDKi+SMAP induces cytotoxic killing of heterogeneous glioblastoma cell lines. Fig. S3. Mice toxicity data. Fig. S4. Full foamtree presentation of the enriched Reactome processes based on significantly regulated phosphopeptides (p < 0.05) from triplet therapy‐treated DAOY s.c. tumor xenografts. Fig. S5. Glycolytic function parameters. [file MOL2-17-1803-s001.pdf]

Figure S1

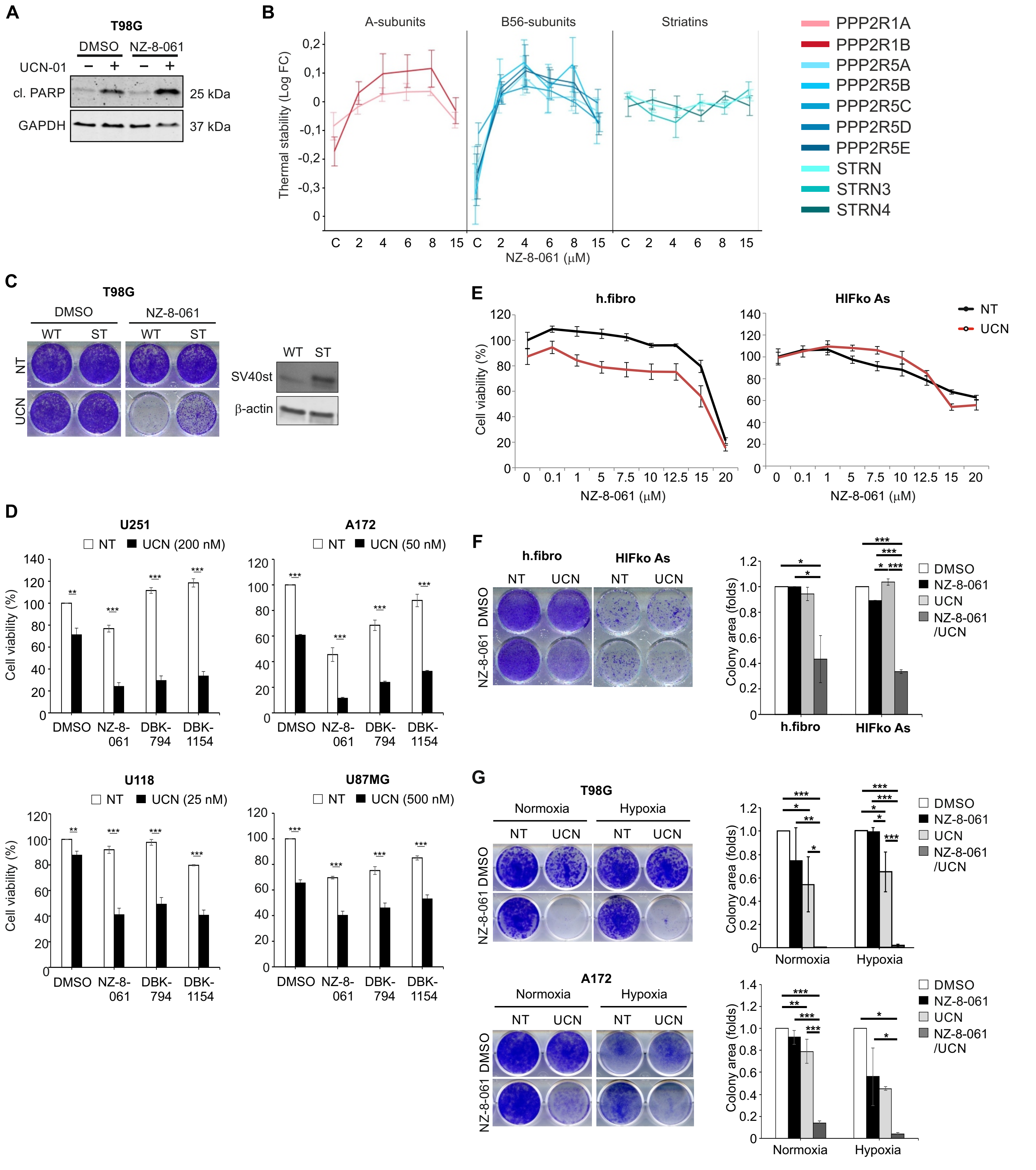

Fig. S1. Small-molecule activators of PP2A exert synthetic lethality in heterogeneous glioblastoma cell lines. A) Immunoblot assessment of cleaved PARP in T98G cells treated with 25 nM UCN-01 and 8 μM NZ-8-061, alone or in combination for 24 h. **B)** In cellulo target engagement of NZ-8-061 with PP2A subunits by PISA assay. T98G cell were treated with NZ-8-061 (2, 4, 6, 8, and 15 μM) for 3h. **C)** Representative images of colony formation assay in control and SV40st-expressing T98G cells treated with 15 nM UCN-01 (UCN) and 8 μM NZ-8-061, alone or in combination. Western blot analysis of simian virus 40 small-t antigen (SV40st, right panel). **D)** Viability of U251, A172, U118 and U87MG cells treated with SMAPs, 10 μM NZ-8-061, 10 μM DBK-794, 5 μM DBK-1154, and UCN-01, alone or in combination for 72 h. Mean±SD from three independent experiments. **P < 0.01, ***P < 0.001, Student's *t*-test. **E)** Viability of human fibroblasts and HIFko astrocytes treated with increasing concentration of NZ-8-061 either alone or in combination with 25 nM UCN-01 for 72h. Mean±SD, n=3. **F)** Representative images (left) and quantified data (right) of colony formation assay in human fibroblasts (h.fibro) and murine HIF knockout astrocytes (HIFko As) treated with 8 μM NZ-8-061, alone or in combination with 25 nM UCN-01. Mean±SD from two independent experiments. *P < 0.05, **P < 0.01, ***P < 0.001, one-way ANOVA. **G)** Representative images (left) and quantified data (right) of colony formation assay in T98G and A172 cells treated with 8 μM NZ-8-061, alone or in combination with UCN-01 (25 nM for T98G; 50 nM for A172) under normoxic and hypoxic conditions. Mean±SD from two independent experiments. *P < 0.05, **P < 0.01, ***P < 0.001, one-way ANOVA.

Figure S2

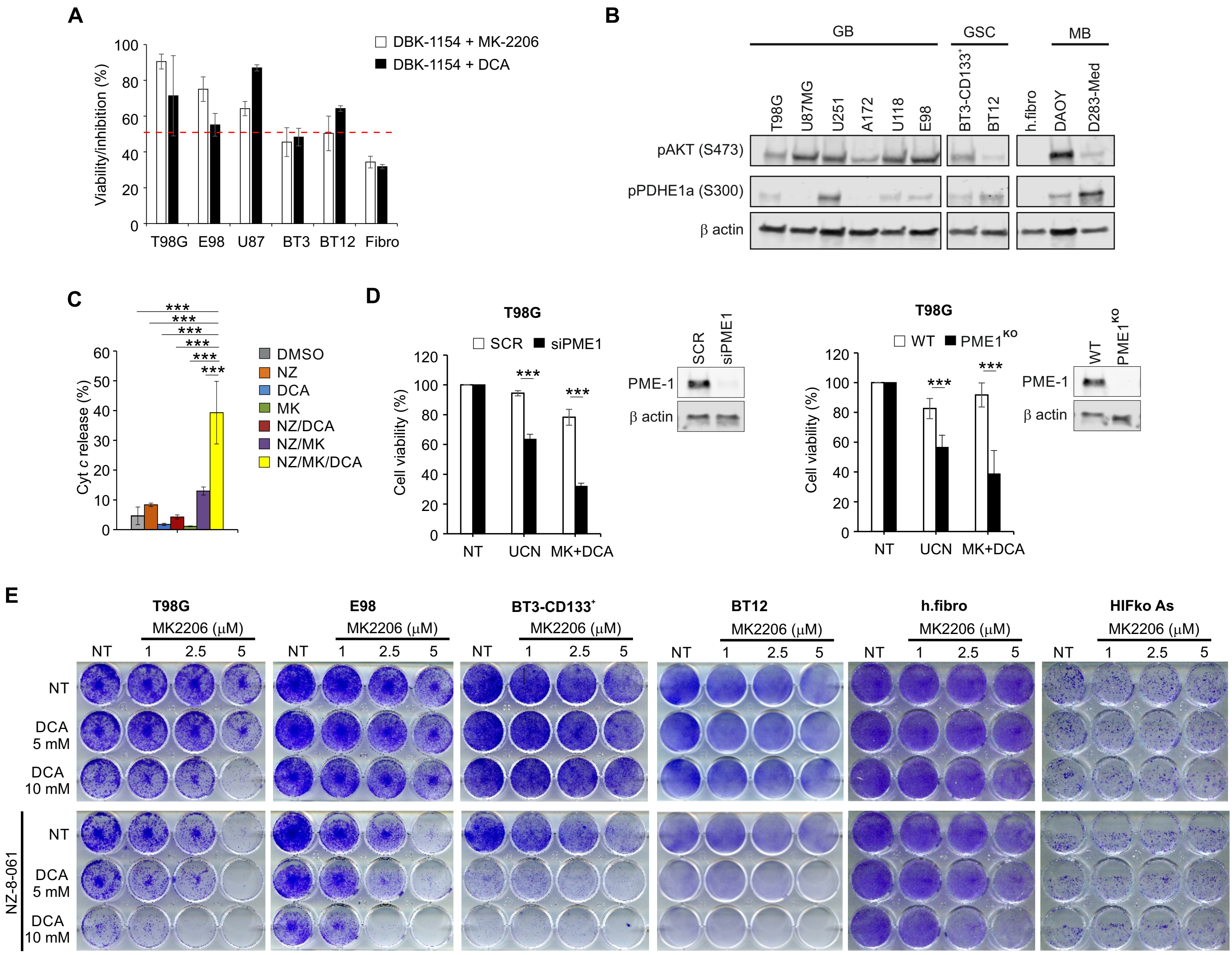

Figure S2. Triplet combination of AKTi+PDKi+SMAP induces cytotoxic cell killing in heterogeneous glioblastoma cell lines. A) Inhibition of cell viability in the indicated glioblastoma cell lines treated with DBK-1154 in combination with MK-2206 or DCA for 72h. Mean±SD from three independent experiments. **B)** Immunoblot assessment of pAKT (S473) and pPDHE1a (S300) in the indicated glioblastoma (GB) and glioblastoma stem-like cells (GSC) lines. Human fibroblasts were used as a control of normal cells. β-actin was used as a loading control. **C)** Cytochrome *c* release in T98G cells treated with 5 μM MK-2206, 20 mM DCA or 8 μM NZ-8-061 alone or in doublet or triplet combination for 24 h. Mean±SD from three independent experiments. *P<0.05, **P<0.01, ***P<0.001, one-way ANOVA. **D)** Cell viability in PME-1 deficient T98G cells treated with 25 nM UCN-01, or doublet combination of 5μM MK-2206 and 20 mM DCA, for 72 h. The efficiency of PME-1 depletion by siRNA or sgRNA confiemed by westen blot analysis (bottom panel). Mean±SD from three independent experiments. ***P<0.001, Student's *t*-test. **E)** Representative images of colony growth assay in heterogeneous GB cell lines under the triplet combination as indicated. Human fibroblasts (h.fibro) and murine HIF knockout astrocytes (HIFko As) were used as a control of normal cells. After 72 h of drug-treatment, medium was replaced with non-drug containing medium and the cells were left for another 72 h or until the control well was confluent (T98G and human fibroblasts: (3)+3 days, and E98, BT3-CD133$^+$, BT12, and HIFko astrocytes: (3)+3+3 days).

Figure S3

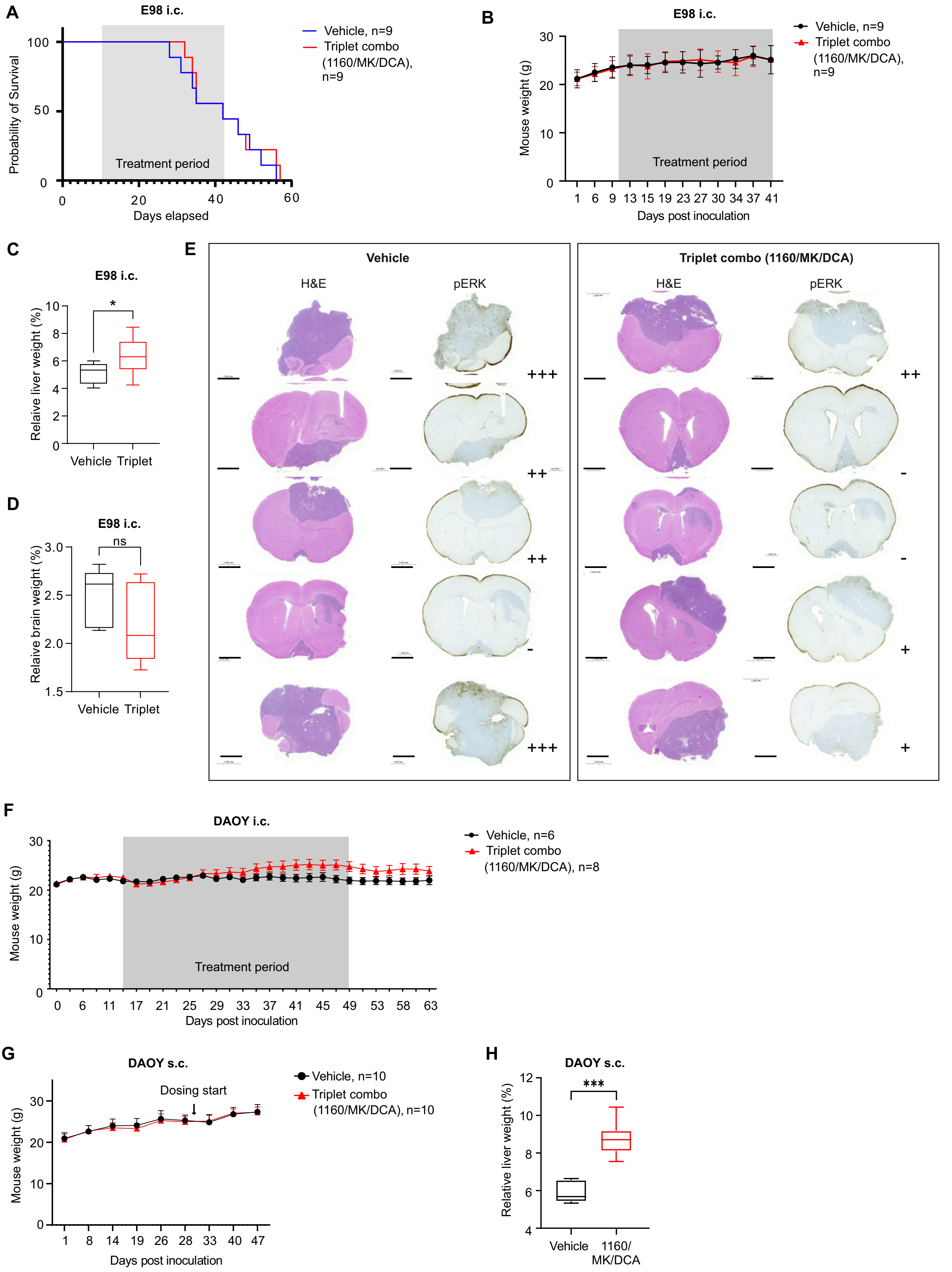

Fig. S3. Mice toxicity data. Probability of survival **A)** and body weights **B)** of mice bearing DAOY i.c. xenograft model with indicated administrations. Data represent mean ± SD. Relative liver **C)** and brain weight **D)** from (A). *P<0.05, Student's t-test. **E)** Brain sections stained with H&E and pERK1/2 from (A). Scale bar, 2000 μm. Body weights of mice bearing DAOY i.c. **F)** and s.c. **G)** xenograft model with indicated administrations. Data represent mean ± SD. **H)** Relative liver weight from mice bearing DAOY s.c. xenograft model from (G). ***P<0.001, Student's t-test.

Figure S4

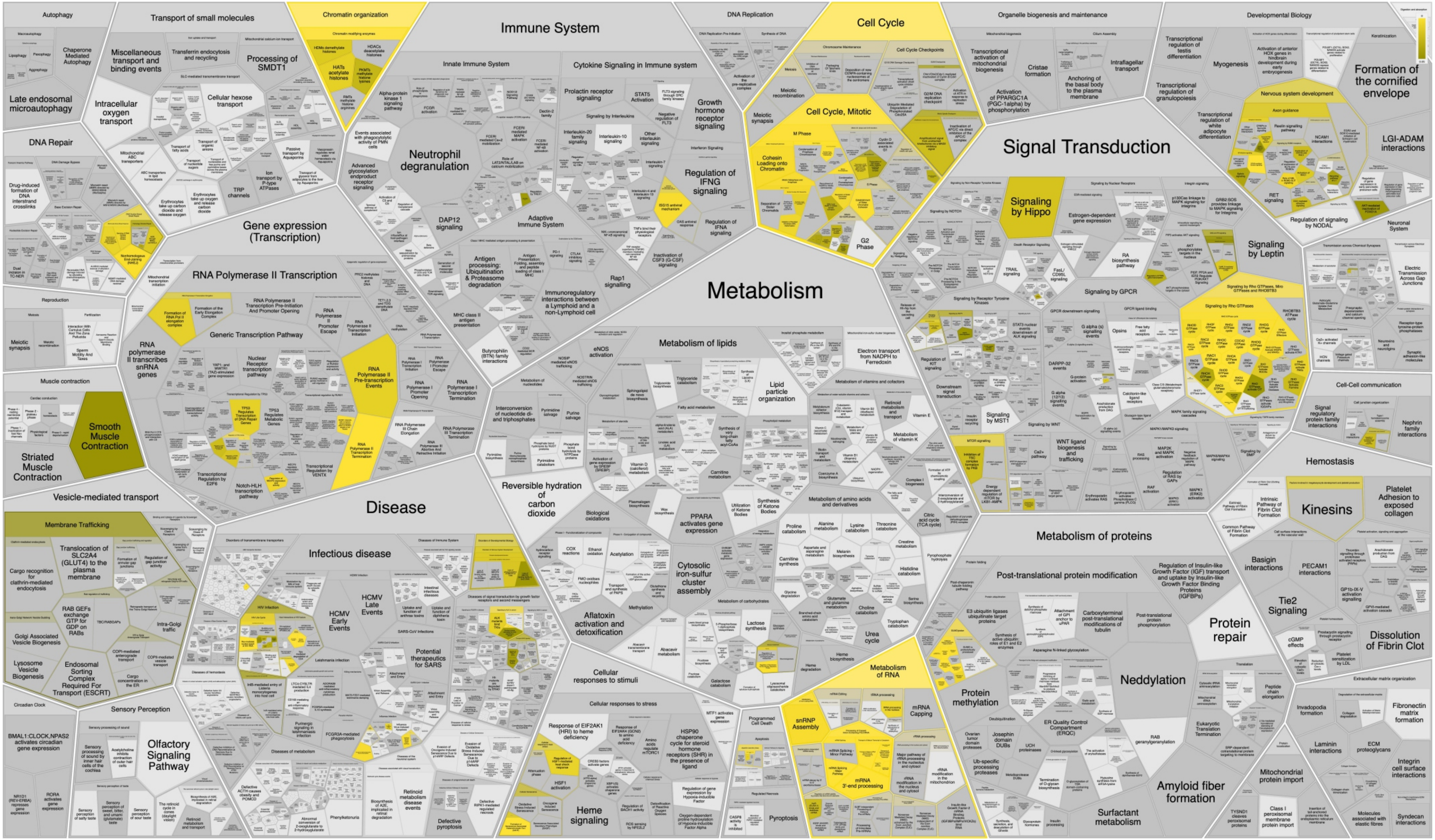

Fig. S4. Full foamtree presentation of the enriched Reactome processes based on Significantly regulated phosphopeptides (p< 0.05) from triplet therapy treated DAOY s.c. tumor xenografts, Related to Fig 4G.

Figure S5

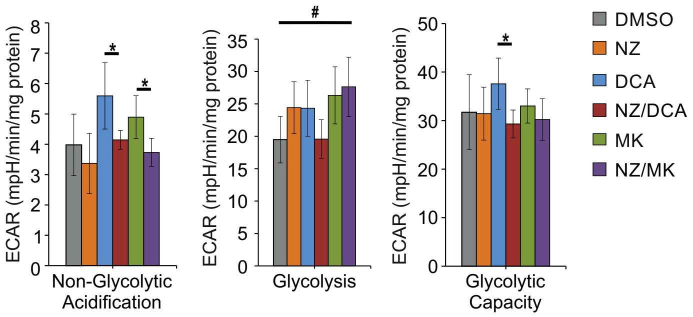

Fig. S5. Glycolytic function parameters. T98G cells were treated with 10 mM DCA or 7 μM MK-2206 (MK) alone or in combination with 10 μM NZ-8-061 (NZ) for 24 h. Mean±SD from three independent experiments. Student's *t*-test *P<0.05 vs NZ, #P<0.05 vs DMSO.
