# Supplementary material for: PP2A‐based triple‐strike therapy overcomes mitochondrial apoptosis resistance in brain cancer cells
Source: Mol Oncol. 2023 Jul 26;17(9):1803–20. doi: 10.1002/1878-0261.13488 (PMC10483611; doi:10.1002/1878-0261.13488)
Supplement: Supplementary file 2 — Table S1. Significantly regulated phosphopeptides (p < 0.05) from triplet therapy‐treated DAOY s.c. xenografts. [file MOL2-17-1803-s002.pdf]

Supplementary table S1. Significantly (p< 0,05) regulated phosphopeptides from triplet therapy treated DAOY s,c, xenografts

Annotated Sequence	Modifications	Master.Protein,Accessions	Positions in Master.Proteins	Modifications in Master.Proteins	Pval	FC	qval
[R],QSIINPDWNFEK,[M]	1xTMT6plex [K12];1xTM	P46459	P46459 [206-217]	P46459 1xTMT6plex [K	0,04	-3,13	0,15
[R],KHSGSRSDEDR,[Y]	1xTMT6plex [K1];1xTM	Q9HCG8	Q9HCG8 [860-870]	Q9HCG8 1xTMT6plex [	0,02	-2,45	0,13
[RC],QVSASELHTSGILGPETLF	1xTMT6plex [N-Term];1	P58107	P58107 [2716-2734]; [3247-3265]; [3	P58107 1xTMT6plex [N	0,01	-2,26	0,12
[R],KNTGSPDR,[K]	1xTMT6plex [K1];1xTM	Q9UIG0	Q9UIG0 [308-315]	Q9UIG0 1xTMT6plex [K	0,01	-2,09	0,12
[R],KEVLASPDR,[L]	1xTMT6plex [K1];1xTM	Q9C0C2	Q9C0C2 [173-181]	Q9C0C2 1xTMT6plex [	0,02	-2,03	0,13
[R],KPIDSLR,[D]	1xTMT6plex [K1];1xTM	Q9UQ35	Q9UQ35 [2680-2686]	Q9UQ35 1xTMT6plex [	0,01	-1,92	0,12
[R],TETPPPLASLNVSK,[L]	1xTMT6plex [K14];1xTM	Q3MHD2	Q3MHD2 [73-86]	Q3MHD2 1xTMT6plex [	0,02	-1,89	0,13
[R],TETPPPLASLNVSK,[L]	1xTMT6plex [K14];1xTM	Q3MHD2	Q3MHD2 [73-86]	Q3MHD2 1xTMT6plex [	0,02	-1,88	0,13
[R],SKSIEER,[E]	1xTMT6plex [K2];1xTM	Q9UBL0;	Q9UBL0 [278-284]; Q15032 [280-286	Q9UBL0 1xTMT6plex [	0,02	-1,88	0,13
[R],SVSGFLHFDTATK,[V]	1xTMT6plex [K13];1xTM	Q92621	Q92621 [1165-1177]	Q92621 1xTMT6plex [K	0,01	-1,82	0,12
[K],FISDRESR,[R]	1xTMT6plex [N-Term];1	Q9H2H9	Q9H2H9 [43-50]	Q9H2H9 1xTMT6plex [	0,01	-1,81	0,12
[R],SVSGFLHFDTATK,[V]	1xTMT6plex [K13];1xTM	Q92621	Q92621 [1165-1177]	Q92621 1xTMT6plex [K	0,03	-1,79	0,13
[R],RDSLSGK,[E]	1xTMT6plex [K7];1xTM	Q92845	Q92845 [100-106]	Q92845 1xTMT6plex [K	0,01	-1,79	0,12
[R],SSSEANQVVRPK,[T]	1xTMT6plex [K12];1xTM	Q6ZT12	Q6ZT12 [1053-1064]	Q6ZT12 1xTMT6plex [K	0,01	-1,79	0,12
[R],QHSSVSSER,[K]	1xTMT6plex [N-Term];1	Q00978	Q00978 [134-142]	Q00978 1xTMT6plex [N	0,01	-1,78	0,12
[K],HSSESPGR,[D]	1xTMT6plex [N-Term];1	P00519	P00519 [880-887]	P00519 1xTMT6plex [N	0,01	-1,78	0,12
[R],KSPENTEGK,[D]	2xTMT6plex [K1; K9];1x	Q15651	Q15651 [5-13]	Q15651 2xTMT6plex [K	0,02	-1,76	0,12
[R],LDSSLPPVR,[R]	1xTMT6plex [N-Term];1	Q9Y4R8	Q9Y4R8 [395-403]	Q9Y4R8 1xTMT6plex [	0,01	-1,75	0,12
[R],KSSSSSEDR,[N]	1xTMT6plex [K1];1xTM	P15056	P15056 [427-435]	P15056 1xTMT6plex [K	0,02	-1,73	0,13
[R],LDKDGIPVSSEAER,[H]	1xTMT6plex [K3];1xTM	Q9Y282	Q9Y282 [108-121]	Q9Y282 1xTMT6plex [K	0,01	-1,69	0,12
[R],KNSSTSTSSSGSR,[R]	1xTMT6plex [K1];1xTM	Q8N6U8	Q8N6U8 [240-252]	Q8N6U8 1xTMT6plex [	0,02	-1,68	0,13
[R],AKPSESR,[H]	1xTMT6plex [K2];1xTM	Q15768	Q15768 [256-262]	Q15768 1xTMT6plex [K	0,02	-1,65	0,12
[R],DDISILATGCK,[G]	1xCarbamidomethyl [C1	Q12888	Q12888 [595-605]	Q12888 1xCarbamidom	0,04	-1,65	0,15
[K],KPIETGSPK,[T]	1xMethyl [K9];1xTMT6p	Q05519	Q05519 [443-451]	Q05519 1xMethyl [K451	0,01	-1,63	0,12
[K],KEESSPSR,[S]	1xTMT6plex [K1];1xTM	P40123	P40123 [253-260]	P40123 1xTMT6plex [K	0,02	-1,63	0,12
[R],LSGALIK,[G]	1xTMT6plex [K7];1xTM	Q9NSC7	Q9NSC7 [392-398]	Q9NSC7 1xTMT6plex [	0,02	-1,63	0,13
[K],KPSSEER,[E]	1xTMT6plex [K1];1xTM	O75475	O75475 [511-517]	O75475 1xTMT6plex [K	0,03	-1,62	0,14
[R],SKDEYEK,[S]	2xTMT6plex [K2; K7];1x	Q13247	Q13247 [265-271]	Q13247 2xTMT6plex [K	0,02	-1,61	0,12
[R],SKSQDQAR,[K]	1xTMT6plex [K2];1xTM	Q13523	Q13523 [230-237]	Q13523 1xTMT6plex [K	0,00	-1,61	0,12
[R],KSSSSSEDR,[N]	1xTMT6plex [K1];1xTM	P15056	P15056 [427-435]	P15056 1xTMT6plex [K	0,03	-1,60	0,13
[R],GDTPGHATPGHGGATSSA	1xTMT6plex [N-Term];1	O75533	O75533 [271-289]	O75533 1xTMT6plex [N	0,01	-1,60	0,12
[R],KLSDASDER,[G]	1xTMT6plex [K1];1xTM	Q658Y4	Q658Y4 [669-677]	Q658Y4 1xTMT6plex [K	0,01	-1,60	0,12
[R],KSPENTEGK,[D]	2xTMT6plex [K1; K9];1x	Q15651	Q15651 [5-13]	Q15651 2xTMT6plex [K	0,01	-1,59	0,12
[R],IDISPSTFR,[K]	1xTMT6plex [N-Term];1	Q9Y2W1	Q9Y2W1 [679-687]	Q9Y2W1 1xTMT6plex [	0,01	-1,59	0,12
[R],RSPSPKPTK,[V]	2xTMT6plex [K6; K9];1x	Q15287	Q15287 [154-162]	Q15287 2xTMT6plex [K	0,02	-1,58	0,13
[R],TVITEEFK,[V]	1xTMT6plex [K8];1xTM	Q96I24	Q96I24 [76-83]	Q96I24 1xTMT6plex [K8	0,03	-1,58	0,13
[R],KSELVVEVK,[S]	2xTMT6plex [K1; K9];1x	P43121	P43121 [605-613]	P43121 2xTMT6plex [K	0,02	-1,56	0,12
[R],SPGFGIK,[K]	1xTMT6plex [K7];1xTM	Q6NWY9	Q6NWY9 [832-838]	Q6NWY9 1xTMT6plex	0,01	-1,56	0,12
[R],SPESRPSVVYPSTK,[F]	1xTMT6plex [K14];1xTM	P42694	P42694 [1317-1330]	P42694 1xTMT6plex [K	0,01	-1,56	0,12
[R],ASPKPTSK,[S]	2xTMT6plex [K4; K8];1x	O43166	O43166 [1527-1534]	O43166 2xTMT6plex [K	0,04	-1,56	0,15
[K],VAHSDKPGSTSTASFR,[D]	1xTMT6plex [K6];1xTM	Q9P0L0	Q9P0L0 [206-221]	Q9P0L0 1xTMT6plex [K	0,02	-1,55	0,12
[R],KADSDSEDK,[G]	2xTMT6plex [K1; K9];1x	P83916	P83916 [86-94]	P83916 2xTMT6plex [K	0,04	-1,53	0,15
[R],KPEEESPR,[K]	1xTMT6plex [K1];1xTM	P49321	P49321 [721-728]	P49321 1xTMT6plex [K	0,02	-1,52	0,13
[R],SPSLDNPTPFPNLGPSENP	1xTMT6plex [K21];1xTM	Q14653	Q14653 [173-193]	Q14653 1xTMT6plex [K	0,02	-1,50	0,13
[R],ARSGTFDLLEMDR,[L]	1xTMT6plex [N-Term];1	Q9NVE7	Q9NVE7 [402-414]	Q9NVE7 1xTMT6plex [	0,04	-1,50	0,16
[R],LSTHSPFR,[T]	1xTMT6plex [N-Term];1	P11171	P11171 [708-715]	P11171 1xTMT6plex [N	0,02	-1,49	0,13
[K],KGNEAVASR,[D]	1xTMT6plex [K1];1xTM	Q8NBJ4	Q8NBJ4 [180-188]	Q8NBJ4 1xTMT6plex [	0,02	-1,49	0,13
[R],DKDSGSSSPLPK,[Y]	2xTMT6plex [K2; K12];1	Q86VQ1	Q86VQ1 [391-402]	Q86VQ1 2xTMT6plex [	0,01	-1,49	0,12
[K],EKMSGSASSENR,[E]	1xOxidation [M3];1xTMT	O43493	O43493 [344-355]	O43493 1xOxidation [M	0,01	-1,49	0,12
[R],SRSHSDNDR,[F]	1xTMT6plex [N-Term];1	O75494	O75494 [156-164]	O75494 1xTMT6plex [N	0,03	-1,48	0,14
[K],SKGSLDR,[L]	1xTMT6plex [K2];1xTM	Q9ULC8	Q9ULC8 [299-305]	Q9ULC8 1xTMT6plex [	0,02	-1,47	0,13
[R],VSPGGGSK,[E]	1xTMT6plex [K8];1xTM	Q6NSZ9	Q6NSZ9 [266-273]	Q6NSZ9 1xTMT6plex [	0,01	-1,47	0,12
[R],EHSPSESEK,[E]	1xTMT6plex [K9];1xTM	Q12986	Q12986 [148-156]	Q12986 1xTMT6plex [K	0,01	-1,46	0,12
[K],KEESSPSR,[S]	1xTMT6plex [K1];1xTM	P40123	P40123 [253-260]	P40123 1xTMT6plex [K	0,03	-1,46	0,14

[R],DKDSGSSSPLPK,[Y]	2xTMT6plex [K2; K12];1	Q86VQ1	Q86VQ1 [391-402]	Q86VQ1 2xTMT6plex [I	0,01	-1,46	0,12
[R],SPSKAVAAR,[A]	1xTMT6plex [K4];1xTM	P60468	P60468 [17-25]	P60468 1xTMT6plex [K	0,00	-1,45	0,11
[R],KPSASER,[L]	1xTMT6plex [K1];1xTM	Q15154	Q15154 [382-388]	Q15154 1xTMT6plex [K	0,01	-1,45	0,12
[R],LDKASPDSR,[H]	1xTMT6plex [K3];1xTM	O15119	O15119 [428-436]	O15119 1xTMT6plex [K	0,00	-1,45	0,11
[R],STQGVTLTDLK,[E]	1xTMT6plex [K11];1xTI	Q9BZL4	Q9BZL4 [559-569]	Q9BZL4 1xTMT6plex [I	0,01	-1,44	0,12
[R],LLASTLVHSVK,[K]	1xTMT6plex [K11];1xTI	Q9NYF8	Q9NYF8 [570-580]	Q9NYF8 1xTMT6plex [I	0,03	-1,44	0,14
[R],KDKSPVR,[E]	2xTMT6plex [K1; K3];1x	Q14498	Q14498 [133-139]	Q14498 2xTMT6plex [K	0,02	-1,44	0,13
[R],KDKEDSDELEEGR,[G]	2xTMT6plex [K1; K3];1x	Q8IZ40	Q8IZ40 [197-209]	Q8IZ40 2xTMT6plex [K	0,02	-1,42	0,12
[R],KYSEVDDSLPSGGEKPSK,[	3xTMT6plex [K1; K15; I	Q05D32	Q05D32 [26-43]	Q05D32 3xTMT6plex [I	0,00	-1,41	0,12
[K],KEEITGTLR,[K]	1xTMT6plex [K1];1xTM	Q9Y490	Q9Y490 [138-146]	Q9Y490 1xTMT6plex [K	0,01	-1,41	0,12
[R],EKAGSPAPSNR,[L]	1xTMT6plex [K2];1xTM	Q9BYW2	Q9BYW2 [610-620]	Q9BYW2 1xTMT6plex [	0,00	-1,41	0,11
[R],SGSAHGSGKSAR,[H]	1xTMT6plex [K9];1xTM	P62995	P62995 [20-31]	P62995 1xTMT6plex [K	0,00	-1,41	0,12
[R],KLSYEIPTEEGGDR,[Y]	1xTMT6plex [K1];1xTM	Q63HR2	Q63HR2 [843-856]	Q63HR2 1xTMT6plex [I	0,03	-1,41	0,14
[R],YDSSNQVK,[G]	1xTMT6plex [K8];1xTM	O95983	O95983 [83-90]	O95983 1xTMT6plex [K	0,02	-1,40	0,13
[R],SSPVRPSSEK,[N]	1xTMT6plex [K10];1xTI	Q9UGL1	Q9UGL1 [1383-1392]	Q9UGL1 1xTMT6plex [I	0,01	-1,39	0,12
[K],AIDSSKK,[L]	2xTMT6plex [K6; K7];1x	Q9NQ86	Q9NQ86 [320-326]	Q9NQ86 2xTMT6plex [I	0,02	-1,39	0,13
[R],SKDASPINR,[W]	1xTMT6plex [K2];1xTM	Q13523	Q13523 [427-435]	Q13523 1xTMT6plex [K	0,01	-1,39	0,12
[R],GSWACSIFDLK,[N]	1xCarbamidomethyl [C5	Q96N67	Q96N67 [189-199]	Q96N67 1xCarbamidom	0,02	-1,38	0,13
[R],KSDASSLMPPLR,[I]	1xTMT6plex [K1];1xTM	Q6IQ26	Q6IQ26 [850-861]	Q6IQ26 1xTMT6plex [K	0,05	-1,38	0,16
[R],SGKNSQEDSEDSEDK,[D]	2xTMT6plex [K3; K15];1	Q9H1E3	Q9H1E3 [50-64]	Q9H1E3 2xTMT6plex [I	0,00	-1,37	0,11
[K],LPESPKR,[A]	1xTMT6plex [K6];1xTM	P17480	P17480 [481-487]	P17480 1xTMT6plex [K	0,02	-1,37	0,12
[R],LTPESQSK,[T]	1xTMT6plex [K8];1xTM	O43426	O43426 [1219-1226]	O43426 1xTMT6plex [K	0,02	-1,36	0,13
[K],TVTVTRPTGVSFK,[K]	1xTMT6plex [K13];1xTI	A5YKK6	A5YKK6 [1051-1063]	A5YKK6 1xTMT6plex [I	0,01	-1,35	0,12
[R],EKSVIDTSTIVR,[K]	1xTMT6plex [K2];1xTM	O43815	O43815 [257-268]	O43815 1xTMT6plex [K	0,00	-1,35	0,11
[R],KGSSSQAPASK,[K]	2xTMT6plex [K1; K11];1	Q9H6F5	Q9H6F5 [215-225]	Q9H6F5 2xTMT6plex [I	0,02	-1,35	0,13
[R],KSEDDSAVPLAK,[A]	2xTMT6plex [K1; K12];1	Q9Y2W2	Q9Y2W2 [599-610]	Q9Y2W2 2xTMT6plex [	0,00	-1,35	0,11
[R],LSTHSPFR,[T]	1xTMT6plex [N-Term];1	P11171	P11171 [708-715]	P11171 1xTMT6plex [N	0,03	-1,34	0,14
[R],ASPDLWPEQLPGVAEFAAS	1xTMT6plex [K21];1xTI	Q52LA3	Q52LA3 [27-47]	Q52LA3 1xTMT6plex [K	0,03	-1,33	0,14
[K],LKEDSPR,[K]	1xTMT6plex [K2];1xTM	Q9ULD9	Q9ULD9 [1266-1272]	Q9ULD9 1xTMT6plex [I	0,01	-1,33	0,12
[R],LSASLPDTVGQLK,[R]	1xTMT6plex [K13];1xTI	Q9HAC8	Q9HAC8 [163-175]	Q9HAC8 1xTMT6plex [I	0,02	-1,32	0,13
[R],TSSPHKEESPK,[K]	2xTMT6plex [K6; K11];1	P51003	P51003 [652-662]	P51003 2xTMT6plex [K	0,01	-1,32	0,12
[R],KDSAQDDQAK,[Q]	2xTMT6plex [K1; K10];1	Q13045	Q13045 [434-443]	Q13045 2xTMT6plex [K	0,05	-1,32	0,16
[R],HSNLMLEELDK,[A]	1xTMT6plex [K11];1xTI	O43491	O43491 [626-636]	O43491 1xTMT6plex [K	0,01	-1,31	0,12
[RK],SKSSSVSR,[S]	1xTMT6plex [K2];1xTM	Q9BRL6;	Q9BRL6 [232-239]; Q01130 [171-178	Q9BRL6 1xTMT6plex [I	0,01	-1,31	0,12
[R],TPPKSPSSAK,[S]	2xTMT6plex [K4; K10];1	P10636	P10636 [548-557]	P10636 2xTMT6plex [K	0,01	-1,31	0,12
[R],KEDQDRSPR,[R]	1xTMT6plex [K1];1xTM	O60318	O60318 [294-302]	O60318 1xTMT6plex [K	0,02	-1,31	0,13
[R],KSSSISEEK,[G]	2xTMT6plex [K1; K9];1x	Q9UKV3	Q9UKV3 [205-213]	Q9UKV3 2xTMT6plex [I	0,01	-1,31	0,12
[R],THSGSSGGSGSR,[E]	1xTMT6plex [N-Term];1	Q8IZP0	Q8IZP0 [238-249]	Q8IZP0 1xTMT6plex [N	0,04	-1,30	0,15
[R],EGGSKSDSEDITR,[S]	1xTMT6plex [K5];1xTM	O00470	O00470 [191-203]	O00470 1xTMT6plex [K	0,00	-1,30	0,11
[R],SFSDVHTMDLGK,[H]	1xTMT6plex [K12];1xTI	Q7Z6M1	Q7Z6M1 [61-72]	Q7Z6M1 1xTMT6plex [I	0,01	-1,29	0,12
[K],GQSQTSPDHR,[S]	1xTMT6plex [N-Term];1	Q9UQ35	Q9UQ35 [1059-1068]	Q9UQ35 1xTMT6plex [I	0,01	-1,28	0,12
[R],SPHHSTVK,[V]	1xTMT6plex [K8];1xTM	Q8TAD8	Q8TAD8 [99-106]	Q8TAD8 1xTMT6plex [I	0,01	-1,28	0,12
[R],DKDSGSSSPLPK,[Y]	2xTMT6plex [K2; K12];1	Q86VQ1	Q86VQ1 [391-402]	Q86VQ1 2xTMT6plex [I	0,01	-1,28	0,12
[R],KSPENTEGKDGSK,[V]	3xTMT6plex [K1; K9; K	Q15651	Q15651 [5-17]	Q15651 3xTMT6plex [K	0,02	-1,27	0,12
[R],KPAMTTPTR,[R]	1xTMT6plex [K1];1xTM	Q99590	Q99590 [313-321]	Q99590 1xTMT6plex [K	0,01	-1,27	0,12
[R],QLSKPLSER,[T]	1xTMT6plex [K4];1xTM	Q6VY07	Q6VY07 [517-525]	Q6VY07 1xTMT6plex [I	0,04	-1,26	0,15
[K],KLDASILEDR,[D]	1xTMT6plex [K1];1xTM	Q92785	Q92785 [196-205]	Q92785 1xTMT6plex [K	0,01	-1,26	0,12
[R],VSPAGGTLDDKPK,[E]	2xTMT6plex [K11; K13]	Q8WY36	Q8WY36 [843-855]	Q8WY36 2xTMT6plex [	0,02	-1,25	0,13
[K],KEPDDSR,[D]	1xTMT6plex [K1];1xTM	Q5SSJ5	Q5SSJ5 [426-432]	Q5SSJ5 1xTMT6plex [K	0,03	-1,25	0,14
[R],SLSFSEPQQPAPAMK,[S]	1xTMT6plex [K15];1xTI	Q9H8N7	Q9H8N7 [447-461]	Q9H8N7 1xTMT6plex [I	0,00	-1,25	0,12
[R],YSNVIQPSSFSK,[S]	1xTMT6plex [K12];1xTI	O15504	O15504 [54-65]	O15504 1xTMT6plex [K	0,02	-1,25	0,13
[R],IDISPSTLR,[K]	1xTMT6plex [N-Term];1	Q9NYF8	Q9NYF8 [655-663]	Q9NYF8 1xTMT6plex [N	0,01	-1,24	0,12
[R],LSLGASR,[G]	1xTMT6plex [N-Term];1	O43318	O43318 [366-372]	O43318 1xTMT6plex [N	0,03	-1,23	0,13
[R],VHSPCPTSGSEK,[K]	1xCarbamidomethyl [C5	Q96LT9	Q96LT9 [106-117]	Q96LT9 1xCarbamidom	0,02	-1,23	0,13
[K],GQSQTSPDHR,[S]	1xTMT6plex [N-Term];1	Q9UQ35	Q9UQ35 [1059-1068]	Q9UQ35 1xTMT6plex [I	0,02	-1,22	0,12
[R],VLHSGSR,[D]	1xTMT6plex [N-Term];1	Q5T200	Q5T200 [1227-1233]	Q5T200 1xTMT6plex [N	0,03	-1,22	0,14
[R],DKGSPAVK,[A]	2xTMT6plex [K2; K8];1x	O15119	O15119 [406-413]	O15119 2xTMT6plex [K	0,01	-1,22	0,12

[R],KSVQPTSEER,[I]	1xTMT6plex [K1];1xTM	Q9UHD8	Q9UHD8 [326-335]	Q9UHD8 1xTMT6plex [	0,00	-1,22	0,12
[R],TPSNTPSAEADWSPGLELH	1xTMT6plex [K23];1xTM	Q9Y3Z3	Q9Y3Z3 [21-43]	Q9Y3Z3 1xTMT6plex [K	0,02	-1,22	0,13
[K],KLDPAQSASR,[E]	1xTMT6plex [K1];1xTM	Q13813	Q13813 [1023-1032]	Q13813 1xTMT6plex [K	0,04	-1,22	0,15
[R],HELSPPQK,[R]	1xTMT6plex [K8];1xTM	Q9BXP5	Q9BXP5 [71-78]	Q9BXP5 1xTMT6plex [I	0,02	-1,22	0,12
[R],KSFLHEQEENVVK,[I]	2xTMT6plex [K1; K13];1	Q13576	Q13576 [684-696]	Q13576 2xTMT6plex [K	0,01	-1,22	0,12
[R],HSPLSQR,[H]	1xTMT6plex [N-Term];1	A7MCY6	A7MCY6 [334-340]	A7MCY6 1xTMT6plex [I	0,02	-1,21	0,13
[K],SKPAGSDGER,[R]	1xTMT6plex [K2];1xTM	Q1KMD3	Q1KMD3 [188-197]	Q1KMD3 1xTMT6plex [	0,01	-1,21	0,12
[R],SLVGGLLQSK,[F]	1xTMT6plex [K10];1xTM	P52948	P52948 [1043-1052]	P52948 1xTMT6plex [K	0,01	-1,21	0,12
[R],KSQENLGNPSK,[N]	2xTMT6plex [K1; K11];1	Q9H0X4	Q9H0X4 [20-30]	Q9H0X4 2xTMT6plex [K	0,01	-1,21	0,12
[R],ASFLGGR,[R]	1xTMT6plex [N-Term];1	Q6UXT9	Q6UXT9 [433-439]	Q6UXT9 1xTMT6plex [N	0,00	-1,21	0,12
[R],KESTDEILGR,[S]	1xTMT6plex [K1];1xTM	Q15042	Q15042 [338-347]	Q15042 1xTMT6plex [K	0,01	-1,21	0,12
[K],LEPGGFGEKPVRPGGGDT	1xTMT6plex [K9];1xTM	Q5JSZ5	Q5JSZ5 [1435-1455]	Q5JSZ5 1xTMT6plex [K	0,00	-1,20	0,12
[R],TDVKSQDVAVSPQQQQCS	1xCarbamidomethyl [C1	Q96F86	Q96F86 [121-139]	Q96F86 1xCarbamidom	0,00	-1,20	0,12
[K],RKPEEESPR,[K]	1xTMT6plex [K2];1xTM	P49321	P49321 [720-728]	P49321 1xTMT6plex [K	0,01	-1,20	0,12
[R],KPSPEELDK,[G]	2xTMT6plex [K1; K9];1x	Q9BSL1	Q9BSL1 [330-338]	Q9BSL1 2xTMT6plex [K	0,02	-1,20	0,13
[R],TSSPHKEESPK,[K]	2xTMT6plex [K6; K11];1	P51003	P51003 [652-662]	P51003 2xTMT6plex [K	0,01	-1,20	0,12
[R],SSPVTKR,[R]	1xTMT6plex [K6];1xTM	Q8IYB3	Q8IYB3 [645-651]	Q8IYB3 1xTMT6plex [K	0,01	-1,20	0,12
[R],DGLAPEKTSPDR,[D]	1xTMT6plex [K7];1xTM	Q7L4I2	Q7L4I2 [9-20]	Q7L4I2 1xTMT6plex [K1	0,00	-1,20	0,11
[R],TLSALLTGR,[A]	1xTMT6plex [N-Term];1	Q8TER5	Q8TER5 [1406-1414]	Q8TER5 1xTMT6plex [N	0,02	-1,20	0,13
[R],HSLASTDEK,[R]	1xTMT6plex [K9];1xTM	Q07954	Q07954 [4519-4527]	Q07954 1xTMT6plex [K	0,04	-1,20	0,15
[R],SLSSSGTK,[H]	1xTMT6plex [K8];1xTM	Q9NZJ5	Q9NZJ5 [1094-1101]	Q9NZJ5 1xTMT6plex [K	0,02	-1,20	0,12
[R],KTPVASDQR,[R]	1xTMT6plex [K1];1xTM	P46013	P46013 [587-595]	P46013 1xTMT6plex [K	0,01	-1,19	0,12
[R],KGPGQPSSPQR,[L]	1xTMT6plex [K1];1xTM	O95400	O95400 [188-198]	O95400 1xTMT6plex [K	0,00	-1,19	0,12
[R],GSFGAMDDPFK,[N]	1xTMT6plex [K11];1xTM	Q9UBC2	Q9UBC2 [592-602]	Q9UBC2 1xTMT6plex [I	0,02	-1,19	0,12
[R],RSPESHR,[R]	1xTMT6plex [N-Term];1	Q86VQ1	Q86VQ1 [130-136]	Q86VQ1 1xTMT6plex [N	0,02	-1,19	0,13
[R],SPPPVSK,[R]	1xTMT6plex [K7];1xTM	Q01130	Q01130 [191-197]	Q01130 1xTMT6plex [K	0,01	-1,19	0,12
[R],RSSDPHR,[L]	1xTMT6plex [N-Term];1	Q5VUA4	Q5VUA4 [669-675]	Q5VUA4 1xTMT6plex [I	0,01	-1,18	0,12
[R],QSVIFHK,[R]	1xTMT6plex [K7];1xTM	P20309	P20309 [577-583]	P20309 1xTMT6plex [K	0,02	-1,18	0,13
[M],SSSPVKR,[Q]	1xTMT6plex [K6];1xTM	P37837	P37837 [2-8]	P37837 1xTMT6plex [K	0,00	-1,18	0,12
[R],LGGPSALPK,[G]	1xTMT6plex [K9];1xTM	Q96CX6	Q96CX6 [103-111]	Q96CX6 1xTMT6plex [K	0,02	-1,18	0,12
[R],TPPKSPATPK,[Q]	2xTMT6plex [K4; K10];1	P11137	P11137 [1649-1658]	P11137 2xTMT6plex [K	0,00	-1,18	0,11
[R],DKSDSDTEGLLFSR,[D]	1xTMT6plex [K2];1xTM	Q9H4G0	Q9H4G0 [646-659]	Q9H4G0 1xTMT6plex [I	0,01	-1,17	0,12
[R],GGSGYHSR,[S]	1xTMT6plex [N-Term];1	Q9UQ35	Q9UQ35 [1816-1823]	Q9UQ35 1xTMT6plex [I	0,02	-1,17	0,12
[K],HKEEDKNSER,[I]	2xTMT6plex [K2; K6];1x	Q9NYF8	Q9NYF8 [478-487]	Q9NYF8 2xTMT6plex [I	0,01	-1,17	0,12
[R],LSAGLYR,[Q]	1xTMT6plex [N-Term];1	Q13506	Q13506 [394-400]	Q13506 1xTMT6plex [N	0,04	-1,16	0,15
[R],SLSSTSSGSK,[D]	1xTMT6plex [K10];1xTM	P23508	P23508 [681-690]	P23508 1xTMT6plex [K	0,01	-1,16	0,12
[R],KASVHSSGR,[G]	1xTMT6plex [K1];1xTM	Q9Y5B9	Q9Y5B9 [1021-1029]	Q9Y5B9 1xTMT6plex [K	0,03	-1,16	0,14
[R],FPESPKR,[F]	1xTMT6plex [K6];1xTM	Q6UB99	Q6UB99 [1987-1993]	Q6UB99 1xTMT6plex [K	0,01	-1,15	0,12
[R],FSPTMGR,[H]	1xTMT6plex [N-Term];1	P78344	P78344 [394-400]	P78344 1xTMT6plex [N	0,03	-1,15	0,14
[R],LQGQLQGGSR,[E]	1xTMT6plex [N-Term];1	Q9UP83	Q9UP83 [189-198]	Q9UP83 1xTMT6plex [N	0,04	-1,15	0,15
[R],EFLPTSWSPVGAGPTPSLY	1xTMT6plex [K20];1xTM	Q9BWH6	Q9BWH6 [596-615]	Q9BWH6 1xTMT6plex [	0,02	-1,15	0,13
[R],NQDLAPNSAEQASILSLVTK	1xTMT6plex [K20];1xTM	Q12905	Q12905 [61-80]	Q12905 1xTMT6plex [K	0,02	-1,15	0,12
[R],IDISPSTLR,[K]	1xTMT6plex [N-Term];1	Q9NYF8	Q9NYF8 [655-663]	Q9NYF8 1xTMT6plex [N	0,01	-1,15	0,12
[K],KQPASPDGR,[T]	1xTMT6plex [K1];1xTM	Q9P2R6	Q9P2R6 [590-598]	Q9P2R6 1xTMT6plex [K	0,04	-1,15	0,15
[R],GGSGYHSR,[S]	1xTMT6plex [N-Term];1	Q9UQ35	Q9UQ35 [1816-1823]	Q9UQ35 1xTMT6plex [I	0,04	-1,14	0,15
[R],SPAIGLNNLDK,[A]	1xTMT6plex [K11];1xTM	Q13576	Q13576 [396-406]	Q13576 1xTMT6plex [K	0,02	-1,14	0,13
[K],DSYYVYTQQELK,[E]	1xTMT6plex [K12];1xTM	Q9H7D0	Q9H7D0 [1286-1297]	Q9H7D0 1xTMT6plex [K	0,04	-1,14	0,15
[K],SKPAGSDGER,[R]	1xTMT6plex [K2];1xTM	Q1KMD3	Q1KMD3 [188-197]	Q1KMD3 1xTMT6plex [	0,03	-1,14	0,14
[K],KSDVEAIFSK,[Y]	2xTMT6plex [K1; K10];1	P07910	P07910 [30-39]	P07910 2xTMT6plex [K	0,05	-1,14	0,16
[R],HRPSPPATPPPKTR,[H]	1xTMT6plex [K12];1xTM	Q8IYB3	Q8IYB3 [399-412]	Q8IYB3 1xTMT6plex [K	0,00	-1,13	0,11
[R],KPNAGGSPAPVR,[R]	1xTMT6plex [K1];1xTM	Q3KQU3	Q3KQU3 [393-404]	Q3KQU3 1xTMT6plex [	0,02	-1,13	0,13
[R],TPPKSPATPK,[Q]	2xTMT6plex [K4; K10];1	P11137	P11137 [1649-1658]	P11137 2xTMT6plex [K	0,02	-1,13	0,12
[R],SSNLVVSHSR,[Y]	1xTMT6plex [N-Term];1	Q6PJI9	Q6PJI9 [772-781]	Q6PJI9 1xTMT6plex [N	0,01	-1,13	0,12
[K],GGTPAGSAR,[G]	1xTMT6plex [N-Term];1	Q14195	Q14195 [512-520]	Q14195 1xTMT6plex [N	0,03	-1,13	0,14
[R],LEAIETQDPSLGCGLPLNCT	2xCarbamidomethyl [C1	Q9NVP2	Q9NVP2 [160-182]	Q9NVP2 2xCarbamidor	0,03	-1,13	0,14
[R],RSSLEKHR,[K]	1xTMT6plex [K6];1xTM	P11137	P11137 [1419-1426]	P11137 1xTMT6plex [K	0,00	-1,13	0,11
[R],MSPKPELTEEQK,[Q]	2xTMT6plex [K4; K12];1	P41208	P41208 [19-30]	P41208 2xTMT6plex [K	0,02	-1,13	0,13

[R],IPHSPAR,[E]	1xTMT6plex [N-Term];1	Q13136	Q13136 [705-711]	Q13136 1xTMT6plex [N	0,01	-1,12	0,12
[R],SPSDLHISPLAK,[K]	1xTMT6plex [K12];1xTN	O95785	O95785 [1127-1138]	O95785 1xTMT6plex [K	0,01	-1,12	0,12
[R],LSPIPEEVPK,[S]	1xTMT6plex [K10];1xTN	Q96T23	Q96T23 [603-612]	Q96T23 1xTMT6plex [K	0,04	-1,12	0,15
[R],RSPVSSSK,[G]	1xTMT6plex [K8];1xTM	Q14686	Q14686 [1808-1815]	Q14686 1xTMT6plex [K	0,02	-1,12	0,13
[R],SVDEVNYWDK,[Q]	1xTMT6plex [K10];1xTN	P12694	P12694 [347-356]	P12694 1xTMT6plex [K	0,01	-1,12	0,12
[R],VSPAAAQLADTLFSK,[G]	1xTMT6plex [K15];1xTN	O14686	O14686 [4358-4372]	O14686 1xTMT6plex [K	0,02	-1,11	0,13
[R],KAEPHSFR,[E]	1xTMT6plex [K1];1xTM	Q63HR2	Q63HR2 [28-35]	Q63HR2 1xTMT6plex [K	0,01	-1,11	0,12
[R],AASPTPK,[Q]	1xTMT6plex [K7];1xTM	Q9UPA5	Q9UPA5 [91-97]	Q9UPA5 1xTMT6plex [I	0,00	-1,11	0,10
[R],AVVVSPK,[E]	1xTMT6plex [K7];1xTM	O75909	O75909 [336-342]	O75909 1xTMT6plex [K	0,03	-1,11	0,14
[R],NFDTLDLPK,[R]	1xTMT6plex [K9];1xTM	O00192	O00192 [639-647]	O00192 1xTMT6plex [K	0,02	-1,11	0,13
[R],KESRPESIIIYR,[S]	1xTMT6plex [K1];1xTM	Q96HH4	Q96HH4 [45-56]	Q96HH4 1xTMT6plex [K	0,01	-1,11	0,12
[K],SHSHTPSR,[R]	1xTMT6plex [N-Term];1	Q9UQ35	Q9UQ35 [472-479]	Q9UQ35 1xTMT6plex [N	0,03	-1,11	0,14
[R],LAGTSGSDK,[G]	1xTMT6plex [K9];1xTM	Q9P016	Q9P016 [8-16]	Q9P016 1xTMT6plex [K	0,03	-1,11	0,14
[K],SVLTPLR,[G]	1xTMT6plex [N-Term];1	O75152	O75152 [576-582]	O75152 1xTMT6plex [N	0,01	-1,11	0,12
[R],KRTEEPDR,[D]	1xTMT6plex [K1];1xTM	O95232	O95232 [236-243]	O95232 1xTMT6plex [K	0,03	-1,10	0,13
[R],IDSLSAQLSQLQK,[Q]	1xTMT6plex [K13];1xTN	P02545	P02545 [299-311]	P02545 1xTMT6plex [K	0,02	-1,10	0,12
[R],LSPENNQVLTK,[K]	1xTMT6plex [K11];1xTN	Q16514	Q16514 [50-60]	Q16514 1xTMT6plex [K	0,02	-1,10	0,13
[R],NEFHSPIGLTRPSPDERESI	1xTMT6plex [K23];1xTN	Q06413	Q06413 [441-463]	Q06413 1xTMT6plex [K	0,02	-1,10	0,12
[K],KSEAPSGESR,[K]	1xTMT6plex [K1];1xTM	B5ME19	B5ME19 [200-209]	B5ME19 1xTMT6plex [K	0,01	-1,09	0,12
[R],KYQEDSDPER,[S]	1xTMT6plex [K1];1xTM	Q6P6C2	Q6P6C2 [59-68]	Q6P6C2 1xTMT6plex [K	0,01	-1,09	0,12
[R],SPSTPAK,[S]	1xTMT6plex [K7];1xTM	Q9BQG0	Q9BQG0 [1241-1247]	Q9BQG0 1xTMT6plex [	0,01	-1,09	0,12
[R],KSSTVMK,[E]	2xTMT6plex [K1; K7];1x	Q15139	Q15139 [419-425]	Q15139 2xTMT6plex [K	0,04	-1,09	0,15
[R],SPACSSLTPSLCK,[L]	2xCarbamidomethyl [C4	Q96R06	Q96R06 [43-55]	Q96R06 2xCarbamidom	0,00	-1,09	0,11
[R],SPACSSLTPSLCK,[L]	2xCarbamidomethyl [C4	Q96R06	Q96R06 [43-55]	Q96R06 2xCarbamidom	0,00	-1,09	0,11
[R],SPHEAGFCVYLK,[G]	1xCarbamidomethyl [C8	Q9NTZ6	Q9NTZ6 [424-435]	Q9NTZ6 1xCarbamidon	0,01	-1,09	0,12
[R],KSELEFETLK,[T]	2xTMT6plex [K1; K10];1	Q5JSH3	Q5JSH3 [261-270]	Q5JSH3 2xTMT6plex [K	0,02	-1,08	0,13
[K],VAHSDKPGSTSTASFR,[D]	1xTMT6plex [K6];1xTM	Q9P0L0	Q9P0L0 [206-221]	Q9P0L0 1xTMT6plex [K	0,01	-1,08	0,12
[R],KHSPSPPPPTPTESR,[K]	1xTMT6plex [K1];1xTM	Q92922	Q92922 [326-340]	Q92922 1xTMT6plex [K	0,00	-1,08	0,11
[R],KAENQNESSQGK,[S]	2xTMT6plex [K1; K12];1	Q9UKI8	Q9UKI8 [126-137]	Q9UKI8 2xTMT6plex [K	0,03	-1,08	0,14
[R],MSPKPELTEEQK,[Q]	1xOxidation [M1];2xTM	P41208	P41208 [19-30]	P41208 1xOxidation [M	0,01	-1,08	0,12
[R],KAASLTEDR,[D]	1xTMT6plex [K1];1xTM	Q04637	Q04637 [1206-1214]	Q04637 1xTMT6plex [K	0,00	-1,07	0,11
[K],TSASDVTNIYPGDAGK,[A]	1xTMT6plex [K16];1xTN	Q15042	Q15042 [536-551]	Q15042 1xTMT6plex [K	0,02	-1,07	0,12
[R],QSSSHLAK,[G]	1xTMT6plex [K8];1xTM	Q9Y5W9	Q9Y5W9 [167-174]	Q9Y5W9 1xTMT6plex [	0,02	-1,07	0,13
[K],IGTLQDRPPSPFR,[E]	1xTMT6plex [N-Term];1	Q86UU1	Q86UU1 [395-407]	Q86UU1 1xTMT6plex [N	0,00	-1,07	0,12
[R],EQSSDLTPSGDVSPVKPLS	1xTMT6plex [K16];1xTN	Q8WYQ5	Q8WYQ5 [365-384]	Q8WYQ5 1xTMT6plex	0,00	-1,07	0,11
[R],RKESSDNQENR,[G]	1xTMT6plex [K2];1xTM	P50221	P50221 [152-162]	P50221 1xTMT6plex [K	0,03	-1,07	0,13
[R],SPHEVFGCVVPEGGSQAA	1xCarbamidomethyl [C8	Q86X51	Q86X51 [105-127]	Q86X51 1xCarbamidom	0,01	-1,06	0,12
[R],SPLQSAESSPTAGK,[K]	1xTMT6plex [K14];1xTN	Q8IWB9	Q8IWB9 [794-807]	Q8IWB9 1xTMT6plex [K	0,01	-1,06	0,12
[R],KLEDTWAYCQDTRK,[G]	1xCarbamidomethyl [C9	Q6ZV65	Q6ZV65 [154-167]	Q6ZV65 1xCarbamidom	0,05	-1,06	0,16
[R],TSSPHKEESPKK,[T]	3xTMT6plex [K6; K11; K	P51003	P51003 [652-663]	P51003 3xTMT6plex [K	0,02	-1,06	0,13
[R],LRSPAQYQVVLSER,[L]	1xTMT6plex [N-Term];1	Q6XQN6	Q6XQN6 [511-524]	Q6XQN6 1xTMT6plex [	0,02	-1,06	0,13
[R],IDISPSTLR,[K]	1xTMT6plex [N-Term];1	Q9NYF8	Q9NYF8 [655-663]	Q9NYF8 1xTMT6plex [N	0,02	-1,06	0,12
[R],GPDSKDSPK,[D]	2xTMT6plex [K5; K9];1x	Q9BUA3	Q9BUA3 [286-294]	Q9BUA3 2xTMT6plex [K	0,03	-1,06	0,14
[R],ASGLVGR,[K]	1xTMT6plex [N-Term];1	Q9UH99	Q9UH99 [115-121]	Q9UH99 1xTMT6plex [N	0,01	-1,06	0,12
[K],VLGTPTR,[E]	1xTMT6plex [N-Term];1	P49840; P	P49840 [335-341]; P49841 [272-278]	P49840 1xTMT6plex [N	0,01	-1,06	0,12
[R],ELQQAELPNRSPGFGIK,[K]	1xTMT6plex [K17];1xTN	Q6NWY9	Q6NWY9 [822-838]	Q6NWY9 1xTMT6plex [	0,00	-1,05	0,12
[K],SPKTVSPPIR,[K]	1xTMT6plex [K3];1xTM	Q9UHB6	Q9UHB6 [604-613]	Q9UHB6 1xTMT6plex [K	0,00	-1,05	0,12
[RK],SPSLSSK,[HQ]	1xTMT6plex [K7];1xTM	Q8IYB3; Q	Q8IYB3 [653-659]; Q9UPU5 [1141-1	Q8IYB3 1xTMT6plex [K	0,01	-1,05	0,12
[R],SPTVLCQK,[V]	1xCarbamidomethyl [C6	Q5VUA4	Q5VUA4 [2035-2042]	Q5VUA4 1xCarbamidon	0,02	-1,05	0,12
[R],KSLLALEK,[E]	2xTMT6plex [K1; K8];1x	Q9Y4D1	Q9Y4D1 [117-124]	Q9Y4D1 2xTMT6plex [K	0,02	-1,05	0,12
[K],TSPDKQK,[Q]	1xMethyl [K5];1xTMT6p	P20807	P20807 [628-634]	P20807 1xMethyl [K632	0,03	-1,04	0,14
[R],STPPYLFPEYPEASK,[N]	1xTMT6plex [K15];1xTN	Q8TE76	Q8TE76 [620-634]	Q8TE76 1xTMT6plex [K	0,02	-1,04	0,12
[R],LSAEYEK,[V]	1xTMT6plex [K7];1xTM	Q02952	Q02952 [370-376]	Q02952 1xTMT6plex [K	0,04	-1,03	0,15
[R],KESSNTDSAGALGTLR,[F]	1xTMT6plex [K1];1xTM	P29474	P29474 [631-646]	P29474 1xTMT6plex [K	0,02	-1,03	0,13
[R],KENETPR,[R]	1xTMT6plex [K1];1xTM	Q14966	Q14966 [479-485]	Q14966 1xTMT6plex [K	0,02	-1,03	0,12
[R],SPVVELSK,[V]	1xTMT6plex [K8];1xTM	P25098	P25098 [670-677]	P25098 1xTMT6plex [K	0,01	-1,03	0,12
[R],YQEGPVRLSPGR,[L]	1xTMT6plex [N-Term];1	P50747	P50747 [291-302]	P50747 1xTMT6plex [N	0,05	-1,03	0,16

[R],SLVQGELVTASK,[A]	1xTMT6plex [K12];1xTM	P35611	P35611 [532-543]	P35611 1xTMT6plex [K	0,05	-1,03	0,16
[R],QKSLTLTPTR,[S]	1xTMT6plex [K2];1xTM	Q3V6T2	Q3V6T2 [1415-1424]	Q3V6T2 1xTMT6plex [K	0,00	-1,03	0,12
[R],GALSSSLR,[D]	1xTMT6plex [N-Term];1	Q5JU85	Q5JU85 [1138-1145]	Q5JU85 1xTMT6plex [N	0,04	-1,03	0,15
[R],GSTGSLK,[S]	1xTMT6plex [K7];1xTM	Q9P1Y5	Q9P1Y5 [372-378]	Q9P1Y5 1xTMT6plex [K	0,01	-1,03	0,12
[R],HFSEHPSTSK,[M]	1xTMT6plex [K10];1xTM	O43719	O43719 [401-410]	O43719 1xTMT6plex [K	0,01	-1,03	0,12
[R],AISPSIK,[E]	1xTMT6plex [K7];1xTM	Q8N201	Q8N201 [102-108]	Q8N201 1xTMT6plex [K	0,04	-1,02	0,15
[R],KPASPGR,[T]	1xTMT6plex [K1];1xTM	Q9P2Y4	Q9P2Y4 [608-614]	Q9P2Y4 1xTMT6plex [K	0,03	-1,02	0,13
[R],LGSASSSHGSIQESHKASR	1xTMT6plex [K16];1xTM	Q16566	Q16566 [339-357]	Q16566 1xTMT6plex [K	0,01	-1,02	0,12
[R],RSSLGAK,[M]	1xTMT6plex [K7];1xTM	Q9UJD0	Q9UJD0 [46-52]	Q9UJD0 1xTMT6plex [K	0,02	-1,02	0,13
[K],TSPIQSK,[S]	1xTMT6plex [K7];1xTM	O43903	O43903 [281-287]	O43903 1xTMT6plex [K	0,03	-1,02	0,14
[R],TLNAETPKSSPLPAKGR,[D]	2xTMT6plex [K8; K15];1	Q7L2J0	Q7L2J0 [208-224]	Q7L2J0 2xTMT6plex [K	0,01	-1,01	0,12
[R],KTPVSEAR,[K]	1xTMT6plex [K1];1xTM	Q92540	Q92540 [623-630]	Q92540 1xTMT6plex [K	0,01	-1,01	0,12
[R],RPTPSTK,[-]	1xTMT6plex [K7];1xTM	Q9UPN4	Q9UPN4 [1077-1083]	Q9UPN4 1xTMT6plex [	0,00	-1,01	0,12
[R],EDSGTFSLGK,[M]	1xTMT6plex [K10];1xTM	O75569	O75569 [16-25]	O75569 1xTMT6plex [K	0,01	-1,01	0,12
[R],FSSSSNLK,[T]	1xTMT6plex [K8];1xTM	Q8IZ20	Q8IZ20 [407-414]	Q8IZ20 1xTMT6plex [K	0,01	-1,01	0,12
[R],KHSPSPPPPTPTESR,[K]	1xTMT6plex [K1];1xTM	Q92922	Q92922 [326-340]	Q92922 1xTMT6plex [K	0,00	-1,00	0,12
[R],KASVHSSGR,[G]	1xTMT6plex [K1];1xTM	Q9Y5B9	Q9Y5B9 [1021-1029]	Q9Y5B9 1xTMT6plex [K	0,02	-1,00	0,13
[R],KRDHSNNDR,[E]	1xTMT6plex [K1];1xTM	Q8NI27	Q8NI27 [1482-1490]	Q8NI27 1xTMT6plex [K	0,00	-1,00	0,12
[R],KAENPSPR,[S]	1xTMT6plex [K1];1xTM	Q9NZJ0	Q9NZJ0 [671-678]	Q9NZJ0 1xTMT6plex [K	0,01	-1,00	0,12
[R],KLSLSEGK,[V]	2xTMT6plex [K1; K8];1x	Q49A26	Q49A26 [128-135]	Q49A26 2xTMT6plex [K	0,03	-1,00	0,13
[R],IDISPSTFR,[K]	1xTMT6plex [N-Term];1	Q9Y2W1	Q9Y2W1 [679-687]	Q9Y2W1 1xTMT6plex [	0,02	-1,00	0,12
[R],SSHKDSPR,[D]	1xTMT6plex [K4];1xTM	O15042	O15042 [969-976]	O15042 1xTMT6plex [K	0,01	-1,00	0,12
[R],ITPEFSK,[W]	1xTMT6plex [K7];1xTM	Q5VTB9	Q5VTB9 [445-451]	Q5VTB9 1xTMT6plex [K	0,03	-1,00	0,13
[R],SRSPDHR,[R]	1xTMT6plex [N-Term];1	Q9NVW2	Q9NVW2 [213-219]	Q9NVW2 1xTMT6plex [	0,03	-1,00	0,13
[R],RKESSDNQENR,[G]	1xTMT6plex [K2];1xTM	P50221	P50221 [152-162]	P50221 1xTMT6plex [K	0,03	-1,00	0,13
[R],KASDVHEVR,[K]	1xTMT6plex [K1];1xTM	P14618	P14618 [247-255]	P14618 1xTMT6plex [K	0,02	-0,99	0,12
[K],QKSQTPPR,[R]	1xTMT6plex [K2];1xTM	Q9UQ35	Q9UQ35 [788-795]	Q9UQ35 1xTMT6plex [	0,04	-0,99	0,15
[R],GSYGDLGGPIITTQVTIPK,[	1xTMT6plex [K19];1xTM	P61978	P61978 [378-396]	P61978 1xTMT6plex [K	0,02	-0,99	0,13
[R],GGSTVPK,[D]	1xTMT6plex [K7];1xTM	Q92504	Q92504 [291-297]	Q92504 1xTMT6plex [K	0,02	-0,99	0,13
[R],KCSQTQCPR,[K]	2xCarbamidomethyl [C2	P29590	P29590 [478-486]	P29590 2xCarbamidom	0,04	-0,99	0,15
[R],LGPPPSHASADATR,[C]	1xTMT6plex [N-Term];1	Q2TAK8	Q2TAK8 [345-358]	Q2TAK8 1xTMT6plex [N	0,00	-0,99	0,12
[R],SPGRPPK,[S]	1xTMT6plex [K7];1xTM	Q14739	Q14739 [86-92]	Q14739 1xTMT6plex [K	0,02	-0,98	0,12
[K],FTPVASK,[F]	1xTMT6plex [K7];1xTM	Q15942	Q15942 [273-279]	Q15942 1xTMT6plex [K	0,04	-0,98	0,15
[K],ASGKTSQVGAASAPAKESF	2xTMT6plex [K4; K16];1	Q13428	Q13428 [364-383]	Q13428 2xTMT6plex [K	0,00	-0,98	0,11
[R],KDDDSDDESQSSHTGK,[K]	2xTMT6plex [K1; K16];1	O94874	O94874 [454-469]	O94874 2xTMT6plex [K	0,01	-0,98	0,12
[R],SVVSFDKVK,[E]	2xTMT6plex [K7; K9];1x	Q15424	Q15424 [601-609]	Q15424 2xTMT6plex [K	0,00	-0,97	0,12
[R],EIAQDFKTDLR,[F]	1xTMT6plex [K7];1xTM	P84243; Q	P84243 [74-84]; Q16695 [74-84]	P84243 1xTMT6plex [K	0,03	-0,97	0,13
[R],SLSPLGGR,[D]	1xTMT6plex [N-Term];1	Q14004	Q14004 [315-322]	Q14004 1xTMT6plex [N	0,01	-0,97	0,12
[R],SHSPVKR,[K]	1xTMT6plex [K6];1xTM	Q5VT06	Q5VT06 [565-571]	Q5VT06 1xTMT6plex [K	0,00	-0,97	0,12
[R],DKSHSHTPSR,[R]	1xTMT6plex [K2];1xTM	Q9UQ35	Q9UQ35 [470-479]	Q9UQ35 1xTMT6plex [	0,00	-0,97	0,12
[R],KGAAEEEQQDSGSEPR,[G	1xTMT6plex [K1];1xTM	Q9UJV9	Q9UJV9 [56-71]	Q9UJV9 1xTMT6plex [K	0,01	-0,97	0,12
[K],RKPSEEAR,[K]	1xTMT6plex [K2];1xTM	Q6UWE0	Q6UWE0 [8-15]	Q6UWE0 1xTMT6plex [	0,01	-0,97	0,12
[R],KLSEADNR,[K]	1xTMT6plex [K1];1xTM	Q9UNL2	Q9UNL2 [103-110]	Q9UNL2 1xTMT6plex [K	0,02	-0,97	0,13
[R],KSDSGVMLPTLR,[V]	1xTMT6plex [K1];1xTM	Q6ZUT9	Q6ZUT9 [832-843]	Q6ZUT9 1xTMT6plex [K	0,02	-0,97	0,12
[R],RPTLGVQLDDK,[R]	1xTMT6plex [K11];1xTM	Q13769	Q13769 [326-336]	Q13769 1xTMT6plex [K	0,01	-0,97	0,12
[R],KNSASAER,[D]	1xTMT6plex [K1];1xTM	Q96T37	Q96T37 [720-727]	Q96T37 1xTMT6plex [K	0,00	-0,97	0,12
[R],HELSPPQKR,[M]	1xTMT6plex [K8];1xTM	Q9BXP5	Q9BXP5 [71-79]	Q9BXP5 1xTMT6plex [K	0,02	-0,96	0,12
[K],TSPIQSK,[S]	1xTMT6plex [K7];1xTM	O43903	O43903 [281-287]	O43903 1xTMT6plex [K	0,03	-0,96	0,14
[K],KKSGPPAPEEEEEEER,[Q]	2xTMT6plex [K1; K2];1x	Q9NTI5	Q9NTI5 [1317-1332]	Q9NTI5 2xTMT6plex [K	0,01	-0,96	0,12
[R],SPRPAAGK,[E]	1xTMT6plex [K8];1xTM	Q9H0C8	Q9H0C8 [13-20]	Q9H0C8 1xTMT6plex [K	0,01	-0,96	0,12
[R],LSPPHSPR,[D]	1xTMT6plex [N-Term];1	Q92974	Q92974 [955-962]	Q92974 1xTMT6plex [N	0,04	-0,96	0,15
[R],SSFSHYSGLK,[H]	1xTMT6plex [K10];1xTM	Q8NC51	Q8NC51 [202-211]	Q8NC51 1xTMT6plex [K	0,02	-0,96	0,12
[R],TPPSSPK,[S]	1xTMT6plex [K7];1xTM	Q9BSE4	Q9BSE4 [90-96]	Q9BSE4 1xTMT6plex [K	0,02	-0,96	0,13
[R],SFAGNLNTYK,[R]	1xTMT6plex [K10];1xTM	Q01813	Q01813 [386-395]	Q01813 1xTMT6plex [K	0,01	-0,96	0,12
[R],DSLREQTLSK,[Q]	1xTMT6plex [K10];1xTM	Q6DN90	Q6DN90 [493-502]	Q6DN90 1xTMT6plex [K	0,03	-0,96	0,14
[R],ASPSKPASAPASR,[S]	1xTMT6plex [K5];1xTM	P27816	P27816 [786-798]	P27816 1xTMT6plex [K	0,00	-0,95	0,12
[R],SPVSSLQIR,[Y]	1xTMT6plex [N-Term];1	P55197	P55197 [689-697]	P55197 1xTMT6plex [N	0,01	-0,95	0,12

[R],LSIPSLTCNPDK,[T]	1xCarbamidomethyl [C8	Q4VCS5	Q4VCS5 [1040-1051]	Q4VCS5 1xCarbamidom	0,04	-0,95	0,15
[R],LSCFAQTVSPAEK,[W]	1xCarbamidomethyl [C3	Q16658	Q16658 [119-131]	Q16658 1xCarbamidom	0,02	-0,95	0,12
[R],ASDKDITVSK,[N]	2xTMT6plex [K4; K10];1	P49790	P49790 [191-200]	P49790 2xTMT6plex [K	0,00	-0,95	0,12
[R],SPFEHSVEHK,[S]	1xTMT6plex [K10];1xTM	O14646	O14646 [1689-1698]	O14646 1xTMT6plex [K	0,01	-0,95	0,12
[R],KESPSSTGSSTWK,[Q]	2xTMT6plex [K1; K13];1	Q6NV74	Q6NV74 [43-55]	Q6NV74 2xTMT6plex [K	0,02	-0,95	0,13
[R],SPSPKPTK,[V]	2xTMT6plex [K5; K8];1x	Q15287	Q15287 [155-162]	Q15287 2xTMT6plex [K	0,01	-0,95	0,12
[R],TSSPPRK,[T]	1xTMT6plex [K7];1xTM	Q8IYB3	Q8IYB3 [378-384]	Q8IYB3 1xTMT6plex [K	0,01	-0,94	0,12
[R],VDGLPSPK,[T]	1xTMT6plex [K8];1xTM	Q14209	Q14209 [112-119]	Q14209 1xTMT6plex [K	0,01	-0,94	0,12
[R],TKGSGQQSER,[G]	1xTMT6plex [K2];1xTM	Q8NDT2	Q8NDT2 [617-626]	Q8NDT2 1xTMT6plex [I	0,04	-0,94	0,15
[R],KPSSEEGK,[R]	2xTMT6plex [K1; K8];1x	Q9Y613	Q9Y613 [355-362]	Q9Y613 2xTMT6plex [K	0,05	-0,94	0,16
[R],KSSRSPSPR,[S]	1xTMT6plex [K1];1xTM	Q8WXA9	Q8WXA9 [402-410]	Q8WXA9 1xTMT6plex [	0,04	-0,94	0,15
[K],ANVSSPHR,[E]	1xTMT6plex [N-Term];1	Q92530	Q92530 [149-156]	Q92530 1xTMT6plex [N	0,01	-0,94	0,12
[R],SKSDLSDR,[Y]	1xTMT6plex [K2];1xTM	Q8TC76	Q8TC76 [259-266]	Q8TC76 1xTMT6plex [K	0,01	-0,94	0,12
[R],SPFLKPDER,[A]	1xTMT6plex [K5];1xTM	Q9H7N4	Q9H7N4 [874-882]	Q9H7N4 1xTMT6plex [K	0,02	-0,94	0,13
[R],KSSTTLR,[E]	1xTMT6plex [K1];1xTM	Q9BZL6	Q9BZL6 [394-400]	Q9BZL6 1xTMT6plex [K	0,03	-0,93	0,13
[K],DTEEPLPVKESDQTLAALLS	2xTMT6plex [K9; K22];1	P18583	P18583 [1678-1699]	P18583 2xTMT6plex [K	0,02	-0,93	0,13
[R],TWTLCGTPEYLAPEIILSKG	1xCarbamidomethyl [C5	P17612	P17612 [196-218]	P17612 1xCarbamidom	0,00	-0,93	0,11
[R],EKQEASESPVK,[A]	2xTMT6plex [K2; K11];1	Q01167	Q01167 [163-173]	Q01167 2xTMT6plex [K	0,00	-0,93	0,11
[K],KSESPFR,[E]	1xTMT6plex [K1];1xTM	Q9BYW2	Q9BYW2 [741-747]	Q9BYW2 1xTMT6plex [	0,01	-0,93	0,12
[R],DQSPFSK,[I]	1xTMT6plex [K7];1xTM	Q13427	Q13427 [685-691]	Q13427 1xTMT6plex [K	0,01	-0,93	0,12
[R],SVSHPGSCSSER,[S]	1xCarbamidomethyl [C8	Q9HB20	Q9HB20 [209-220]	Q9HB20 1xCarbamidom	0,00	-0,93	0,12
[R],INVYYNEATGGK,[Y]	1xTMT6plex [K12];1xTM	P68371	P68371 [47-58]	P68371 1xTMT6plex [K	0,00	-0,93	0,12
[R],SLLLEPPRGTPDR,[S]	1xTMT6plex [N-Term];1	Q53GT1	Q53GT1 [596-608]	Q53GT1 1xTMT6plex [N	0,00	-0,93	0,12
[R],LSPAQGPPQPQSSAK,[K]	1xTMT6plex [K15];1xTM	A4D1P6	A4D1P6 [276-290]	A4D1P6 1xTMT6plex [K	0,02	-0,93	0,13
[K],KSQGAGSSPR,[R]	1xTMT6plex [K1];1xTM	Q96IQ9	Q96IQ9 [289-298]	Q96IQ9 1xTMT6plex [K	0,01	-0,93	0,12
[R],KPEEESPRK,[D]	2xTMT6plex [K1; K9];1x	P49321	P49321 [721-729]	P49321 2xTMT6plex [K	0,00	-0,93	0,12
[K],KEPSTVSR,[D]	1xTMT6plex [K1];1xTM	P11137	P11137 [1448-1455]	P11137 1xTMT6plex [K	0,01	-0,92	0,12
[R],SPSTSPK,[F]	1xTMT6plex [K7];1xTM	Q96SK2	Q96SK2 [147-153]	Q96SK2 1xTMT6plex [K	0,01	-0,92	0,12
[R],APSPTKR,[K]	1xTMT6plex [K6];1xTM	Q15287	Q15287 [25-31]	Q15287 1xTMT6plex [K	0,02	-0,92	0,12
[R],SPLLEVK,[G]	1xTMT6plex [K7];1xTM	Q9BW19	Q9BW19 [6-12]	Q9BW19 1xTMT6plex [	0,02	-0,92	0,13
[KT],YSPTSPK,[YG]	1xTMT6plex [K7];1xTM	P24928	P24928 [1860-1866]; [1867-1873]; [1	P24928 1xTMT6plex [K	0,01	-0,92	0,12
[R],LPSSPVYEDAASFK,[A]	1xTMT6plex [K14];1xTM	Q14247	Q14247 [415-428]	Q14247 1xTMT6plex [K	0,01	-0,92	0,12
[R],TSPAPWK,[R]	1xTMT6plex [K7];1xTM	Q9UQ35	Q9UQ35 [1856-1862]	Q9UQ35 1xTMT6plex [I	0,01	-0,91	0,12
[R],SISTLAQRPR,[G]	1xTMT6plex [N-Term];1	Q00975	Q00975 [2021-2030]	Q00975 1xTMT6plex [N	0,01	-0,91	0,12
[R],SGEQHSPAR,[E]	1xTMT6plex [N-Term];1	Q53HC0	Q53HC0 [273-281]	Q53HC0 1xTMT6plex [N	0,01	-0,91	0,12
[R],SSPVESLK,[K]	1xTMT6plex [K8];1xTM	Q9NRA8	Q9NRA8 [77-84]	Q9NRA8 1xTMT6plex [I	0,03	-0,91	0,13
[R],SPPADAIPKSK,[K]	2xTMT6plex [K9; K11];1	P18754	P18754 [11-21]	P18754 2xTMT6plex [K	0,00	-0,91	0,11
[R],SSFSHYSGLK,[H]	1xTMT6plex [K10];1xTM	Q8NC51	Q8NC51 [202-211]	Q8NC51 1xTMT6plex [K	0,04	-0,91	0,15
[R],LDPFADGGKTPDPK,[M]	2xTMT6plex [K9; K14];1	O75533	O75533 [133-146]	O75533 2xTMT6plex [K	0,02	-0,91	0,12
[R],KGSDDAPYSPTAR,[V]	1xTMT6plex [K1];1xTM	O75151	O75151 [897-909]	O75151 1xTMT6plex [K	0,00	-0,91	0,12
[R],AQTAPTK,[T]	1xTMT6plex [K7];1xTM	Q14669	Q14669 [1419-1425]	Q14669 1xTMT6plex [K	0,00	-0,90	0,12
[R],RSPSLSSK,[H]	1xTMT6plex [K8];1xTM	Q8IYB3	Q8IYB3 [652-659]	Q8IYB3 1xTMT6plex [K	0,02	-0,90	0,12
[R],TMIISPER,[L]	1xTMT6plex [N-Term];1	O75533	O75533 [125-132]	O75533 1xTMT6plex [N	0,02	-0,90	0,12
[R],FSDGAASIQAFK,[A]	1xTMT6plex [K12];1xTM	Q9Y2K2	Q9Y2K2 [625-636]	Q9Y2K2 1xTMT6plex [K	0,04	-0,90	0,16
[R],GRDSPYQSR,[G]	1xTMT6plex [N-Term];1	Q13242	Q13242 [201-209]	Q13242 1xTMT6plex [N	0,01	-0,90	0,12
[R],SPGPPPPVGVK,[T]	1xTMT6plex [K11];1xTM	Q9H1B7	Q9H1B7 [69-79]	Q9H1B7 1xTMT6plex [K	0,01	-0,90	0,12
[R],KMSFDIIDK,[S]	1xOxidation [M2];2xTMT	Q8IX01	Q8IX01 [313-321]	Q8IX01 1xOxidation [M2	0,05	-0,90	0,16
[R],SPSAPVCK,[A]	1xCarbamidomethyl [C7	Q6P1L5	Q6P1L5 [192-199]	Q6P1L5 1xCarbamidom	0,02	-0,90	0,12
[K],KSPSEAR,[Q]	1xTMT6plex [K1];1xTM	P46821	P46821 [2071-2077]	P46821 1xTMT6plex [K	0,01	-0,90	0,12
[K],LVSSPGR,[S]	1xTMT6plex [N-Term];1	Q9H6X5	Q9H6X5 [333-339]	Q9H6X5 1xTMT6plex [N	0,04	-0,90	0,15
[R],KGYIGTLGK,[N]	1xMethyl [K1];1xTMT6p	Q05BV3	Q05BV3 [854-862]	Q05BV3 1xMethyl [K854	0,05	-0,90	0,16
[K],VGPATPSAQVGK,[W]	1xTMT6plex [K12];1xTM	Q13428	Q13428 [529-540]	Q13428 1xTMT6plex [K	0,03	-0,90	0,14
[R],KISVVSATKGVPAGNSDTE	2xTMT6plex [K1; K9];1x	Q9UKV3	Q9UKV3 [823-847]	Q9UKV3 2xTMT6plex [I	0,00	-0,90	0,12
[R],KPSPEPEGEVGPPK,[I]	2xTMT6plex [K1; K14];1	Q7Z5L9	Q7Z5L9 [358-371]	Q7Z5L9 2xTMT6plex [K	0,02	-0,90	0,13
[R],RTDALTSSPGR,[D]	1xTMT6plex [N-Term];1	P49736	P49736 [34-44]	P49736 1xTMT6plex [N	0,01	-0,90	0,12
[R],DYGHSSSR,[D]	1xTMT6plex [N-Term];1	P38159; Q	P38159 [245-252]; Q96E39 [245-252	P38159 1xTMT6plex [N	0,04	-0,89	0,16
[R],KSDAGISSADAPR,[K]	1xTMT6plex [K1];1xTM	Q96FE5	Q96FE5 [601-613]	Q96FE5 1xTMT6plex [K	0,01	-0,89	0,12

[R],CSAGLGALAQRPGSVSK,[V	1xCarbamidomethyl [C1	Q9NXV2	Q9NXV2 [28-44]	Q9NXV2 1xCarbamidon	0,01	-0,89	0,12
[R],VQTPLLR,[A]	1xTMT6plex [N-Term];1	Q9UHD8	Q9UHD8 [47-53]	Q9UHD8 1xTMT6plex [	0,02	-0,89	0,12
[R],SRLSLGPSADR,[E]	1xTMT6plex [N-Term];1	Q9C0D6	Q9C0D6 [539-549]	Q9C0D6 1xTMT6plex [N	0,04	-0,89	0,15
[R],STTPAPK,[S]	1xTMT6plex [K7];1xTM	Q9UQ35	Q9UQ35 [250-256]	Q9UQ35 1xTMT6plex [I	0,01	-0,89	0,12
[R],SFSGLIK,[R]	1xTMT6plex [K7];1xTM	Q14CB8	Q14CB8 [422-428]	Q14CB8 1xTMT6plex [K	0,02	-0,89	0,13
[R],KASPEPPDSAEGALKLGEE	2xTMT6plex [K1; K15];1	Q9H1B7	Q9H1B7 [545-566]	Q9H1B7 2xTMT6plex [K	0,00	-0,89	0,11
[R],SSVQGASSR,[E]	1xTMT6plex [N-Term];1	O95817	O95817 [268-276]	O95817 1xTMT6plex [N	0,03	-0,89	0,14
[K],SASDSGCDPASK,[K]	1xCarbamidomethyl [C7	Q7LBC6	Q7LBC6 [289-300]	Q7LBC6 1xCarbamidon	0,00	-0,89	0,11
[R],NSFSFSDEK,[L]	1xTMT6plex [K9];1xTM	Q9H4X1	Q9H4X1 [86-94]	Q9H4X1 1xTMT6plex [K	0,02	-0,89	0,13
[K],VKSGTPPR,[Q]	1xTMT6plex [K2];1xTM	Q9UQ35	Q9UQ35 [844-851]	Q9UQ35 1xTMT6plex [I	0,00	-0,89	0,11
[K],GTRSSLSSK,[K]	1xTMT6plex [K9];1xTM	Q9H5V7	Q9H5V7 [168-176]	Q9H5V7 1xTMT6plex [K	0,03	-0,89	0,13
[R],KGPGQPSSPQR,[L]	1xTMT6plex [K1];1xTM	O95400	O95400 [188-198]	O95400 1xTMT6plex [K	0,00	-0,88	0,12
[RK],SPSLSSK,[HQ]	1xTMT6plex [K7];1xTM	Q8IYB3; (	Q8IYB3 [653-659]; Q9UPU5 [1141-1	Q8IYB3 1xTMT6plex [K	0,01	-0,88	0,12
[R],ERFSPPR,[H]	1xTMT6plex [N-Term];1	Q9BXP5	Q9BXP5 [64-70]	Q9BXP5 1xTMT6plex [N	0,01	-0,88	0,12
[R],SSTSSIDSNISSK,[S]	1xTMT6plex [K13];1xTM	Q8IVL1	Q8IVL1 [1232-1244]	Q8IVL1 1xTMT6plex [K	0,03	-0,88	0,14
[K],LSPSWNPK,[I]	1xTMT6plex [K8];1xTM	Q96BY7	Q96BY7 [254-261]	Q96BY7 1xTMT6plex [K	0,02	-0,88	0,13
[K],GKTPEEIR,[K]	1xTMT6plex [K2];1xTM	P63208	P63208 [129-136]	P63208 1xTMT6plex [K	0,02	-0,88	0,13
[R],RPYCSLTK,[S]	1xCarbamidomethyl [C4	Q9P273	Q9P273 [7-14]	Q9P273 1xCarbamidom	0,00	-0,88	0,12
[R],GDTPGHATPGHGGATSSA	1xTMT6plex [N-Term];1	O75533	O75533 [271-289]	O75533 1xTMT6plex [N	0,00	-0,88	0,11
[R],DLQQYQSQAK,[Q]	1xTMT6plex [K10];1xTM	O75396	O75396 [29-38]	O75396 1xTMT6plex [K	0,03	-0,88	0,14
[K],AVQEKSSSPPPR,[K]	1xTMT6plex [K5];1xTM	Q9Y2W1	Q9Y2W1 [528-539]	Q9Y2W1 1xTMT6plex [	0,00	-0,88	0,11
[R],KESSPNSR,[A]	1xTMT6plex [K1];1xTM	Q9H0G5	Q9H0G5 [454-461]	Q9H0G5 1xTMT6plex [I	0,01	-0,88	0,12
[R],GTASPGSQLK,[A]	1xTMT6plex [K10];1xTM	O43918	O43918 [150-159]	O43918 1xTMT6plex [K	0,05	-0,88	0,16
[R],SPVSSQQSLK,[N]	1xTMT6plex [K10];1xTM	Q8IWJ2	Q8IWJ2 [1483-1492]	Q8IWJ2 1xTMT6plex [K	0,03	-0,88	0,14
[R],NSPGSQVASNPR,[Q]	1xTMT6plex [N-Term];1	Q9H0D6	Q9H0D6 [447-458]	Q9H0D6 1xTMT6plex [N	0,03	-0,87	0,14
[R],SVQSAAVPAPTSQLLSSLE	1xTMT6plex [K20];1xTM	Q96T58	Q96T58 [309-328]	Q96T58 1xTMT6plex [K	0,01	-0,87	0,12
[R],ASPVAALK,[A]	1xTMT6plex [K8];1xTM	O15417	O15417 [998-1005]	O15417 1xTMT6plex [K	0,02	-0,87	0,13
[R],SPDKPGGSPSASR,[R]	1xTMT6plex [K4];1xTM	Q9Y3T9	Q9Y3T9 [49-61]	Q9Y3T9 1xTMT6plex [K	0,03	-0,87	0,13
[KR],SVIISIK,[GQ]	1xTMT6plex [K7];1xTM	Q0IIM8; P	Q0IIM8 [358-364]; P09496 [236-242]	Q0IIM8 1xTMT6plex [K	0,02	-0,87	0,13
[R],YFQSPSR,[S]	1xTMT6plex [N-Term];1	Q16629	Q16629 [189-195]	Q16629 1xTMT6plex [N	0,01	-0,87	0,12
[R],EDSGTFSLGK,[M]	1xTMT6plex [K10];1xTM	O75569	O75569 [16-25]	O75569 1xTMT6plex [K	0,01	-0,87	0,12
[R],SRDSSHER,[T]	1xTMT6plex [N-Term];1	Q9BSF8	Q9BSF8 [71-78]	Q9BSF8 1xTMT6plex [N	0,03	-0,87	0,14
[R],SPSPKPTK,[V]	2xTMT6plex [K5; K8];1x	Q15287	Q15287 [155-162]	Q15287 2xTMT6plex [K	0,01	-0,87	0,12
[R],TIHTFPCLK,[E]	1xCarbamidomethyl [C7	Q86X02	Q86X02 [154-162]	Q86X02 1xCarbamidom	0,02	-0,87	0,12
[R],SRSPHEK,[K]	1xTMT6plex [K7];1xTM	Q12872	Q12872 [844-850]	Q12872 1xTMT6plex [K	0,05	-0,87	0,16
[R],DQSPFSK,[I]	1xTMT6plex [K7];1xTM	Q13427	Q13427 [685-691]	Q13427 1xTMT6plex [K	0,01	-0,87	0,12
[K],SRDSESHSR,[V]	1xTMT6plex [N-Term];1	Q15424	Q15424 [614-622]	Q15424 1xTMT6plex [N	0,05	-0,87	0,16
[R],KLESTESR,[S]	1xTMT6plex [K1];1xTM	P02545	P02545 [420-427]	P02545 1xTMT6plex [K	0,04	-0,86	0,15
[R],KRPESGSIR,[K]	1xTMT6plex [K1];1xTM	O75044	O75044 [839-847]	O75044 1xTMT6plex [K	0,01	-0,86	0,12
[R],GYSFTTTAER,[E]	1xTMT6plex [N-Term];1	P60709	P60709 [197-206]	P60709 1xTMT6plex [N	0,02	-0,86	0,13
[R],LESLSYQLSGLQK,[Q]	1xTMT6plex [K13];1xTM	Q03252	Q03252 [314-326]	Q03252 1xTMT6plex [K	0,01	-0,86	0,12
[R],NSSEASSGDFLDLK,[G]	1xTMT6plex [K14];1xTM	Q9UK76	Q9UK76 [86-99]	Q9UK76 1xTMT6plex [K	0,03	-0,86	0,14
[R],RSTPAPK,[E]	1xTMT6plex [K7];1xTM	O95071	O95071 [635-641]	O95071 1xTMT6plex [K	0,04	-0,86	0,15
[R],LSNRPAFMPSEGR,[M]	1xTMT6plex [N-Term];1	P12814	P12814 [347-359]	P12814 1xTMT6plex [N	0,01	-0,86	0,12
[R],INSLTFK,[K]	1xTMT6plex [K7];1xTM	Q96HP0	Q96HP0 [1306-1312]	Q96HP0 1xTMT6plex [K	0,05	-0,86	0,16
[R],GTSTGIISK,[I]	1xTMT6plex [K9];1xTM	Q9P0L2	Q9P0L2 [647-655]	Q9P0L2 1xTMT6plex [K	0,01	-0,86	0,12
[K],KPTGSLPSPSGVR,[K]	1xTMT6plex [K1];1xTM	O00423	O00423 [106-118]	O00423 1xTMT6plex [K	0,02	-0,86	0,12
[K],WAHDKFSGEEGEIEDDES(	1xTMT6plex [K5];1xTM	Q9Y2W1	Q9Y2W1 [922-944]	Q9Y2W1 1xTMT6plex [	0,01	-0,86	0,12
[K],LVSSPGR,[S]	1xTMT6plex [N-Term];1	Q9H6X5	Q9H6X5 [333-339]	Q9H6X5 1xTMT6plex [N	0,03	-0,86	0,14
[K],NSAAATSPK,[G]	1xTMT6plex [K9];1xTM	Q13554	Q13554 [361-369]	Q13554 1xTMT6plex [K	0,02	-0,86	0,12
[R],ELLNVHSANVSK,[V]	1xTMT6plex [K12];1xTM	Q8IZT6	Q8IZT6 [257-268]	Q8IZT6 1xTMT6plex [K	0,03	-0,86	0,13
[R],KSFTSLYK,[D]	2xTMT6plex [K1; K8];1x	Q5SW79	Q5SW79 [957-964]	Q5SW79 2xTMT6plex [	0,01	-0,85	0,12
[R],SPPLMVK,[K]	1xOxidation [M5];1xTM1	Q8N264	Q8N264 [415-421]	Q8N264 1xOxidation [M	0,04	-0,85	0,15
[R],AQKSEPR,[E]	1xTMT6plex [K3];1xTM	Q9Y388	Q9Y388 [213-219]	Q9Y388 1xTMT6plex [K	0,04	-0,85	0,15
[R],RPPSPEPSTKVSEEAESQ(	2xTMT6plex [K10; K25]	Q01082	Q01082 [2099-2123]	Q01082 2xTMT6plex [K	0,00	-0,85	0,12
[M],GLLSILR,[K]	1xTMT6plex [N-Term];1	P36405	P36405 [2-8]	P36405 1xTMT6plex [N	0,04	-0,85	0,15
[R],TSPQVLGSILK,[S]	1xTMT6plex [K11];1xTM	Q7Z417	Q7Z417 [571-581]	Q7Z417 1xTMT6plex [K	0,01	-0,85	0,12

[R],TPVKPSSVEEEDSFFR,[Q]	1xTMT6plex [K4];1xTM	Q9UHB7	Q9UHB7 [674-689]	Q9UHB7 1xTMT6plex [I	0,03	-0,85	0,13
[R],KESSPNSR,[A]	1xTMT6plex [K1];1xTM	Q9H0G5	Q9H0G5 [454-461]	Q9H0G5 1xTMT6plex [I	0,02	-0,85	0,12
[R],CPTPEIQK,[K]	1xCarbamidomethyl [C1	P40818	P40818 [575-582]	P40818 1xCarbamidom	0,03	-0,85	0,13
[R],HKTDPSAAGR,[K]	1xTMT6plex [K2];1xTM	Q96L91	Q96L91 [2292-2301]	Q96L91 1xTMT6plex [K	0,01	-0,85	0,12
[K],SPPPKATEEK,[K]	2xTMT6plex [K5; K10];1	Q9NQG5	Q9NQG5 [134-143]	Q9NQG5 2xTMT6plex [	0,02	-0,85	0,12
[R],SKSHESQLGNR,[I]	1xTMT6plex [K2];1xTM	Q8IVT5	Q8IVT5 [309-319]	Q8IVT5 1xTMT6plex [K	0,02	-0,85	0,12
[K],SKLESHSDSPAWSLSGR,[I	1xTMT6plex [K2];1xTM	Q13029	Q13029 [773-789]	Q13029 1xTMT6plex [K	0,01	-0,85	0,12
[R],VPGSSGHLHK,[T]	1xTMT6plex [K10];1xTM	Q9UEW8	Q9UEW8 [367-376]	Q9UEW8 1xTMT6plex [	0,03	-0,84	0,14
[R],GSFSCPK,[T]	1xCarbamidomethyl [C5	Q5T011	Q5T011 [2958-2964]	Q5T011 1xCarbamidom	0,03	-0,84	0,14
[K],KSPGADVTCSLPR,[H]	1xCarbamidomethyl [C9	Q96BY7	Q96BY7 [1742-1754]	Q96BY7 1xCarbamidom	0,01	-0,84	0,12
[K],EKSLIVEGK,[R]	2xTMT6plex [K2; K9];1x	P35659	P35659 [49-57]	P35659 2xTMT6plex [K	0,00	-0,84	0,12
[K],TSASDVTNIYPGDAGK,[A]	1xTMT6plex [K16];1xTM	Q15042	Q15042 [536-551]	Q15042 1xTMT6plex [K	0,02	-0,84	0,13
[R],SVVSFDK,[V]	1xTMT6plex [K7];1xTM	Q15424	Q15424 [601-607]	Q15424 1xTMT6plex [K	0,02	-0,84	0,13
[R],LSGSARPQELVGTFIQQEV	2xTMT6plex [K21; K32]	O15027	O15027 [533-564]	O15027 2xTMT6plex [K	0,02	-0,84	0,13
[R],LTGTSNNSAGK,[I]	1xTMT6plex [K11];1xTM	Q7Z401	Q7Z401 [967-977]	Q7Z401 1xTMT6plex [K	0,01	-0,84	0,12
[R],SPTGPSNSFLANMGGTVAI	1xTMT6plex [K20];1xTM	Q96I25	Q96I25 [222-241]	Q96I25 1xTMT6plex [K2	0,01	-0,84	0,12
[R],RSTPAPK,[E]	1xTMT6plex [K7];1xTM	O95071	O95071 [635-641]	O95071 1xTMT6plex [K	0,04	-0,84	0,15
[R],ASPAHSPR,[E]	1xTMT6plex [N-Term];1	Q04724	Q04724 [262-269]	Q04724 1xTMT6plex [N	0,04	-0,84	0,15
[R],VLHSGSR,[D]	1xTMT6plex [N-Term];1	Q5T200	Q5T200 [1227-1233]	Q5T200 1xTMT6plex [N	0,01	-0,84	0,12
[R],QKSPAQSDSTTQR,[R]	1xTMT6plex [K2];1xTM	Q9UHB7	Q9UHB7 [547-559]	Q9UHB7 1xTMT6plex [I	0,01	-0,84	0,12
[R],TQLWASEPGTPPLPTSLPS	1xTMT6plex [K25];1xTM	Q9BXP5	Q9BXP5 [535-559]	Q9BXP5 1xTMT6plex [I	0,03	-0,83	0,14
[R],KDSAAALSDR,[E]	1xTMT6plex [K1];1xTM	Q92565	Q92565 [16-25]	Q92565 1xTMT6plex [K	0,02	-0,83	0,13
[R],NSVTPLASPEPTK,[K]	1xTMT6plex [K13];1xTM	Q16875	Q16875 [460-472]	Q16875 1xTMT6plex [K	0,01	-0,83	0,12
[K],TTTAAGSSHSRPGVPVEGS	1xTMT6plex [N-Term];1	Q8NE01	Q8NE01 [682-703]	Q8NE01 1xTMT6plex [N	0,03	-0,83	0,13
[R],KSSISSISGR,[D]	1xTMT6plex [K1];1xTM	Q86SQ0	Q86SQ0 [413-422]	Q86SQ0 1xTMT6plex [I	0,01	-0,83	0,12
[R],ASINSLLSDK,[R]	1xTMT6plex [K10];1xTM	Q04656	Q04656 [1462-1471]	Q04656 1xTMT6plex [K	0,02	-0,83	0,13
[R],GSVHSLDAGLLLPSGDPFS	1xTMT6plex [K20];1xTM	Q15276	Q15276 [373-392]	Q15276 1xTMT6plex [K	0,02	-0,83	0,13
[R],LPSSPVYEDAASFK,[A]	1xTMT6plex [K14];1xTM	Q14247	Q14247 [415-428]	Q14247 1xTMT6plex [K	0,01	-0,83	0,12
[R],LSPLPVVSR,[L]	1xTMT6plex [N-Term];1	O14686	O14686 [653-661]	O14686 1xTMT6plex [N	0,00	-0,83	0,12
[R],NSVTPLASPEPTK,[K]	1xTMT6plex [K13];1xTM	Q16875	Q16875 [460-472]	Q16875 1xTMT6plex [K	0,00	-0,83	0,12
[R],TPPITTK,[E]	1xTMT6plex [K7];1xTM	Q5T200	Q5T200 [1033-1039]	Q5T200 1xTMT6plex [K	0,01	-0,83	0,12
[R],RDSQDGSSYR,[R]	1xTMT6plex [N-Term];1	Q14847	Q14847 [144-153]	Q14847 1xTMT6plex [N	0,02	-0,83	0,13
[R],SPHGQTRPPASLK,[H]	1xTMT6plex [K13];1xTM	Q9H8V3	Q9H8V3 [716-728]	Q9H8V3 1xTMT6plex [I	0,02	-0,83	0,12
[R],SKSPGHHR,[S]	1xTMT6plex [K2];1xTM	Q8NAV1	Q8NAV1 [279-286]	Q8NAV1 1xTMT6plex [I	0,02	-0,83	0,13
[R],KISVVSATK,[G]	2xTMT6plex [K1; K9];1x	Q9UKV3	Q9UKV3 [823-831]	Q9UKV3 2xTMT6plex [I	0,04	-0,83	0,15
[R],FSGGFGAR,[D]	1xTMT6plex [N-Term];1	O00571	O00571 [593-600]	O00571 1xTMT6plex [N	0,05	-0,82	0,16
[K],KASPEHR,[I]	1xTMT6plex [K1];1xTM	Q13415	Q13415 [309-315]	Q13415 1xTMT6plex [K	0,03	-0,82	0,13
[K],LSKSDEQLSSLDR,[D]	1xTMT6plex [K3];1xTM	Q13905	Q13905 [357-369]	Q13905 1xTMT6plex [K	0,00	-0,82	0,12
[R],QSFAVLR,[G]	1xTMT6plex [N-Term];1	Q8TE77	Q8TE77 [36-42]	Q8TE77 1xTMT6plex [N	0,02	-0,82	0,13
[R],FSLSPSLGPQASR,[F]	1xTMT6plex [N-Term];1	Q9NYF3	Q9NYF3 [231-243]	Q9NYF3 1xTMT6plex [N	0,01	-0,82	0,12
[R],SSSLKER,[S]	1xTMT6plex [K5];1xTM	Q9UHB6	Q9UHB6 [581-587]	Q9UHB6 1xTMT6plex [I	0,01	-0,82	0,12
[R],SGSIKGSR,[Y]	1xTMT6plex [K5];1xTM	Q16629	Q16629 [181-188]	Q16629 1xTMT6plex [K	0,03	-0,82	0,13
[K],FTSSPGLSTEDLK,[R]	1xTMT6plex [K13];1xTM	O14936	O14936 [48-60]	O14936 1xTMT6plex [K	0,04	-0,82	0,15
[R],FEIHTPVSDK,[K]	1xTMT6plex [K10];1xTM	Q15361	Q15361 [8-17]	Q15361 1xTMT6plex [K	0,00	-0,82	0,12
[R],KEDSLTASQR,[K]	1xTMT6plex [K1];1xTM	O75920	O75920 [27-36]	O75920 1xTMT6plex [K	0,01	-0,82	0,12
[R],GKLPESPKR,[A]	2xTMT6plex [K2; K8];1x	P17480	P17480 [479-487]	P17480 2xTMT6plex [K	0,00	-0,82	0,12
[R],KLSDHSVR,[E]	1xTMT6plex [K1];1xTM	Q96Q42	Q96Q42 [351-358]	Q96Q42 1xTMT6plex [I	0,01	-0,81	0,12
[R],QLSPQSK,[S]	1xTMT6plex [K7];1xTM	Q86VM9	Q86VM9 [891-897]	Q86VM9 1xTMT6plex [I	0,01	-0,81	0,12
[R],KGSQITQQSTNQSR,[N]	1xTMT6plex [K1];1xTM	P50750	P50750 [345-358]	P50750 1xTMT6plex [K	0,00	-0,81	0,12
[R],KLESGGGGEGGEGTEEED	1xTMT6plex [K1];1xTM	Q96LA8	Q96LA8 [8-29]	Q96LA8 1xTMT6plex [K	0,01	-0,81	0,12
[R],RPEASPVQK,[K]	1xTMT6plex [K9];1xTM	Q3KQU3	Q3KQU3 [406-414]	Q3KQU3 1xTMT6plex [	0,04	-0,81	0,15
[R],RRSPEQSR,[S]	1xTMT6plex [N-Term];1	Q6P1L5	Q6P1L5 [178-185]	Q6P1L5 1xTMT6plex [N	0,00	-0,81	0,11
[R],SKSPSGQK,[R]	2xTMT6plex [K2; K8];1x	Q9NTZ6	Q9NTZ6 [411-418]	Q9NTZ6 2xTMT6plex [I	0,02	-0,81	0,12
[R],KSYESSEDCSEAAGSPAR,	1xCarbamidomethyl [C9	Q6P6C2	Q6P6C2 [370-387]	Q6P6C2 1xCarbamidom	0,03	-0,81	0,13
[R],RASPSPPPK,[R]	1xTMT6plex [K9];1xTM	Q8IYB3	Q8IYB3 [624-632]	Q8IYB3 1xTMT6plex [K	0,01	-0,81	0,12
[R],SRSPATAK,[R]	1xTMT6plex [K8];1xTM	Q9UQ35	Q9UQ35 [484-491]	Q9UQ35 1xTMT6plex [I	0,03	-0,81	0,14
[K],ANVSSPHR,[E]	1xTMT6plex [N-Term];1	Q92530	Q92530 [149-156]	Q92530 1xTMT6plex [N	0,03	-0,81	0,13

[R],LERQISQDVK,[L]	1xTMT6plex [K10];1xTM	Q01433	Q01433 [163-172]	Q01433 1xTMT6plex [K	0,04	-0,81	0,15
[K],RPASLSTAPSEK,[G]	1xTMT6plex [K12];1xTM	Q9UPN4	Q9UPN4 [111-122]	Q9UPN4 1xTMT6plex [	0,00	-0,81	0,12
[R],ASSEDTLNKPGSTAASGVV	1xTMT6plex [K9];1xTM	Q5M775	Q5M775 [53-72]	Q5M775 1xTMT6plex [K	0,02	-0,81	0,12
[R],KPSGLNGEASK,[S]	2xTMT6plex [K1; K11];1	P16070	P16070 [695-705]	P16070 2xTMT6plex [K	0,02	-0,81	0,12
[R],GSEEDSPKK,[E]	2xTMT6plex [K8; K9];1x	O43719	O43719 [493-501]	O43719 2xTMT6plex [K	0,01	-0,81	0,12
[R],KESETQCQTENK,[Y]	1xCarbamidomethyl [C7	Q9NZJ5	Q9NZJ5 [553-564]	Q9NZJ5 1xCarbamidom	0,03	-0,81	0,14
[R],KTTPSPGR,[Q]	1xTMT6plex [K1];1xTM	Q14686	Q14686 [1339-1346]	Q14686 1xTMT6plex [K	0,01	-0,80	0,12
[R],VPGSSGHLHK,[T]	1xTMT6plex [K10];1xTM	Q9UEW8	Q9UEW8 [367-376]	Q9UEW8 1xTMT6plex [	0,02	-0,80	0,13
[R],ISVGGLPVLASMTK,[A]	1xTMT6plex [K14];1xTM	Q8WVQ1	Q8WVQ1 [20-33]	Q8WVQ1 1xTMT6plex	0,02	-0,80	0,13
[R],SSEDLAGPLPSSVSSSSTT	2xTMT6plex [K22; K24]	Q9NRF2	Q9NRF2 [125-148]	Q9NRF2 2xTMT6plex [	0,01	-0,80	0,12
[R],SVNFSLTPNEIK,[V]	1xTMT6plex [K12];1xTM	P46821	P46821 [1276-1287]	P46821 1xTMT6plex [K	0,02	-0,80	0,13
[R],LHQSASSSTSSLSTR,[S]	1xTMT6plex [N-Term];1	O15085	O15085 [648-662]	O15085 1xTMT6plex [N	0,01	-0,80	0,12
[R],ASPPVWHAVR,[M]	1xTMT6plex [N-Term];1	Q86X51	Q86X51 [396-405]	Q86X51 1xTMT6plex [N	0,02	-0,80	0,13
[K],AESGKEEDKK,[S]	3xTMT6plex [K5; K9; K	Q99442	Q99442 [115-124]	Q99442 3xTMT6plex [K	0,05	-0,80	0,16
[R],SHHSLASSLR,[S]	1xTMT6plex [N-Term];1	Q92997	Q92997 [636-645]	Q92997 1xTMT6plex [N	0,00	-0,80	0,11
[R],KSQSENVPASR,[S]	1xTMT6plex [K1];1xTM	O60281	O60281 [1991-2001]	O60281 1xTMT6plex [K	0,00	-0,80	0,11
[K],IHHSSESK,[D]	1xTMT6plex [K8];1xTM	P16383	P16383 [103-110]	P16383 1xTMT6plex [K	0,04	-0,80	0,16
[R],APSPLPK,[M]	1xTMT6plex [K7];1xTM	Q9BSL1	Q9BSL1 [96-102]	Q9BSL1 1xTMT6plex [K	0,01	-0,80	0,12
[R],ASSLNFLNK,[S]	1xTMT6plex [K9];1xTM	Q9H0B6	Q9H0B6 [580-588]	Q9H0B6 1xTMT6plex [K	0,00	-0,79	0,12
[R],SSPVESLK,[K]	1xTMT6plex [K8];1xTM	Q9NRA8	Q9NRA8 [77-84]	Q9NRA8 1xTMT6plex [	0,02	-0,79	0,12
[K],VAHSDKPGSTSTASFR,[D]	1xTMT6plex [K6];1xTM	Q9P0L0	Q9P0L0 [206-221]	Q9P0L0 1xTMT6plex [K	0,03	-0,79	0,13
[R],KSRDSESHSR,[V]	1xTMT6plex [K1];1xTM	Q15424	Q15424 [613-622]	Q15424 1xTMT6plex [K	0,01	-0,79	0,12
[R],VSASSPTTK,[D]	1xTMT6plex [K9];1xTM	Q9BVI0	Q9BVI0 [515-523]	Q9BVI0 1xTMT6plex [K	0,01	-0,79	0,12
[K],AALLKASPK,[K]	2xTMT6plex [K5; K9];1x	P50914	P50914 [133-141]	P50914 2xTMT6plex [K	0,00	-0,79	0,11
[R],RTDALTSSPGR,[D]	1xTMT6plex [N-Term];1	P49736	P49736 [34-44]	P49736 1xTMT6plex [N	0,01	-0,79	0,12
[K],SSISSISGR,[D]	1xTMT6plex [N-Term];1	Q86SQ0	Q86SQ0 [414-422]	Q86SQ0 1xTMT6plex [N	0,03	-0,79	0,14
[R],SPPLIGSESAYESFLSADDK	1xTMT6plex [K20];1xTM	P46821	P46821 [1400-1419]	P46821 1xTMT6plex [K	0,02	-0,79	0,12
[R],TSTSAVPNLFVPLNTNPK,[E	1xTMT6plex [K18];1xTM	P35611	P35611 [480-497]	P35611 1xTMT6plex [K	0,03	-0,79	0,14
[R],TGSESSQTGTSTTSSR,[N]	1xTMT6plex [N-Term];1	P23588	P23588 [420-435]	P23588 1xTMT6plex [N	0,00	-0,79	0,12
[R],TPPLSRRK,[A]	1xMethyl [K8];1xTMT6p	Q9Y3Q8	Q9Y3Q8 [229-236]	Q9Y3Q8 1xMethyl [K23	0,02	-0,79	0,12
[K],QSPSSSPTR,[E]	1xTMT6plex [N-Term];1	P49585	P49585 [318-326]	P49585 1xTMT6plex [N	0,01	-0,79	0,12
[K],ESAQLSGASPK,[E]	1xTMT6plex [K11];1xTM	Q9NYF0	Q9NYF0 [477-487]	Q9NYF0 1xTMT6plex [K	0,04	-0,79	0,15
[K],SSHCDSPPR,[S]	1xCarbamidomethyl [C4	Q12983	Q12983 [126-134]	Q12983 1xCarbamidom	0,02	-0,79	0,13
[K],LGLHSLR,[H]	1xTMT6plex [N-Term];1	P61204	P61204 [143-149]	P61204 1xTMT6plex [N	0,04	-0,79	0,15
[R],IAHSPSLQSLK,[K]	1xTMT6plex [K11];1xTM	Q8N1G1	Q8N1G1 [791-801]	Q8N1G1 1xTMT6plex [	0,05	-0,78	0,16
[R],ASGQAFELILSPR,[S]	1xTMT6plex [N-Term];1	P16949	P16949 [15-27]	P16949 1xTMT6plex [N	0,01	-0,78	0,12
[R],CSTGSPSK,[D]	1xCarbamidomethyl [C1	Q5SW79	Q5SW79 [967-974]	Q5SW79 1xCarbamidor	0,02	-0,78	0,13
[R],SSGFISELPSEEGK,[K]	1xTMT6plex [K14];1xTM	Q5VZK9	Q5VZK9 [967-980]	Q5VZK9 1xTMT6plex [K	0,01	-0,78	0,12
[R],SPQTLAPVGEDAMK,[T]	1xTMT6plex [K14];1xTM	Q92797	Q92797 [1243-1256]	Q92797 1xTMT6plex [K	0,00	-0,78	0,12
[R],SSPPSAGNSPSSLK,[F]	1xTMT6plex [K14];1xTM	P57682	P57682 [70-83]	P57682 1xTMT6plex [K	0,01	-0,78	0,12
[R],SPSVSPSK,[Q]	1xTMT6plex [K8];1xTM	Q96JG6	Q96JG6 [494-501]	Q96JG6 1xTMT6plex [K	0,03	-0,78	0,14
[R],KPSLVASK,[L]	2xTMT6plex [K1; K8];1x	P56211	P56211 [102-109]	P56211 2xTMT6plex [K	0,04	-0,78	0,15
[R],LDSCGKPGELGLPHPLSTG	1xCarbamidomethyl [C4	Q96GZ6	Q96GZ6 [11-41]	Q96GZ6 1xCarbamidom	0,02	-0,78	0,13
[R],HSLSSTK,[L]	1xTMT6plex [K7];1xTM	P42858	P42858 [1861-1867]	P42858 1xTMT6plex [K	0,02	-0,78	0,12
[R],RPQHNSSGASPR,[R]	1xTMT6plex [N-Term];1	Q9BRD0	Q9BRD0 [205-216]	Q9BRD0 1xTMT6plex [	0,01	-0,78	0,12
[R],NSITVPYK,[V]	1xTMT6plex [K8];1xTM	Q00577	Q00577 [255-262]	Q00577 1xTMT6plex [K	0,03	-0,78	0,13
[R],DKSDSDTEGLLFSR,[D]	1xTMT6plex [K2];1xTM	Q9H4G0	Q9H4G0 [646-659]	Q9H4G0 1xTMT6plex [	0,01	-0,78	0,12
[R],SETSSIADPSSPNPFGSSNI	1xTMT6plex [K22];1xTM	Q86UP3	Q86UP3 [2854-2875]	Q86UP3 1xTMT6plex [K	0,03	-0,78	0,14
[K],SFKLSGFSFK,[K]	2xTMT6plex [K3; K10];1	P29966	P29966 [163-172]	P29966 2xTMT6plex [K	0,01	-0,78	0,12
[R],SDSVTGHTSQK,[E]	1xTMT6plex [K11];1xTM	Q9NR09	Q9NR09 [488-498]	Q9NR09 1xTMT6plex [K	0,00	-0,78	0,11
[R],LSWPESEGKPR,[V]	1xTMT6plex [K9];1xTM	Q99490	Q99490 [210-220]	Q99490 1xTMT6plex [K	0,00	-0,77	0,12
[R],TSFSTSDVSK,[L]	1xTMT6plex [K10];1xTM	Q13459	Q13459 [1353-1362]	Q13459 1xTMT6plex [K	0,04	-0,77	0,15
[K],YTLENKEEGSLSDTEADAV	2xTMT6plex [K6; K34];1	Q8N1G4	Q8N1G4 [509-542]	Q8N1G4 2xTMT6plex [	0,01	-0,77	0,12
[R],SLHTDALK,[K]	1xTMT6plex [K8];1xTM	Q9H792	Q9H792 [1292-1299]	Q9H792 1xTMT6plex [K	0,02	-0,77	0,13
[R],TQAPTKASE,[-]	1xTMT6plex [K6];1xTM	P47914	P47914 [151-159]	P47914 1xTMT6plex [K	0,02	-0,77	0,13
[R],ASLADALPLHIAPR,[W]	1xTMT6plex [N-Term];1	Q8ND25	Q8ND25 [122-135]	Q8ND25 1xTMT6plex [N	0,00	-0,77	0,11
[R],KTTPSPGR,[Q]	1xTMT6plex [K1];1xTM	Q14686	Q14686 [1339-1346]	Q14686 1xTMT6plex [K	0,01	-0,77	0,12

[R],SFSISPVR,[L]	1xTMT6plex [N-Term];1	P18583	P18583 [2009-2016]	P18583 1xTMT6plex [N	0,01	-0,77	0,12
[R],KPSDLCTINAK,[E]	1xCarbamidomethyl [C6	Q6P2D8	Q6P2D8 [101-111]	Q6P2D8 1xCarbamidon	0,04	-0,77	0,15
[K],KSPTSLK,[R]	2xTMT6plex [K1; K7];1x	Q9NYZ3	Q9NYZ3 [137-143]	Q9NYZ3 2xTMT6plex [K	0,03	-0,77	0,14
[R],LSLSPLR,[G]	1xTMT6plex [N-Term];1	Q66K74	Q66K74 [654-660]	Q66K74 1xTMT6plex [N	0,05	-0,77	0,16
[R],DKSDSDTEGLLFSR,[D]	1xTMT6plex [K2];1xTM	Q9H4G0	Q9H4G0 [646-659]	Q9H4G0 1xTMT6plex [K	0,01	-0,77	0,12
[R],MKSQAFIEMETR,[E]	1xTMT6plex [K2];1xTM	P43243	P43243 [531-542]	P43243 1xTMT6plex [K	0,02	-0,77	0,12
[R],SNSHSDHIR,[R]	1xTMT6plex [N-Term];1	Q6PH81	Q6PH81 [89-97]	Q6PH81 1xTMT6plex [N	0,00	-0,77	0,11
[R],HTGQVGTK,[L]	1xTMT6plex [K8];1xTM	Q9NZJ5	Q9NZJ5 [981-988]	Q9NZJ5 1xTMT6plex [K	0,00	-0,77	0,12
[R],FALVTPK,[K]	1xTMT6plex [K7];1xTM	P49792	P49792 [1408-1414]	P49792 1xTMT6plex [K	0,00	-0,77	0,12
[R],FSPVLGR,[A]	1xTMT6plex [N-Term];1	Q9NRG9	Q9NRG9 [494-500]	Q9NRG9 1xTMT6plex [	0,01	-0,77	0,12
[R],QPSWDPSPVSSTVPAPSPI	1xTMT6plex [K28];1xTM	Q9NQC3	Q9NQC3 [105-132]	Q9NQC3 1xTMT6plex [	0,02	-0,77	0,12
[R],SYSPDGK,[E]	1xTMT6plex [K7];1xTM	P43243	P43243 [596-602]	P43243 1xTMT6plex [K	0,02	-0,77	0,12
[R],VSLVGADDLRK,[M]	1xTMT6plex [K11];1xTM	Q14160	Q14160 [1377-1387]	Q14160 1xTMT6plex [K	0,03	-0,77	0,14
[R],TSPSKSPFLHSGMK,[M]	2xPhospho [T1; S2];2xT	O14497	O14497 [1599-1612]	O14497 2xPhospho [T1	0,00	-0,76	0,12
[R],TSPSKSPFLHSGMK,[M]	2xTMT6plex [K5; K14];1	O14497	O14497 [1599-1612]	O14497 2xTMT6plex [K	0,00	-0,76	0,12
[R],YSTPHAFTFNTSSPSSEGS	1xTMT6plex [N-Term];1	P04049	P04049 [232-254]	P04049 1xTMT6plex [N	0,03	-0,76	0,15
[R],RPDLYGNLGHPLS,[-]	1xTMT6plex [N-Term];1	Q86X76	Q86X76 [315-327]	Q86X76 1xTMT6plex [N	0,00	-0,76	0,12
[R],LSLQDVPELVDAK,[K]	1xTMT6plex [K13];1xTM	Q8N5W9	Q8N5W9 [5-17]	Q8N5W9 1xTMT6plex [	0,04	-0,76	0,16
[R],TKSDTEPQK,[S]	2xTMT6plex [K2; K9];1x	Q8NDI1	Q8NDI1 [647-655]	Q8NDI1 2xTMT6plex [K	0,02	-0,76	0,12
[K],TVQSNSPISALAPTGK,[E]	1xTMT6plex [K16];1xTM	O95292	O95292 [201-216]	O95292 1xTMT6plex [K	0,02	-0,76	0,13
[R],APAVPPARPGSR,[G]	1xTMT6plex [N-Term];1	Q05193	Q05193 [785-796]	Q05193 1xTMT6plex [N	0,01	-0,76	0,12
[K],VKPETPPR,[Q]	1xTMT6plex [K2];1xTM	Q9UQ35	Q9UQ35 [979-986]	Q9UQ35 1xTMT6plex [I	0,00	-0,76	0,12
[R],DISPEKSELDLGEPGPPGV	1xCarbamidomethyl [C3	Q5VUA4	Q5VUA4 [1711-1741]	Q5VUA4 1xCarbamidor	0,00	-0,76	0,12
[R],SRASPATHR,[R]	1xTMT6plex [N-Term];1	Q9UQ35	Q9UQ35 [1866-1874]	Q9UQ35 1xTMT6plex [I	0,01	-0,76	0,12
[R],RPPSPEPSTK,[V]	1xTMT6plex [K10];1xTM	Q01082	Q01082 [2099-2108]	Q01082 1xTMT6plex [K	0,01	-0,76	0,12
[K],KPLSPVR,[D]	1xTMT6plex [K1];1xTM	Q9UQ84	Q9UQ84 [812-818]	Q9UQ84 1xTMT6plex [I	0,02	-0,76	0,12
[R],GSPDGSLQTGKPSAPK,[K]	2xTMT6plex [K11; K16]	Q8IZL8	Q8IZL8 [480-495]	Q8IZL8 2xTMT6plex [K	0,01	-0,76	0,12
[R],NTETSKSPEK,[D]	2xTMT6plex [K6; K10];1	O14617	O14617 [823-832]	O14617 2xTMT6plex [K	0,01	-0,76	0,12
[K],HRSESESK,[K]	1xTMT6plex [K8];1xTM	Q9UQ35	Q9UQ35 [218-225]	Q9UQ35 1xTMT6plex [I	0,03	-0,76	0,13
[R],KPGSHASSSAR,[R]	1xTMT6plex [K1];1xTM	Q9H7N4	Q9H7N4 [561-571]	Q9H7N4 1xTMT6plex [K	0,02	-0,76	0,12
[R],IGHHSTSDDSSAYR,[S]	1xTMT6plex [N-Term];1	P12694	P12694 [333-346]	P12694 1xTMT6plex [N	0,01	-0,75	0,12
[K],LPTERPCLLEACDESPASR,	2xCarbamidomethyl [C7	P82987	P82987 [614-632]	P82987 2xCarbamidom	0,04	-0,75	0,15
[R],TGAGGGGGSPCTK,[A]	1xCarbamidomethyl [C1	P31277	P31277 [215-227]	P31277 1xCarbamidom	0,01	-0,75	0,12
[R],WDETPKTER,[D]	1xTMT6plex [K6];1xTM	O75533	O75533 [293-301]	O75533 1xTMT6plex [K	0,02	-0,75	0,13
[R],KNSASAERDR,[K]	1xTMT6plex [K1];1xTM	Q96T37	Q96T37 [720-729]	Q96T37 1xTMT6plex [K	0,04	-0,75	0,15
[K],LNESTFDTQITK,[K]	1xTMT6plex [K12];1xTM	P78527	P78527 [1858-1869]	P78527 1xTMT6plex [K	0,01	-0,75	0,12
[K],KSPLSSILFSALDSDTR,[I]	1xTMT6plex [K1];1xTM	Q7Z3B3	Q7Z3B3 [267-283]	Q7Z3B3 1xTMT6plex [K	0,04	-0,75	0,15
[R],YEPSDKDRQSPPPAK,[R]	2xTMT6plex [K6; K15];1	Q86VM9	Q86VM9 [833-847]	Q86VM9 2xTMT6plex [I	0,02	-0,75	0,12
[R],DGLAPEKTSPDRDK,[K]	2xTMT6plex [K7; K14];1	Q7L4I2	Q7L4I2 [9-22]	Q7L4I2 2xTMT6plex [K1	0,04	-0,75	0,16
[R],SSSPYSK,[S]	1xTMT6plex [K7];1xTM	Q9NYF8	Q9NYF8 [146-152]	Q9NYF8 1xTMT6plex [K	0,04	-0,75	0,15
[R],ATTPLYHGFK,[E]	1xTMT6plex [K10];1xTM	Q5T4S7	Q5T4S7 [903-912]	Q5T4S7 1xTMT6plex [K	0,01	-0,75	0,12
[K],TNHSSPEAQSK,[Q]	1xTMT6plex [K11];1xTM	O95819	O95819 [576-586]	O95819 1xTMT6plex [K	0,01	-0,75	0,12
[K],RASPSKPASAPASR,[S]	1xTMT6plex [K6];1xTM	P27816	P27816 [785-798]	P27816 1xTMT6plex [K	0,02	-0,75	0,13
[R],KESQEHTK,[D]	2xTMT6plex [K1; K8];1x	Q9H814	Q9H814 [162-169]	Q9H814 2xTMT6plex [K	0,03	-0,75	0,14
[R],VSPENLVDK,[S]	1xTMT6plex [K9];1xTM	O75376	O75376 [2119-2127]	O75376 1xTMT6plex [K	0,02	-0,75	0,13
[R],SKSETGDSSIFR,[K]	1xTMT6plex [K2];1xTM	O43166	O43166 [286-297]	O43166 1xTMT6plex [K	0,05	-0,75	0,16
[R],KSQVTLDVEK,[L]	2xTMT6plex [K1; K10];1	Q96CN9	Q96CN9 [449-458]	Q96CN9 2xTMT6plex [K	0,02	-0,75	0,13
[R],SPTGPSNSFLANMGGTVAH	1xTMT6plex [K20];1xTM	Q96I25	Q96I25 [222-241]	Q96I25 1xTMT6plex [K2	0,01	-0,75	0,12
[K],TKDSGSISLQETR,[R]	1xTMT6plex [K2];1xTM	Q9H2G2	Q9H2G2 [774-786]	Q9H2G2 1xTMT6plex [I	0,05	-0,75	0,16
[R],CATPVIIDEILPSK,[K]	1xCarbamidomethyl [C1	Q9ULW0	Q9ULW0 [145-158]	Q9ULW0 1xCarbamidor	0,01	-0,75	0,12
[R],SHIASPSPCPDR,[M]	1xCarbamidomethyl [C9	O75665	O75665 [758-769]	O75665 1xCarbamidom	0,02	-0,75	0,13
[R],KMSGGSTMSSGGGNTNNS	2xOxidation [M2; M8];2	Q86U70	Q86U70 [300-320]	Q86U70 2xOxidation [M	0,04	-0,75	0,15
[R],KSSVTEE,[-]	1xTMT6plex [K1];1xTM	O94874	O94874 [788-794]	O94874 1xTMT6plex [K	0,02	-0,74	0,13
[R],SNSHSDHIR,[R]	1xTMT6plex [N-Term];1	Q6PH81	Q6PH81 [89-97]	Q6PH81 1xTMT6plex [N	0,00	-0,74	0,11
[R],HSNLMLEDLDK,[S]	1xTMT6plex [K11];1xTM	P11171	P11171 [663-673]	P11171 1xTMT6plex [K	0,01	-0,74	0,12
[R],RDSSVIK,[E]	1xTMT6plex [K7];1xTM	Q6NT76	Q6NT76 [168-174]	Q6NT76 1xTMT6plex [K	0,01	-0,74	0,12
[R],LPSPTSPFSSLSQDQAATS	1xTMT6plex [K20];1xTM	Q8WWI1	Q8WWI1 [986-1005]	Q8WWI1 1xTMT6plex [	0,01	-0,74	0,12

[K],KGDVEGSQSQDEGEGSGE	1xTMT6plex [K1];1xTM	Q9UQE7	Q9UQE7 [1059-1079]	Q9UQE7 1xTMT6plex [	0,04	-0,74	0,15
[R],LDPFADGGKTPDPK,[M]	1xMethyl [K9];1xTMT6p	O75533	O75533 [133-146]	O75533 1xMethyl [K141	0,01	-0,74	0,12
[R],QSHSGSISPYPKVK,[A]	2xTMT6plex [K12; K14]	Q9UQ35	Q9UQ35 [987-1000]	Q9UQ35 2xTMT6plex [	0,00	-0,74	0,11
[R],GLSPSPSPASK,[S]	1xTMT6plex [K11];1xTM	A1L443	A1L443 [685-695]	A1L443 1xTMT6plex [K	0,03	-0,74	0,14
[R],STTPAPK,[S]	1xTMT6plex [K7];1xTM	Q9UQ35	Q9UQ35 [250-256]	Q9UQ35 1xTMT6plex [	0,01	-0,74	0,12
[K],KSPSEAR,[Q]	1xTMT6plex [K1];1xTM	P46821	P46821 [2071-2077]	P46821 1xTMT6plex [K	0,01	-0,74	0,12
[R],LQDVSGQLSSSK,[K]	1xTMT6plex [K12];1xTM	Q15059	Q15059 [672-683]	Q15059 1xTMT6plex [K	0,01	-0,73	0,12
[R],KDDSDDDGGGWITPSNIK,[	2xTMT6plex [K1; K18];1	Q9ULX3	Q9ULX3 [198-215]	Q9ULX3 2xTMT6plex [K	0,02	-0,73	0,12
[R],TSPGGFK,[F]	1xTMT6plex [K7];1xTM	Q5MIZ7	Q5MIZ7 [767-773]	Q5MIZ7 1xTMT6plex [K	0,02	-0,73	0,13
[K],KEPVAGSPFER,[R]	1xTMT6plex [K1];1xTM	Q96JB3	Q96JB3 [342-352]	Q96JB3 1xTMT6plex [K	0,01	-0,73	0,12
[R],SKSQNILSTEEER,[T]	1xTMT6plex [K2];1xTM	Q6NT16	Q6NT16 [436-448]	Q6NT16 1xTMT6plex [K	0,02	-0,73	0,13
[K],QFTGKSTK,[T]	1xMethyl [K5];1xTMT6p	Q15397	Q15397 [8-15]	Q15397 1xMethyl [K12]	0,04	-0,73	0,15
[K],QASDTGSNDAHNK,[K]	1xTMT6plex [K13];1xTM	P20810	P20810 [69-81]	P20810 1xTMT6plex [K	0,05	-0,73	0,16
[R],SRSISLR,[R]	1xTMT6plex [N-Term];1	Q16629	Q16629 [163-169]	Q16629 1xTMT6plex [N	0,00	-0,73	0,11
[R],ASHSSERPPPR,[Q]	1xTMT6plex [N-Term];1	O15027	O15027 [1225-1235]	O15027 1xTMT6plex [N	0,03	-0,73	0,13
[R],SSSSSASPSSPSSR,[E]	1xTMT6plex [N-Term];1	Q9NYF8	Q9NYF8 [751-764]	Q9NYF8 1xTMT6plex [N	0,04	-0,73	0,15
[R],RDSWSYINSK,[S]	1xTMT6plex [K10];1xTM	P13612	P13612 [1019-1028]	P13612 1xTMT6plex [K	0,01	-0,73	0,12
[R],KASATEAR,[Y]	1xTMT6plex [K1];1xTM	O75146	O75146 [439-446]	O75146 1xTMT6plex [K	0,01	-0,73	0,12
[R],KSPSGPVK,[S]	2xTMT6plex [K1; K8];1x	Q9BVC5	Q9BVC5 [181-188]	Q9BVC5 2xTMT6plex [	0,01	-0,72	0,12
[R],KSSTPEEVK,[K]	2xTMT6plex [K1; K9];1x	P23528	P23528 [22-30]	P23528 2xTMT6plex [K	0,00	-0,72	0,11
[R],GTPGPDSSGSLGSGEFTG	1xTMT6plex [K20];1xTM	Q9UBC2	Q9UBC2 [365-384]	Q9UBC2 1xTMT6plex [	0,02	-0,72	0,13
[R],QGLKSPQESLSDLGAIESL	1xTMT6plex [K4];1xTM	Q96JG6	Q96JG6 [11-30]	Q96JG6 1xTMT6plex [K	0,02	-0,72	0,12
[R],SPILLPK,[G]	1xTMT6plex [K7];1xTM	Q8TD16	Q8TD16 [582-588]	Q8TD16 1xTMT6plex [K	0,01	-0,72	0,12
[K],LAIRESIESEIR,[R]	1xTMT6plex [N-Term];1	P55072	P55072 [697-708]	P55072 1xTMT6plex [N	0,02	-0,72	0,13
[K],LLRQSPPLPGR,[E]	1xTMT6plex [N-Term];1	Q9C0B5	Q9C0B5 [550-560]	Q9C0B5 1xTMT6plex [N	0,01	-0,72	0,12
[R],TLDPLWK,[V]	1xTMT6plex [K7];1xTM	Q9Y5S2	Q9Y5S2 [858-864]	Q9Y5S2 1xTMT6plex [K	0,00	-0,72	0,12
[R],LSLPADIR,[IL]	1xTMT6plex [N-Term];1	Q00537; (	Q00537 [145-152]; Q00536 [118-125	Q00537 1xTMT6plex [N	0,04	-0,72	0,15
[K],TVTPASSAK,[T]	1xTMT6plex [K9];1xTM	Q16555	Q16555 [512-520]	Q16555 1xTMT6plex [K	0,04	-0,72	0,15
[K],LFGGPGSR,[R]	1xTMT6plex [N-Term];1	Q96PE2	Q96PE2 [132-139]	Q96PE2 1xTMT6plex [N	0,02	-0,72	0,13
[R],TQSIQGK,[G]	1xTMT6plex [K7];1xTM	Q53HL2	Q53HL2 [152-158]	Q53HL2 1xTMT6plex [K	0,02	-0,72	0,13
[R],KHSGTDR,[T]	1xTMT6plex [K1];1xTM	Q53HC0	Q53HC0 [323-329]	Q53HC0 1xTMT6plex [K	0,05	-0,72	0,16
[R],SQAGHTLHHQESR,[R]	1xTMT6plex [N-Term];1	Q99959	Q99959 [172-184]	Q99959 1xTMT6plex [N	0,02	-0,72	0,13
[K],KTEVQAHSPSR,[K]	1xTMT6plex [K1];1xTM	P11137	P11137 [1473-1483]	P11137 1xTMT6plex [K	0,01	-0,72	0,12
[R],ASPSPPPK,[R]	1xTMT6plex [K8];1xTM	Q8IYB3	Q8IYB3 [625-632]	Q8IYB3 1xTMT6plex [K	0,02	-0,72	0,13
[R],ATSPESTSR,[S]	1xTMT6plex [N-Term];1	Q8N3X1	Q8N3X1 [462-470]	Q8N3X1 1xTMT6plex [N	0,02	-0,72	0,12
[K],VNVTVDYIRPASPATETVPA	1xTMT6plex [N-Term];1	Q7KZF4	Q7KZF4 [415-438]	Q7KZF4 1xTMT6plex [N	0,05	-0,72	0,16
[K],TKPSSSSR,[Q]	1xTMT6plex [K2];1xTM	Q7Z2K8	Q7Z2K8 [718-725]	Q7Z2K8 1xTMT6plex [K	0,04	-0,72	0,15
[R],SSLEGPTILDIEK,[F]	1xTMT6plex [K13];1xTM	Q7L576	Q7L576 [582-594]	Q7L576 1xTMT6plex [K	0,01	-0,71	0,12
[R],TSSPHKEESPK,[K]	2xTMT6plex [K6; K11];1	P51003	P51003 [652-662]	P51003 2xTMT6plex [K	0,01	-0,71	0,12
[R],LSETSIK,[D]	1xTMT6plex [K7];1xTM	Q9UHB6	Q9UHB6 [262-268]	Q9UHB6 1xTMT6plex [	0,04	-0,71	0,15
[R],ALSVLGCGHTSSTK,[C]	1xCarbamidomethyl [C7	Q5T4S7	Q5T4S7 [3858-3871]	Q5T4S7 1xCarbamidom	0,03	-0,71	0,14
[K],SSQSSTHESESESK,[E]	1xTMT6plex [K14];1xTM	Q8N9E0	Q8N9E0 [141-154]	Q8N9E0 1xTMT6plex [K	0,04	-0,71	0,15
[R],AMSTTSISSPQPGKLR,[S]	1xTMT6plex [K14];1xTM	Q9UJU6	Q9UJU6 [267-282]	Q9UJU6 1xTMT6plex [K	0,02	-0,71	0,13
[K],GKSDSEDGEK,[E]	2xTMT6plex [K2; K10];1	O00203	O00203 [748-757]	O00203 2xTMT6plex [K	0,04	-0,71	0,15
[R],THSIESSGK,[L]	1xTMT6plex [K9];1xTM	P45985	P45985 [78-86]	P45985 1xTMT6plex [K	0,00	-0,71	0,11
[R],VSASSPTTK,[D]	1xTMT6plex [K9];1xTM	Q9BVI0	Q9BVI0 [515-523]	Q9BVI0 1xTMT6plex [K	0,02	-0,71	0,12
[R],IGSPLSPK,[K]	1xTMT6plex [K8];1xTM	Q659C4	Q659C4 [338-345]	Q659C4 1xTMT6plex [K	0,01	-0,71	0,12
[R],LSTGTTVEDVQK,[I]	1xTMT6plex [K12];1xTM	Q86SQ0	Q86SQ0 [467-478]	Q86SQ0 1xTMT6plex [K	0,04	-0,71	0,15
[R],SKSHESQLGNR,[I]	1xTMT6plex [K2];1xTM	Q8IVT5	Q8IVT5 [309-319]	Q8IVT5 1xTMT6plex [K	0,01	-0,71	0,12
[R],SPSPKPR,[G]	1xTMT6plex [K5];1xTM	Q9UQ35	Q9UQ35 [1762-1768]	Q9UQ35 1xTMT6plex [	0,01	-0,71	0,12
[K],NPPSPRPSQLPVLDR,[D]	1xTMT6plex [N-Term];1	Q6ZRS2	Q6ZRS2 [3013-3027]	Q6ZRS2 1xTMT6plex [N	0,02	-0,71	0,12
[R],SPPGPAGSSPK,[Q]	1xTMT6plex [K11];1xTM	Q9H9D4	Q9H9D4 [338-348]	Q9H9D4 1xTMT6plex [K	0,00	-0,71	0,12
[R],EGSPIPHDPEFGSKLASVPI	1xTMT6plex [K14];1xTM	P85037	P85037 [443-464]	P85037 1xTMT6plex [K	0,00	-0,71	0,11
[K],GSISEDELITAIK,[E]	1xTMT6plex [K13];1xTM	Q16799	Q16799 [349-361]	Q16799 1xTMT6plex [K	0,03	-0,71	0,14
[R],APGYPSSPVTTASGTTLR,[	1xTMT6plex [N-Term];1	Q9HCK8	Q9HCK8 [2513-2530]	Q9HCK8 1xTMT6plex [	0,01	-0,71	0,12
[R],KASQLVGIEK,[K]	2xTMT6plex [K1; K10];1	Q9NPD8	Q9NPD8 [182-191]	Q9NPD8 2xTMT6plex [	0,02	-0,71	0,13
[R],GSLASLDSLR,[K]	1xTMT6plex [N-Term];1	O60716	O60716 [345-354]	O60716 1xTMT6plex [N	0,04	-0,71	0,15

[K],EKSQEVSSK,[S]	2xTMT6plex [K2; K9];1x	Q9NYV4	Q9NYV4 [131-139]	Q9NYV4 2xTMT6plex [	0,02	-0,70	0,12
[R],SASTISK,[S]	1xTMT6plex [K7];1xTM	P16389	P16389 [449-455]	P16389 1xTMT6plex [K	0,02	-0,70	0,13
[K],SSSPVKK,[S]	2xTMT6plex [K6; K7];1x	P46821	P46821 [2254-2260]	P46821 2xTMT6plex [K	0,00	-0,70	0,12
[K],VVKQASEGPLK,[G]	2xTMT6plex [K3; K11];1	P04406	P04406 [261-271]	P04406 2xTMT6plex [K	0,01	-0,70	0,12
[R],SVHSEHSAR,[S]	1xTMT6plex [N-Term];1	O15027	O15027 [1347-1355]	O15027 1xTMT6plex [N	0,02	-0,70	0,13
[R],DRKSSPSK,[D]	2xTMT6plex [K3; K8];1x	Q9BUQ8	Q9BUQ8 [35-42]	Q9BUQ8 2xTMT6plex [	0,02	-0,70	0,12
[R],NFTKPQDGDVIAPLITPQK,[	2xTMT6plex [K4; K19];1	Q9Y3Z3	Q9Y3Z3 [577-595]	Q9Y3Z3 2xTMT6plex [K	0,02	-0,70	0,13
[R],SPIALPVK,[Q]	1xTMT6plex [K8];1xTM	Q8TF01	Q8TF01 [211-218]	Q8TF01 1xTMT6plex [K	0,01	-0,70	0,12
[K],AEGEPQEESPLKSK,[S]	2xTMT6plex [K12; K14]	Q9NYF8	Q9NYF8 [169-182]	Q9NYF8 2xTMT6plex [	0,00	-0,70	0,12
[R],VNSASSSNPPAEVDPDTILK	1xTMT6plex [K20];1xTM	Q9BY77	Q9BY77 [381-400]	Q9BY77 1xTMT6plex [K	0,01	-0,70	0,12
[R],RVSHSPPPK,[Q]	1xTMT6plex [K9];1xTM	Q8IYB3	Q8IYB3 [634-642]	Q8IYB3 1xTMT6plex [K	0,01	-0,70	0,12
[R],RSSPESDR,[Q]	1xTMT6plex [N-Term];1	Q5T200	Q5T200 [1277-1284]	Q5T200 1xTMT6plex [N	0,00	-0,70	0,11
[R],SSDKPIR,[T]	1xTMT6plex [K4];1xTM	Q99961	Q99961 [291-297]	Q99961 1xTMT6plex [K	0,03	-0,70	0,14
[R],GGSISVQVNSIK,[F]	1xTMT6plex [K12];1xTM	Q14978	Q14978 [684-695]	Q14978 1xTMT6plex [K	0,03	-0,70	0,14
[R],KGSQITQQSTNQSR,[N]	1xTMT6plex [K1];1xTM	P50750	P50750 [345-358]	P50750 1xTMT6plex [K	0,03	-0,70	0,13
[R],SPPQSPGPGK,[A]	1xTMT6plex [K10];1xTM	Q86VQ6	Q86VQ6 [4-13]	Q86VQ6 1xTMT6plex [	0,01	-0,69	0,12
[R],KADSDSEDKGEESKPK,[K]	4xTMT6plex [K1; K9; K	P83916	P83916 [86-101]	P83916 4xTMT6plex [K	0,01	-0,69	0,12
[R],LSPVPVPR,[A]	1xTMT6plex [N-Term];1	Q9UKM9	Q9UKM9 [134-141]	Q9UKM9 1xTMT6plex [	0,03	-0,69	0,14
[R],YSPSQNSPIHHIPSR,[R]	1xTMT6plex [N-Term];1	Q9NYF8	Q9NYF8 [284-298]	Q9NYF8 1xTMT6plex [N	0,00	-0,69	0,11
[R],LSSTSLASGHSVR,[L]	1xTMT6plex [N-Term];1	Q9BZL6	Q9BZL6 [196-208]	Q9BZL6 1xTMT6plex [N	0,02	-0,69	0,12
[R],LEDLDTCMMTPK,[S]	1xCarbamidomethyl [C7	Q5VZK9	Q5VZK9 [907-918]	Q5VZK9 1xCarbamidom	0,01	-0,69	0,12
[R],LRDPDSHIVK,[K]	1xTMT6plex [K10];1xTM	Q96EK5	Q96EK5 [495-504]	Q96EK5 1xTMT6plex [K	0,01	-0,69	0,12
[R],LPLVPESPR,[R]	1xTMT6plex [N-Term];1	Q86WB0	Q86WB0 [315-323]	Q86WB0 1xTMT6plex [	0,00	-0,69	0,12
[R],SKSDSYTLDPDTLR,[K]	1xTMT6plex [K2];1xTM	O75592	O75592 [2869-2882]	O75592 1xTMT6plex [K	0,00	-0,69	0,12
[R],SRTPSLPTPPTR,[E]	1xTMT6plex [N-Term];1	P10636	P10636 [527-538]	P10636 1xTMT6plex [N	0,04	-0,69	0,15
[R],HESGASIK,[I]	1xTMT6plex [K8];1xTM	P61978	P61978 [415-422]	P61978 1xTMT6plex [K	0,02	-0,69	0,13
[K],IHHSSESK,[D]	1xTMT6plex [K8];1xTM	P16383	P16383 [103-110]	P16383 1xTMT6plex [K	0,04	-0,69	0,15
[R],YNRSDIMPSGR,[S]	1xTMT6plex [N-Term];1	Q8TB72	Q8TB72 [697-707]	Q8TB72 1xTMT6plex [N	0,03	-0,69	0,14
[R],SKDASPINR,[W]	1xTMT6plex [K2];1xTM	Q13523	Q13523 [427-435]	Q13523 1xTMT6plex [K	0,04	-0,69	0,15
[K],TKDSGSISLQETR,[R]	1xTMT6plex [K2];1xTM	Q9H2G2	Q9H2G2 [774-786]	Q9H2G2 1xTMT6plex [	0,01	-0,69	0,12
[R],SGSSPGLR,[D]	1xTMT6plex [N-Term];1	Q9UQ35	Q9UQ35 [1441-1448]	Q9UQ35 1xTMT6plex [	0,00	-0,68	0,12
[R],SGSSPGLR,[D]	1xTMT6plex [N-Term];1	Q9UQ35	Q9UQ35 [1441-1448]	Q9UQ35 1xTMT6plex [	0,00	-0,68	0,12
[R],SLEDLHQK,[K]	1xTMT6plex [K8];1xTM	B2RTY4	B2RTY4 [1258-1265]	B2RTY4 1xTMT6plex [K	0,01	-0,68	0,12
[K],KSASSGEMSK,[K]	2xTMT6plex [K1; K10];1	Q8TDR0	Q8TDR0 [305-314]	Q8TDR0 2xTMT6plex [	0,04	-0,68	0,16
[K],RSSGLQSGHSSQR,[T]	1xTMT6plex [N-Term];1	Q9UHB7	Q9UHB7 [123-135]	Q9UHB7 1xTMT6plex [	0,01	-0,68	0,12
[K],SLIPDIKPLAGQEAVVDLHA	1xTMT6plex [K7];1xTM	Q9UKV3	Q9UKV3 [873-904]	Q9UKV3 1xTMT6plex [	0,00	-0,68	0,12
[K],SLIPDIKPLAGQEAVVDLHA	1xTMT6plex [K7];1xTM	Q9UKV3	Q9UKV3 [873-904]	Q9UKV3 1xTMT6plex [	0,00	-0,68	0,12
[R],TSPQVLGSILK,[S]	1xTMT6plex [K11];1xTM	Q7Z417	Q7Z417 [571-581]	Q7Z417 1xTMT6plex [K	0,03	-0,68	0,14
[K],YSHSNSR,[I]	1xTMT6plex [N-Term];1	O95163	O95163 [1182-1188]	O95163 1xTMT6plex [N	0,03	-0,68	0,14
[R],RLSVCGR,[T]	1xCarbamidomethyl [C5	Q9NPG4	Q9NPG4 [1138-1144]	Q9NPG4 1xCarbamidom	0,03	-0,68	0,14
[R],IHFSPSEPK,[T]	1xTMT6plex [K9];1xTM	Q8IZT6	Q8IZT6 [602-610]	Q8IZT6 1xTMT6plex [K	0,02	-0,68	0,12
[R],SLGEISALTSK,[L]	1xTMT6plex [K11];1xTM	P18206	P18206 [434-444]	P18206 1xTMT6plex [K	0,01	-0,68	0,12
[K],NLSLEAIQLCDR,[D]	1xCarbamidomethyl [C1	Q96FC7	Q96FC7 [23-34]	Q96FC7 1xCarbamidom	0,01	-0,68	0,12
[R],TPHTPGTPK,[S]	1xTMT6plex [K9];1xTM	P11137	P11137 [1625-1633]	P11137 1xTMT6plex [K	0,03	-0,68	0,14
[R],SSGFISELPSEEGK,[K]	1xTMT6plex [K14];1xTM	Q5VZK9	Q5VZK9 [967-980]	Q5VZK9 1xTMT6plex [K	0,01	-0,68	0,12
[R],SPSQYHDFAEK,[L]	1xTMT6plex [K11];1xTM	Q9UGU0	Q9UGU0 [1005-1015]	Q9UGU0 1xTMT6plex [	0,00	-0,68	0,12
[R],TSLADSSIFDSK,[V]	1xTMT6plex [K12];1xTM	Q9H8V3	Q9H8V3 [14-25]	Q9H8V3 1xTMT6plex [K	0,02	-0,68	0,12
[K],KSESEVEEAAAIIAQRPDNP	1xTMT6plex [K1];1xTM	Q16643	Q16643 [271-291]	Q16643 1xTMT6plex [K	0,04	-0,68	0,15
[K],HGLAHDEMKSPR,[E]	1xTMT6plex [K9];1xTM	Q9Y2W1	Q9Y2W1 [689-700]	Q9Y2W1 1xTMT6plex [	0,02	-0,68	0,12
[K],IQTPPPKKEPPK,[E]	3xTMT6plex [K7; K8; K	P48634	P48634 [876-887]	P48634 3xTMT6plex [K	0,01	-0,67	0,12
[K],DASSGKENRSPSR,[R]	1xTMT6plex [K6];1xTM	Q13523	Q13523 [332-344]	Q13523 1xTMT6plex [K	0,00	-0,67	0,12
[R],YFQSPSR,[S]	1xTMT6plex [N-Term];1	Q16629	Q16629 [189-195]	Q16629 1xTMT6plex [N	0,01	-0,67	0,12
[R],DPHLLDPTVEYVK,[F]	1xTMT6plex [K13];1xTM	O75815	O75815 [107-119]	O75815 1xTMT6plex [K	0,02	-0,67	0,12
[R],SFGHFPGPEFLDVEK,[T]	1xTMT6plex [K15];1xTM	Q8IWW6	Q8IWW6 [176-190]	Q8IWW6 1xTMT6plex [	0,02	-0,67	0,13
[R],EDTSLPSLSK,[D]	1xTMT6plex [K10];1xTM	Q9NRY4	Q9NRY4 [998-1007]	Q9NRY4 1xTMT6plex [	0,05	-0,67	0,16
[R],AAQQQQPSASPR,[A]	1xTMT6plex [N-Term];1	Q86U86	Q86U86 [1444-1455]	Q86U86 1xTMT6plex [N	0,01	-0,67	0,12
[R],AAQQQQPSASPR,[A]	1xTMT6plex [N-Term];1	Q86U86	Q86U86 [1444-1455]	Q86U86 1xTMT6plex [N	0,01	-0,67	0,12

[R],SPPLPAVIR,[N]	1xTMT6plex [N-Term];1	Q9Y6X9	Q9Y6X9 [615-623]	Q9Y6X9 1xTMT6plex [N	0,01	-0,67	0,12
[R],SKSPASVDR,[Q]	1xTMT6plex [K2];1xTM	Q13243	Q13243 [248-256]	Q13243 1xTMT6plex [K	0,01	-0,67	0,12
[K],LGSASQAGPPGSSR,[K]	1xTMT6plex [N-Term];1	Q9P0K8	Q9P0K8 [27-40]	Q9P0K8 1xTMT6plex [N	0,01	-0,67	0,12
[K],KQATSPASK,[K]	2xTMT6plex [K1; K9];1x	P17480	P17480 [385-393]	P17480 2xTMT6plex [K	0,00	-0,67	0,12
[R],LSWPNHYSGASESQTR,[S]	1xTMT6plex [N-Term];1	Q9H706	Q9H706 [624-639]	Q9H706 1xTMT6plex [N	0,03	-0,67	0,14
[R],TSPVIMAR,[V]	1xTMT6plex [N-Term];1	O15085	O15085 [254-261]	O15085 1xTMT6plex [N	0,01	-0,67	0,12
[K],KVAGAATPK,[K]	1xMethyl [K1];1xTMT6p	P16402	P16402 [141-149]	P16402 1xMethyl [K141	0,03	-0,67	0,14
[R],LNSSDPSLIGLK,[Q]	1xTMT6plex [K12];1xTM	Q9UHI6	Q9UHI6 [266-277]	Q9UHI6 1xTMT6plex [K	0,00	-0,67	0,12
[R],SPAKTIAPQNAPR,[D]	1xTMT6plex [K4];1xTM	Q9NYF8	Q9NYF8 [300-312]	Q9NYF8 1xTMT6plex [K	0,00	-0,67	0,12
[R],SQENILQGFSTSHK,[E]	1xTMT6plex [K14];1xTM	Q86V48	Q86V48 [611-624]	Q86V48 1xTMT6plex [K	0,01	-0,67	0,12
[R],STSYGYSR,[S]	1xTMT6plex [N-Term];1	Q13242	Q13242 [187-194]	Q13242 1xTMT6plex [N	0,03	-0,67	0,15
[R],SPACSSLTPSLCK,[L]	2xCarbamidomethyl [C4	Q96R06	Q96R06 [43-55]	Q96R06 2xCarbamidom	0,00	-0,67	0,11
[R],YSQGDDDGSSSSGGSSVA	1xTMT6plex [K26];1xTM	Q9UH99	Q9UH99 [11-36]	Q9UH99 1xTMT6plex [K	0,03	-0,67	0,14
[R],GSLSLSFER,[E]	1xTMT6plex [N-Term];1	P57078	P57078 [433-441]	P57078 1xTMT6plex [N	0,00	-0,67	0,12
[K],KESKEEETSIDVAGKPNEVT	4xTMT6plex [K1; K4; K	P53985	P53985 [459-479]	P53985 4xTMT6plex [K	0,02	-0,67	0,12
[R],SSNVSYK,[Y]	1xTMT6plex [K7];1xTM	P11717	P11717 [2346-2352]	P11717 1xTMT6plex [K	0,05	-0,67	0,16
[R],MLQAISPK,[Q]	1xTMT6plex [K8];1xTM	P49585; Q	P49585 [310-317]; Q9Y5K3 [310-317	P49585 1xTMT6plex [K	0,05	-0,66	0,16
[K],TPPAPSPFDLPELK,[H]	1xTMT6plex [K14];1xTM	O95239	O95239 [1181-1194]	O95239 1xTMT6plex [K	0,02	-0,66	0,12
[K],ASVHSSGR,[G]	1xTMT6plex [N-Term];1	Q9Y5B9	Q9Y5B9 [1022-1029]	Q9Y5B9 1xTMT6plex [N	0,02	-0,66	0,13
[R],NLNLVSSTASIK,[D]	1xTMT6plex [K12];1xTM	Q9NRY4	Q9NRY4 [764-775]	Q9NRY4 1xTMT6plex [K	0,04	-0,66	0,15
[R],LALLNEKQAASPLEPK,[E]	2xTMT6plex [K7; K16];1	Q14980	Q14980 [261-276]	Q14980 2xTMT6plex [K	0,00	-0,66	0,11
[R],GSPHLLR,[K]	1xTMT6plex [N-Term];1	O75069	O75069 [193-199]	O75069 1xTMT6plex [N	0,04	-0,66	0,16
[R],NSIQFTDGYEVK,[E]	1xTMT6plex [K12];1xTM	P51812	P51812 [414-425]	P51812 1xTMT6plex [K	0,04	-0,66	0,15
[R],ALSSSKQSSSSR,[D]	1xTMT6plex [K6];1xTM	P53999	P53999 [48-59]	P53999 1xTMT6plex [K	0,03	-0,66	0,14
[R],SPFLQKQLTQPETHFGR,[E	1xTMT6plex [K6];1xTM	Q9UJU6	Q9UJU6 [283-299]	Q9UJU6 1xTMT6plex [K	0,03	-0,66	0,14
[R],LSTAITLLPLEEGR,[T]	1xTMT6plex [N-Term];1	Q86UU1	Q86UU1 [50-63]	Q86UU1 1xTMT6plex [N	0,05	-0,66	0,16
[R],TPARPFGSVGR,[R]	1xTMT6plex [N-Term];1	Q9Y4F5	Q9Y4F5 [485-495]	Q9Y4F5 1xTMT6plex [N	0,01	-0,66	0,12
[K],QDGPMPKPHSVSLNDTETI	1xTMT6plex [K7];1xTM	Q9P0L0	Q9P0L0 [155-173]	Q9P0L0 1xTMT6plex [K	0,04	-0,66	0,15
[R],GVSQEKEAQISSAIVSSVQS	2xTMT6plex [K6; K21];1	Q12872	Q12872 [907-927]	Q12872 2xTMT6plex [K	0,00	-0,66	0,12
[R],VTVHSTPVR,[R]	1xTMT6plex [N-Term];1	Q9NYZ3	Q9NYZ3 [484-492]	Q9NYZ3 1xTMT6plex [N	0,03	-0,66	0,14
[R],RPSTYGIPR,[L]	1xTMT6plex [N-Term];1	P78347	P78347 [410-418]	P78347 1xTMT6plex [N	0,00	-0,66	0,12
[K],KIDSPPIR,[R]	1xTMT6plex [K1];1xTM	Q7Z6B7	Q7Z6B7 [929-936]	Q7Z6B7 1xTMT6plex [K	0,02	-0,66	0,12
[R],KNSASAER,[D]	1xTMT6plex [K1];1xTM	Q96T37	Q96T37 [720-727]	Q96T37 1xTMT6plex [K	0,01	-0,66	0,12
[R],LDSSACLHAVGDK,[A]	1xCarbamidomethyl [C6	O94808	O94808 [242-254]	O94808 1xCarbamidom	0,03	-0,66	0,13
[R],NPVASPTQPSGTEK,[G]	1xTMT6plex [K14];1xTM	O43435	O43435 [323-336]	O43435 1xTMT6plex [K	0,01	-0,66	0,12
[K],LGIHEDSTNR,[R]	1xTMT6plex [N-Term];1	P08238	P08238 [439-448]	P08238 1xTMT6plex [N	0,04	-0,66	0,15
[R],EQMMNSSISSGSGSLR,[T]	2xOxidation [M3; M4];1	Q12959	Q12959 [562-577]	Q12959 2xOxidation [M	0,02	-0,66	0,13
[R],LESLSAATTFEPLEPVK,[D]	1xTMT6plex [K17];1xTM	Q8N1F7	Q8N1F7 [78-94]	Q8N1F7 1xTMT6plex [K	0,03	-0,66	0,14
[R],KSQSPSPK,[N]	2xTMT6plex [K1; K8];1x	Q99590	Q99590 [829-836]	Q99590 2xTMT6plex [K	0,01	-0,66	0,12
[R],LPSPTPENK,[D]	1xTMT6plex [K9];1xTM	Q5VWQ8	Q5VWQ8 [700-708]	Q5VWQ8 1xTMT6plex	0,01	-0,65	0,12
[R],RSGAMVK,[M]	1xTMT6plex [K7];1xTM	P14866	P14866 [28-34]	P14866 1xTMT6plex [K	0,02	-0,65	0,13
[R],IEQFSQEHSVK,[K]	1xTMT6plex [K11];1xTM	Q2TBE0	Q2TBE0 [67-77]	Q2TBE0 1xTMT6plex [K	0,02	-0,65	0,13
[R],LTVSSLQESGLK,[V]	1xTMT6plex [K12];1xTM	P21333	P21333 [2335-2346]	P21333 1xTMT6plex [K	0,05	-0,65	0,16
[R],SRSPSSEHR,[A]	1xTMT6plex [N-Term];1	Q9P1Y6	Q9P1Y6 [1163-1171]	Q9P1Y6 1xTMT6plex [N	0,04	-0,65	0,15
[R],NFSNSPGPYVFCITEK,[G]	1xCarbamidomethyl [C1	Q8NAP3	Q8NAP3 [126-141]	Q8NAP3 1xCarbamidon	0,02	-0,65	0,12
[R],RGSGGGSMSK,[T]	1xTMT6plex [K10];1xTM	O00287	O00287 [130-139]	O00287 1xTMT6plex [K	0,02	-0,65	0,12
[R],THKHSPEK,[R]	2xTMT6plex [K3; K8];1x	Q86X95	Q86X95 [361-368]	Q86X95 2xTMT6plex [K	0,02	-0,65	0,13
[R],ISVYYNEATGGK,[Y]	1xTMT6plex [K12];1xTM	P07437	P07437 [47-58]	P07437 1xTMT6plex [K	0,02	-0,65	0,13
[R],ISVYYNEATGGK,[Y]	1xTMT6plex [K12];1xTM	P07437	P07437 [47-58]	P07437 1xTMT6plex [K	0,02	-0,65	0,13
[R],GLSSSEK,[D]	1xTMT6plex [K7];1xTM	Q8IYH5	Q8IYH5 [87-93]	Q8IYH5 1xTMT6plex [K	0,01	-0,65	0,12
[R],LGSSTLGSK,[S]	1xTMT6plex [K9];1xTM	Q7Z460	Q7Z460 [278-286]	Q7Z460 1xTMT6plex [K	0,02	-0,65	0,13
[M],ARTKQTAR,[K]	1xTMT6plex [K4];1xTM	P84243; F	P84243 [2-9]; P0DPK5 [2-9]; Q5TEC	P84243 1xTMT6plex [K	0,01	-0,65	0,12
[R],DPQTPVLQTK,[H]	1xTMT6plex [K10];1xTM	Q9ULW0	Q9ULW0 [366-375]	Q9ULW0 1xTMT6plex [	0,01	-0,65	0,12
[R],DGLAPEKTSPDRDK,[K]	2xTMT6plex [K7; K14];1	Q7L4I2	Q7L4I2 [9-22]	Q7L4I2 2xTMT6plex [K1	0,02	-0,65	0,13
[R],HSFSAGPELLR,[Q]	1xTMT6plex [N-Term];1	Q5TH69	Q5TH69 [2078-2088]	Q5TH69 1xTMT6plex [N	0,05	-0,65	0,16
[R],TNSTGGSSGSSVGGGSGK	1xTMT6plex [K18];1xTM	Q8IUD2	Q8IUD2 [35-52]	Q8IUD2 1xTMT6plex [K	0,02	-0,65	0,13
[R],STQESLTAGGTDLKR,[E]	1xTMT6plex [K14];1xTM	Q15345	Q15345 [326-340]	Q15345 1xTMT6plex [K	0,02	-0,65	0,12

[R],SVSVDESRPELIFR,[L]	1xTMT6plex [N-Term];1	Q96BY7	Q96BY7 [495-508]	Q96BY7 1xTMT6plex [N	0,04	-0,65	0,15
[K],KLSEPSSLQYLPYR,[D]	1xTMT6plex [K1];1xTM	P04035	P04035 [502-515]	P04035 1xTMT6plex [K	0,01	-0,65	0,12
[R],RTDALTSSPGR,[D]	1xTMT6plex [N-Term];1	P49736	P49736 [34-44]	P49736 1xTMT6plex [N	0,01	-0,65	0,12
[R],KTPEVVQSTRPIIEGSISQG	2xTMT6plex [K1; K24];1	O75376	O75376 [1366-1389]	O75376 2xTMT6plex [K	0,02	-0,65	0,12
[K],LPEPSVLSEVTKPPPCLENS	1xCarbamidomethyl [C1	Q9BZL4	Q9BZL4 [479-503]	Q9BZL4 1xCarbamidom	0,01	-0,65	0,12
[R],KPASLMAPLK,[R]	1xOxidation [M6];2xTMT	Q8NE01	Q8NE01 [464-473]	Q8NE01 1xOxidation [M	0,03	-0,65	0,13
[R],STTPAPK,[S]	1xTMT6plex [K7];1xTM	Q9UQ35	Q9UQ35 [250-256]	Q9UQ35 1xTMT6plex [I	0,03	-0,65	0,13
[R],VMATTSASLK,[R]	1xTMT6plex [K10];1xTM	P30622	P30622 [299-308]	P30622 1xTMT6plex [K	0,01	-0,64	0,12
[R],KSFDASDTLALPR,[H]	1xTMT6plex [K1];1xTM	Q5JXC2	Q5JXC2 [302-314]	Q5JXC2 1xTMT6plex [K	0,05	-0,64	0,16
[K],SLRQSFR,[R]	1xTMT6plex [N-Term];1	Q15334	Q15334 [659-665]	Q15334 1xTMT6plex [N	0,01	-0,64	0,12
[R],GLNSSFETSPKK,[V]	2xTMT6plex [K11; K12]	Q8IV63	Q8IV63 [51-62]	Q8IV63 2xTMT6plex [K	0,00	-0,64	0,11
[K],QLESKSNEHDHSK,[S]	2xTMT6plex [K5; K13];1	Q13427	Q13427 [522-534]	Q13427 2xTMT6plex [K	0,01	-0,64	0,12
[R],LTQGIGR,[S]	1xTMT6plex [N-Term];1	Q7Z6Z7	Q7Z6Z7 [2539-2545]	Q7Z6Z7 1xTMT6plex [N	0,04	-0,64	0,16
[R],TASPPPPPK,[R]	1xTMT6plex [K9];1xTM	Q8IYB3	Q8IYB3 [614-622]	Q8IYB3 1xTMT6plex [K	0,01	-0,64	0,12
[R],KSSTSGSDFDTK,[K]	2xTMT6plex [K1; K12];1	P46100	P46100 [782-793]	P46100 2xTMT6plex [K	0,00	-0,64	0,12
[R],LTPESQGLLK,[V]	1xTMT6plex [K10];1xTM	Q9BRR0	Q9BRR0 [206-215]	Q9BRR0 1xTMT6plex [I	0,04	-0,64	0,15
[R],TVQSPQSK,[-]	1xTMT6plex [K8];1xTM	Q15695	Q15695 [472-479]	Q15695 1xTMT6plex [K	0,03	-0,64	0,13
[R],YSPSPPPK,[R]	1xTMT6plex [K8];1xTM	Q8IYB3	Q8IYB3 [604-611]	Q8IYB3 1xTMT6plex [K	0,02	-0,64	0,13
[K],TSATVGPK,[A]	1xTMT6plex [K8];1xTM	P16104	P16104 [121-128]	P16104 1xTMT6plex [K	0,03	-0,64	0,13
[R],ASSLNFLNKSVEEPTQPGG	1xTMT6plex [K9];1xTM	Q9H0B6	Q9H0B6 [580-605]	Q9H0B6 1xTMT6plex [K	0,00	-0,64	0,11
[K],FSVSPVVR,[V]	1xTMT6plex [N-Term];1	P13639	P13639 [499-506]	P13639 1xTMT6plex [N	0,02	-0,64	0,12
[R],SLTAHSLLPLAEK,[Q]	1xTMT6plex [K13];1xTM	Q86VI3	Q86VI3 [1424-1436]	Q86VI3 1xTMT6plex [K	0,04	-0,64	0,16
[K],TEVQAHSPSR,[K]	1xTMT6plex [N-Term];1	P11137	P11137 [1474-1483]	P11137 1xTMT6plex [N	0,01	-0,64	0,12
[K],ATVTPSPVKGK,[G]	2xTMT6plex [K9; K11];1	Q9H1E3	Q9H1E3 [176-186]	Q9H1E3 2xTMT6plex [K	0,00	-0,64	0,12
[R],ASQEANLLTLAQK,[A]	1xTMT6plex [K13];1xTM	Q6PJG2	Q6PJG2 [460-472]	Q6PJG2 1xTMT6plex [K	0,03	-0,64	0,14
[K],LRNSDIELR,[K]	1xTMT6plex [N-Term];1	Q12968; C	Q12968 [543-551]; Q14934 [529-537	Q12968 1xTMT6plex [N	0,05	-0,64	0,16
[R],KSPVGMLDLSSWSSPEVLF	1xTMT6plex [K1];1xTM	O95613	O95613 [2213-2231]	O95613 1xTMT6plex [K	0,04	-0,64	0,15
[R],NLVGRGSSPR,[G]	1xTMT6plex [N-Term];1	P55197	P55197 [673-682]	P55197 1xTMT6plex [N	0,01	-0,64	0,12
[R],TPGPPGLTTTPAPPDK,[L]	1xTMT6plex [K16];1xTM	Q7Z5J4	Q7Z5J4 [1068-1083]	Q7Z5J4 1xTMT6plex [K	0,00	-0,64	0,12
[R],SVSHGSNHTQKPDEQR,[S]	1xTMT6plex [K11];1xTM	Q9Y520	Q9Y520 [924-939]	Q9Y520 1xTMT6plex [K	0,02	-0,64	0,13
[R],FQHGHSR,[R]	1xTMT6plex [N-Term];1	Q96PV6	Q96PV6 [516-522]	Q96PV6 1xTMT6plex [N	0,02	-0,64	0,13
[R],SGAGSSPETK,[E]	1xTMT6plex [K10];1xTM	Q9UQ35	Q9UQ35 [1214-1223]	Q9UQ35 1xTMT6plex [I	0,04	-0,64	0,15
[R],LSASSTGSTR,[S]	1xTMT6plex [N-Term];1	P56945	P56945 [427-436]	P56945 1xTMT6plex [N	0,02	-0,64	0,13
[R],VLRPPGGGSNFSLGFDEPT	1xTMT6plex [N-Term];1	Q9UK76	Q9UK76 [20-43]	Q9UK76 1xTMT6plex [N	0,00	-0,64	0,12
[R],FSREEFPTLQAAGDQDK,[A	1xTMT6plex [K17];1xTM	P48634	P48634 [165-181]	P48634 1xTMT6plex [K	0,00	-0,64	0,12
[R],ESSILAK,[L]	1xTMT6plex [K7];1xTM	O95782	O95782 [609-615]	O95782 1xTMT6plex [K	0,02	-0,64	0,13
[R],SPSDLHISPLAK,[K]	1xTMT6plex [K12];1xTM	O95785	O95785 [1127-1138]	O95785 1xTMT6plex [K	0,00	-0,64	0,12
[R],KRPESGSIR,[K]	1xTMT6plex [K1];1xTM	O75044	O75044 [839-847]	O75044 1xTMT6plex [K	0,01	-0,63	0,12
[R],SRSPATAK,[R]	1xTMT6plex [K8];1xTM	Q9UQ35	Q9UQ35 [484-491]	Q9UQ35 1xTMT6plex [I	0,05	-0,63	0,16
[R],SPPADAIPK,[S]	1xTMT6plex [K9];1xTM	P18754	P18754 [11-19]	P18754 1xTMT6plex [K	0,01	-0,63	0,12
[K],TVSPPIR,[K]	1xTMT6plex [N-Term];1	Q9UHB6	Q9UHB6 [607-613]	Q9UHB6 1xTMT6plex [I	0,03	-0,63	0,13
[R],SRSPHSSQSR,[N]	1xTMT6plex [N-Term];1	Q96T58	Q96T58 [242-251]	Q96T58 1xTMT6plex [N	0,04	-0,63	0,15
[R],SPAAGSSQSSGWPNDVDA	1xTMT6plex [K24];1xTM	Q14938	Q14938 [301-324]	Q14938 1xTMT6plex [K	0,03	-0,63	0,14
[K],QLVAGNSPK,[K]	1xTMT6plex [K9];1xTM	Q96EU6	Q96EU6 [67-75]	Q96EU6 1xTMT6plex [K	0,02	-0,63	0,13
[R],SKSEDMDNVQSK,[R]	2xTMT6plex [K2; K12];1	Q6VMQ6	Q6VMQ6 [557-568]	Q6VMQ6 2xTMT6plex [	0,01	-0,63	0,12
[R],SGSSPEHAR,[H]	1xTMT6plex [N-Term];1	Q96PX6	Q96PX6 [322-330]	Q96PX6 1xTMT6plex [N	0,04	-0,63	0,15
[K],GLECSDWKPEAGLSPPR,[K	1xCarbamidomethyl [C4	O75764	O75764 [102-118]	O75764 1xCarbamidom	0,02	-0,63	0,12
[K],TVDSPKR,[R]	1xTMT6plex [K6];1xTM	Q9NYV4	Q9NYV4 [212-218]	Q9NYV4 1xTMT6plex [I	0,00	-0,63	0,11
[R],GSPSSHLLGADHGLR,[K]	1xTMT6plex [N-Term];1	Q6NWY9	Q6NWY9 [763-777]	Q6NWY9 1xTMT6plex	0,01	-0,63	0,12
[R],LKSDERPVHIK,[-]	2xTMT6plex [K2; K11];1	Q9UPN9	Q9UPN9 [1117-1127]	Q9UPN9 2xTMT6plex [I	0,00	-0,63	0,11
[K],TGKEYIPGQPPLSQSSDSS	1xTMT6plex [K3];1xTM	P07814	P07814 [868-889]	P07814 1xTMT6plex [K	0,04	-0,63	0,15
[R],DLSFLLDKK,[A]	2xTMT6plex [K8; K9];1x	O75151	O75151 [703-711]	O75151 2xTMT6plex [K	0,01	-0,63	0,12
[R],KQSDSDLIPER,[A]	1xTMT6plex [K1];1xTM	Q9P2M4	Q9P2M4 [89-99]	Q9P2M4 1xTMT6plex [I	0,03	-0,63	0,14
[K],VIIPSPPKDDSPQLSEELR,[I	1xTMT6plex [K8];1xTM	Q96JM2	Q96JM2 [1743-1761]	Q96JM2 1xTMT6plex [K	0,01	-0,63	0,12
[R],KISVVSATK,[G]	2xTMT6plex [K1; K9];1x	Q9UKV3	Q9UKV3 [823-831]	Q9UKV3 2xTMT6plex [I	0,03	-0,63	0,14
[K],LLKEGEEPTVYSDEEEPKD	2xTMT6plex [K3; K18];1	O00264	O00264 [170-192]	O00264 2xTMT6plex [K	0,00	-0,63	0,11
[R],RSPVPAQIAITVPK,[T]	1xTMT6plex [K14];1xTM	O43432	O43432 [494-507]	O43432 1xTMT6plex [K	0,01	-0,63	0,12

[R],SVSPPPK,[R]	1xTMT6plex [K7];1xTM	Q13247	Q13247 [314-320]	Q13247 1xTMT6plex [K	0,01	-0,63	0,12
[R],ISSSSFSR,[V]	1xTMT6plex [N-Term];1	P05787	P05787 [33-40]	P05787 1xTMT6plex [N	0,02	-0,63	0,13
[R],TQSSLTVSEVLTR,[R]	1xTMT6plex [N-Term];1	Q86V48	Q86V48 [993-1005]	Q86V48 1xTMT6plex [N	0,04	-0,63	0,16
[R],SPYSGPK,[F]	1xTMT6plex [K7];1xTM	Q14498	Q14498 [97-103]	Q14498 1xTMT6plex [K	0,02	-0,63	0,13
[R],KASGSENEGDYNPGR,[K]	1xTMT6plex [K1];1xTM	Q02880	Q02880 [1548-1562]	Q02880 1xTMT6plex [K	0,01	-0,63	0,12
[K],KTESHKPGK,[N]	3xTMT6plex [K1; K6; K	Q8IUE6	Q8IUE6 [120-128]	Q8IUE6 3xTMT6plex [K	0,01	-0,63	0,12
[R],KSSVNGSSATSSG,[-]	1xTMT6plex [K1];1xTM	P35226	P35226 [314-326]	P35226 1xTMT6plex [K	0,02	-0,63	0,13
[R],RPSLGELLLR,[H]	1xTMT6plex [N-Term];1	Q8N612	Q8N612 [857-866]	Q8N612 1xTMT6plex [N	0,01	-0,63	0,12
[R],GHYTIGK,[E]	1xTMT6plex [K7];1xTM	Q71U36; I	Q71U36 [106-112]; P68366 [106-112	Q71U36 1xTMT6plex [K	0,05	-0,63	0,16
[R],GLLSNHTGSPR,[S]	1xTMT6plex [N-Term];1	Q13111	Q13111 [764-774]	Q13111 1xTMT6plex [N	0,04	-0,62	0,15
[R],AILGSYR,[K]	1xTMT6plex [N-Term];1	Q96RK0	Q96RK0 [1385-1391]	Q96RK0 1xTMT6plex [N	0,03	-0,62	0,14
[R],DNSPPPAFKPEPPK,[A]	2xTMT6plex [K9; K14];1	Q9UDY2	Q9UDY2 [984-997]	Q9UDY2 2xTMT6plex [I	0,03	-0,62	0,13
[R],SLPITIEMLK,[V]	1xTMT6plex [K10];1xTM	Q8IZ21	Q8IZ21 [464-473]	Q8IZ21 1xTMT6plex [K	0,04	-0,62	0,15
[R],TSSPKPR,[F]	1xTMT6plex [K5];1xTM	P11532	P11532 [289-295]	P11532 1xTMT6plex [K	0,01	-0,62	0,12
[R],AAPTTPPPPVK,[R]	1xTMT6plex [K11];1xTM	Q9NZQ3	Q9NZQ3 [177-187]	Q9NZQ3 1xTMT6plex [	0,02	-0,62	0,13
[R],DKSPPPLSWGK,[S]	2xTMT6plex [K2; K11];1	Q9UK61	Q9UK61 [1649-1659]	Q9UK61 2xTMT6plex [K	0,02	-0,62	0,13
[K],ASVHSSGR,[G]	1xTMT6plex [N-Term];1	Q9Y5B9	Q9Y5B9 [1022-1029]	Q9Y5B9 1xTMT6plex [N	0,01	-0,62	0,12
[R],KDTDDIESPK,[R]	2xTMT6plex [K1; K10];1	Q9UPQ0	Q9UPQ0 [162-171]	Q9UPQ0 2xTMT6plex [	0,01	-0,62	0,12
[R],KDSGSPNPAR,[G]	1xTMT6plex [K1];1xTM	P01106	P01106 [157-166]	P01106 1xTMT6plex [K	0,04	-0,62	0,16
[R],TKSSDSDLSPPR,[R]	1xTMT6plex [K2];1xTM	Q9BRD0	Q9BRD0 [399-410]	Q9BRD0 1xTMT6plex [I	0,04	-0,62	0,15
[R],AESIDKK,[I]	2xTMT6plex [K6; K7];1x	Q9NZZ3	Q9NZZ3 [28-34]	Q9NZZ3 2xTMT6plex [K	0,03	-0,62	0,14
[R],YHSLGNISR,[G]	1xTMT6plex [N-Term];1	Q09019	Q09019 [543-551]	Q09019 1xTMT6plex [N	0,02	-0,62	0,12
[R],RPPVLDESWIR,[E]	1xTMT6plex [N-Term];1	O94901	O94901 [131-141]	O94901 1xTMT6plex [N	0,00	-0,62	0,12
[R],SVYFKPSLTPSGEFR,[K]	1xTMT6plex [K5];1xTM	P49790	P49790 [380-394]	P49790 1xTMT6plex [K	0,00	-0,62	0,12
[R],TSIPVHSK,[Q]	1xTMT6plex [K8];1xTM	Q96QT4	Q96QT4 [1487-1494]	Q96QT4 1xTMT6plex [K	0,01	-0,62	0,12
[R],RDSASYR,[D]	1xTMT6plex [N-Term];1	Q92841	Q92841 [597-603]	Q92841 1xTMT6plex [N	0,02	-0,62	0,13
[R],SSLSSHSHQSQIYR,[S]	1xTMT6plex [N-Term];1	O15027	O15027 [1368-1381]	O15027 1xTMT6plex [N	0,02	-0,62	0,13
[R],SHKDEVVK,[I]	2xTMT6plex [K3; K8];1x	P46940	P46940 [804-811]	P46940 2xTMT6plex [K	0,02	-0,62	0,13
[R],KESSEHYQR,[D]	1xTMT6plex [K1];1xTM	Q9BR77	Q9BR77 [208-216]	Q9BR77 1xTMT6plex [K	0,03	-0,62	0,14
[K],VKSGTPPR,[Q]	1xTMT6plex [K2];1xTM	Q9UQ35	Q9UQ35 [844-851]	Q9UQ35 1xTMT6plex [I	0,01	-0,62	0,12
[K],SESDKDVK,[Q]	2xTMT6plex [K5; K8];1x	Q05519	Q05519 [412-419]	Q05519 2xTMT6plex [K	0,00	-0,62	0,11
[R],QLSPQSK,[S]	1xTMT6plex [K7];1xTM	Q86VM9	Q86VM9 [891-897]	Q86VM9 1xTMT6plex [I	0,02	-0,62	0,12
[R],ATVASSTQK,[F]	1xTMT6plex [K9];1xTM	Q9UHD8	Q9UHD8 [54-62]	Q9UHD8 1xTMT6plex [	0,04	-0,62	0,15
[R],GSPPTSSNIVQGQIK,[Q]	1xTMT6plex [K15];1xTM	Q9Y4E6	Q9Y4E6 [934-948]	Q9Y4E6 1xTMT6plex [K	0,02	-0,62	0,12
[R],GSPGGSLGGALQK,[E]	1xTMT6plex [K13];1xTM	Q9Y283	Q9Y283 [660-672]	Q9Y283 1xTMT6plex [K	0,01	-0,62	0,12
[R],KNSTDLDSAPEDPTSPK,[R	2xTMT6plex [K1; K17];1	Q96RK0	Q96RK0 [1395-1411]	Q96RK0 2xTMT6plex [K	0,01	-0,62	0,12
[R],GSPTTGFIEQK,[G]	1xTMT6plex [K11];1xTM	Q6ZRV2	Q6ZRV2 [891-901]	Q6ZRV2 1xTMT6plex [K	0,04	-0,62	0,15
[K],GSRPPLILQSQSLPCSSPR,	1xCarbamidomethyl [C1	Q6KC79	Q6KC79 [290-308]	Q6KC79 1xCarbamidom	0,00	-0,62	0,12
[R],EDEGPPGYPEPLAPGHGSI	1xTMT6plex [K25];1xTM	P19484	P19484 [406-430]	P19484 1xTMT6plex [K	0,01	-0,61	0,12
[R],VSPCHSR,[Q]	1xCarbamidomethyl [C4	Q674X7	Q674X7 [309-315]	Q674X7 1xCarbamidom	0,00	-0,61	0,12
[R],QEPSPKPNNK,[T]	2xTMT6plex [K6; K10];1	Q8N684	Q8N684 [57-66]	Q8N684 2xTMT6plex [K	0,02	-0,61	0,13
[R],SRSPHSSQSR,[N]	1xTMT6plex [N-Term];1	Q96T58	Q96T58 [242-251]	Q96T58 1xTMT6plex [N	0,04	-0,61	0,15
[K],TDSSPNQAR,[A]	1xTMT6plex [N-Term];1	P26599	P26599 [138-146]	P26599 1xTMT6plex [N	0,03	-0,61	0,13
[K],SFATASHR,[N]	1xTMT6plex [N-Term];1	Q9NYF8	Q9NYF8 [422-429]	Q9NYF8 1xTMT6plex [N	0,01	-0,61	0,12
[R],IQTSLTSASLGSADENSVA	1xTMT6plex [K26];1xTM	P49815	P49815 [991-1016]	P49815 1xTMT6plex [K	0,03	-0,61	0,14
[R],KSSRSPSPR,[S]	1xTMT6plex [K1];1xTM	Q8WXA9	Q8WXA9 [402-410]	Q8WXA9 1xTMT6plex [	0,02	-0,61	0,13
[R],AKSVEDDAEGHLIYHVGDW	1xTMT6plex [K2];1xTM	P49760	P49760 [140-162]	P49760 1xTMT6plex [K	0,02	-0,61	0,13
[R],KSSVNGSSATSSG,[-]	1xTMT6plex [K1];1xTM	P35226	P35226 [314-326]	P35226 1xTMT6plex [K	0,01	-0,61	0,12
[R],WDETPASQMGGSTPVLTP	1xOxidation [M9];1xTM	O75533	O75533 [338-357]	O75533 1xOxidation [M	0,01	-0,61	0,12
[K],TVSPALISR,[F]	1xTMT6plex [N-Term];1	Q86VP6	Q86VP6 [374-382]	Q86VP6 1xTMT6plex [N	0,00	-0,61	0,12
[R],KGSSPSR,[S]	1xTMT6plex [K1];1xTM	Q8IYB3	Q8IYB3 [662-668]	Q8IYB3 1xTMT6plex [K	0,05	-0,61	0,16
[R],SVSANPK,[Q]	1xTMT6plex [K7];1xTM	Q7KZI7	Q7KZI7 [398-404]	Q7KZI7 1xTMT6plex [K	0,01	-0,61	0,12
[R],AIQLTPGESSK,[T]	1xTMT6plex [K11];1xTM	P80192	P80192 [561-571]	P80192 1xTMT6plex [K	0,04	-0,61	0,15
[R],ASHTLLPSHR,[L]	1xTMT6plex [N-Term];1	Q9UKV3	Q9UKV3 [560-569]	Q9UKV3 1xTMT6plex [I	0,03	-0,61	0,13
[R],AEEGKSPFR,[E]	1xTMT6plex [K5];1xTM	O15014	O15014 [753-761]	O15014 1xTMT6plex [K	0,00	-0,61	0,12
[R],ASSVLAQR,[R]	1xTMT6plex [N-Term];1	O14681	O14681 [45-52]	O14681 1xTMT6plex [N	0,01	-0,61	0,12
[K],FSPVTPKFTPVASK,[F]	2xTMT6plex [K7; K14];1	Q15942	Q15942 [266-279]	Q15942 2xTMT6plex [K	0,00	-0,61	0,11

[K],SFQQSSLSR,[D]	1xTMT6plex [N-Term];1	P43243	P43243 [4-12]	P43243 1xTMT6plex [N	0,03	-0,61	0,14
[K],SPPPKSK,[S]	2xTMT6plex [K5; K7];1x	Q9HCE5	Q9HCE5 [399-405]	Q9HCE5 2xTMT6plex [	0,00	-0,61	0,12
[K],KESKEEETSIDVAGKPNEV	4xTMT6plex [K1; K4; K	P53985	P53985 [459-479]	P53985 4xTMT6plex [K	0,01	-0,61	0,12
[K],SPNELVDDLFKGAK,[E]	2xTMT6plex [K11; K14]	Q9UNZ2	Q9UNZ2 [114-127]	Q9UNZ2 2xTMT6plex [	0,01	-0,61	0,12
[R],KATEGSGSMR,[G]	1xTMT6plex [K1];1xTM	O94874	O94874 [424-433]	O94874 1xTMT6plex [K	0,02	-0,61	0,12
[K],GAFSVVR,[R]	1xTMT6plex [N-Term];1	Q13557;	Q13557 [23-29]; Q13554 [23-29]	Q13557 1xTMT6plex [N	0,01	-0,61	0,12
[R],GPSPAPASSPK,[R]	1xTMT6plex [K11];1xT	Q9H7N4	Q9H7N4 [717-727]	Q9H7N4 1xTMT6plex [	0,03	-0,61	0,13
[R],LLLERPSPIR,[D]	1xTMT6plex [N-Term];1	Q96T37	Q96T37 [694-703]	Q96T37 1xTMT6plex [N	0,02	-0,61	0,13
[K],VSLGGASR,[K]	1xTMT6plex [N-Term];1	Q15386	Q15386 [14-21]	Q15386 1xTMT6plex [N	0,04	-0,61	0,15
[K],TASLPGYGR,[N]	1xTMT6plex [N-Term];1	O14639	O14639 [653-661]	O14639 1xTMT6plex [N	0,01	-0,61	0,12
[R],NKDTPACSR,[R]	1xCarbamidomethyl [C7	P42695	P42695 [1478-1486]	P42695 1xCarbamidom	0,04	-0,60	0,15
[R],LSLSTSK,[L]	1xTMT6plex [K7];1xTM	P54886	P54886 [426-432]	P54886 1xTMT6plex [K	0,02	-0,60	0,13
[R],LSLSTSK,[L]	1xTMT6plex [K7];1xTM	P54886	P54886 [426-432]	P54886 1xTMT6plex [K	0,02	-0,60	0,13
[R],RESLSYIPK,[G]	1xTMT6plex [K9];1xTM	A1L390	A1L390 [739-747]	A1L390 1xTMT6plex [K	0,05	-0,60	0,16
[R],LSSTDDGYIDLQFK,[K]	1xTMT6plex [K14];1xT	Q96A57	Q96A57 [22-35]	Q96A57 1xTMT6plex [K	0,03	-0,60	0,14
[K],AGSKIPTPK,[G]	2xTMT6plex [K4; K9];1x	O60308	O60308 [903-911]	O60308 2xTMT6plex [K	0,01	-0,60	0,12
[R],TNSPSSAK,[K]	1xTMT6plex [K8];1xTM	Q14669	Q14669 [83-90]	Q14669 1xTMT6plex [K	0,03	-0,60	0,14
[R],KGSPCDTLASSTEK,[R]	1xCarbamidomethyl [C5	Q15788	Q15788 [20-33]	Q15788 1xCarbamidom	0,01	-0,60	0,12
[R],VMATTSASLK,[R]	1xOxidation [M2];1xTM	P30622	P30622 [299-308]	P30622 1xOxidation [M	0,05	-0,60	0,16
[R],VSPSPSQESLSSSK,[S]	1xTMT6plex [K14];1xT	Q5JSH3	Q5JSH3 [560-573]	Q5JSH3 1xTMT6plex [K	0,03	-0,60	0,14
[R],SPGGLGAAQLK,[E]	1xTMT6plex [K11];1xT	Q03828	Q03828 [127-137]	Q03828 1xTMT6plex [K	0,01	-0,60	0,12
[K],AKPEGSPR,[R]	1xTMT6plex [K2];1xTM	Q86V59	Q86V59 [423-430]	Q86V59 1xTMT6plex [K	0,02	-0,60	0,13
[K],IHGTGLELTTK,[Q]	1xTMT6plex [K11];1xT	Q68DQ2	Q68DQ2 [1872-1882]	Q68DQ2 1xTMT6plex [	0,03	-0,60	0,13
[K],KFELLPTPPLSPSR,[GR]	1xTMT6plex [K1];1xTM	P01106	P01106 [52-65]	P01106 1xTMT6plex [K	0,03	-0,60	0,13
[K],KFELLPTPPLSPSR,[GR]	1xTMT6plex [K1];1xTM	P01106	P01106 [52-65]	P01106 1xTMT6plex [K	0,03	-0,60	0,13
[K],LEVFTTPK,[K]	1xTMT6plex [K8];1xTM	Q8WYP5	Q8WYP5 [1252-1259]	Q8WYP5 1xTMT6plex [	0,05	-0,60	0,16
[R],DTPGHGSGWAETPR,[T]	1xTMT6plex [N-Term];1	O75533	O75533 [302-315]	O75533 1xTMT6plex [N	0,05	-0,60	0,16
[GR],AASAPAKESPR,[K]	1xTMT6plex [K7];1xTM	Q13428	Q13428 [373-383]; [438-448]	Q13428 1xTMT6plex [K	0,01	-0,60	0,12
[R],KTSDANETEDHLESLICK,[V	1xCarbamidomethyl [C1	Q09161	Q09161 [20-37]	Q09161 1xCarbamidom	0,00	-0,60	0,12
[R],LPSSPVYEDAASFK,[A]	1xTMT6plex [K14];1xT	Q14247	Q14247 [415-428]	Q14247 1xTMT6plex [K	0,01	-0,60	0,12
[R],KTSDANETEDHLESLICK,[V	1xCarbamidomethyl [C1	Q09161	Q09161 [20-37]	Q09161 1xCarbamidom	0,00	-0,60	0,12
[R],TESEVPPRPASPK,[V]	1xTMT6plex [K13];1xT	Q9Y3L3	Q9Y3L3 [534-546]	Q9Y3L3 1xTMT6plex [K	0,04	-0,60	0,15
[R],SCFESSPDPELKSR,[T]	1xCarbamidomethyl [C2	Q9UQ35	Q9UQ35 [871-884]	Q9UQ35 1xCarbamidor	0,00	-0,60	0,11
[R],KQKGSEENLDEAR,[E]	2xTMT6plex [K1; K3];1x	P35611	P35611 [582-594]	P35611 2xTMT6plex [K	0,03	-0,60	0,14
[R],SRSHTPSR,[R]	1xTMT6plex [N-Term];2	P18583	P18583 [1929-1936]	P18583 1xTMT6plex [N	0,04	-0,60	0,15
[K],LSPTSLVHPVMSTLPELSCK	1xCarbamidomethyl [C1	Q9UGP4	Q9UGP4 [383-402]	Q9UGP4 1xCarbamidor	0,03	-0,60	0,13
[M],AHAASQLK,[K]	1xTMT6plex [K8];1xTM	Q12955	Q12955 [2-9]	Q12955 1xTMT6plex [K	0,02	-0,60	0,12
[R],DLFAFQESPPR,[F]	1xTMT6plex [N-Term];1	P51957	P51957 [556-566]	P51957 1xTMT6plex [N	0,03	-0,60	0,14
[R],KSQSPSPK,[N]	2xTMT6plex [K1; K8];1x	Q99590	Q99590 [829-836]	Q99590 2xTMT6plex [K	0,01	-0,60	0,12
[R],ASPSKPASAPASR,[S]	1xTMT6plex [K5];1xTM	P27816	P27816 [786-798]	P27816 1xTMT6plex [K	0,02	-0,60	0,13
[R],SVSPPPK,[R]	1xTMT6plex [K7];1xTM	Q13247	Q13247 [314-320]	Q13247 1xTMT6plex [K	0,01	-0,60	0,12
[R],EVSSRPSTPGLSVVSGISA	1xPhospho [S18];1xTM	Q14C86	Q14C86 [755-781]	Q14C86 1xPhospho [S7	0,01	-0,59	0,12
[R],EVSSRPSTPGLSVVSGISA	1xPhospho [S15];1xTM	Q14C86	Q14C86 [755-781]	Q14C86 1xPhospho [S7	0,01	-0,59	0,12
[R],RKDDSYFDR,[Y]	1xTMT6plex [K2];1xTM	Q9HCD5	Q9HCD5 [139-147]	Q9HCD5 1xTMT6plex [	0,03	-0,59	0,13
[K],HGLAHDEMKSPR,[E]	1xOxidation [M8];1xTM	Q9Y2W1	Q9Y2W1 [689-700]	Q9Y2W1 1xOxidation [M	0,03	-0,59	0,14
[K],IWASSMDLLCTADR,[D]	1xCarbamidomethyl [C1	Q12923	Q12923 [298-311]	Q12923 1xCarbamidom	0,00	-0,59	0,12
[R],KLSHDAESEREDPAPAK,[K	2xTMT6plex [K1; K17];1	Q92766	Q92766 [173-189]	Q92766 2xTMT6plex [K	0,00	-0,59	0,12
[R],ISKPSVSAFFTGPEELK,[D]	2xTMT6plex [K3; K17];1	Q8WVZ9	Q8WVZ9 [25-41]	Q8WVZ9 2xTMT6plex [	0,01	-0,59	0,12
[R],VKEEPPSPPQSPR,[V]	1xTMT6plex [K2];1xTM	Q00613	Q00613 [297-309]	Q00613 1xTMT6plex [K	0,00	-0,59	0,11
[R],SSSSLLASPGHISVK,[E]	1xTMT6plex [K15];1xT	A0FGR8	A0FGR8 [736-750]	A0FGR8 1xTMT6plex [	0,03	-0,59	0,13
[R],CSPVPGLSSSPSGSPLHGK	1xCarbamidomethyl [C1	Q9H6U6	Q9H6U6 [479-497]	Q9H6U6 1xCarbamidor	0,01	-0,59	0,12
[K],LVPEEVTSTVTK,[S]	1xTMT6plex [K12];1xT	Q03188	Q03188 [508-519]	Q03188 1xTMT6plex [K	0,05	-0,59	0,16
[R],SSTPSPSPLNLSSSR,[N]	1xTMT6plex [N-Term];1	P37275	P37275 [700-714]	P37275 1xTMT6plex [N	0,00	-0,59	0,11
[R],KSLSDSESDDSK,[S]	2xTMT6plex [K1; K12];1	Q13185	Q13185 [92-103]	Q13185 2xTMT6plex [K	0,00	-0,59	0,12
[R],SFSLASSSNSPISQR,[R]	1xTMT6plex [N-Term];1	Q9BXB4	Q9BXB4 [172-186]	Q9BXB4 1xTMT6plex [N	0,02	-0,59	0,13
[R],ASLSDIGFGK,[L]	1xTMT6plex [K10];1xT	Q07002	Q07002 [131-140]	Q07002 1xTMT6plex [K	0,01	-0,59	0,12
[K],KSQDLESVQEVGGSYWQR	1xTMT6plex [K1];1xTM	Q9H583	Q9H583 [1189-1206]	Q9H583 1xTMT6plex [K	0,05	-0,59	0,16

[R],YSVLNNDDYFADVSPLR,[A	1xTMT6plex [N-Term];1	Q1ED39	Q1ED39 [29-45]	Q1ED39 1xTMT6plex [N	0,04	-0,59	0,15
[R],DPTPRPPGLPEEATALAAP	1xTMT6plex [N-Term];1	Q96GP6	Q96GP6 [723-745]	Q96GP6 1xTMT6plex [N	0,03	-0,59	0,14
[K],ICSSHSLPLSR,[T]	1xCarbamidomethyl [C2	Q68DA7	Q68DA7 [196-206]	Q68DA7 1xCarbamidom	0,03	-0,59	0,13
[R],KADSLSSPR,[K]	1xTMT6plex [K1];1xTM	Q6BDS2	Q6BDS2 [429-437]	Q6BDS2 1xTMT6plex [	0,00	-0,59	0,11
[K],HLTHCQSR,[H]	1xCarbamidomethyl [C5	Q96JM3	Q96JM3 [783-790]	Q96JM3 1xCarbamidom	0,02	-0,59	0,13
[R],SPSTLLPK,[K]	1xTMT6plex [K8];1xTM	P27816	P27816 [825-832]	P27816 1xTMT6plex [K	0,01	-0,59	0,12
[R],ASTIFLSKSQTDVR,[E]	1xTMT6plex [K8];1xTM	Q8ND76;	Q8ND76 [65-78]; Q8N7R7 [87-100]	Q8ND76 1xTMT6plex [K	0,00	-0,59	0,12
[K],KDGSASAAAK,[K]	2xTMT6plex [K1; K10];1	P43243	P43243 [702-711]	P43243 2xTMT6plex [K	0,01	-0,59	0,12
[R],GLQLTPGIGGMQQHFFDDI	1xOxidation [M11];1xTM	P12270	P12270 [2112-2132]	P12270 1xOxidation [M	0,01	-0,58	0,12
[R],SRSHSPMSNR,[R]	1xTMT6plex [N-Term];2	Q13595	Q13595 [96-105]	Q13595 1xTMT6plex [N	0,01	-0,58	0,12
[R],YLAPSGPSGTLK,[A]	1xTMT6plex [K12];1xTM	Q16658	Q16658 [230-241]	Q16658 1xTMT6plex [K	0,02	-0,58	0,12
[R],EALGLGPPAAQLTPPPAPV	1xTMT6plex [N-Term];1	Q8IY67	Q8IY67 [451-472]	Q8IY67 1xTMT6plex [N	0,03	-0,58	0,14
[R],HASTSSPADK,[A]	1xTMT6plex [K10];1xTM	Q5T4S7	Q5T4S7 [1758-1767]	Q5T4S7 1xTMT6plex [K	0,02	-0,58	0,12
[R],TPSTKPK,[Q]	2xTMT6plex [K5; K7];1x	Q5M775	Q5M775 [149-155]	Q5M775 2xTMT6plex [K	0,02	-0,58	0,13
[R],SLHSAHSLASR,[R]	1xTMT6plex [N-Term];1	O15027	O15027 [1356-1366]	O15027 1xTMT6plex [N	0,01	-0,58	0,12
[R],GTSPVTQVK,[D]	1xTMT6plex [K9];1xTM	P35712	P35712 [409-417]	P35712 1xTMT6plex [K	0,05	-0,58	0,16
[K],SKSATTTPSGSPR,[T]	1xTMT6plex [K2];1xTM	Q2M2I8	Q2M2I8 [668-680]	Q2M2I8 1xTMT6plex [K	0,05	-0,58	0,16
[R],ISGPECTTPK,[A]	1xCarbamidomethyl [C6	Q5VWG9	Q5VWG9 [426-435]	Q5VWG9 1xCarbamido	0,01	-0,58	0,12
[K],FATLSLHDR,[K]	1xTMT6plex [N-Term];1	Q8TBZ3	Q8TBZ3 [461-469]	Q8TBZ3 1xTMT6plex [N	0,03	-0,58	0,14
[R],LDQSDEDFKPR,[I]	1xTMT6plex [K9];1xTM	Q9P2E5	Q9P2E5 [61-71]	Q9P2E5 1xTMT6plex [K	0,02	-0,58	0,13
[K],GKAESGKEEDK,[K]	3xTMT6plex [K2; K7; K	Q99442	Q99442 [113-123]	Q99442 3xTMT6plex [K	0,03	-0,58	0,13
[R],GKATPAEEEK,[T]	2xTMT6plex [K2; K10];1	Q15269	Q15269 [259-268]	Q15269 2xTMT6plex [K	0,02	-0,58	0,12
[R],SSPGAGPSDHHSASR,[D]	1xTMT6plex [N-Term];1	Q9Y2K7	Q9Y2K7 [739-753]	Q9Y2K7 1xTMT6plex [N	0,03	-0,58	0,14
[R],KLSDDPCPVESK,[K]	1xCarbamidomethyl [C7	O60244	O60244 [615-626]	O60244 1xCarbamidom	0,02	-0,58	0,13
[R],ASPGGVSTSSSDGK,[A]	1xTMT6plex [K14];1xTM	P54259	P54259 [33-46]	P54259 1xTMT6plex [K	0,02	-0,58	0,12
[R],TGSQHGPQNAAAATFQR,[	1xTMT6plex [N-Term];1	Q9UQB3	Q9UQB3 [472-488]	Q9UQB3 1xTMT6plex [	0,01	-0,58	0,12
[R],KPSHTSAVSIAGK,[E]	2xTMT6plex [K1; K13];1	Q9HC35	Q9HC35 [92-104]	Q9HC35 2xTMT6plex [K	0,02	-0,58	0,12
[K],RPVSDLLSGK,[K]	1xTMT6plex [K10];1xTM	Q16825	Q16825 [817-826]	Q16825 1xTMT6plex [K	0,03	-0,58	0,14
[R],WDETPKTER,[D]	1xTMT6plex [K6];1xTM	O75533	O75533 [293-301]	O75533 1xTMT6plex [K	0,04	-0,58	0,15
[R],AKTQTPPVSPAPQPTEER,[	1xTMT6plex [K2];1xTM	Q14247	Q14247 [397-414]	Q14247 1xTMT6plex [K	0,02	-0,58	0,12
[K],SPCPQEKSK,[D]	1xCarbamidomethyl [C3	Q9UQ35	Q9UQ35 [1014-1022]	Q9UQ35 1xCarbamidor	0,00	-0,58	0,11
[K],KQNSLGSSDTLK,[K]	2xTMT6plex [K1; K12];1	Q99698	Q99698 [2146-2157]	Q99698 2xTMT6plex [K	0,03	-0,58	0,14
[R],SPSVDSARK,[E]	1xTMT6plex [K9];1xTM	O00192	O00192 [343-351]	O00192 1xTMT6plex [K	0,00	-0,58	0,12
[R],MLQAISPK,[Q]	1xOxidation [M1];1xTM	P49585; Q	P49585 [310-317]; Q9Y5K3 [310-317	P49585 1xOxidation [M	0,03	-0,58	0,14
[K],SPHKDLGHR,[K]	1xTMT6plex [K4];1xTM	O94888	O94888 [350-358]	O94888 1xTMT6plex [K	0,00	-0,58	0,12
[K],YKDNPFSLGESFGSR,[W]	1xTMT6plex [K2];1xTM	Q8N6H7	Q8N6H7 [358-372]	Q8N6H7 1xTMT6plex [K	0,01	-0,58	0,12
[R],DSFSHSPGAVSSLK,[V]	1xTMT6plex [K14];1xTM	Q9H6R7	Q9H6R7 [685-698]	Q9H6R7 1xTMT6plex [K	0,01	-0,58	0,12
[R],ASSNKDFTPNR,[D]	1xTMT6plex [K5];1xTM	Q7Z6E9	Q7Z6E9 [1461-1471]	Q7Z6E9 1xTMT6plex [K	0,00	-0,58	0,12
[K],IDSNISPK,[K]	1xTMT6plex [K8];1xTM	Q9H501	Q9H501 [148-155]	Q9H501 1xTMT6plex [K	0,02	-0,58	0,13
[R],SSLPNGEGLQLK,[E]	1xTMT6plex [K12];1xTM	Q9ULR3	Q9ULR3 [123-134]	Q9ULR3 1xTMT6plex [K	0,01	-0,58	0,12
[R],VTQHESDNENEIQIQNK,[L]	1xTMT6plex [K17];1xTM	Q5VZL5	Q5VZL5 [117-133]	Q5VZL5 1xTMT6plex [K	0,01	-0,58	0,12
[R],RVSHSPPPKQR,[S]	1xTMT6plex [K9];1xTM	Q8IYB3	Q8IYB3 [634-644]	Q8IYB3 1xTMT6plex [K	0,00	-0,58	0,12
[R],SLPAFPTSSLLTQSQK,[L]	1xTMT6plex [K16];1xTM	Q99698	Q99698 [2105-2120]	Q99698 1xTMT6plex [K	0,03	-0,58	0,13
[R],GNVVPSPLPTR,[R]	1xTMT6plex [N-Term];1	Q9Y2V2	Q9Y2V2 [36-46]	Q9Y2V2 1xTMT6plex [N	0,02	-0,58	0,13
[R],LRDHSSER,[S]	1xTMT6plex [N-Term];1	Q7Z3B3	Q7Z3B3 [788-795]	Q7Z3B3 1xTMT6plex [N	0,01	-0,57	0,12
[R],TSSPSTASTK,[S]	1xTMT6plex [K10];1xTM	P25054	P25054 [2348-2357]	P25054 1xTMT6plex [K	0,04	-0,57	0,15
[K],RNSGLSK,[E]	1xTMT6plex [K7];1xTM	O95251	O95251 [271-277]	O95251 1xTMT6plex [K	0,02	-0,57	0,13
[R],YSHSGSSSPDTK,[V]	1xTMT6plex [K12];1xTM	Q9UQ35	Q9UQ35 [967-978]	Q9UQ35 1xTMT6plex [	0,03	-0,57	0,14
[R],SSRPASASSSTK,[D]	1xTMT6plex [K12];1xTM	Q14686	Q14686 [2038-2049]	Q14686 1xTMT6plex [K	0,00	-0,57	0,12
[R],GSLASLDSLR,[K]	1xTMT6plex [N-Term];1	O60716	O60716 [345-354]	O60716 1xTMT6plex [N	0,04	-0,57	0,15
[R],EDSVKPGAHLTVK,[K]	2xTMT6plex [K5; K13];1	P51991	P51991 [114-126]	P51991 2xTMT6plex [K	0,01	-0,57	0,12
[K],DGHSPMSK,[G]	1xOxidation [M6];1xTM	P78559	P78559 [2661-2668]	P78559 1xOxidation [M	0,03	-0,57	0,14
[R],GTSTGIISK,[I]	1xTMT6plex [K9];1xTM	Q9P0L2	Q9P0L2 [647-655]	Q9P0L2 1xTMT6plex [K	0,04	-0,57	0,15
[R],HAPSLHGSTELLPLSR,[D]	1xTMT6plex [N-Term];1	Q9H5N1	Q9H5N1 [186-201]	Q9H5N1 1xTMT6plex [N	0,01	-0,57	0,12
[R],ITSPLMEPSSIEK,[I]	1xTMT6plex [K13];1xTM	P28066	P28066 [54-66]	P28066 1xTMT6plex [K	0,02	-0,57	0,13
[R],GLVRPGSSR,[E]	1xTMT6plex [N-Term];1	P49750	P49750 [1082-1090]	P49750 1xTMT6plex [N	0,01	-0,57	0,12
[R],VPGSSGHLHK,[T]	1xTMT6plex [K10];1xTM	Q9UEW8	Q9UEW8 [367-376]	Q9UEW8 1xTMT6plex [	0,01	-0,57	0,12

[R],GFSDSGGGPPAK,[Q]	1xTMT6plex [K12];1xTM	Q9H307	Q9H307 [64-75]	Q9H307 1xTMT6plex [K	0,04	-0,57	0,15
[R],NSPASPLK,[E]	1xTMT6plex [K8];1xTM	O43572	O43572 [277-284]	O43572 1xTMT6plex [K	0,04	-0,57	0,16
[R],TNTPQGVLPSSQLK,[S]	1xTMT6plex [K14];1xTM	Q14004	Q14004 [1056-1069]	Q14004 1xTMT6plex [K	0,01	-0,57	0,12
[R],ALMTSHGSVEGR,[S]	1xOxidation [M3];1xTM	Q9H8V3	Q9H8V3 [854-865]	Q9H8V3 1xOxidation [M	0,00	-0,57	0,12
[K],EKEPGEQASVPLSPK,[K]	2xTMT6plex [K2; K15];1	P42858	P42858 [1187-1201]	P42858 2xTMT6plex [K	0,01	-0,57	0,12
[R],SASASHQADIK,[E]	1xTMT6plex [K11];1xTM	Q14739	Q14739 [97-107]	Q14739 1xTMT6plex [K	0,02	-0,57	0,13
[R],SPQQTVPYVVPLSPK,[L]	1xTMT6plex [K15];1xTM	Q9UQR0	Q9UQR0 [499-513]	Q9UQR0 1xTMT6plex [	0,01	-0,57	0,12
[K],ELVGPPLAETVFTPK,[T]	1xTMT6plex [K15];1xTM	P49792	P49792 [1384-1398]	P49792 1xTMT6plex [K	0,03	-0,57	0,14
[R],KSSVTEE,[-]	1xTMT6plex [K1];1xTM	O94874	O94874 [788-794]	O94874 1xTMT6plex [K	0,02	-0,57	0,13
[R],RGSNPVK,[R]	1xTMT6plex [K7];1xTM	Q8N6Y2	Q8N6Y2 [40-46]	Q8N6Y2 1xTMT6plex [K	0,01	-0,57	0,12
[R],KASSSEGSMK,[V]	2xTMT6plex [K1; K10];1	Q9UGU5	Q9UGU5 [478-487]	Q9UGU5 2xTMT6plex [	0,03	-0,57	0,14
[K],WSPPQNYK,[K]	1xTMT6plex [K8];1xTM	Q96K21	Q96K21 [143-150]	Q96K21 1xTMT6plex [K	0,03	-0,57	0,14
[K],DISQNKR,[A]	1xTMT6plex [K6];1xTM	P0DMV8	P0DMV8 [252-258]	P0DMV8 1xTMT6plex [	0,02	-0,56	0,13
[K],STSTSSMK,[K]	1xTMT6plex [K8];1xTM	P27816	P27816 [863-870]	P27816 1xTMT6plex [K	0,03	-0,56	0,13
[R],GYSSLLK,[R]	1xTMT6plex [K7];1xTM	P17844	P17844 [517-523]	P17844 1xTMT6plex [K	0,01	-0,56	0,12
[R],ASPIQPALDPSR,[S]	1xTMT6plex [N-Term];1	Q99500	Q99500 [325-336]	Q99500 1xTMT6plex [N	0,02	-0,56	0,12
[R],ASEDTTSGSPPKK,[S]	2xTMT6plex [K12; K13]	Q9BZZ5	Q9BZZ5 [456-468]	Q9BZZ5 2xTMT6plex [K	0,00	-0,56	0,12
[R],TCSLPDLSKLFR,[T]	1xCarbamidomethyl [C2	Q96PY6	Q96PY6 [1050-1061]	Q96PY6 1xCarbamidom	0,00	-0,56	0,12
[K],IEEVLSPEGSPSKSPSK,[K]	2xPhospho [S10; S12];2	Q9UEY8	Q9UEY8 [668-684]	Q9UEY8 2xPhospho [S	0,00	-0,56	0,12
[R],ISSSSFSR,[V]	1xTMT6plex [N-Term];1	P05787	P05787 [33-40]	P05787 1xTMT6plex [N	0,01	-0,56	0,12
[R],LSPGESAYQK,[L]	1xTMT6plex [K10];1xTM	Q6NYC8	Q6NYC8 [223-232]	Q6NYC8 1xTMT6plex [	0,03	-0,56	0,13
[R],GSVHSLDAGLLLPSGDPFS	1xTMT6plex [K20];1xTM	Q15276	Q15276 [373-392]	Q15276 1xTMT6plex [K	0,05	-0,56	0,17
[R],VPSVAEAPQLRPAGTAAAK	1xTMT6plex [K19];1xTM	Q63ZY3	Q63ZY3 [538-556]	Q63ZY3 1xTMT6plex [K	0,02	-0,56	0,12
[R],RSPRPDHPGTPPHK,[T]	1xTMT6plex [K14];1xTM	P30291	P30291 [164-177]	P30291 1xTMT6plex [K	0,03	-0,56	0,14
[R],RPNSGPAGGSK,[E]	1xTMT6plex [K11];1xTM	P50221	P50221 [104-114]	P50221 1xTMT6plex [K	0,01	-0,56	0,12
[K],LGDFGLSR,[Y]	1xTMT6plex [N-Term];1	Q05397; Q	Q05397 [562-569]; Q14289 [565-572	Q05397 1xTMT6plex [N	0,05	-0,56	0,16
[K],HGSSLNR,[T]	1xTMT6plex [N-Term];1	Q13191	Q13191 [791-797]	Q13191 1xTMT6plex [N	0,02	-0,56	0,12
[R],KDTPHYK,[K]	2xTMT6plex [K1; K7];1x	Q14160	Q14160 [605-611]	Q14160 2xTMT6plex [K	0,00	-0,56	0,12
[R],SCGHQTSASSLK,[A]	1xCarbamidomethyl [C2	Q9HB90	Q9HB90 [376-387]	Q9HB90 1xCarbamidom	0,02	-0,56	0,13
[K],ISAKTLTDEVNSPDSDRR,[D	1xTMT6plex [K4];1xTM	Q8TAQ2	Q8TAQ2 [272-289]	Q8TAQ2 1xTMT6plex [	0,00	-0,56	0,12
[K],QKYLSFTPPEK,[D]	2xTMT6plex [K2; K11];1	Q13177	Q13177 [137-147]	Q13177 2xTMT6plex [K	0,03	-0,56	0,13
[R],ENVFQEHQTLK,[E]	1xTMT6plex [K11];1xTM	Q14247	Q14247 [60-70]	Q14247 1xTMT6plex [K	0,04	-0,56	0,15
[K],KEPAITSQNSPEAR,[E]	1xTMT6plex [K1];1xTM	P23193	P23193 [91-104]	P23193 1xTMT6plex [K	0,01	-0,56	0,12
[R],FRLTPPSPVR,[S]	1xTMT6plex [N-Term];2	Q8TAP8	Q8TAP8 [81-90]	Q8TAP8 1xTMT6plex [N	0,00	-0,56	0,11
[K],KVEFTDSPESR,[K]	1xTMT6plex [K1];1xTM	Q99697	Q99697 [34-44]	Q99697 1xTMT6plex [K	0,02	-0,56	0,13
[R],KPTKPESQSPGK,[R]	3xTMT6plex [K1; K4; K	Q5H9F3	Q5H9F3 [1184-1195]	Q5H9F3 3xTMT6plex [K	0,01	-0,56	0,12
[R],LDFEGPDAKLSGPSLK,[M]	2xTMT6plex [K9; K16];1	Q09666	Q09666 [4890-4905]	Q09666 2xTMT6plex [K	0,01	-0,56	0,12
[R],DSFSGVK,[D]	1xTMT6plex [K7];1xTM	Q14865	Q14865 [263-269]	Q14865 1xTMT6plex [K	0,01	-0,56	0,12
[R],KPIDSLRDSR,[S]	1xTMT6plex [K1];1xTM	Q9UQ35	Q9UQ35 [2680-2689]	Q9UQ35 1xTMT6plex [	0,00	-0,56	0,12
[R],GFEGSCSQK,[E]	1xCarbamidomethyl [C6	O43719	O43719 [475-483]	O43719 1xCarbamidom	0,03	-0,56	0,14
[R],KTSPTNEK,[T]	2xTMT6plex [K1; K8];1x	Q96QE3	Q96QE3 [68-75]	Q96QE3 2xTMT6plex [K	0,01	-0,56	0,12
[R],KGSAVDASVQEESPVTK,[E	2xTMT6plex [K1; K17];1	O95613	O95613 [42-58]	O95613 2xTMT6plex [K	0,03	-0,56	0,14
[R],SGQVLEVSGSK,[A]	1xTMT6plex [K11];1xTM	P21281	P21281 [83-93]	P21281 1xTMT6plex [K	0,05	-0,56	0,16
[K],NKGSGSLDG,[-]	1xTMT6plex [K2];1xTM	Q9NYR9	Q9NYR9 [183-191]	Q9NYR9 1xTMT6plex [	0,05	-0,56	0,16
[K],LDYGQHVVAGTPGR,[V]	1xTMT6plex [N-Term];1	P38919	P38919 [153-166]	P38919 1xTMT6plex [N	0,03	-0,56	0,14
[R],GERDSQTQAILTK,[L]	1xTMT6plex [K13];1xTM	Q13098	Q13098 [236-248]	Q13098 1xTMT6plex [K	0,05	-0,56	0,16
[R],ASGQAFELILSPR,[S]	1xTMT6plex [N-Term];1	P16949	P16949 [15-27]	P16949 1xTMT6plex [N	0,00	-0,56	0,12
[R],SGAGSSPETK,[E]	1xTMT6plex [K10];1xTM	Q9UQ35	Q9UQ35 [1214-1223]	Q9UQ35 1xTMT6plex [	0,02	-0,55	0,13
[R],KPGSHASSSAR,[R]	1xTMT6plex [K1];1xTM	Q9H7N4	Q9H7N4 [561-571]	Q9H7N4 1xTMT6plex [K	0,01	-0,55	0,12
[R],SDSLLSFR,[L]	1xTMT6plex [N-Term];1	Q00587	Q00587 [190-197]	Q00587 1xTMT6plex [N	0,02	-0,55	0,13
[R],ASLPELPR,[M]	1xTMT6plex [N-Term];1	Q8TF74	Q8TF74 [143-150]	Q8TF74 1xTMT6plex [N	0,02	-0,55	0,13
[K],LGNSLLR,[R]	1xTMT6plex [N-Term];1	Q14980	Q14980 [2059-2065]	Q14980 1xTMT6plex [N	0,02	-0,55	0,13
[R],GHSTEIPVGR,[S]	1xTMT6plex [N-Term];1	Q00975	Q00975 [1958-1967]	Q00975 1xTMT6plex [N	0,00	-0,55	0,12
[R],HASSANQYK,[Y]	1xTMT6plex [K9];1xTM	Q8TED9	Q8TED9 [601-609]	Q8TED9 1xTMT6plex [K	0,02	-0,55	0,12
[R],LLSVNIR,[V]	1xTMT6plex [N-Term];1	P07814	P07814 [1334-1340]	P07814 1xTMT6plex [N	0,01	-0,55	0,12
[R],MNSLTFK,[K]	1xOxidation [M1];1xTM	Q96N67	Q96N67 [1381-1387]	Q96N67 1xOxidation [M	0,02	-0,55	0,13
[K],FSPFPVQDRPESSLVFK,[D]	1xTMT6plex [K17];1xTM	Q9UQ35	Q9UQ35 [1187-1203]	Q9UQ35 1xTMT6plex [	0,02	-0,55	0,13

[K],RFSGTVR,[L]	1xTMT6plex [N-Term];1	P62906	P62906 [48-54]	P62906 1xTMT6plex [N	0,02	-0,55	0,12
[R],LEDLDTCMMTPK,[S]	1xCarbamidomethyl [C7	Q5VZK9	Q5VZK9 [907-918]	Q5VZK9 1xCarbamidom	0,02	-0,55	0,12
[R],LEDLDTCMMTPK,[S]	1xCarbamidomethyl [C7	Q5VZK9	Q5VZK9 [907-918]	Q5VZK9 1xCarbamidom	0,02	-0,55	0,12
[K],SATVKPGAVGAGEFVSPCE	1xCarbamidomethyl [C1	Q12888	Q12888 [1686-1719]	Q12888 1xCarbamidom	0,00	-0,55	0,11
[R],ASFSRPTFLQLSPGGLR,[R	1xTMT6plex [N-Term];2	Q5JR12	Q5JR12 [65-81]	Q5JR12 1xTMT6plex [N	0,01	-0,55	0,12
[R],SPSLDNPTPFPNLGPSENP	1xTMT6plex [K21];1xTM	Q14653	Q14653 [173-193]	Q14653 1xTMT6plex [K	0,03	-0,55	0,13
[R],KPSVGSPSLTR,[R]	1xTMT6plex [K1];1xTM	Q9NRJ4	Q9NRJ4 [572-582]	Q9NRJ4 1xTMT6plex [K	0,05	-0,55	0,16
[K],EKVSPGR,[T]	1xTMT6plex [K2];1xTM	P42858	P42858 [2931-2937]	P42858 1xTMT6plex [K	0,01	-0,55	0,12
[R],VSVSPGR,[T]	1xTMT6plex [N-Term];1	Q8IYB3	Q8IYB3 [428-434]	Q8IYB3 1xTMT6plex [N	0,01	-0,55	0,12
[K],HSQSMIEDAQLPLEQK,[K]	1xTMT6plex [K16];1xTM	Q13576	Q13576 [1355-1370]	Q13576 1xTMT6plex [K	0,02	-0,55	0,13
[R],FSISFAR,[H]	1xTMT6plex [N-Term];1	O43314	O43314 [1107-1113]	O43314 1xTMT6plex [N	0,02	-0,55	0,13
[R],KSSTSGSDFDTK,[K]	2xTMT6plex [K1; K12];1	P46100	P46100 [782-793]	P46100 2xTMT6plex [K	0,00	-0,55	0,11
[R],SYSPDGKESPSDK,[K]	2xTMT6plex [K7; K13];1	P43243	P43243 [596-608]	P43243 2xTMT6plex [K	0,00	-0,55	0,11
[K],SKPNLPSESR,[S]	1xTMT6plex [K2];1xTM	Q08170	Q08170 [450-459]	Q08170 1xTMT6plex [K	0,02	-0,55	0,13
[R],SSPPGHYYQK,[S]	1xTMT6plex [K10];1xTM	Q9NX40	Q9NX40 [122-131]	Q9NX40 1xTMT6plex [K	0,02	-0,55	0,13
[K],AAVTPGK,[K]	1xTMT6plex [K7];1xTM	P19338	P19338 [81-87]	P19338 1xTMT6plex [K	0,05	-0,55	0,16
[R],ETVSPPCFDEYK,[M]	1xCarbamidomethyl [C7	Q13029	Q13029 [793-804]	Q13029 1xCarbamidom	0,02	-0,55	0,13
[R],SSFSQHAR,[T]	1xTMT6plex [N-Term];1	P02545	P02545 [428-435]	P02545 1xTMT6plex [N	0,04	-0,55	0,15
[R],SPSTSPK,[F]	1xTMT6plex [K7];1xTM	Q96SK2	Q96SK2 [147-153]	Q96SK2 1xTMT6plex [K	0,00	-0,55	0,12
[K],LPISSSTSNLHVDR,[E]	1xTMT6plex [N-Term];1	Q86TV6	Q86TV6 [155-168]	Q86TV6 1xTMT6plex [N	0,03	-0,55	0,14
[K],LPISSSTSNLHVDR,[E]	1xTMT6plex [N-Term];1	Q86TV6	Q86TV6 [155-168]	Q86TV6 1xTMT6plex [N	0,03	-0,55	0,14
[K],ALKQNGEKSTK,[T]	1xMethyl [K8];2xTMT6p	Q96IX9	Q96IX9 [67-77]	Q96IX9 1xMethyl [K74];	0,04	-0,55	0,15
[R],SSPSGNTQSSPK,[S]	1xTMT6plex [K12];1xTM	O00512	O00512 [10-21]	O00512 1xTMT6plex [K	0,03	-0,55	0,13
[R],GSDRTPER,[S]	1xTMT6plex [N-Term];1	Q8NDT2	Q8NDT2 [627-634]	Q8NDT2 1xTMT6plex [N	0,05	-0,55	0,16
[K],ELKTDSSPNQAR,[A]	1xTMT6plex [K3];1xTM	P26599	P26599 [135-146]	P26599 1xTMT6plex [K	0,01	-0,55	0,12
[R],KDSPHSR,[S]	1xTMT6plex [K1];1xTM	Q8NEY8	Q8NEY8 [138-144]	Q8NEY8 1xTMT6plex [K	0,04	-0,54	0,15
[R],TPSDKSAHTVEHGSPR,[T]	1xTMT6plex [K5];1xTM	Q96GP6	Q96GP6 [705-720]	Q96GP6 1xTMT6plex [K	0,00	-0,54	0,11
[K],ELVSRELK,[D]	1xTMT6plex [K8];1xTM	Q5VYS8	Q5VYS8 [579-586]	Q5VYS8 1xTMT6plex [K	0,00	-0,54	0,11
[K],NKGSGSLDG,[-]	1xTMT6plex [K2];1xTM	Q9NYR9	Q9NYR9 [183-191]	Q9NYR9 1xTMT6plex [K	0,03	-0,54	0,13
[R],IQTSLTSASLGSADENSVAC	1xTMT6plex [K26];1xTM	P49815	P49815 [991-1016]	P49815 1xTMT6plex [K	0,03	-0,54	0,14
[K],KKSEAPSGESR,[K]	2xTMT6plex [K1; K2];1x	B5ME19	B5ME19 [199-209]	B5ME19 2xTMT6plex [K	0,02	-0,54	0,13
[R],LSESQLSFR,[R]	1xTMT6plex [N-Term];1	Q96PK6	Q96PK6 [617-625]	Q96PK6 1xTMT6plex [N	0,02	-0,54	0,13
[R],SGGDTHSPPR,[G]	1xTMT6plex [N-Term];1	O43295	O43295 [868-877]	O43295 1xTMT6plex [N	0,02	-0,54	0,13
[R],MPQPSSGRESPR,[H]	1xTMT6plex [N-Term];1	P56181	P56181 [96-107]	P56181 1xTMT6plex [N	0,04	-0,54	0,15
[K],KPAGATPK,[K]	2xTMT6plex [K1; K8];1x	P16401	P16401 [133-140]	P16401 2xTMT6plex [K	0,04	-0,54	0,15
[R],KENPSPLFSIK,[K]	2xTMT6plex [K1; K11];1	Q9P1Y6	Q9P1Y6 [810-820]	Q9P1Y6 2xTMT6plex [K	0,03	-0,54	0,14
[K],VAGAATPK,[K]	1xPhospho [T6];1xTMT	P16402	P16402 [142-149]	P16402 1xPhospho [T14	0,02	-0,54	0,13
[K],SFDAVLEALSR,[G]	1xTMT6plex [N-Term];1	Q6P1N0	Q6P1N0 [292-302]	Q6P1N0 1xTMT6plex [N	0,02	-0,54	0,12
[R],ESASSSSSVKK,[A]	2xTMT6plex [K10; K11]	Q9NPF5	Q9NPF5 [453-463]	Q9NPF5 2xTMT6plex [K	0,00	-0,54	0,12
[K],LSPQMQVHQDK,[T]	1xTMT6plex [K11];1xTM	Q12830	Q12830 [2369-2379]	Q12830 1xTMT6plex [K	0,01	-0,54	0,12
[R],EKTPSEDK,[L]	2xTMT6plex [K2; K8];1x	Q8NFC6	Q8NFC6 [731-738]	Q8NFC6 2xTMT6plex [K	0,05	-0,54	0,16
[K],IPLFNTDVDNLEGKTPPVFA	2xTMT6plex [K14; K24]	Q96PZ0	Q96PZ0 [596-619]	Q96PZ0 2xTMT6plex [K	0,04	-0,54	0,15
[R],FEVADEDKQSR,[L]	1xTMT6plex [K8];1xTM	Q9H6X4	Q9H6X4 [44-54]	Q9H6X4 1xTMT6plex [K	0,01	-0,54	0,12
[R],TPTAPAVNLAGAR,[T]	1xTMT6plex [N-Term];1	Q9UQ35	Q9UQ35 [2289-2301]	Q9UQ35 1xTMT6plex [N	0,04	-0,54	0,15
[R],SSPATHSP,[-]	1xTMT6plex [N-Term];1	P10276	P10276 [455-462]	P10276 1xTMT6plex [N	0,02	-0,54	0,13
[R],SPSLDNPTPFPNLGPSENP	1xTMT6plex [K21];1xTM	Q14653	Q14653 [173-193]	Q14653 1xTMT6plex [K	0,02	-0,54	0,12
[R],KASPEPPDSAEGALK,[L]	2xTMT6plex [K1; K15];1	Q9H1B7	Q9H1B7 [545-559]	Q9H1B7 2xTMT6plex [K	0,01	-0,54	0,12
[R],TLSLRNSISR,[I]	1xTMT6plex [N-Term];2	Q96FS4	Q96FS4 [906-915]	Q96FS4 1xTMT6plex [N	0,03	-0,54	0,14
[R],SATLPLPAR,[S]	1xTMT6plex [N-Term];1	Q9BX66	Q9BX66 [495-503]	Q9BX66 1xTMT6plex [N	0,01	-0,54	0,12
[K],SLSDSESDDSKSK,[K]	2xTMT6plex [K11; K13]	Q13185	Q13185 [93-105]	Q13185 2xTMT6plex [K	0,00	-0,54	0,10
[K],SSTPPRQSPSR,[S]	1xTMT6plex [N-Term];2	Q9UQ35	Q9UQ35 [901-911]	Q9UQ35 1xTMT6plex [N	0,01	-0,54	0,12
[K],KASPEAASTPR,[D]	1xTMT6plex [K1];1xTM	P29590	P29590 [401-411]	P29590 1xTMT6plex [K	0,02	-0,54	0,13
[R],RPGFSPTSHR,[L]	1xTMT6plex [N-Term];1	Q8WUA4	Q8WUA4 [897-906]	Q8WUA4 1xTMT6plex [	0,01	-0,54	0,12
[R],GDFHSPIVLGRPPNTEDRE	1xTMT6plex [K24];1xTM	Q02078	Q02078 [475-498]	Q02078 1xTMT6plex [K	0,02	-0,54	0,12
[R],SRSPLAIR,[R]	1xTMT6plex [N-Term];1	Q9UQ35	Q9UQ35 [2044-2051]	Q9UQ35 1xTMT6plex [N	0,05	-0,54	0,17
[R],YFQSPSR,[S]	1xTMT6plex [N-Term];1	Q16629	Q16629 [189-195]	Q16629 1xTMT6plex [N	0,02	-0,54	0,13
[R],ESGSPYSVLVDTK,[M]	1xTMT6plex [K13];1xTM	Q9Y2D9	Q9Y2D9 [54-66]	Q9Y2D9 1xTMT6plex [K	0,00	-0,54	0,12

[R],KLSHDAESEREDPAPAK,[K	2xTMT6plex [K1; K17];1	Q92766	Q92766 [173-189]	Q92766 2xTMT6plex [K	0,00	-0,54	0,11
[R],SKSEDMDNVQSK,[R]	1xOxidation [M6];2xTMT	Q6VMQ6	Q6VMQ6 [557-568]	Q6VMQ6 1xOxidation [I	0,01	-0,54	0,12
[K],DGWNQNHFITPVSTLER,[D	1xTMT6plex [N-Term];1	Q99569	Q99569 [1004-1020]	Q99569 1xTMT6plex [N	0,00	-0,54	0,11
[R],KDDDSDDESQSSHTGKK,[I	3xTMT6plex [K1; K16; I	O94874	O94874 [454-470]	O94874 3xTMT6plex [K	0,01	-0,54	0,12
[R],VESGYFSLEK,[T]	1xTMT6plex [K10];1xTN	Q6WCQ1	Q6WCQ1 [263-272]	Q6WCQ1 1xTMT6plex	0,03	-0,54	0,14
[R],TPTSTPVHVK,[Q]	1xTMT6plex [K10];1xTN	Q9ULM3	Q9ULM3 [478-487]	Q9ULM3 1xTMT6plex [I	0,01	-0,54	0,12
[K],KTESASVQGR,[N]	1xTMT6plex [K1];1xTM	Q9P2E9	Q9P2E9 [599-608]	Q9P2E9 1xTMT6plex [K	0,02	-0,54	0,12
[R],KVSSAEGAAK,[E]	2xTMT6plex [K1; K10];1	P05114	P05114 [5-14]	P05114 2xTMT6plex [K	0,03	-0,53	0,14
[R],STPNLSLDDVK,[V]	1xTMT6plex [K11];1xTN	Q15788	Q15788 [762-772]	Q15788 1xTMT6plex [K	0,04	-0,53	0,15
[K],ELKTDSSPNQAR,[A]	1xTMT6plex [K3];1xTM	P26599	P26599 [135-146]	P26599 1xTMT6plex [K	0,00	-0,53	0,11
[K],FATLSLHDR,[K]	1xTMT6plex [N-Term];1	Q8TBZ3	Q8TBZ3 [461-469]	Q8TBZ3 1xTMT6plex [N	0,02	-0,53	0,13
[K],LDSPDPFKLNDPFQPFPGN	2xTMT6plex [K8; K23];1	P42566	P42566 [794-816]	P42566 2xTMT6plex [K	0,01	-0,53	0,12
[R],GLNSSFETSPKK,[V]	2xTMT6plex [K11; K12]	Q8IV63	Q8IV63 [51-62]	Q8IV63 2xTMT6plex [K	0,00	-0,53	0,12
[R],KRDSDAGSSTPTTSTR,[S]	1xTMT6plex [K1];1xTM	P51532	P51532 [1414-1429]	P51532 1xTMT6plex [K	0,01	-0,53	0,12
[R],KTSPGPTHR,[G]	1xTMT6plex [K1];1xTM	Q14678	Q14678 [1340-1348]	Q14678 1xTMT6plex [K	0,03	-0,53	0,13
[K],SISSPSVSSETMDKPVDLST	1xTMT6plex [K14];1xTN	P49792	P49792 [2802-2822]	P49792 1xTMT6plex [K	0,01	-0,53	0,12
[R],VFEEATSPGPDLAFLTSCPI	1xCarbamidomethyl [C1	Q9C073	Q9C073 [349-369]	Q9C073 1xCarbamidom	0,01	-0,53	0,12
[R],SLDSLDPAGLLTSPK,[F]	1xTMT6plex [K15];1xTN	O14490	O14490 [419-433]	O14490 1xTMT6plex [K	0,05	-0,53	0,16
[R],DKSPSSLLEDAK,[E]	2xTMT6plex [K2; K12];1	P42684	P42684 [629-640]	P42684 2xTMT6plex [K	0,01	-0,53	0,12
[K],SLSSSLDDTEVKK,[V]	2xTMT6plex [K12; K13]	O95292	O95292 [156-168]	O95292 2xTMT6plex [K	0,01	-0,53	0,12
[K],VATPGPASK,[C]	1xTMT6plex [K9];1xTM	C9J069	C9J069 [20-28]	C9J069 1xTMT6plex [K	0,04	-0,53	0,15
[R],GAVDGILHMFTPK,[L]	1xOxidation [M9];1xTMT	Q9H1V8	Q9H1V8 [276-288]	Q9H1V8 1xOxidation [M	0,02	-0,53	0,13
[R],TVPSTPTLVVPHR,[T]	1xTMT6plex [N-Term];1	P12270	P12270 [2133-2145]	P12270 1xTMT6plex [N	0,02	-0,53	0,12
[R],NPASPSK,[S]	1xTMT6plex [K7];1xTM	Q8NDB6	Q8NDB6 [8-14]	Q8NDB6 1xTMT6plex [I	0,03	-0,53	0,14
[R],ESVDSRDSSHSR,[E]	1xTMT6plex [N-Term];1	Q9Y2W1	Q9Y2W1 [739-750]	Q9Y2W1 1xTMT6plex [	0,04	-0,53	0,15
[R],RNSCNVGGGGGGFK,[H]	1xCarbamidomethyl [C4	Q7L2J0	Q7L2J0 [150-163]	Q7L2J0 1xCarbamidom	0,02	-0,53	0,13
[R],TNSMGSATGPLPGTK,[V]	1xOxidation [M4];1xTMT	O15014	O15014 [465-479]	O15014 1xOxidation [M	0,04	-0,53	0,15
[R],RDSTEAPKPK,[S]	2xTMT6plex [K8; K10];1	Q8N201	Q8N201 [1316-1325]	Q8N201 2xTMT6plex [K	0,04	-0,53	0,15
[K],VTKAGGSAALSPSK,[K]	2xTMT6plex [K3; K14];1	Q92522	Q92522 [21-34]	Q92522 2xTMT6plex [K	0,00	-0,53	0,11
[R],FPQSPHSGR,[H]	1xTMT6plex [N-Term];1	Q13796	Q13796 [419-427]	Q13796 1xTMT6plex [N	0,04	-0,53	0,16
[K],KAEGSPSEGK,[K]	2xTMT6plex [K1; K10];1	Q9P2E9	Q9P2E9 [569-578]	Q9P2E9 2xTMT6plex [K	0,02	-0,53	0,12
[R],NSPRPSPK,[Q]	1xTMT6plex [K8];1xTM	Q9P270	Q9P270 [348-355]	Q9P270 1xTMT6plex [K	0,01	-0,53	0,12
[R],SAFLSQR,[Y]	1xTMT6plex [N-Term];1	P35658	P35658 [970-976]	P35658 1xTMT6plex [N	0,00	-0,53	0,11
[R],STLGDLDTVAGLEK,[E]	1xTMT6plex [K14];1xTN	Q5VU43	Q5VU43 [740-753]	Q5VU43 1xTMT6plex [K	0,00	-0,53	0,11
[R],LPSANQGSPFK,[S]	1xTMT6plex [K11];1xTN	Q56NI9	Q56NI9 [68-78]	Q56NI9 1xTMT6plex [K	0,03	-0,53	0,14
[K],RSFSKEVEER,[S]	1xTMT6plex [K5];1xTM	Q04637	Q04637 [1184-1193]	Q04637 1xTMT6plex [K	0,01	-0,53	0,12
[R],GSQNSSEHRPPASSTSED\	1xTMT6plex [K20];1xTN	O15014	O15014 [412-431]	O15014 1xTMT6plex [K	0,03	-0,53	0,13
[K],KAPSASDSDSK,[A]	2xTMT6plex [K1; K11];1	Q7Z4V5	Q7Z4V5 [227-237]	Q7Z4V5 2xTMT6plex [K	0,03	-0,53	0,13
[R],VGKPSPK,[A]	2xTMT6plex [K3; K7];1x	Q7Z5J4	Q7Z5J4 [1098-1104]	Q7Z5J4 2xTMT6plex [K	0,01	-0,53	0,12
[R],VHGSLGDTPR,[S]	1xTMT6plex [N-Term];1	Q96KQ7	Q96KQ7 [37-46]	Q96KQ7 1xTMT6plex [N	0,00	-0,53	0,12
[R],KSRDSESHSR,[V]	1xTMT6plex [K1];1xTM	Q15424	Q15424 [613-622]	Q15424 1xTMT6plex [K	0,03	-0,53	0,13
[K],DKVSPLQNLASINNK,[K]	2xTMT6plex [K2; K15];1	Q03112	Q03112 [621-635]	Q03112 2xTMT6plex [K	0,01	-0,53	0,12
[R],QSHSSSSPHPK,[V]	1xTMT6plex [K11];1xTN	Q9UQ35	Q9UQ35 [833-843]	Q9UQ35 1xTMT6plex [I	0,03	-0,53	0,14
[R],SPALHGTPTAGCGSR,[G]	1xCarbamidomethyl [C1	Q8IY92	Q8IY92 [584-598]	Q8IY92 1xCarbamidom	0,02	-0,53	0,13
[R],LLDHMAPPPVADQASPR,[A	1xOxidation [M5];1xTMT	Q9C004	Q9C004 [111-127]	Q9C004 1xOxidation [M	0,01	-0,52	0,12
[R],LQLSVTEVGTEK,[L]	1xTMT6plex [K12];1xTN	P55196	P55196 [421-432]	P55196 1xTMT6plex [K	0,04	-0,52	0,15
[K],KDDSHSAEDSEDEKEDHK,	3xTMT6plex [K1; K14; I	Q9H1E3	Q9H1E3 [70-87]	Q9H1E3 3xTMT6plex [K	0,02	-0,52	0,13
[R],TSSPHKEESPK,[K]	2xTMT6plex [K6; K11];1	P51003	P51003 [652-662]	P51003 2xTMT6plex [K	0,00	-0,52	0,11
[R],RSPTKSSLDYR,[R]	1xTMT6plex [K5];1xTM	Q96PK6	Q96PK6 [626-636]	Q96PK6 1xTMT6plex [K	0,02	-0,52	0,13
[RK],TSCSPQK,[AT]	1xCarbamidomethyl [C3	Q8IY33; Q	Q8IY33 [269-275]; Q8IV63 [126-132]	Q8IY33 1xCarbamidom	0,03	-0,52	0,14
[K],IISPGSSTPSSTR,[S]	1xTMT6plex [N-Term];1	Q5VT52	Q5VT52 [756-768]	Q5VT52 1xTMT6plex [N	0,04	-0,52	0,15
[K],TAAKGEAAAERPGEAAVAS	2xTMT6plex [K4; K23];1	P29966	P29966 [8-30]	P29966 2xTMT6plex [K	0,04	-0,52	0,15
[R],RVSQDLEVEKPDASPTSLQ	1xTMT6plex [K10];1xTN	O94885	O94885 [88-108]	O94885 1xTMT6plex [K	0,01	-0,52	0,12
[R],EFDDPGQQVPASPQGPAV	1xTMT6plex [K21];1xTN	Q8NF99	Q8NF99 [131-151]	Q8NF99 1xTMT6plex [K	0,03	-0,52	0,14
[R],KGSDSSIPDEEK,[M]	2xTMT6plex [K1; K12];1	Q15652	Q15652 [315-326]	Q15652 2xTMT6plex [K	0,02	-0,52	0,13
[K],ESPGSSEFFQEAVSHGK,[F	1xTMT6plex [K17];1xTN	Q8N108	Q8N108 [487-503]	Q8N108 1xTMT6plex [K	0,04	-0,52	0,15
[R],GSIDREDGSLQGPIGNQHI	2xTMT6plex [K25; K33]	Q05397	Q05397 [842-874]	Q05397 2xTMT6plex [K	0,01	-0,52	0,12

[R],SFGTRPLSSGFSPEEAQQ(	2xTMT6plex [K25; K26]	Q9C0C2	Q9C0C2 [976-1001]	Q9C0C2 2xTMT6plex [I	0,01	-0,52	0,12
[R],SSPSARPPDVPGQQPQAA	1xTMT6plex [K19];1xTN	Q96JP5	Q96JP5 [82-100]	Q96JP5 1xTMT6plex [K	0,02	-0,52	0,13
[R],SSPGHAPAR,[A]	1xTMT6plex [N-Term];1	Q10586	Q10586 [164-172]	Q10586 1xTMT6plex [N	0,05	-0,52	0,16
[R],LPALGEAHVSPEVATADK,[,	1xTMT6plex [K18];1xTN	Q9P1Y6	Q9P1Y6 [1220-1237]	Q9P1Y6 1xTMT6plex [K	0,03	-0,52	0,14
[R],AKSPTPESSTIASYVTLR,[K	1xTMT6plex [K2];1xTM	Q9HAU0	Q9HAU0 [853-870]	Q9HAU0 1xTMT6plex [I	0,00	-0,52	0,12
[R],VNLEESSGVENSPAGARPK	1xTMT6plex [K19];1xTN	Q8WVM8	Q8WVM8 [292-310]	Q8WVM8 1xTMT6plex	0,04	-0,52	0,15
[R],SSLFSPR,[R]	1xTMT6plex [N-Term];1	Q7RTP6	Q7RTP6 [1700-1706]	Q7RTP6 1xTMT6plex [N	0,02	-0,52	0,12
[R],GLLSNHTGSPR,[S]	1xTMT6plex [N-Term];1	Q13111	Q13111 [764-774]	Q13111 1xTMT6plex [N	0,02	-0,52	0,13
[R],SGKSPSPSPTSPGSLR,[K]	1xTMT6plex [K3];1xTM	O15075	O15075 [327-342]	O15075 1xTMT6plex [K	0,01	-0,52	0,12
[R],FIQELSGSSPK,[R]	1xTMT6plex [K11];1xTN	Q01664	Q01664 [116-126]	Q01664 1xTMT6plex [K	0,02	-0,52	0,13
[R],TNTPQGVLPSSQLK,[S]	1xTMT6plex [K14];1xTN	Q14004	Q14004 [1056-1069]	Q14004 1xTMT6plex [K	0,01	-0,52	0,12
[R],DLVPRPDSPAQQLK,[S]	1xTMT6plex [K14];1xTN	P78414	P78414 [440-453]	P78414 1xTMT6plex [K	0,01	-0,51	0,12
[K],FSPGAPGGSGSQPNQK,[L]	1xTMT6plex [K16];1xTN	Q15942	Q15942 [280-295]	Q15942 1xTMT6plex [K	0,04	-0,51	0,15
[R],TPSPPPFR,[G]	1xTMT6plex [N-Term];1	Q7L4I2	Q7L4I2 [220-227]	Q7L4I2 1xTMT6plex [N-	0,04	-0,51	0,15
[R],NSSSSTSPVSK,[K]	1xTMT6plex [K11];1xTN	P25054	P25054 [2238-2248]	P25054 1xTMT6plex [K	0,01	-0,51	0,12
[R],AAPTTPPPPVK,[R]	1xTMT6plex [K11];1xTN	Q9NZQ3	Q9NZQ3 [177-187]	Q9NZQ3 1xTMT6plex [I	0,01	-0,51	0,12
[K],TSPTTQSK,[H]	1xTMT6plex [K8];1xTM	Q5UIP0	Q5UIP0 [2242-2249]	Q5UIP0 1xTMT6plex [K	0,03	-0,51	0,14
[R],GSLASLDSLR,[K]	1xTMT6plex [N-Term];1	O60716	O60716 [345-354]	O60716 1xTMT6plex [N	0,04	-0,51	0,15
[R],KGDSEAEALSEIK,[D]	2xTMT6plex [K1; K13];1	Q5T1M5	Q5T1M5 [1009-1021]	Q5T1M5 2xTMT6plex [I	0,01	-0,51	0,12
[K],SEHSLFQAKGSPAGGAK,[T	2xTMT6plex [K9; K17];1	Q8NEY1	Q8NEY1 [142-158]	Q8NEY1 2xTMT6plex [I	0,00	-0,51	0,11
[K],NEEDEGHSNSSPR,[H]	1xTMT6plex [N-Term];1	Q14103	Q14103 [73-85]	Q14103 1xTMT6plex [N	0,05	-0,51	0,16
[R],GASGSIFSK,[F]	1xTMT6plex [K9];1xTM	Q7KZI7	Q7KZI7 [629-637]	Q7KZI7 1xTMT6plex [K	0,01	-0,51	0,12
[R],RPSSVLR,[R]	1xTMT6plex [N-Term];1	Q96N67	Q96N67 [494-500]	Q96N67 1xTMT6plex [N	0,01	-0,51	0,12
[K],SSILLDVKPWDDETDMAK,[	2xTMT6plex [K8; K18];1	P24534	P24534 [140-157]	P24534 2xTMT6plex [K	0,03	-0,51	0,13
[K],KSYSSTLR,[G]	1xTMT6plex [K1];1xTM	Q9NXD2	Q9NXD2 [578-585]	Q9NXD2 1xTMT6plex [I	0,03	-0,51	0,14
[K],RPTPTFHSSR,[T]	1xTMT6plex [N-Term];1	Q9UPN3	Q9UPN3 [7271-7280]	Q9UPN3 1xTMT6plex [I	0,01	-0,51	0,12
[R],TIQFVDWCPTGFK,[V]	1xCarbamidomethyl [C8	Q71U36	Q71U36 [340-352]	Q71U36 1xCarbamidom	0,02	-0,51	0,13
[R],KTLDELSQGTTTVK,[E]	2xTMT6plex [K1; K14];1	P58107	P58107 [1542-1555]	P58107 2xTMT6plex [K	0,01	-0,51	0,12
[R],SQSFNNQAFHAK,[Y]	1xTMT6plex [K12];1xTN	Q6NUP7	Q6NUP7 [773-784]	Q6NUP7 1xTMT6plex [I	0,02	-0,51	0,13
[R],TAITTPNFK,[K]	1xTMT6plex [K9];1xTM	Q9BXS6	Q9BXS6 [178-186]	Q9BXS6 1xTMT6plex [I	0,03	-0,51	0,14
[R],EVSSRPSTPGLSVVSGISAT	1xTMT6plex [K27];1xTN	Q14C86	Q14C86 [755-781]	Q14C86 1xTMT6plex [K	0,05	-0,51	0,16
[R],QSHSGSISPYPK,[V]	1xTMT6plex [K12];1xTN	Q9UQ35	Q9UQ35 [987-998]	Q9UQ35 1xTMT6plex [I	0,02	-0,51	0,13
[K],EVSNFTMGSPLR,[G]	1xTMT6plex [N-Term];1	Q9BYV9	Q9BYV9 [401-412]	Q9BYV9 1xTMT6plex [N	0,01	-0,51	0,12
[R],TFSEPGDHPGMLTSGK,[R]	1xOxidation [M11];1xTN	Q9HA47	Q9HA47 [251-266]	Q9HA47 1xOxidation [M	0,01	-0,51	0,12
[K],YIASTQRPDGTWR,[K]	1xTMT6plex [N-Term];1	Q9BRP8	Q9BRP8 [15-27]	Q9BRP8 1xTMT6plex [I	0,01	-0,51	0,12
[K],TPTSSPASSPLVAK,[K]	1xTMT6plex [K14];1xTN	Q14684	Q14684 [728-741]	Q14684 1xTMT6plex [K	0,02	-0,51	0,13
[K],SPLAGNKDNK,[F]	2xTMT6plex [K7; K10];1	O75151	O75151 [625-634]	O75151 2xTMT6plex [K	0,02	-0,51	0,12
[R],KDPFSPDK,[K]	2xTMT6plex [K1; K8];1x	Q5PSV4	Q5PSV4 [193-200]	Q5PSV4 2xTMT6plex [I	0,02	-0,51	0,12
[R],GPSRDSLPLPVR,[S]	1xTMT6plex [N-Term];1	Q86YR5	Q86YR5 [440-451]	Q86YR5 1xTMT6plex [N	0,04	-0,51	0,15
[R],KGSVDQYLLR,[S]	1xTMT6plex [K1];1xTM	Q8NCN4	Q8NCN4 [691-700]	Q8NCN4 1xTMT6plex [	0,04	-0,51	0,15
[K],SALSPSKSPAK,[L]	2xTMT6plex [K7; K11];1	P49792	P49792 [2273-2283]	P49792 2xTMT6plex [K	0,02	-0,51	0,13
[R],IGVTLAGHQK,[K]	1xTMT6plex [K10];1xTN	P54760; F	P54760 [950-959]; P54753 [968-977]	P54760 1xTMT6plex [K	0,02	-0,51	0,13
[R],ESASPTIPNLDLLEAHTK,[E	1xTMT6plex [K18];1xTN	O75151	O75151 [536-553]	O75151 1xTMT6plex [K	0,00	-0,51	0,11
[R],FDFGEDSEHSEEPKK,[E]	2xTMT6plex [K14; K15]	Q9UPN6	Q9UPN6 [267-281]	Q9UPN6 2xTMT6plex [I	0,05	-0,51	0,16
[R],SLHSAHSLASR,[R]	1xTMT6plex [N-Term];1	O15027	O15027 [1356-1366]	O15027 1xTMT6plex [N	0,01	-0,51	0,12
[K],YFASIHPASTK,[I]	1xTMT6plex [K11];1xTN	Q8TF05	Q8TF05 [927-937]	Q8TF05 1xTMT6plex [K	0,03	-0,51	0,14
[K],IQRSEAK,[T]	1xTMT6plex [K7];1xTM	Q9H2G2	Q9H2G2 [1074-1080]	Q9H2G2 1xTMT6plex [I	0,04	-0,51	0,15
[R],ASVGSLPGLR,[I]	1xTMT6plex [N-Term];1	Q86YR5	Q86YR5 [568-577]	Q86YR5 1xTMT6plex [N	0,03	-0,50	0,13
[R],KPSVPDSASPADDSFVDPC	1xTMT6plex [K1];1xTM	P16333	P16333 [83-103]	P16333 1xTMT6plex [K	0,02	-0,50	0,13
[K],VKQDSESPK,[S]	2xTMT6plex [K2; K9];1x	Q2TB10	Q2TB10 [451-459]	Q2TB10 2xTMT6plex [I	0,00	-0,50	0,11
[R],KSSPSSPAVR,[D]	1xTMT6plex [K1];1xTM	Q8WVR3	Q8WVR3 [493-502]	Q8WVR3 1xTMT6plex [	0,01	-0,50	0,12
[R],SVPTAYRVSR,[S]	1xTMT6plex [N-Term];1	Q12872	Q12872 [824-833]	Q12872 1xTMT6plex [N	0,02	-0,50	0,13
[R],ISAPNVDFNLEGPKVK,[G]	2xTMT6plex [K14; K16]	Q09666	Q09666 [5447-5462]	Q09666 2xTMT6plex [K	0,00	-0,50	0,12
[K],LSPQMQVHQDK,[T]	1xOxidation [M5];1xTM	Q12830	Q12830 [2369-2379]	Q12830 1xOxidation [M	0,01	-0,50	0,12
[R],LTPPPADLPLALPAHGLWS	1xTMT6plex [N-Term];1	Q15742	Q15742 [433-452]	Q15742 1xTMT6plex [N	0,03	-0,50	0,14
[K],EVSNFTMGSPLR,[G]	1xTMT6plex [N-Term];1	Q9BYV9	Q9BYV9 [401-412]	Q9BYV9 1xTMT6plex [N	0,01	-0,50	0,12
[R],KGSPCDTLASSTEKR,[R]	1xCarbamidomethyl [C5	Q15788	Q15788 [20-34]	Q15788 1xCarbamidom	0,02	-0,50	0,13

[R],KSAPSTGGVK,[K]	2xTMT6plex [K1; K10];1	P84243	P84243 [28-37]	P84243 2xTMT6plex [K	0,02	-0,50	0,13
[R],NFTKPQDGDVIAPLITPQKK	3xTMT6plex [K4; K19; K	Q9Y3Z3	Q9Y3Z3 [577-596]	Q9Y3Z3 3xTMT6plex [K	0,01	-0,50	0,12
[R],ASSEDTLNKPGSTAASGVV	1xTMT6plex [K9];1xTM	Q5M775	Q5M775 [53-72]	Q5M775 1xTMT6plex [K	0,03	-0,50	0,13
[R],LSSGDPSTSPSLSQTTPSK	1xTMT6plex [K19];1xTM	Q99504	Q99504 [254-272]	Q99504 1xTMT6plex [K	0,00	-0,50	0,11
[R],RLPQLPSER,[A]	1xTMT6plex [N-Term];1	Q9Y4F5	Q9Y4F5 [700-708]	Q9Y4F5 1xTMT6plex [N	0,03	-0,50	0,13
[R],SGSPLEK,[D]	1xTMT6plex [K7];1xTM	Q9NRA8	Q9NRA8 [136-142]	Q9NRA8 1xTMT6plex [	0,05	-0,50	0,16
[R],VKSPSPK,[S]	2xTMT6plex [K2; K7];1x	O15042	O15042 [944-950]	O15042 2xTMT6plex [K	0,03	-0,50	0,14
[R],TFSEPGDHPGMLTSGK,[R]	1xTMT6plex [K16];1xTM	Q9HA47	Q9HA47 [251-266]	Q9HA47 1xTMT6plex [K	0,01	-0,50	0,12
[R],FSEGVLQSPSQDQEK,[L]	1xTMT6plex [K15];1xTM	Q9C0C2	Q9C0C2 [428-442]	Q9C0C2 1xTMT6plex [K	0,02	-0,50	0,13
[R],TPSDGALKPETLLASR,[S]	1xTMT6plex [K8];1xTM	P80192	P80192 [915-930]	P80192 1xTMT6plex [K	0,01	-0,50	0,12
[K],SKPNLPSESR,[S]	1xTMT6plex [K2];1xTM	Q08170	Q08170 [450-459]	Q08170 1xTMT6plex [K	0,02	-0,50	0,13
[K],VSPSKSPSLSPSPPSPLEK,	2xTMT6plex [K5; K19];1	P46821	P46821 [1251-1269]	P46821 2xTMT6plex [K	0,00	-0,50	0,11
[K],VKAIISPR,[Q]	1xTMT6plex [K2];1xTM	Q9UQ35	Q9UQ35 [919-926]	Q9UQ35 1xTMT6plex [	0,01	-0,50	0,12
[R],AAPACSPK,[E]	1xCarbamidomethyl [C5	Q96BV0	Q96BV0 [526-533]	Q96BV0 1xCarbamidom	0,03	-0,50	0,14
[R],KSAPATGGVK,[K]	2xTMT6plex [K1; K10];1	Q5TEC6;	Q5TEC6 [28-37]; Q16695 [28-37]	Q5TEC6 2xTMT6plex [K	0,03	-0,50	0,14
[R],DLSPTLIDNSAAK,[Q]	1xTMT6plex [K13];1xTM	Q9P2B4	Q9P2B4 [486-498]	Q9P2B4 1xTMT6plex [K	0,00	-0,50	0,12
[R],MAPPVDDLSPK,[K]	1xOxidation [M1];1xTMT	Q92576	Q92576 [1125-1135]	Q92576 1xOxidation [M	0,01	-0,50	0,12
[R],RTSAYTLIAPNINR,[R]	1xTMT6plex [N-Term];1	Q96J88	Q96J88 [63-76]	Q96J88 1xTMT6plex [N	0,02	-0,50	0,13
[K],SVIESNRISR,[E]	1xTMT6plex [N-Term];1	B2RTY4	B2RTY4 [1211-1220]	B2RTY4 1xTMT6plex [N	0,04	-0,50	0,15
[R],ALALDLGSPAALR,[E]	1xTMT6plex [N-Term];1	Q8TER5	Q8TER5 [924-936]	Q8TER5 1xTMT6plex [N	0,01	-0,50	0,12
[R],HSLSSTK,[L]	1xTMT6plex [K7];1xTM	P42858	P42858 [1861-1867]	P42858 1xTMT6plex [K	0,02	-0,50	0,13
[K],SRDSESHSR,[V]	1xTMT6plex [N-Term];1	Q15424	Q15424 [614-622]	Q15424 1xTMT6plex [N	0,05	-0,50	0,16
[R],HHSVEIK,[I]	1xTMT6plex [K7];1xTM	Q14151	Q14151 [511-517]	Q14151 1xTMT6plex [K	0,02	-0,50	0,12
[R],LESLSETPGPSSPR,[Q]	1xTMT6plex [N-Term];1	Q12774	Q12774 [1115-1128]	Q12774 1xTMT6plex [N	0,02	-0,50	0,13
[K],TSNRYSPESQAQSVHHQR	1xTMT6plex [N-Term];1	O75376	O75376 [2097-2118]	O75376 1xTMT6plex [N	0,04	-0,49	0,15
[K],SYFAINHNPDAK,[D]	1xTMT6plex [K12];1xTM	Q9NQ69	Q9NQ69 [284-295]	Q9NQ69 1xTMT6plex [	0,01	-0,49	0,12
[R],RGNDPLTSSPGR,[S]	1xTMT6plex [N-Term];1	P49736	P49736 [19-30]	P49736 1xTMT6plex [N	0,03	-0,49	0,14
[K],VESTSPK,[T]	1xTMT6plex [K7];1xTM	P41229	P41229 [283-289]	P41229 1xTMT6plex [K	0,05	-0,49	0,16
[R],SFSPLQPGQAPTGR,[A]	1xTMT6plex [N-Term];1	Q7Z3C6	Q7Z3C6 [654-667]	Q7Z3C6 1xTMT6plex [N	0,03	-0,49	0,14
[R],FPPEDFRHSPEDFR,[R]	1xTMT6plex [N-Term];1	Q8IXT5	Q8IXT5 [567-580]	Q8IXT5 1xTMT6plex [N	0,01	-0,49	0,12
[R],SVASSQPAKPTK,[V]	2xTMT6plex [K9; K12];1	Q07157	Q07157 [175-186]	Q07157 2xTMT6plex [K	0,02	-0,49	0,13
[R],VASSSSSPK,[S]	1xTMT6plex [K9];1xTM	Q15652	Q15652 [1052-1060]	Q15652 1xTMT6plex [K	0,02	-0,49	0,13
[R],ASTPYIEK,[Q]	1xTMT6plex [K8];1xTM	Q969Z0	Q969Z0 [63-70]	Q969Z0 1xTMT6plex [K	0,05	-0,49	0,16
[K],APLKPYPVSPSDK,[V]	2xTMT6plex [K4; K13];1	P41252	P41252 [1039-1051]	P41252 2xTMT6plex [K	0,01	-0,49	0,12
[R],VISHDRDSPPPPPPPPPP	1xTMT6plex [K26];1xTM	Q69YN4	Q69YN4 [131-156]	Q69YN4 1xTMT6plex [K	0,01	-0,49	0,12
[R],IEEIKTPDSFEESQGEEIGK,	2xTMT6plex [K5; K20];1	Q5VZK9	Q5VZK9 [1123-1142]	Q5VZK9 2xTMT6plex [K	0,03	-0,49	0,14
[R],SRTLEGK,[L]	1xTMT6plex [K7];1xTM	P18615	P18615 [89-95]	P18615 1xTMT6plex [K	0,02	-0,49	0,13
[R],SPHQLLSPSSFSPSATPSQ	1xTMT6plex [K20];1xTM	Q14181	Q14181 [141-160]	Q14181 1xTMT6plex [K	0,00	-0,49	0,11
[R],SKLSPSPSLR,[K]	1xTMT6plex [K2];1xTM	Q5T200	Q5T200 [204-213]	Q5T200 1xTMT6plex [K	0,04	-0,49	0,15
[R],SFTAADSKDEEVK,[V]	2xTMT6plex [K8; K13];1	Q8N2U9	Q8N2U9 [110-122]	Q8N2U9 2xTMT6plex [K	0,01	-0,49	0,12
[R],QISQDVK,[L]	1xTMT6plex [K7];1xTM	Q01433	Q01433 [166-172]	Q01433 1xTMT6plex [K	0,05	-0,49	0,16
[K],KEPQSPR,[T]	1xTMT6plex [K1];1xTM	P43268	P43268 [97-103]	P43268 1xTMT6plex [K	0,01	-0,49	0,12
[R],EAGELPTSPLHLLSPGTPR,	1xTMT6plex [N-Term];1	Q969R5	Q969R5 [60-78]	Q969R5 1xTMT6plex [N	0,03	-0,49	0,14
[R],EAGELPTSPLHLLSPGTPR,	1xTMT6plex [N-Term];1	Q969R5	Q969R5 [60-78]	Q969R5 1xTMT6plex [N	0,03	-0,49	0,14
[R],GGNFGFGDSR,[G]	1xTMT6plex [N-Term];1	P22626	P22626 [204-213]	P22626 1xTMT6plex [N	0,02	-0,49	0,13
[R],RPTGDFGFSLR,[R]	1xTMT6plex [N-Term];1	Q92614	Q92614 [226-236]	Q92614 1xTMT6plex [N	0,03	-0,49	0,14
[R],EEKSPDDLKK,[K]	3xTMT6plex [K3; K9; K	Q99549	Q99549 [161-170]	Q99549 3xTMT6plex [K	0,00	-0,49	0,11
[K],LGSALLIR,[D]	1xTMT6plex [N-Term];1	Q14993	Q14993 [79-86]	Q14993 1xTMT6plex [N	0,03	-0,49	0,15
[R],KNTHENIQLSQSK,[K]	2xTMT6plex [K1; K13];1	Q6ZWT7	Q6ZWT7 [472-484]	Q6ZWT7 2xTMT6plex [	0,01	-0,49	0,12
[R],IHLGSSPK,[K]	1xTMT6plex [K8];1xTM	Q9H410	Q9H410 [53-60]	Q9H410 1xTMT6plex [K	0,02	-0,49	0,13
[K],NKASVLQK,[R]	1xMethyl [K2];1xTMT6p	A6QL64	A6QL64 [1402-1409]	A6QL64 1xMethyl [K140	0,02	-0,49	0,12
[R],ATLLESIR,[Q]	1xTMT6plex [N-Term];1	Q6VEQ5	Q6VEQ5 [363-370]	Q6VEQ5 1xTMT6plex [	0,01	-0,49	0,12
[R],SHVATCSK,[Y]	1xCarbamidomethyl [C6	Q9Y508	Q9Y508 [105-112]	Q9Y508 1xCarbamidom	0,04	-0,49	0,15
[R],SSPGAGPSDHHSASR,[D]	1xTMT6plex [N-Term];1	Q9Y2K7	Q9Y2K7 [739-753]	Q9Y2K7 1xTMT6plex [N	0,01	-0,49	0,12
[R],SIFASPESVTGK,[V]	1xTMT6plex [K12];1xTM	O75940	O75940 [197-208]	O75940 1xTMT6plex [K	0,02	-0,49	0,12
[R],KVYEDSGIPLPAESPK,[K]	2xTMT6plex [K1; K16];1	Q8IXM2	Q8IXM2 [83-98]	Q8IXM2 2xTMT6plex [K	0,04	-0,49	0,15
[R],YHGHSMSDPGVSYR,[T]	1xTMT6plex [N-Term];1	P08559	P08559 [289-302]	P08559 1xTMT6plex [N	0,00	-0,49	0,12

[K],SPTTTQSPK,[S]	1xTMT6plex [K9];1xTM	Q14669	Q14669 [1030-1038]	Q14669 1xTMT6plex [K	0,03	-0,49	0,14
[K],TGGTTSPK,[H]	1xTMT6plex [K8];1xTM	Q9UPA5	Q9UPA5 [432-439]	Q9UPA5 1xTMT6plex [I	0,00	-0,49	0,12
[K],SPSLSPSPPSPLEKTPLGEF	1xTMT6plex [K14];1xTM	P46821	P46821 [1256-1275]	P46821 1xTMT6plex [K	0,00	-0,49	0,11
[R],CSPVPGLSSSPSGSPLHGK	1xCarbamidomethyl [C1	Q9H6U6	Q9H6U6 [479-497]	Q9H6U6 1xCarbamidon	0,00	-0,49	0,11
[R],VLDDVSIRSPETK,[C]	1xTMT6plex [K13];1xTM	Q12830	Q12830 [1292-1304]	Q12830 1xTMT6plex [K	0,03	-0,49	0,14
[R],TPPSFPTPPPWLKPGELER	1xTMT6plex [K13];1xTM	Q8WXX7	Q8WXX7 [799-817]	Q8WXX7 1xTMT6plex [	0,01	-0,49	0,12
[K],KAAGGATPK,[K]	2xTMT6plex [K1; K9];1x	P16403	P16403 [140-148]	P16403 2xTMT6plex [K	0,01	-0,48	0,12
[R],VAFISLSPVR,[R]	1xTMT6plex [N-Term];1	Q6ZU65	Q6ZU65 [7-16]	Q6ZU65 1xTMT6plex [N	0,01	-0,48	0,12
[R],LEADQSPK,[N]	1xTMT6plex [K8];1xTM	P49454	P49454 [816-823]	P49454 1xTMT6plex [K	0,01	-0,48	0,12
[R],KHSPSPPPPTPTESR,[K]	1xTMT6plex [K1];1xTM	Q92922	Q92922 [326-340]	Q92922 1xTMT6plex [K	0,00	-0,48	0,11
[R],EKHPNSSPR,[T]	1xTMT6plex [K2];1xTM	P29374	P29374 [1158-1166]	P29374 1xTMT6plex [K	0,02	-0,48	0,12
[R],LVEDKPGSR,[R]	1xTMT6plex [K5];1xTM	Q08170	Q08170 [172-180]	Q08170 1xTMT6plex [K	0,01	-0,48	0,12
[R],FPGLALRPSSR,[E]	1xTMT6plex [N-Term];1	Q15735	Q15735 [894-904]	Q15735 1xTMT6plex [N	0,04	-0,48	0,15
[R],IGSDPYLGQR,[L]	1xTMT6plex [N-Term];1	Q00975	Q00975 [2254-2263]	Q00975 1xTMT6plex [N	0,03	-0,48	0,13
[R],SPSPQGTK,[A]	1xTMT6plex [K8];1xTM	Q9ULL5	Q9ULL5 [915-922]	Q9ULL5 1xTMT6plex [K	0,02	-0,48	0,13
[R],KTGGSSPETK,[Y]	2xTMT6plex [K1; K10];1	Q86Y38	Q86Y38 [241-250]	Q86Y38 2xTMT6plex [K	0,02	-0,48	0,13
[R],KVSLVLEK,[M]	2xTMT6plex [K1; K8];1x	Q9P2M7	Q9P2M7 [330-337]	Q9P2M7 2xTMT6plex [I	0,03	-0,48	0,14
[R],GSPLLGPVVPGPSPIPSVTE	1xTMT6plex [K21];1xTM	P49454	P49454 [2899-2919]	P49454 1xTMT6plex [K	0,02	-0,48	0,12
[K],RVSLVGADDLR,[K]	1xTMT6plex [N-Term];1	Q14160	Q14160 [1376-1386]	Q14160 1xTMT6plex [N	0,04	-0,48	0,15
[K],DSKPSSTPR,[S]	1xTMT6plex [K3];1xTM	P06748	P06748 [213-221]	P06748 1xTMT6plex [K	0,02	-0,48	0,12
[RK],RRSPSPR,[RFS]	1xTMT6plex [N-Term];1	Q8IYB3; (	Q8IYB3 [588-594]; Q5VUA4 [303-309	Q8IYB3 1xTMT6plex [N	0,03	-0,48	0,14
[R],SGPQSASTAVK,[E]	1xTMT6plex [K11];1xTM	Q9BTC0	Q9BTC0 [123-133]	Q9BTC0 1xTMT6plex [I	0,00	-0,48	0,12
[R],QSSTPSAPELGQQPDVNIS	1xTMT6plex [K22];1xTM	Q93100	Q93100 [699-720]	Q93100 1xTMT6plex [K	0,02	-0,48	0,12
[R],NPMSDPK,[K]	1xTMT6plex [K7];1xTM	P52948	P52948 [521-527]	P52948 1xTMT6plex [K	0,05	-0,48	0,16
[R],IVQISGNSMPR,[G]	1xOxidation [M9];1xTMT	Q9NWH9	Q9NWH9 [1007-1017]	Q9NWH9 1xOxidation [	0,04	-0,48	0,16
[R],MHLPSPTDSNFYR,[A]	1xOxidation [M1];1xTMT	P00533	P00533 [987-999]	P00533 1xOxidation [M	0,00	-0,48	0,11
[R],ASLYNAVTIEDVQK,[L]	1xTMT6plex [K14];1xTM	Q9Y617	Q9Y617 [343-356]	Q9Y617 1xTMT6plex [K	0,05	-0,48	0,16
[R],ITPPAAKPGSPQAK,[S]	2xTMT6plex [K7; K14];1	P35658	P35658 [669-682]	P35658 2xTMT6plex [K	0,01	-0,48	0,12
[R],TSPADGKPR,[L]	1xTMT6plex [K7];1xTM	Q12955	Q12955 [4332-4340]	Q12955 1xTMT6plex [K	0,01	-0,48	0,12
[R],VSGSFPEDSSK,[E]	1xTMT6plex [K11];1xTM	O15400	O15400 [128-138]	O15400 1xTMT6plex [K	0,01	-0,48	0,12
[K],AAGGATPK,[K]	1xTMT6plex [K8];1xTM	P16403	P16403 [141-148]	P16403 1xTMT6plex [K	0,03	-0,48	0,14
[R],TLGLSSPCDNR,[V]	1xCarbamidomethyl [C8	Q16828	Q16828 [346-356]	Q16828 1xCarbamidom	0,04	-0,48	0,15
[R],KPEDVLDDDDAGSAPLK,[S	2xTMT6plex [K1; K17];1	P35613	P35613 [350-366]	P35613 2xTMT6plex [K	0,03	-0,48	0,14
[R],WDSYENLSADGEVLHTQG	1xTMT6plex [K27];1xTM	Q68CZ2	Q68CZ2 [330-356]	Q68CZ2 1xTMT6plex [K	0,02	-0,48	0,13
[K],SASSPKPDTK,[V]	2xTMT6plex [K6; K10];1	Q8N111	Q8N111 [7-16]	Q8N111 2xTMT6plex [K	0,03	-0,48	0,14
[R],EVKLSPEGQK,[V]	2xTMT6plex [K3; K10];1	Q9C035	Q9C035 [82-91]	Q9C035 2xTMT6plex [K	0,04	-0,48	0,15
[K],ESPTQISIGAFPSTK,[I]	1xTMT6plex [K15];1xTM	Q5VWN6	Q5VWN6 [2008-2022]	Q5VWN6 1xTMT6plex [	0,01	-0,48	0,12
[R],VSQDLEVEKPDASPTSLQL	1xTMT6plex [K9];1xTM	O94885	O94885 [89-108]	O94885 1xTMT6plex [K	0,00	-0,48	0,11
[R],TPSRHSCSGSSPPR,[V]	1xCarbamidomethyl [C7	Q9UQ35	Q9UQ35 [885-898]	Q9UQ35 1xCarbamidon	0,01	-0,48	0,12
[R],DTSPSSGSAVSSSK,[V]	1xTMT6plex [K14];1xTM	Q8NEY8	Q8NEY8 [203-216]	Q8NEY8 1xTMT6plex [I	0,03	-0,48	0,13
[R],DQHHLGSPSR,[L]	1xTMT6plex [N-Term];1	Q9H8M2	Q9H8M2 [560-569]	Q9H8M2 1xTMT6plex [I	0,04	-0,48	0,15
[R],KPIDSLRDSR,[S]	1xTMT6plex [K1];1xTM	Q9UQ35	Q9UQ35 [2680-2689]	Q9UQ35 1xTMT6plex [I	0,01	-0,48	0,12
[R],QKSPEIHR,[R]	1xTMT6plex [K2];1xTM	Q9NYF8	Q9NYF8 [646-653]	Q9NYF8 1xTMT6plex [I	0,02	-0,48	0,13
[R],LLSMPGAQGAAAAGSEPPI	1xOxidation [M4];1xTMT	Q96KQ7	Q96KQ7 [151-179]	Q96KQ7 1xOxidation [M	0,00	-0,48	0,11
[K],APNTPDILEIEFK,[K]	1xTMT6plex [K13];1xTM	P00966	P00966 [216-228]	P00966 1xTMT6plex [K	0,05	-0,47	0,16
[R],KKSPIINESR,[S]	2xTMT6plex [K1; K2];1x	Q13523	Q13523 [275-284]	Q13523 2xTMT6plex [K	0,02	-0,47	0,13
[R],KPLSQQER,[D]	1xTMT6plex [K1];1xTM	Q8TDB6	Q8TDB6 [209-216]	Q8TDB6 1xTMT6plex [I	0,01	-0,47	0,12
[R],SHSPPLK,[L]	1xTMT6plex [K7];1xTM	Q86TP1	Q86TP1 [319-325]	Q86TP1 1xTMT6plex [K	0,01	-0,47	0,12
[R],SSTPLHSPSPIR,[V]	1xTMT6plex [N-Term];1	O95817	O95817 [283-294]	O95817 1xTMT6plex [N	0,02	-0,47	0,13
[R],QFSTASDHEQPGVSG,[-]	1xTMT6plex [N-Term];1	Q66K14	Q66K14 [1236-1250]	Q66K14 1xTMT6plex [N	0,00	-0,47	0,11
[K],KASLDTSELER,[A]	1xTMT6plex [K1];1xTM	O75962	O75962 [2120-2130]	O75962 1xTMT6plex [K	0,02	-0,47	0,13
[R],QSHSSSSPHPK,[V]	1xTMT6plex [K11];1xTM	Q9UQ35	Q9UQ35 [833-843]	Q9UQ35 1xTMT6plex [I	0,02	-0,47	0,12
[K],SKSPSPPR,[L]	1xTMT6plex [K2];1xTM	Q9UKV3	Q9UKV3 [384-391]	Q9UKV3 1xTMT6plex [I	0,01	-0,47	0,12
[K],TLPLDKSINHQIESPSER,[R]	1xTMT6plex [K6];1xTM	Q9UPQ0	Q9UPQ0 [960-977]	Q9UPQ0 1xTMT6plex [	0,02	-0,47	0,12
[R],KPGSHASSSAR,[R]	1xTMT6plex [K1];1xTM	Q9H7N4	Q9H7N4 [561-571]	Q9H7N4 1xTMT6plex [I	0,03	-0,47	0,14
[K],FPSSPLR,[I]	1xTMT6plex [N-Term];1	P06400	P06400 [792-798]	P06400 1xTMT6plex [N	0,02	-0,47	0,13
[R],AKTQTPPVSPAPQPTEER,[	1xTMT6plex [K2];1xTM	Q14247	Q14247 [397-414]	Q14247 1xTMT6plex [K	0,04	-0,47	0,15

[R],VLSPPGR,[R]	1xTMT6plex [N-Term];1	Q86VQ6	Q86VQ6 [30-36]	Q86VQ6 1xTMT6plex [N	0,01	-0,47	0,12
[K],GSPVTTTK,[A]	1xTMT6plex [K8];1xTM	Q7Z2K8	Q7Z2K8 [614-621]	Q7Z2K8 1xTMT6plex [K	0,01	-0,47	0,12
[R],GEIGGLSLSPSPER,[R]	1xTMT6plex [N-Term];1	Q96GP6	Q96GP6 [643-656]	Q96GP6 1xTMT6plex [N	0,04	-0,47	0,15
[R],KQDSGHLDLR,[N]	1xTMT6plex [K1];1xTM	Q86U44	Q86U44 [27-36]	Q86U44 1xTMT6plex [K	0,02	-0,47	0,12
[K],HVGHLLRTK,[A]	1xTMT6plex [K9];1xTM	P48637	P48637 [444-452]	P48637 1xTMT6plex [K	0,02	-0,47	0,13
[R],LPNNSSRPSTPTINVLESK,[	1xTMT6plex [K19];1xTM	Q15910	Q15910 [358-376]	Q15910 1xTMT6plex [K	0,05	-0,47	0,16
[R],ALLETASPR,[K]	1xTMT6plex [N-Term];1	Q96BT3	Q96BT3 [41-49]	Q96BT3 1xTMT6plex [N	0,00	-0,47	0,11
[R],GPSPEPLK,[L]	1xTMT6plex [K8];1xTM	Q92674	Q92674 [282-289]	Q92674 1xTMT6plex [K	0,02	-0,47	0,13
[K],QVHPDTGISSK,[A]	1xTMT6plex [K11];1xTM	Q16778; C	Q16778 [48-58]; Q5QNW6 [48-58]; C	Q16778 1xTMT6plex [K	0,00	-0,47	0,12
[K],LPGIPQGNLGAAVSPTSIHT	1xTMT6plex [K22];1xTM	P35711	P35711 [357-378]	P35711 1xTMT6plex [K	0,01	-0,47	0,12
[K],QQSPQEPK,[S]	1xTMT6plex [K8];1xTM	Q9UHB6	Q9UHB6 [696-703]	Q9UHB6 1xTMT6plex [	0,00	-0,47	0,12
[R],KPGSHASSSAR,[R]	1xTMT6plex [K1];1xTM	Q9H7N4	Q9H7N4 [561-571]	Q9H7N4 1xTMT6plex [K	0,01	-0,47	0,12
[R],RRPTLGVQLDDK,[R]	1xTMT6plex [K12];1xTM	Q13769	Q13769 [325-336]	Q13769 1xTMT6plex [K	0,01	-0,47	0,12
[K],AKEESPSK,[A]	2xTMT6plex [K2; K8];1x	Q6ZN18	Q6ZN18 [367-374]	Q6ZN18 2xTMT6plex [K	0,01	-0,47	0,12
[R],ASVALTPTAPSR,[T]	1xTMT6plex [N-Term];1	P28290	P28290 [1151-1162]	P28290 1xTMT6plex [N	0,02	-0,47	0,13
[R],SRSVSPHR,[G]	1xTMT6plex [N-Term];2	Q86UR5	Q86UR5 [951-958]	Q86UR5 1xTMT6plex [N	0,05	-0,47	0,16
[R],DREIMTSVTFGTPER,[R]	1xTMT6plex [N-Term];1	P35712	P35712 [108-122]	P35712 1xTMT6plex [N	0,01	-0,47	0,12
[R],SPSTESQLMSK,[G]	1xTMT6plex [K11];1xTM	P35612	P35612 [530-540]	P35612 1xTMT6plex [K	0,02	-0,46	0,12
[R],VSLEPHQGPGTPESK,[K]	1xTMT6plex [K15];1xTM	Q14980	Q14980 [1990-2004]	Q14980 1xTMT6plex [K	0,02	-0,46	0,13
[K],KPNSVPQELAATTEK,[T]	2xTMT6plex [K1; K15];1	Q9UPQ0	Q9UPQ0 [618-632]	Q9UPQ0 2xTMT6plex [	0,01	-0,46	0,12
[K],DLDRPESQSPK,[R]	1xTMT6plex [K11];1xTM	Q8WUA4	Q8WUA4 [159-169]	Q8WUA4 1xTMT6plex [	0,03	-0,46	0,13
[R],LINSELGSPSR,[T]	1xTMT6plex [N-Term];1	Q68CP4	Q68CP4 [236-246]	Q68CP4 1xTMT6plex [N	0,01	-0,46	0,12
[R],SPTESVNTPVGK,[D]	1xTMT6plex [K12];1xTM	Q8WXG6	Q8WXG6 [1059-1070]	Q8WXG6 1xTMT6plex [	0,01	-0,46	0,12
[K],RPSAPFR,[S]	1xTMT6plex [N-Term];1	Q9NZH0	Q9NZH0 [366-372]	Q9NZH0 1xTMT6plex [N	0,00	-0,46	0,12
[R],RSPESCSKPEK,[I]	1xCarbamidomethyl [C6	Q03111	Q03111 [474-484]	Q03111 1xCarbamidom	0,04	-0,46	0,15
[K],SPHDSKSPLDHR,[S]	1xTMT6plex [K6];1xTM	O14647	O14647 [1795-1806]	O14647 1xTMT6plex [K	0,04	-0,46	0,16
[K],VQTTPSKPGGDR,[Y]	1xTMT6plex [K7];1xTM	Q12834	Q12834 [67-78]	Q12834 1xTMT6plex [K	0,01	-0,46	0,12
[K],GSSPKPK,[S]	2xTMT6plex [K5; K7];1x	Q9UKJ3	Q9UKJ3 [437-443]	Q9UKJ3 2xTMT6plex [K	0,04	-0,46	0,15
[R],LKSIEER,[Q]	1xTMT6plex [K2];1xTM	O15164	O15164 [1040-1046]	O15164 1xTMT6plex [K	0,04	-0,46	0,15
[R],RVPPPALSR,[D]	1xTMT6plex [N-Term];1	Q14152	Q14152 [1329-1337]	Q14152 1xTMT6plex [N	0,01	-0,46	0,12
[K],NTTSSPCK,[G]	1xCarbamidomethyl [C7	Q7Z5J4	Q7Z5J4 [1547-1554]	Q7Z5J4 1xCarbamidom	0,04	-0,46	0,16
[R],YSHSGSSSPDTK,[V]	1xTMT6plex [K12];1xTM	Q9UQ35	Q9UQ35 [967-978]	Q9UQ35 1xTMT6plex [	0,01	-0,46	0,12
[R],SSPPSAGNSPSSLK,[F]	1xTMT6plex [K14];1xTM	P57682	P57682 [70-83]	P57682 1xTMT6plex [K	0,05	-0,46	0,16
[K],HIEQLTTASEHCDLAIK,[T]	1xCarbamidomethyl [C1	P23508	P23508 [191-207]	P23508 1xCarbamidom	0,03	-0,46	0,13
[R],SPGPAADGPIDLSK,[K]	1xTMT6plex [K14];1xTM	Q8IX07	Q8IX07 [786-799]	Q8IX07 1xTMT6plex [K	0,01	-0,46	0,12
[K],NPPENSDSGTGKK,[E]	2xTMT6plex [K12; K13]	P55010	P55010 [144-156]	P55010 2xTMT6plex [K	0,02	-0,46	0,13
[K],SLHTLFGDK,[L]	1xTMT6plex [K9];1xTM	P02768	P02768 [89-97]	P02768 1xTMT6plex [K	0,05	-0,46	0,16
[R],KSSTPEEVK,[K]	2xTMT6plex [K1; K9];1x	P23528	P23528 [22-30]	P23528 2xTMT6plex [K	0,00	-0,46	0,11
[K],IEEAMDGSETPQLFTVLPEK	1xTMT6plex [K20];1xTM	Q13435	Q13435 [771-790]	Q13435 1xTMT6plex [K	0,04	-0,46	0,15
[R],QGLGPIHSR,[T]	1xTMT6plex [N-Term];1	Q99835	Q99835 [764-772]	Q99835 1xTMT6plex [N	0,02	-0,46	0,12
[K],MISTPSPK,[K]	1xOxidation [M1];1xTMT	Q53F19	Q53F19 [410-417]	Q53F19 1xOxidation [M	0,01	-0,46	0,12
[K],YSPTSPTYSPTSPVYTPTSP	1xTMT6plex [K21];1xTM	P24928	P24928 [1888-1908]	P24928 1xTMT6plex [K	0,02	-0,46	0,13
[K],YSPTSPTYSPTSPVYTPTSP	1xPhospho [T4];1xTMT	P24928	P24928 [1888-1908]	P24928 1xPhospho [T1	0,02	-0,46	0,13
[K],YSPTSPTYSPTSPVYTPTSP	1xPhospho [Y1];1xTMT	P24928	P24928 [1888-1908]	P24928 1xPhospho [Y1	0,02	-0,46	0,13
[R],KLSPGPTTLK,[R]	2xTMT6plex [K1; K10];1	Q5T6C5	Q5T6C5 [573-582]	Q5T6C5 2xTMT6plex [K	0,03	-0,46	0,13
[K],VWSPLVTEEGKR,[H]	1xTMT6plex [K11];1xTM	O00151	O00151 [88-99]	O00151 1xTMT6plex [K	0,01	-0,46	0,12
[R],RMSDPPLSTMPTAPGLR,[K	1xTMT6plex [N-Term];1	P78314	P78314 [276-292]	P78314 1xTMT6plex [N	0,03	-0,46	0,14
[K],RYHSLGNISR,[G]	1xTMT6plex [N-Term];1	Q09019	Q09019 [542-551]	Q09019 1xTMT6plex [N	0,02	-0,46	0,12
[K],LQSPIKEENTTAVEEIGR,[T	1xTMT6plex [K6];1xTM	Q9NS73	Q9NS73 [89-106]	Q9NS73 1xTMT6plex [K	0,03	-0,46	0,13
[R],VAELALSLSSTSDDEPPSSV	1xTMT6plex [K25];1xTM	Q8N1G2	Q8N1G2 [20-44]	Q8N1G2 1xTMT6plex [	0,03	-0,46	0,14
[R],GSLACPSPTCFSPQESPSK	2xCarbamidomethyl [C5	Q7Z2K8	Q7Z2K8 [89-107]	Q7Z2K8 2xCarbamidom	0,01	-0,46	0,12
[R],TSSDSALHTSVMNPSPQDT	1xOxidation [M12];1xTM	Q53ET0	Q53ET0 [169-200]	Q53ET0 1xOxidation [M	0,03	-0,46	0,14
[R],SSSSSASPSSPSSR,[E]	1xTMT6plex [N-Term];1	Q9NYF8	Q9NYF8 [751-764]	Q9NYF8 1xTMT6plex [N	0,02	-0,46	0,12
[K],RLSSSTVAISYSPDR,[V]	1xTMT6plex [N-Term];1	Q96T17	Q96T17 [184-198]	Q96T17 1xTMT6plex [N	0,01	-0,46	0,12
[R],KDTDDIESPKR,[S]	2xTMT6plex [K1; K10];1	Q9UPQ0	Q9UPQ0 [162-172]	Q9UPQ0 2xTMT6plex [	0,00	-0,46	0,11
[R],KGTTPPRSPEASPK,[H]	2xTMT6plex [K1; K14];1	Q9HDC5	Q9HDC5 [458-471]	Q9HDC5 2xTMT6plex [	0,03	-0,45	0,13
[K],VEQNSEPCAGSSSESDLQT	1xCarbamidomethyl [C8	Q8N806	Q8N806 [253-274]	Q8N806 1xCarbamidom	0,03	-0,45	0,13

[K],VEQNSEPCAGSSSESDLQT	1xCarbamidomethyl [C8	Q8N806	Q8N806 [253-274]	Q8N806 1xCarbamidom	0,03	-0,45	0,13
[K],ATPKLDSSPSVSSTLAAK,[D	2xTMT6plex [K4; K18];1	Q13428	Q13428 [1221-1238]	Q13428 2xTMT6plex [K	0,00	-0,45	0,11
[R],LNASPAAR,[E]	1xTMT6plex [N-Term];1	Q9UGU0	Q9UGU0 [571-578]	Q9UGU0 1xTMT6plex [	0,02	-0,45	0,12
[R],DASVFQDESNMSVLDIPSA	1xTMT6plex [K23];1xTM	P21675	P21675 [1661-1683]	P21675 1xTMT6plex [K	0,05	-0,45	0,16
[R],VKSSTPPRQSPSR,[S]	1xTMT6plex [K2];1xTM	Q9UQ35	Q9UQ35 [899-911]	Q9UQ35 1xTMT6plex [I	0,02	-0,45	0,12
[R],VSEGGPAEIAGLQIGDK,[I]	1xTMT6plex [K17];1xTM	O14907	O14907 [60-76]	O14907 1xTMT6plex [K	0,01	-0,45	0,12
[R],SLLGMLSDLQVYK,[D]	1xTMT6plex [K13];1xTM	Q13619	Q13619 [216-228]	Q13619 1xTMT6plex [K	0,03	-0,45	0,14
[R],APTPPSTPVPLPEK,[R]	1xTMT6plex [K14];1xTM	Q9UMN6	Q9UMN6 [586-599]	Q9UMN6 1xTMT6plex [	0,04	-0,45	0,15
[R],SPISPELHSAPLTPVAR,[D]	1xTMT6plex [N-Term];1	Q7Z3B3	Q7Z3B3 [991-1007]	Q7Z3B3 1xTMT6plex [N	0,00	-0,45	0,12
[K],WTPVGPAPSTSQSQK,[R]	1xTMT6plex [K15];1xTM	Q9UHB7	Q9UHB7 [108-122]	Q9UHB7 1xTMT6plex [I	0,00	-0,45	0,12
[K],IESFGSPK,[G]	1xTMT6plex [K8];1xTM	P49792	P49792 [3202-3209]	P49792 1xTMT6plex [K	0,02	-0,45	0,12
[K],AKPASPAR,[R]	1xTMT6plex [K2];1xTM	P78559	P78559 [2613-2620]	P78559 1xTMT6plex [K	0,00	-0,45	0,11
[R],LESLSETPGPSSPR,[Q]	1xTMT6plex [N-Term];1	Q12774	Q12774 [1115-1128]	Q12774 1xTMT6plex [N	0,01	-0,45	0,12
[K],KLSPGDR,[V]	1xTMT6plex [K1];1xTM	P31629	P31629 [627-633]	P31629 1xTMT6plex [K	0,01	-0,45	0,12
[R],ISSSSFSR,[V]	1xTMT6plex [N-Term];1	P05787	P05787 [33-40]	P05787 1xTMT6plex [N	0,03	-0,45	0,14
[R],ENSPSSQSAGLSSINK,[E]	1xTMT6plex [K16];1xTM	Q9H2Y7	Q9H2Y7 [1277-1292]	Q9H2Y7 1xTMT6plex [K	0,01	-0,45	0,12
[R],LRSPPEALVQGR,[Y]	1xTMT6plex [N-Term];1	Q9UHB6	Q9UHB6 [130-141]	Q9UHB6 1xTMT6plex [I	0,01	-0,45	0,12
[K],SPENTEGKDGSKVTK,[Q]	3xTMT6plex [K8; K12; K	Q15651	Q15651 [6-20]	Q15651 3xTMT6plex [K	0,01	-0,45	0,12
[R],QSHSSSSPHPK,[V]	1xTMT6plex [K11];1xTM	Q9UQ35	Q9UQ35 [833-843]	Q9UQ35 1xTMT6plex [I	0,04	-0,45	0,15
[K],LGIHEDSQNR,[K]	1xTMT6plex [N-Term];1	P07900	P07900 [447-456]	P07900 1xTMT6plex [N	0,02	-0,45	0,12
[K],LEASDCDHQQNSPTLERPG	1xCarbamidomethyl [C6	Q9HAN9	Q9HAN9 [106-125]	Q9HAN9 1xCarbamidon	0,00	-0,45	0,12
[K],KTSPQVK,[S]	2xTMT6plex [K1; K7];1x	Q6ZUT1	Q6ZUT1 [135-141]	Q6ZUT1 2xTMT6plex [K	0,03	-0,45	0,14
[R],SSTLSQLPGDK,[S]	1xTMT6plex [K11];1xTM	O60271	O60271 [593-603]	O60271 1xTMT6plex [K	0,04	-0,45	0,15
[R],IDFTPVSPAPSPTR,[G]	1xTMT6plex [N-Term];2	Q7Z309	Q7Z309 [109-122]	Q7Z309 1xTMT6plex [N	0,01	-0,45	0,12
[K],FTPVASKFSPGAPGGSGSQ	2xTMT6plex [K7; K23];1	Q15942	Q15942 [273-295]	Q15942 2xTMT6plex [K	0,00	-0,45	0,11
[R],VPSPTPAPK,[E]	1xTMT6plex [K9];1xTM	Q9UQ35	Q9UQ35 [2579-2587]	Q9UQ35 1xTMT6plex [I	0,01	-0,45	0,12
[R],DTPGHGSGWAETPR,[T]	1xTMT6plex [N-Term];1	O75533	O75533 [302-315]	O75533 1xTMT6plex [N	0,02	-0,45	0,12
[R],FLMPEAYPSSPR,[K]	1xTMT6plex [N-Term];1	O43166	O43166 [153-164]	O43166 1xTMT6plex [N	0,03	-0,45	0,14
[K],SVASLNRSPERR,[K]	1xTMT6plex [N-Term];1	Q12923	Q12923 [1026-1037]	Q12923 1xTMT6plex [N	0,00	-0,45	0,12
[R],SQSFTHSPSGDPK,[A]	1xTMT6plex [K13];1xTM	Q9Y4F5	Q9Y4F5 [419-431]	Q9Y4F5 1xTMT6plex [K	0,02	-0,45	0,13
[R],LPNNSSRPSTPTINVLESK,[	1xTMT6plex [K19];1xTM	Q15910	Q15910 [358-376]	Q15910 1xTMT6plex [K	0,02	-0,45	0,13
[R],GISLLHEVDTQYSALK,[V]	1xTMT6plex [K16];1xTM	Q01850	Q01850 [342-357]	Q01850 1xTMT6plex [K	0,00	-0,45	0,12
[K],TSQVGAASAPAKESPR,[K]	1xTMT6plex [K12];1xTM	Q13428	Q13428 [368-383]	Q13428 1xTMT6plex [K	0,01	-0,45	0,12
[K],DVLSPLRPSR,[R]	1xTMT6plex [N-Term];1	Q12756	Q12756 [1091-1100]	Q12756 1xTMT6plex [N	0,01	-0,45	0,12
[K],LLKEGEEPTVYSDEEEPK,[I	2xTMT6plex [K3; K18];1	O00264	O00264 [170-187]	O00264 2xTMT6plex [K	0,00	-0,45	0,11
[R],SNSPLPVPPSK,[A]	1xTMT6plex [K11];1xTM	Q13247	Q13247 [301-311]	Q13247 1xTMT6plex [K	0,01	-0,45	0,12
[R],TPSLPTPPTR,[E]	1xTMT6plex [N-Term];1	P10636	P10636 [529-538]	P10636 1xTMT6plex [N	0,01	-0,45	0,12
[K],AGMSSNQSISSPVLDAVPR	1xTMT6plex [N-Term];1	Q9UQ35	Q9UQ35 [1394-1412]	Q9UQ35 1xTMT6plex [I	0,00	-0,45	0,11
[R],GAESPFEEKSGK,[Q]	2xTMT6plex [K9; K12];1	P46821	P46821 [1424-1435]	P46821 2xTMT6plex [K	0,00	-0,45	0,11
[R],TVSDSIK,[K]	1xTMT6plex [K7];1xTM	Q9Y490	Q9Y490 [1639-1645]	Q9Y490 1xTMT6plex [K	0,04	-0,45	0,15
[R],SLHSAHSLASR,[R]	1xTMT6plex [N-Term];1	O15027	O15027 [1356-1366]	O15027 1xTMT6plex [N	0,01	-0,45	0,12
[R],GLVSVPK,[Y]	1xTMT6plex [K7];1xTM	Q9P2D7	Q9P2D7 [482-488]	Q9P2D7 1xTMT6plex [K	0,01	-0,45	0,12
[R],GMYDGPVFDLTTTPKGGTP	1xTMT6plex [K15];1xTM	Q14195	Q14195 [497-520]	Q14195 1xTMT6plex [K	0,02	-0,45	0,12
[R],TSEEDTPKK,[K]	2xTMT6plex [K8; K9];1x	Q8WYH8	Q8WYH8 [147-155]	Q8WYH8 2xTMT6plex [	0,05	-0,45	0,16
[R],KLESLDALEPEEK,[A]	2xTMT6plex [K1; K13];1	Q14807	Q14807 [559-571]	Q14807 2xTMT6plex [K	0,02	-0,45	0,13
[R],SVSHGSNHTQKPDEQR,[S]	1xTMT6plex [K11];1xTM	Q9Y520	Q9Y520 [924-939]	Q9Y520 1xTMT6plex [K	0,02	-0,45	0,13
[K],QVHPDTGISSK,[A]	1xTMT6plex [K11];1xTM	Q16778; Q	Q16778 [48-58]; Q5QNW6 [48-58]; Q	Q16778 1xTMT6plex [K	0,02	-0,45	0,12
[R],TISDGTISASKQESGK,[M]	2xTMT6plex [K11; K16]	Q5T0N5	Q5T0N5 [293-308]	Q5T0N5 2xTMT6plex [K	0,00	-0,44	0,11
[R],FITLLLPGGAQTAVRPGSPS	1xTMT6plex [N-Term];1	Q9Y4A5	Q9Y4A5 [1611-1635]	Q9Y4A5 1xTMT6plex [N	0,03	-0,44	0,14
[R],LKSEDGVEGDLGETQSR,[T	1xTMT6plex [K2];1xTM	Q09666	Q09666 [133-149]	Q09666 1xTMT6plex [K	0,05	-0,44	0,16
[R],ALSTTASTAAFDK,[Q]	1xTMT6plex [K13];1xTM	P57772	P57772 [26-38]	P57772 1xTMT6plex [K	0,00	-0,44	0,12
[R],ISQDLALIAR,[E]	1xTMT6plex [N-Term];1	Q5SW79	Q5SW79 [1305-1314]	Q5SW79 1xTMT6plex [	0,03	-0,44	0,14
[K],ADSDSEDKGEESKPK,[K]	3xTMT6plex [K8; K13; K	P83916	P83916 [87-101]	P83916 3xTMT6plex [K	0,01	-0,44	0,12
[R],KGSVVNVNPTNTRPQSDTP	1xTMT6plex [K1];1xTM	O95819	O95819 [898-919]	O95819 1xTMT6plex [K	0,01	-0,44	0,12
[R],EFTGSPPSSATK,[K]	1xTMT6plex [K12];1xTM	Q92609	Q92609 [550-561]	Q92609 1xTMT6plex [K	0,01	-0,44	0,12
[K],RLSLPADIR,[IL]	1xTMT6plex [N-Term];1	Q00537; Q	Q00537 [144-152]; Q00536 [117-125	Q00537 1xTMT6plex [N	0,04	-0,44	0,15
[R],SVASSQPAKPTK,[V]	2xTMT6plex [K9; K12];1	Q07157	Q07157 [175-186]	Q07157 2xTMT6plex [K	0,00	-0,44	0,11

[K],ALGVISNFQSSPK,[Y]	1xTMT6plex [K13];1xTN	O94876	O94876 [404-416]	O94876 1xTMT6plex [K	0,02	-0,44	0,12
[K],LDRSHDK,[S]	1xTMT6plex [K7];1xTM	O60828	O60828 [103-109]	O60828 1xTMT6plex [K	0,05	-0,44	0,16
[R],SHKEPPSPADVPEK,[T]	2xTMT6plex [K3; K14];1	Q86VP3	Q86VP3 [343-356]	Q86VP3 2xTMT6plex [K	0,04	-0,44	0,15
[R],QEIESETTSEEQIQEEK,[S]	1xTMT6plex [K17];1xTN	P13611	P13611 [2108-2124]	P13611 1xTMT6plex [K	0,03	-0,44	0,13
[R],RLSSSLNPSK,[R]	1xTMT6plex [K10];1xTN	Q13535	Q13535 [433-442]	Q13535 1xTMT6plex [K	0,03	-0,44	0,14
[RK],LGKDSPSNK,[L]	2xTMT6plex [K3; K9];1x	P51805; Q	P51805 [1766-1774]; Q9UIW2 [1791-	P51805 2xTMT6plex [K	0,04	-0,44	0,15
[R],VTAEDKGTGNK,[N]	2xTMT6plex [K6; K11];1	P11021	P11021 [511-521]	P11021 2xTMT6plex [K	0,03	-0,44	0,14
[R],LSEQLAHTPTAFK,[R]	1xTMT6plex [K13];1xTN	Q9UBF8	Q9UBF8 [510-522]	Q9UBF8 1xTMT6plex [K	0,01	-0,44	0,12
[K],LSPPVASGGIPHQSPPTK,[V	1xTMT6plex [K18];1xTN	Q96T58	Q96T58 [2480-2497]	Q96T58 1xTMT6plex [K	0,00	-0,44	0,12
[K],AFTIQGAPSDSGPLR,[I]	1xTMT6plex [N-Term];1	P49418	P49418 [242-256]	P49418 1xTMT6plex [N	0,01	-0,44	0,12
[R],HNSISEAK,[M]	1xTMT6plex [K8];1xTM	Q01970	Q01970 [1103-1110]	Q01970 1xTMT6plex [K	0,04	-0,44	0,15
[K],VGPGNHGTEGSGGER,[H]	1xTMT6plex [N-Term];1	Q99442	Q99442 [325-339]	Q99442 1xTMT6plex [N	0,02	-0,44	0,13
[R],SSSETILSSLAGSDIVK,[G]	1xTMT6plex [K17];1xTN	Q01850	Q01850 [309-325]	Q01850 1xTMT6plex [K	0,00	-0,44	0,12
[R],TKEGVGSPK,[L]	2xTMT6plex [K2; K9];1x	Q5T4S7	Q5T4S7 [451-459]	Q5T4S7 2xTMT6plex [K	0,03	-0,44	0,14
[K],SLPKQASDTGSNDAHNK,[K	2xTMT6plex [K4; K17];1	P20810	P20810 [65-81]	P20810 2xTMT6plex [K	0,01	-0,44	0,12
[R],IMGPNYTPGKK,[E]	2xTMT6plex [K10; K11]	P13639	P13639 [429-439]	P13639 2xTMT6plex [K	0,02	-0,44	0,13
[R],SKSETGDSSIFR,[K]	1xTMT6plex [K2];1xTM	O43166	O43166 [286-297]	O43166 1xTMT6plex [K	0,02	-0,44	0,13
[R],SYSPDGKESPSDKK,[S]	3xTMT6plex [K7; K13; K	P43243	P43243 [596-609]	P43243 3xTMT6plex [K	0,01	-0,44	0,12
[R],KSPSPGR,[R]	1xTMT6plex [K1];1xTM	Q9HCG8	Q9HCG8 [90-96]	Q9HCG8 1xTMT6plex [	0,05	-0,44	0,16
[R],SGYSSPGSPGTPGSR,[S]	1xTMT6plex [N-Term];1	P10636	P10636 [512-526]	P10636 1xTMT6plex [N	0,03	-0,44	0,14
[R],IKSDDETCR,[N]	1xCarbamidomethyl [C8	Q8IZD2	Q8IZD2 [916-924]	Q8IZD2 1xCarbamidom	0,01	-0,44	0,12
[K],LDLLGNLPGSKR,[RQ]	1xTMT6plex [K11];1xTN	P46013	P46013 [1928-1939]; [2411-2422]	P46013 1xTMT6plex [K	0,01	-0,44	0,12
[R],GLSEHNALLQR,[D]	1xTMT6plex [N-Term];1	Q00975	Q00975 [2231-2241]	Q00975 1xTMT6plex [N	0,03	-0,44	0,13
[K],EKPSFELSGALLEDTNTFR,	1xTMT6plex [K2];1xTM	Q8TAD8	Q8TAD8 [222-240]	Q8TAD8 1xTMT6plex [K	0,03	-0,44	0,14
[R],KADSDSEDKGEESKPK,[K]	1xMethyl [K9];3xTMT6p	P83916	P83916 [86-101]	P83916 1xMethyl [K94];	0,00	-0,44	0,12
[R],TAITTPNFK,[K]	1xTMT6plex [K9];1xTM	Q9BXS6	Q9BXS6 [178-186]	Q9BXS6 1xTMT6plex [K	0,05	-0,44	0,16
[R],SQYNFIADVVEK,[T]	1xTMT6plex [K12];1xTN	O43464	O43464 [145-156]	O43464 1xTMT6plex [K	0,05	-0,44	0,16
[K],LSPNPPNLTK,[K]	1xTMT6plex [K10];1xTN	P51532	P51532 [1451-1460]	P51532 1xTMT6plex [K	0,03	-0,44	0,13
[R],SKSPGLGAPEEPQEIPLK,[T	2xTMT6plex [K2; K18];1	Q9BXA9	Q9BXA9 [917-934]	Q9BXA9 2xTMT6plex [K	0,02	-0,44	0,12
[K],GPGQPSSPQR,[L]	1xTMT6plex [N-Term];1	O95400	O95400 [189-198]	O95400 1xTMT6plex [N	0,03	-0,44	0,14
[R],SYSLSELSVLQAK,[S]	1xTMT6plex [K13];1xTN	Q9P0V3	Q9P0V3 [244-256]	Q9P0V3 1xTMT6plex [K	0,01	-0,44	0,12
[K],KPESTDDEEK,[I]	2xTMT6plex [K1; K10];1	O95714	O95714 [1562-1571]	O95714 2xTMT6plex [K	0,04	-0,44	0,15
[R],HASTSSPADK,[A]	1xTMT6plex [K10];1xTN	Q5T4S7	Q5T4S7 [1758-1767]	Q5T4S7 1xTMT6plex [K	0,02	-0,44	0,12
[R],SKSDSYTLDPDTLR,[K]	1xTMT6plex [K2];1xTM	O75592	O75592 [2869-2882]	O75592 1xTMT6plex [K	0,00	-0,44	0,12
[R],QLSALHR,[A]	1xTMT6plex [N-Term];1	P61313	P61313 [32-38]	P61313 1xTMT6plex [N	0,01	-0,44	0,12
[R],QLSMTLR,[G]	1xTMT6plex [N-Term];1	Q00536	Q00536 [10-16]	Q00536 1xTMT6plex [N	0,04	-0,44	0,16
[K],GKNSDEEAPK,[T]	2xTMT6plex [K2; K10];1	Q9UNL4	Q9UNL4 [147-156]	Q9UNL4 2xTMT6plex [K	0,02	-0,44	0,13
[K],SKSPSPPR,[L]	1xTMT6plex [K2];1xTM	Q9UKV3	Q9UKV3 [384-391]	Q9UKV3 1xTMT6plex [I	0,01	-0,43	0,12
[R],FGLGSPLPSPR,[A]	1xTMT6plex [N-Term];1	Q14934	Q14934 [209-219]	Q14934 1xTMT6plex [N	0,04	-0,43	0,16
[R],SSPNVANQPPSPGGK,[S]	1xTMT6plex [K15];1xTN	P55196	P55196 [1172-1186]	P55196 1xTMT6plex [K	0,05	-0,43	0,16
[R],SPSPQGTK,[A]	1xTMT6plex [K8];1xTM	Q9ULL5	Q9ULL5 [915-922]	Q9ULL5 1xTMT6plex [K	0,03	-0,43	0,13
[R],KHPSSPAR,[V]	1xTMT6plex [K1];1xTM	Q9ULW0	Q9ULW0 [289-296]	Q9ULW0 1xTMT6plex [	0,02	-0,43	0,13
[R],LFSADPFDLEAQAK,[I]	1xTMT6plex [K14];1xTN	Q5TDH0	Q5TDH0 [192-205]	Q5TDH0 1xTMT6plex [I	0,04	-0,43	0,15
[K],KPDSPPK,[V]	2xTMT6plex [K1; K7];1x	Q96T23	Q96T23 [745-751]	Q96T23 2xTMT6plex [K	0,02	-0,43	0,13
[R],AHSLNTVK,[Q]	1xTMT6plex [K8];1xTM	O43572	O43572 [171-178]	O43572 1xTMT6plex [K	0,01	-0,43	0,12
[R],TLSDPPSPLPHGPPNK,[G]	1xTMT6plex [K16];1xTN	Q9ULH1	Q9ULH1 [837-852]	Q9ULH1 1xTMT6plex [K	0,00	-0,43	0,12
[R],IGSTIFGER,[D]	1xTMT6plex [N-Term];1	O94903	O94903 [242-250]	O94903 1xTMT6plex [N	0,03	-0,43	0,13
[R],SPGAPGPLTLK,[E]	1xTMT6plex [K11];1xTN	Q15942	Q15942 [344-354]	Q15942 1xTMT6plex [K	0,02	-0,43	0,13
[K],NLQYYDISAK,[S]	1xTMT6plex [K10];1xTN	P62826	P62826 [143-152]	P62826 1xTMT6plex [K	0,02	-0,43	0,12
[R],EHALLAYTLGVK,[Q]	1xTMT6plex [K12];1xTN	Q05639; P	Q05639 [135-146]; P68104 [135-146]	Q05639 1xTMT6plex [K	0,00	-0,43	0,12
[K],STGGISVPGPMGPSGPR,[G	1xOxidation [M11];1xTN	P02452	P02452 [171-187]	P02452 1xOxidation [M	0,01	-0,43	0,12
[R],YSSDPTGALTEDSIDDTFLF	1xTMT6plex [K31];1xTN	P00533	P00533 [1069-1099]	P00533 1xTMT6plex [K	0,03	-0,43	0,13
[R],LASTSDIEEK,[E]	1xTMT6plex [K10];1xTN	O14974	O14974 [505-514]	O14974 1xTMT6plex [K	0,03	-0,43	0,14
[R],SSSASSPPKK,[E]	2xTMT6plex [K9; K10];1	P51608	P51608 [355-364]	P51608 2xTMT6plex [K	0,01	-0,43	0,12
[R],KTTSTEEPR,[G]	1xTMT6plex [K1];1xTM	Q5THJ4	Q5THJ4 [2090-2098]	Q5THJ4 1xTMT6plex [K	0,02	-0,43	0,12
[R],SQSFTHSPSGDPK,[A]	1xTMT6plex [K13];1xTN	Q9Y4F5	Q9Y4F5 [419-431]	Q9Y4F5 1xTMT6plex [K	0,00	-0,43	0,12
[R],VDSGTEKPGLVAPESPVR,[	1xTMT6plex [K7];1xTM	O15021	O15021 [1765-1782]	O15021 1xTMT6plex [K	0,01	-0,43	0,12

[K],RATPINLASAIR,[K]	1xTMT6plex [N-Term];1	P55199	P55199 [178-189]	P55199 1xTMT6plex [N	0,03	-0,43	0,13
[R],SPTGPSNSFLANMGGTVAI	1xOxidation [M13];1xTM	Q96I25	Q96I25 [222-241]	Q96I25 1xOxidation [M2	0,02	-0,43	0,13
[R],SMSLIPTSPQVPGEWPSPE	1xOxidation [M2];1xTMT	Q5PRF9	Q5PRF9 [236-259]	Q5PRF9 1xOxidation [M	0,03	-0,43	0,13
[K],TGGKEAASGTTPQK,[S]	2xTMT6plex [K4; K14];1	Q13428	Q13428 [1260-1273]	Q13428 2xTMT6plex [K	0,00	-0,43	0,11
[R],QGSPGSVSK,[Q]	1xTMT6plex [K9];1xTM	O00425	O00425 [182-190]	O00425 1xTMT6plex [K	0,01	-0,43	0,12
[R],SSSPGKLLGSGYGGLTGGS	1xTMT6plex [K6];1xTM	Q7Z460	Q7Z460 [686-706]	Q7Z460 1xTMT6plex [K	0,00	-0,43	0,11
[K],AHSEVFTKPSGQQTLSPDR	1xTMT6plex [K8];1xTM	P15822	P15822 [2667-2685]	P15822 1xTMT6plex [K	0,00	-0,43	0,12
[K],HAYKDDSPRR,[R]	1xTMT6plex [K4];1xTM	Q02040	Q02040 [627-636]	Q02040 1xTMT6plex [K	0,02	-0,43	0,13
[R],AAPTTPPPPVK,[R]	1xTMT6plex [K11];1xTM	Q9NZQ3	Q9NZQ3 [177-187]	Q9NZQ3 1xTMT6plex [	0,01	-0,43	0,12
[R],SKSDGEAKPEPSPSPR,[I]	2xTMT6plex [K2; K8];1x	P26358	P26358 [141-156]	P26358 2xTMT6plex [K	0,02	-0,43	0,12
[K],APSIDGK,[E]	1xTMT6plex [K7];1xTM	Q9H0E9	Q9H0E9 [385-391]	Q9H0E9 1xTMT6plex [K	0,02	-0,43	0,13
[R],NTVDLVTTCHIHSSSDDEID	1xCarbamidomethyl [C9	Q5H9R7	Q5H9R7 [511-532]	Q5H9R7 1xCarbamidom	0,01	-0,43	0,12
[K],TASESISNLSEAGSIK,[K]	1xTMT6plex [K16];1xTM	P30622	P30622 [191-206]	P30622 1xTMT6plex [K	0,01	-0,43	0,12
[R],TVGTNTPPSPGFGWR,[A]	1xTMT6plex [N-Term];1	Q9HBL0	Q9HBL0 [1184-1198]	Q9HBL0 1xTMT6plex [N	0,02	-0,43	0,13
[R],SPVPAQIAITVPK,[T]	1xTMT6plex [K13];1xTM	O43432	O43432 [495-507]	O43432 1xTMT6plex [K	0,01	-0,43	0,12
[R],EVSGHSVR,[G]	1xTMT6plex [N-Term];1	Q9NWH9	Q9NWH9 [907-914]	Q9NWH9 1xTMT6plex [	0,01	-0,43	0,12
[R],RYSDFEWLR,[S]	1xTMT6plex [N-Term];1	O60493	O60493 [70-78]	O60493 1xTMT6plex [N	0,02	-0,43	0,13
[R],AESLPLSSNLQSK,[E]	1xTMT6plex [K13];1xTM	Q8N1W1	Q8N1W1 [533-545]	Q8N1W1 1xTMT6plex [	0,01	-0,43	0,12
[R],TASFSESR,[A]	1xTMT6plex [N-Term];1	P53396	P53396 [453-460]	P53396 1xTMT6plex [N	0,01	-0,43	0,12
[R],KSSLTQEEAPVSWEK,[R]	2xTMT6plex [K1; K15];1	Q76L83	Q76L83 [569-583]	Q76L83 2xTMT6plex [K	0,02	-0,43	0,13
[R],YMSQMSVPEQAELEK,[L]	1xTMT6plex [K15];1xTM	Q15154	Q15154 [88-102]	Q15154 1xTMT6plex [K	0,01	-0,43	0,12
[R],AHSLLFENSDSFSEDSSTL	1xTMT6plex [N-Term];1	Q8IZ21	Q8IZ21 [441-461]	Q8IZ21 1xTMT6plex [N	0,03	-0,42	0,14
[R],GSLTPHPAACDK,[D]	1xCarbamidomethyl [C1	P55201	P55201 [843-854]	P55201 1xCarbamidom	0,02	-0,42	0,13
[R],TASFSESR,[A]	1xTMT6plex [N-Term];1	P53396	P53396 [453-460]	P53396 1xTMT6plex [N	0,00	-0,42	0,11
[R],TEHSLHYSPTSR,[Q]	1xTMT6plex [N-Term];1	P51587	P51587 [2088-2099]	P51587 1xTMT6plex [N	0,01	-0,42	0,12
[R],YSPSQNSPIHHIPSR,[R]	1xTMT6plex [N-Term];2	Q9NYF8	Q9NYF8 [284-298]	Q9NYF8 1xTMT6plex [N	0,01	-0,42	0,12
[R],EFTGSPPSSATKK,[D]	2xTMT6plex [K12; K13]	Q92609	Q92609 [550-562]	Q92609 2xTMT6plex [K	0,02	-0,42	0,12
[R],DYLQAQHPPSPIK,[S]	1xTMT6plex [K13];1xTM	Q9P219	Q9P219 [218-230]	Q9P219 1xTMT6plex [K	0,00	-0,42	0,12
[K],IGPLGLSPK,[K]	1xTMT6plex [K9];1xTM	P30050	P30050 [32-40]	P30050 1xTMT6plex [K	0,02	-0,42	0,13
[K],EEQQQALEQFAAAAAHSTP	1xTMT6plex [N-Term];1	Q9Y2J2	Q9Y2J2 [39-59]	Q9Y2J2 1xTMT6plex [N	0,01	-0,42	0,12
[R],ISVSSPGR,[G]	1xTMT6plex [N-Term];1	O75864	O75864 [557-564]	O75864 1xTMT6plex [N	0,03	-0,42	0,13
[R],SKLSPSPSLR,[K]	1xTMT6plex [K2];1xTM	Q5T200	Q5T200 [204-213]	Q5T200 1xTMT6plex [K	0,03	-0,42	0,14
[R],SKTQLSPSIK,[R]	2xTMT6plex [K2; K10];1	Q8NFC6	Q8NFC6 [3014-3023]	Q8NFC6 2xTMT6plex [	0,00	-0,42	0,11
[R],RPGHGSLTNISR,[H]	1xTMT6plex [N-Term];1	Q7Z6B7	Q7Z6B7 [912-923]	Q7Z6B7 1xTMT6plex [N	0,01	-0,42	0,12
[K],LAPEEEAGGAGTPVITEIFS	1xTMT6plex [N-Term];1	Q9UIS9	Q9UIS9 [559-583]	Q9UIS9 1xTMT6plex [N	0,04	-0,42	0,16
[R],FSTYSQSPPDTPSLR,[E]	1xTMT6plex [N-Term];1	Q6ZS17	Q6ZS17 [345-359]	Q6ZS17 1xTMT6plex [N	0,02	-0,42	0,12
[R],KRNSEDEAQEAK,[D]	2xTMT6plex [K1; K12];1	A2RRP1	A2RRP1 [1934-1945]	A2RRP1 2xTMT6plex [K	0,02	-0,42	0,12
[R],YSHSGSSSPDTK,[V]	1xTMT6plex [K12];1xTM	Q9UQ35	Q9UQ35 [967-978]	Q9UQ35 1xTMT6plex [	0,03	-0,42	0,13
[R],SDSPPAPLRPWVPLR,[K]	1xTMT6plex [N-Term];1	Q15772	Q15772 [417-431]	Q15772 1xTMT6plex [N	0,01	-0,42	0,12
[R],LLSDPELSATESPLADK,[K]	1xTMT6plex [K17];1xTM	Q9H694	Q9H694 [677-693]	Q9H694 1xTMT6plex [K	0,01	-0,42	0,12
[R],ELHPQLLSPTK,[A]	1xTMT6plex [K11];1xTM	Q8TBN0	Q8TBN0 [172-182]	Q8TBN0 1xTMT6plex [K	0,01	-0,42	0,12
[K],SSPSKHQPPPIR,[N]	1xTMT6plex [K5];1xTM	Q14194	Q14194 [521-532]	Q14194 1xTMT6plex [K	0,03	-0,42	0,13
[R],TPQLKDSSQTSR,[F]	1xTMT6plex [K5];1xTM	Q6PKG0	Q6PKG0 [788-799]	Q6PKG0 1xTMT6plex [	0,02	-0,42	0,13
[R],KSSTPEEVK,[G]	2xTMT6plex [K1; K9];1x	P23528	P23528 [22-30]	P23528 2xTMT6plex [K	0,02	-0,42	0,13
[R],KQHSYIEQGK,[K]	2xTMT6plex [K1; K10];1	P49711	P49711 [458-467]	P49711 2xTMT6plex [K	0,04	-0,42	0,15
[R],AEQLASVQYTLPK,[T]	1xTMT6plex [K13];1xTM	Q07157	Q07157 [580-592]	Q07157 1xTMT6plex [K	0,05	-0,42	0,16
[R],LGAGGGSPEKSPSAQELK,	2xTMT6plex [K10; K18]	Q9UNE7	Q9UNE7 [13-30]	Q9UNE7 2xTMT6plex [	0,01	-0,42	0,12
[K],KDGWNQNHFITPVSTLER,[	1xTMT6plex [K1];1xTM	Q99569	Q99569 [1003-1020]	Q99569 1xTMT6plex [K	0,00	-0,42	0,12
[R],RQSSGSATNVASTPDNR,[	1xTMT6plex [N-Term];1	Q7Z460	Q7Z460 [644-660]	Q7Z460 1xTMT6plex [N	0,02	-0,42	0,13
[K],LPPPETPDACLK,[L]	1xCarbamidomethyl [C1	Q7Z5J4	Q7Z5J4 [1292-1303]	Q7Z5J4 1xCarbamidom	0,02	-0,42	0,12
[R],KSPTMEQAVQTASAHLPAF	1xTMT6plex [K1];1xTM	Q8ND56	Q8ND56 [191-214]	Q8ND56 1xTMT6plex [K	0,00	-0,42	0,12
[K],SKGHYEVTGSDDETGK,[L]	2xTMT6plex [K2; K16];1	Q09666	Q09666 [5832-5847]	Q09666 2xTMT6plex [K	0,01	-0,42	0,12
[K],ASEGASSESLLSVPGQK,[N	1xTMT6plex [K17];1xTM	Q8TDJ6	Q8TDJ6 [924-940]	Q8TDJ6 1xTMT6plex [K	0,00	-0,42	0,12
[R],TPVSPVK,[F]	1xTMT6plex [K7];1xTM	P23443	P23443 [444-450]	P23443 1xTMT6plex [K	0,02	-0,42	0,12
[R],QHLGATGGPGAQLGASFL	1xTMT6plex [N-Term];1	Q9NWW5	Q9NWW5 [8-28]	Q9NWW5 1xTMT6plex	0,02	-0,42	0,12
[R],RHSEVETDSKK,[K]	2xTMT6plex [K10; K11]	Q9NX58	Q9NX58 [274-284]	Q9NX58 2xTMT6plex [K	0,03	-0,42	0,13
[R],AGSISTLDSLDFAR,[Y]	1xTMT6plex [N-Term];1	Q9P260	Q9P260 [178-191]	Q9P260 1xTMT6plex [N	0,00	-0,42	0,11

[R],LLLDIPLQTPHK,[L]	1xTMT6plex [K12];1xTM	P49792; Q	P49792 [2145-2156]; Q99666 [1169-1	P49792 1xTMT6plex [K	0,02	-0,42	0,13
[R],SPLEHSSPEK,[E]	1xTMT6plex [K10];1xTM	Q13459	Q13459 [1109-1118]	Q13459 1xTMT6plex [K	0,01	-0,42	0,12
[R],SRTSPITR,[R]	1xTMT6plex [N-Term];1	Q9UQ35	Q9UQ35 [1972-1979]	Q9UQ35 1xTMT6plex [I	0,00	-0,42	0,12
[R],SPVPSAFSDQSR,[C]	1xTMT6plex [N-Term];1	Q9UQ35	Q9UQ35 [2449-2460]	Q9UQ35 1xTMT6plex [I	0,04	-0,42	0,15
[K],KPAQEETEETSSQESAEED	1xTMT6plex [K1];1xTM	P52926	P52926 [91-109]	P52926 1xTMT6plex [K	0,01	-0,42	0,12
[R],AYTHQVVTR,[W]	1xTMT6plex [N-Term];1	P50613	P50613 [168-176]	P50613 1xTMT6plex [N	0,01	-0,42	0,12
[R],SPHQLLSPSSFSPSATPSQ	1xTMT6plex [K20];1xTM	Q14181	Q14181 [141-160]	Q14181 1xTMT6plex [K	0,00	-0,42	0,12
[R],FLEDDPSDPTYTSSLGGK,[	1xTMT6plex [K18];1xTM	P54753	P54753 [782-799]	P54753 1xTMT6plex [K	0,02	-0,42	0,13
[R],FLEDDPSDPTYTSSLGGK,[	1xTMT6plex [K18];1xTM	P54753	P54753 [782-799]	P54753 1xTMT6plex [K	0,02	-0,42	0,13
[K],TSSSSPLSSPSK,[S]	1xTMT6plex [K12];1xTM	Q8IWB9	Q8IWB9 [158-169]	Q8IWB9 1xTMT6plex [K	0,01	-0,42	0,12
[R],QSHSGSISPYPK,[V]	1xTMT6plex [K12];1xTM	Q9UQ35	Q9UQ35 [987-998]	Q9UQ35 1xTMT6plex [I	0,02	-0,42	0,13
[R],SSAPFSPPSGPPEK,[-]	1xTMT6plex [K14];1xTM	Q5TZA2	Q5TZA2 [2004-2017]	Q5TZA2 1xTMT6plex [K	0,00	-0,42	0,12
[R],SPGGHSDGASPR,[S]	1xTMT6plex [N-Term];1	Q63HR2	Q63HR2 [991-1002]	Q63HR2 1xTMT6plex [N	0,03	-0,42	0,13
[R],QTQSASSTLQK,[H]	1xTMT6plex [K11];1xTM	O95819	O95819 [839-849]	O95819 1xTMT6plex [K	0,00	-0,42	0,12
[R],YHGHSMSDPGVSYR,[T]	1xOxidation [M6];1xTMT	P08559	P08559 [289-302]	P08559 1xOxidation [M	0,00	-0,42	0,12
[R],LGSVEAPK,[T]	1xTMT6plex [K8];1xTM	P46087	P46087 [56-63]	P46087 1xTMT6plex [K	0,01	-0,42	0,12
[K],VAQGATEKSPEDK,[V]	2xTMT6plex [K8; K13];1	P49321	P49321 [443-455]	P49321 2xTMT6plex [K	0,01	-0,42	0,12
[R],STSVPAPYISVTPDASPNVF	1xTMT6plex [K28];1xTM	O75592	O75592 [3424-3451]	O75592 1xTMT6plex [K	0,03	-0,42	0,14
[R],STSVPAPYISVTPDASPNVF	1xTMT6plex [K28];1xTM	O75592	O75592 [3424-3451]	O75592 1xTMT6plex [K	0,03	-0,42	0,14
[R],SLSSTSSGSK,[D]	1xTMT6plex [K10];1xTM	P23508	P23508 [681-690]	P23508 1xTMT6plex [K	0,03	-0,42	0,13
[R],HFSEHPSTSK,[M]	1xTMT6plex [K10];1xTM	O43719	O43719 [401-410]	O43719 1xTMT6plex [K	0,02	-0,41	0,12
[R],GQSPQSSPDLPVHYIPVAP	1xTMT6plex [N-Term];1	Q9H2A7	Q9H2A7 [232-254]	Q9H2A7 1xTMT6plex [N	0,03	-0,41	0,14
[R],HPSPQSPDR,[G]	1xTMT6plex [N-Term];1	Q8N3V7	Q8N3V7 [230-238]	Q8N3V7 1xTMT6plex [N	0,01	-0,41	0,12
[R],RLSEGVEPWR,[G]	1xTMT6plex [N-Term];1	Q92794	Q92794 [939-948]	Q92794 1xTMT6plex [N	0,02	-0,41	0,13
[K],LSPSPSLR,[K]	1xTMT6plex [N-Term];1	Q5T200	Q5T200 [206-213]	Q5T200 1xTMT6plex [N	0,05	-0,41	0,17
[R],HSIAQLDPEALGNIK,[K]	1xTMT6plex [K15];1xTM	Q53HL2	Q53HL2 [249-263]	Q53HL2 1xTMT6plex [K	0,02	-0,41	0,12
[R],LSGSDLGGHSSLLER,[L]	1xTMT6plex [N-Term];1	O95613	O95613 [2476-2490]	O95613 1xTMT6plex [N	0,01	-0,41	0,12
[R],VTPTLPK,[Q]	1xTMT6plex [K7];1xTM	Q9UPP1	Q9UPP1 [885-891]	Q9UPP1 1xTMT6plex [I	0,03	-0,41	0,14
[R],YHCAIPGSK,[K]	1xCarbamidomethyl [C3	Q709C8	Q709C8 [3634-3642]	Q709C8 1xCarbamidom	0,01	-0,41	0,12
[R],VKSSTPPRQSPSR,[S]	1xTMT6plex [K2];1xTM	Q9UQ35	Q9UQ35 [899-911]	Q9UQ35 1xTMT6plex [I	0,01	-0,41	0,12
[R],TVKSEHETFK,[F]	2xTMT6plex [K3; K10];1	Q9P2F8	Q9P2F8 [297-306]	Q9P2F8 2xTMT6plex [K	0,03	-0,41	0,13
[K],EVIKSPTASR,[I]	1xTMT6plex [K4];1xTM	O94885	O94885 [438-447]	O94885 1xTMT6plex [K	0,02	-0,41	0,12
[R],SRASPVSR,[R]	1xTMT6plex [N-Term];1	Q9UQ35	Q9UQ35 [1913-1920]	Q9UQ35 1xTMT6plex [I	0,01	-0,41	0,12
[R],SNSPLPVPPSK,[A]	1xTMT6plex [K11];1xTM	Q13247	Q13247 [301-311]	Q13247 1xTMT6plex [K	0,02	-0,41	0,13
[R],TISQSSSLK,[S]	1xTMT6plex [K9];1xTM	Q9UHB7	Q9UHB7 [834-842]	Q9UHB7 1xTMT6plex [I	0,01	-0,41	0,12
[RK],SPSLSSK,[HQ]	1xTMT6plex [K7];1xTM	Q8IYB3; Q	Q8IYB3 [653-659]; Q9UPU5 [1141-1	Q8IYB3 1xTMT6plex [K	0,01	-0,41	0,12
[R],KSHSPVK,[R]	2xTMT6plex [K1; K7];1x	P78549	P78549 [50-56]	P78549 2xTMT6plex [K	0,00	-0,41	0,12
[K],RPSGSLGK,[R]	1xTMT6plex [K8];1xTM	Q9NZH0	Q9NZH0 [358-365]	Q9NZH0 1xTMT6plex [I	0,00	-0,41	0,12
[R],IRTPSYSPTQR,[S]	1xTMT6plex [N-Term];1	Q03164	Q03164 [2145-2155]	Q03164 1xTMT6plex [N	0,00	-0,41	0,11
[KR],GTGASGSFK,[L]	1xTMT6plex [K9];1xTM	P16401; F	P16401 [101-109]; P16402 [99-107];	P16401 1xTMT6plex [K	0,05	-0,41	0,16
[R],GISPIVFDR,[S]	1xTMT6plex [N-Term];1	Q96MU7	Q96MU7 [306-314]	Q96MU7 1xTMT6plex [I	0,02	-0,41	0,12
[R],GYPSPGAHSPR,[A]	1xTMT6plex [N-Term];1	Q63HR2	Q63HR2 [817-827]	Q63HR2 1xTMT6plex [N	0,02	-0,41	0,12
[R],VSSLAGFTDCHR,[T]	1xCarbamidomethyl [C1	Q96QT4	Q96QT4 [1475-1486]	Q96QT4 1xCarbamidom	0,00	-0,41	0,12
[K],TIGGGDDSFNTFFSETGAG	1xTMT6plex [K20];1xTM	Q71U36	Q71U36 [41-60]	Q71U36 1xTMT6plex [K	0,03	-0,41	0,14
[R],APSPGAYK,[T]	1xTMT6plex [K8];1xTM	P54259	P54259 [643-650]	P54259 1xTMT6plex [K	0,03	-0,41	0,13
[R],SPIIHSPK,[R]	1xTMT6plex [K8];1xTM	O94913	O94913 [489-496]	O94913 1xTMT6plex [K	0,01	-0,41	0,12
[K],MISTPSPK,[K]	1xTMT6plex [K8];1xTM	Q53F19	Q53F19 [410-417]	Q53F19 1xTMT6plex [K	0,01	-0,41	0,12
[R],EESGKPGAHVTVK,[K]	2xTMT6plex [K5; K13];1	P22626	P22626 [100-112]	P22626 2xTMT6plex [K	0,02	-0,41	0,12
[R],TDSTSDGRPAWMR,[T]	1xTMT6plex [N-Term];1	Q14204	Q14204 [4366-4378]	Q14204 1xTMT6plex [N	0,01	-0,41	0,12
[R],LSHLSDSK,[N]	1xTMT6plex [K8];1xTM	Q14690	Q14690 [396-403]	Q14690 1xTMT6plex [K	0,02	-0,41	0,12
[R],SPSKPLPEVTDEYK,[N]	2xTMT6plex [K4; K14];1	P05455	P05455 [92-105]	P05455 2xTMT6plex [K	0,00	-0,41	0,11
[K],IEEVLSPEGSPSKSPSK,[K]	2xTMT6plex [K13; K17]	Q9UEY8	Q9UEY8 [668-684]	Q9UEY8 2xTMT6plex [I	0,00	-0,41	0,11
[K],DHSVNSFK,[N]	1xTMT6plex [K8];1xTM	Q9Y4E1	Q9Y4E1 [907-914]	Q9Y4E1 1xTMT6plex [K	0,01	-0,41	0,12
[K],IEEVLSPEGSPSKSPSK,[K]	2xTMT6plex [K13; K17]	Q9UEY8	Q9UEY8 [668-684]	Q9UEY8 2xTMT6plex [I	0,00	-0,41	0,11
[R],GLQLTPGIGGMQQHFFDDI	1xTMT6plex [N-Term];1	P12270	P12270 [2112-2132]	P12270 1xTMT6plex [N	0,03	-0,41	0,14
[R],KASPEPEGEAAGKMTTEE(	2xTMT6plex [K1; K13];1	Q8IU81	Q8IU81 [382-403]	Q8IU81 2xTMT6plex [K	0,02	-0,41	0,13
[K],RVETSEHFR,[I]	1xTMT6plex [N-Term];1	P42167	P42167 [261-269]	P42167 1xTMT6plex [N	0,01	-0,41	0,12

[R],ESSPLYSPTFSDSTSAVKEI	2xTMT6plex [K18; K20]	P46821	P46821 [1791-1810]	P46821 2xTMT6plex [K	0,00	-0,41	0,11
[R],LHTVGGPGSARPR,[A]	1xTMT6plex [N-Term];1	Q96RK0	Q96RK0 [341-353]	Q96RK0 1xTMT6plex [N	0,03	-0,41	0,14
[R],SYSSSHSSPAK,[I]	1xTMT6plex [K11];1xTM	Q9H792	Q9H792 [723-733]	Q9H792 1xTMT6plex [K	0,03	-0,41	0,14
[R],KTSEDQAAILPGK,[L]	2xTMT6plex [K1; K13];1	Q12968	Q12968 [342-354]	Q12968 2xTMT6plex [K	0,01	-0,41	0,12
[R],TPSSPHK,[K]	1xTMT6plex [K7];1xTM	Q9C0A6	Q9C0A6 [1122-1128]	Q9C0A6 1xTMT6plex [K	0,02	-0,41	0,13
[K],HAHSSSLQQAASR,[S]	1xTMT6plex [N-Term];1	O95425	O95425 [248-260]	O95425 1xTMT6plex [N	0,02	-0,41	0,13
[K],GKDSTEMR,[R]	1xTMT6plex [K2];1xTM	P52292	P52292 [21-28]	P52292 1xTMT6plex [K	0,05	-0,41	0,16
[R],HSASPVVFTSAR,[S]	1xTMT6plex [N-Term];1	O43166	O43166 [1430-1441]	O43166 1xTMT6plex [N	0,01	-0,41	0,12
[K],ASVITQVFHVPLEER,[K]	1xTMT6plex [N-Term];1	Q00341	Q00341 [73-87]	Q00341 1xTMT6plex [N	0,02	-0,41	0,13
[K],EDVSISPGEFEHNFLGENK,	1xTMT6plex [K19];1xTM	Q99550	Q99550 [262-280]	Q99550 1xTMT6plex [K	0,02	-0,41	0,12
[K],AAAVGTANK,[S]	1xTMT6plex [K9];1xTM	P18206	P18206 [647-655]	P18206 1xTMT6plex [K	0,03	-0,41	0,14
[R],EFSGGSYSAK,[R]	1xTMT6plex [K10];1xTM	Q5VT25	Q5VT25 [1649-1658]	Q5VT25 1xTMT6plex [K	0,01	-0,41	0,12
[R],GGFDSPFYR,[D]	1xTMT6plex [N-Term];1	Q92574	Q92574 [501-509]	Q92574 1xTMT6plex [N	0,02	-0,41	0,12
[K],EIPFNEDPNPNTHSSGPSTI	1xTMT6plex [K22];1xTM	Q86YS7	Q86YS7 [267-288]	Q86YS7 1xTMT6plex [K	0,00	-0,41	0,11
[K],EIPFNEDPNPNTHSSGPSTI	1xTMT6plex [K22];1xTM	Q86YS7	Q86YS7 [267-288]	Q86YS7 1xTMT6plex [K	0,00	-0,41	0,11
[R],SPQTLAPVGEDAMK,[T]	1xOxidation [M13];1xTM	Q92797	Q92797 [1243-1256]	Q92797 1xOxidation [M	0,04	-0,41	0,15
[R],SGAHSSASPPRSR,[S]	1xTMT6plex [N-Term];2	P18615	P18615 [174-186]	P18615 1xTMT6plex [N	0,02	-0,41	0,13
[R],HPLPSPK,[N]	1xTMT6plex [K7];1xTM	Q9BYP7	Q9BYP7 [901-907]	Q9BYP7 1xTMT6plex [K	0,00	-0,41	0,12
[R],KTSLVIVESADNQPETCER,	1xCarbamidomethyl [C1	Q01484	Q01484 [3843-3861]	Q01484 1xCarbamidom	0,03	-0,41	0,14
[R],AQPSASLGVGYR,[T]	1xTMT6plex [N-Term];1	Q96PK6	Q96PK6 [251-262]	Q96PK6 1xTMT6plex [N	0,02	-0,40	0,13
[R],ATAPQTQHVSPMR,[Q]	1xOxidation [M12];1xTM	P29692	P29692 [124-136]	P29692 1xOxidation [M	0,02	-0,40	0,13
[K],STASPASTK,[T]	1xTMT6plex [K9];1xTM	Q9ULU4	Q9ULU4 [472-480]	Q9ULU4 1xTMT6plex [K	0,05	-0,40	0,16
[R],GSSPSHSATSVHTSV,[-]	1xTMT6plex [N-Term];1	Q13613	Q13613 [651-665]	Q13613 1xTMT6plex [N	0,01	-0,40	0,12
[R],LLASPSSR,[S]	1xTMT6plex [N-Term];1	Q15003	Q15003 [84-91]	Q15003 1xTMT6plex [N	0,04	-0,40	0,15
[K],RLDSSACLHAVGDK,[A]	1xCarbamidomethyl [C7	O94808	O94808 [241-254]	O94808 1xCarbamidom	0,00	-0,40	0,12
[K],IWDPTPSHTPAGAATPGR,[	1xTMT6plex [N-Term];1	O75533	O75533 [253-270]	O75533 1xTMT6plex [N	0,00	-0,40	0,12
[K],KSDANASYLR,[A]	1xTMT6plex [K1];1xTM	Q12955	Q12955 [38-47]	Q12955 1xTMT6plex [K	0,04	-0,40	0,15
[R],RHSVTDKR,[D]	1xTMT6plex [K7];1xTM	Q17R89	Q17R89 [800-807]	Q17R89 1xTMT6plex [K	0,03	-0,40	0,13
[R],ASGEMASAQYITAALR,[D]	1xOxidation [M5];1xTM	P54578	P54578 [142-157]	P54578 1xOxidation [M	0,03	-0,40	0,14
[R],LSTSTTIENIEITIK,[S]	1xTMT6plex [K15];1xTM	Q9UEY8	Q9UEY8 [649-663]	Q9UEY8 1xTMT6plex [I	0,02	-0,40	0,13
[R],INHTVILDDPFDDPPDLLIPD	1xTMT6plex [N-Term];1	Q8WUA2	Q8WUA2 [156-183]	Q8WUA2 1xTMT6plex [	0,02	-0,40	0,13
[R],ERSPALKSPLQSVVVR,[R]	1xTMT6plex [K7];1xTM	Q9Y2W1	Q9Y2W1 [246-261]	Q9Y2W1 1xTMT6plex [	0,01	-0,40	0,12
[K],LSDSYSNTLPVR,[K]	1xTMT6plex [N-Term];1	Q9UQB8	Q9UQB8 [333-344]	Q9UQB8 1xTMT6plex [	0,04	-0,40	0,15
[R],GSLASLDSLRK,[G]	1xTMT6plex [K11];1xTM	O60716	O60716 [345-355]	O60716 1xTMT6plex [K	0,03	-0,40	0,14
[R],KSSSISEEK,[G]	2xTMT6plex [K1; K9];1x	Q9UKV3	Q9UKV3 [205-213]	Q9UKV3 2xTMT6plex [I	0,02	-0,40	0,13
[R],LFVENDSPSDGGTPGR,[M]	1xTMT6plex [N-Term];1	Q08999	Q08999 [682-697]	Q08999 1xTMT6plex [N	0,03	-0,40	0,14
[R],IHHTVEEK,[E]	1xTMT6plex [K8];1xTM	Q9UKV3	Q9UKV3 [762-769]	Q9UKV3 1xTMT6plex [I	0,01	-0,40	0,12
[R],RASDTSLTQGLK,[A]	1xTMT6plex [K12];1xTM	A0A0B4J2	A0A0B4J2F2 [573-584]	A0A0B4J2F2 1xTMT6pl	0,02	-0,40	0,12
[K],QDDSPPRPIIGPALPPGFIK,	1xTMT6plex [K20];1xTM	Q8IXQ4	Q8IXQ4 [102-121]	Q8IXQ4 1xTMT6plex [K	0,02	-0,40	0,12
[K],DSKPSSTPR,[S]	1xTMT6plex [K3];1xTM	P06748	P06748 [213-221]	P06748 1xTMT6plex [K	0,01	-0,40	0,12
[R],SPVPSPGSSSPQLQVK,[S]	1xTMT6plex [K16];1xTM	Q8N3F8	Q8N3F8 [612-627]	Q8N3F8 1xTMT6plex [K	0,01	-0,40	0,12
[R],RKSDDVHLR,[K]	1xTMT6plex [K2];1xTM	Q8NHM5	Q8NHM5 [793-801]	Q8NHM5 1xTMT6plex [	0,03	-0,40	0,14
[R],QSHSGSISPYPK,[V]	1xTMT6plex [K12];1xTM	Q9UQ35	Q9UQ35 [987-998]	Q9UQ35 1xTMT6plex [I	0,01	-0,40	0,12
[R],RTSFLEGTLR,[R]	1xTMT6plex [N-Term];1	Q9HD67	Q9HD67 [1881-1890]	Q9HD67 1xTMT6plex [N	0,02	-0,40	0,13
[R],IQNSPQK,[K]	1xTMT6plex [K7];1xTM	Q96MN5	Q96MN5 [11-17]	Q96MN5 1xTMT6plex [I	0,05	-0,40	0,16
[R],KHSEEAEFTPPLK,[C]	2xTMT6plex [K1; K13];1	Q3B726	Q3B726 [314-326]	Q3B726 2xTMT6plex [K	0,02	-0,40	0,13
[K],IQQNQSIR,[R]	1xTMT6plex [N-Term];1	O75116	O75116 [1369-1376]	O75116 1xTMT6plex [N	0,05	-0,40	0,16
[K],TPTSSPASSPLVAK,[K]	1xTMT6plex [K14];1xTM	Q14684	Q14684 [728-741]	Q14684 1xTMT6plex [K	0,03	-0,40	0,14
[R],SPPPSLAK,[Q]	1xTMT6plex [K8];1xTM	P54284	P54284 [152-159]	P54284 1xTMT6plex [K	0,02	-0,40	0,12
[R],SRSHSAGK,[S]	1xTMT6plex [K8];1xTM	Q08170	Q08170 [257-264]	Q08170 1xTMT6plex [K	0,04	-0,40	0,16
[K],QSILPSPK,[F]	1xTMT6plex [K8];1xTM	Q96JA4	Q96JA4 [235-242]	Q96JA4 1xTMT6plex [K	0,02	-0,40	0,13
[R],ALLETASPR,[K]	1xTMT6plex [N-Term];1	Q96BT3	Q96BT3 [41-49]	Q96BT3 1xTMT6plex [N	0,00	-0,40	0,12
[R],ALAAGADSPK,[T]	1xTMT6plex [K10];1xTM	Q4KMP7	Q4KMP7 [125-134]	Q4KMP7 1xTMT6plex [	0,05	-0,40	0,16
[K],ERTLSADASVNTLPVVVSR,	1xTMT6plex [N-Term];1	Q92615	Q92615 [564-582]	Q92615 1xTMT6plex [N	0,01	-0,40	0,12
[K],LESTSPK,[G]	1xTMT6plex [K7];1xTM	Q03111	Q03111 [263-269]	Q03111 1xTMT6plex [K	0,05	-0,40	0,16
[R],SLEPAENVHGAGGGAFPAS	1xTMT6plex [K24];1xTM	P12931	P12931 [17-48]	P12931 1xTMT6plex [K	0,00	-0,40	0,11
[R],LPSTSGSEGVPFR,[T]	1xTMT6plex [N-Term];1	P29317	P29317 [895-907]	P29317 1xTMT6plex [N	0,04	-0,40	0,15

[R],SASPPQK,[R]	1xTMT6plex [K7];1xTM	P55199	P55199 [317-323]	P55199 1xTMT6plex [K	0,02	-0,40	0,13
[R],RRSDSLK,[E]	1xTMT6plex [K7];1xTM	Q8WXF0	Q8WXF0 [132-138]	Q8WXF0 1xTMT6plex [	0,01	-0,40	0,12
[R],SPSPAHLPDDPK,[V]	1xTMT6plex [K12];1xTM	Q92615	Q92615 [599-610]	Q92615 1xTMT6plex [K	0,01	-0,40	0,12
[K],SHNDFVAILDLPEGEHQYK,	1xTMT6plex [K19];1xTM	O43741	O43741 [108-126]	O43741 1xTMT6plex [K	0,04	-0,40	0,16
[K],DKDVTLSPVK,[A]	2xTMT6plex [K2; K10];1	Q8NFC6	Q8NFC6 [1525-1534]	Q8NFC6 2xTMT6plex [I	0,01	-0,40	0,12
[R],RNSFTPLSSSNTIR,[R]	1xTMT6plex [N-Term];1	O60825	O60825 [464-477]	O60825 1xTMT6plex [N	0,00	-0,40	0,12
[R],IQVAQEKQVAEQGGDLSPA	1xTMT6plex [K7];1xTM	O94804	O94804 [422-443]	O94804 1xTMT6plex [K	0,00	-0,40	0,11
[K],ARSPTDDKVK,[I]	2xTMT6plex [K8; K10];1	Q13523	Q13523 [255-264]	Q13523 2xTMT6plex [K	0,00	-0,39	0,11
[K],KDDSHSAEDSEDEKEDHK,	3xTMT6plex [K1; K14; K	Q9H1E3	Q9H1E3 [70-87]	Q9H1E3 3xTMT6plex [K	0,01	-0,39	0,12
[K],VSSSPQK,[E]	1xTMT6plex [K7];1xTM	Q7Z5J4	Q7Z5J4 [1189-1195]	Q7Z5J4 1xTMT6plex [K	0,04	-0,39	0,16
[K],SPEKIEEVLSPEGSPSK,[S]	2xTMT6plex [K4; K17];1	Q9UEY8	Q9UEY8 [664-680]	Q9UEY8 2xTMT6plex [I	0,00	-0,39	0,11
[K],STNSPPPK,[S]	1xTMT6plex [K8];1xTM	P35711	P35711 [379-386]	P35711 1xTMT6plex [K	0,02	-0,39	0,13
[R],LAAAASPHSGGR,[A]	1xTMT6plex [N-Term];1	O95613	O95613 [3269-3280]	O95613 1xTMT6plex [N	0,01	-0,39	0,12
[R],LKATVTPSPVK,[G]	2xTMT6plex [K2; K11];1	Q9H1E3	Q9H1E3 [174-184]	Q9H1E3 2xTMT6plex [K	0,00	-0,39	0,11
[K],VKAQTPPGPSLSGSK,[S]	2xTMT6plex [K2; K15];1	Q9UQ35	Q9UQ35 [999-1013]	Q9UQ35 2xTMT6plex [I	0,00	-0,39	0,11
[R],SLSPLIK,[F]	1xTMT6plex [K7];1xTM	Q7Z4H7	Q7Z4H7 [906-912]	Q7Z4H7 1xTMT6plex [K	0,00	-0,39	0,12
[R],KPGSAGGPK,[V]	2xTMT6plex [K1; K9];1x	O75122	O75122 [44-52]	O75122 2xTMT6plex [K	0,02	-0,39	0,13
[R],APPPPSSPPPGGAPDGSEI	1xTMT6plex [K30];1xTM	O00459	O00459 [257-286]	O00459 1xTMT6plex [K	0,00	-0,39	0,12
[R],SFSEPFGR,[D]	1xTMT6plex [N-Term];1	P58340	P58340 [32-39]	P58340 1xTMT6plex [N	0,00	-0,39	0,11
[R],APSHMSSSHSFPQLAR,[N]	1xOxidation [M5];1xTM	Q9Y2J4	Q9Y2J4 [174-189]	Q9Y2J4 1xOxidation [M	0,02	-0,39	0,13
[R],RLDSCGKPGELGLPHPLST	1xCarbamidomethyl [C5	Q96GZ6	Q96GZ6 [10-41]	Q96GZ6 1xCarbamidon	0,03	-0,39	0,13
[R],AWAGSPK,[G]	1xTMT6plex [K7];1xTM	O43175	O43175 [345-351]	O43175 1xTMT6plex [K	0,02	-0,39	0,13
[K],KGSCNLSR,[V]	1xCarbamidomethyl [C4	Q06210	Q06210 [251-258]	Q06210 1xCarbamidom	0,04	-0,39	0,16
[R],SFSMQDLTTIR,[G]	1xTMT6plex [N-Term];1	Q9H902	Q9H902 [150-160]	Q9H902 1xTMT6plex [N	0,05	-0,39	0,16
[R],SLLSPLHR,[S]	1xTMT6plex [N-Term];1	Q9BY84	Q9BY84 [498-505]	Q9BY84 1xTMT6plex [N	0,03	-0,39	0,14
[R],AHLQTHSDVK,[K]	1xTMT6plex [K10];1xTM	O43623	O43623 [230-239]	O43623 1xTMT6plex [K	0,00	-0,39	0,11
[R],SSSDEQGLSYSSLK,[N]	1xTMT6plex [K14];1xTM	Q9NPH3	Q9NPH3 [555-568]	Q9NPH3 1xTMT6plex [I	0,00	-0,39	0,12
[R],AHTPLNTPDPSTK,[L]	1xTMT6plex [K13];1xTM	Q9BYW2	Q9BYW2 [1851-1863]	Q9BYW2 1xTMT6plex [	0,01	-0,39	0,12
[R],LRSGGTEGLLAEK,[L]	1xTMT6plex [K13];1xTM	Q9UHB9	Q9UHB9 [265-277]	Q9UHB9 1xTMT6plex [I	0,00	-0,39	0,12
[K],SKPLTSSR,[S]	1xTMT6plex [K2];1xTM	Q96RU2	Q96RU2 [512-519]	Q96RU2 1xTMT6plex [K	0,01	-0,39	0,12
[R],SPLSGIPVR,[T]	1xTMT6plex [N-Term];1	Q5M775	Q5M775 [847-855]	Q5M775 1xTMT6plex [N	0,01	-0,39	0,12
[R],SSEDVSTHAATK,[A]	1xTMT6plex [K12];1xTM	O14492	O14492 [117-128]	O14492 1xTMT6plex [K	0,00	-0,39	0,11
[R],SGASEANLIVAK,[S]	1xTMT6plex [K12];1xTM	P46013	P46013 [648-659]	P46013 1xTMT6plex [K	0,04	-0,39	0,15
[R],KAQVSPQS,[-]	1xTMT6plex [K1];1xTM	Q8IYB3	Q8IYB3 [897-904]	Q8IYB3 1xTMT6plex [K	0,03	-0,39	0,14
[R],AHSLGGLDPAFTSTEDLNC	1xCarbamidomethyl [C1	O60292	O60292 [389-408]	O60292 1xCarbamidom	0,01	-0,39	0,12
[R],RPSPLAPRPPPSR,[W]	1xTMT6plex [N-Term];1	Q9UMN6	Q9UMN6 [1915-1927]	Q9UMN6 1xTMT6plex [	0,01	-0,39	0,12
[R],GPKPEPPGSGSPAPPR,[R]	1xTMT6plex [K3];1xTM	Q9UKJ3	Q9UKJ3 [730-745]	Q9UKJ3 1xTMT6plex [K	0,01	-0,39	0,12
[R],KATGPPVSELITK,[A]	2xTMT6plex [K1; K13];1	P16401	P16401 [37-49]	P16401 2xTMT6plex [K	0,01	-0,39	0,12
[R],NRTPSDVKELVLDNSR,[S]	1xTMT6plex [K8];1xTM	P39687	P39687 [13-28]	P39687 1xTMT6plex [K	0,00	-0,39	0,11
[R],LNHSPPQSSSR,[Y]	1xTMT6plex [N-Term];1	Q5T8P6	Q5T8P6 [124-134]	Q5T8P6 1xTMT6plex [N	0,01	-0,39	0,12
[R],YSHSGSSSPDTK,[V]	1xTMT6plex [K12];1xTM	Q9UQ35	Q9UQ35 [967-978]	Q9UQ35 1xTMT6plex [I	0,03	-0,39	0,13
[K],ASSTSLTSTQPTK,[T]	1xTMT6plex [K13];1xTM	P35658	P35658 [1352-1364]	P35658 1xTMT6plex [K	0,04	-0,39	0,15
[K],ASNTSTPTK,[G]	1xTMT6plex [K9];1xTM	Q14004	Q14004 [489-497]	Q14004 1xTMT6plex [K	0,03	-0,39	0,14
[K],RHPSTLPVISDAR,[S]	1xTMT6plex [N-Term];1	Q92558	Q92558 [486-498]	Q92558 1xTMT6plex [N	0,04	-0,39	0,16
[R],KYSASSGGLCEEATAAK,[V	1xCarbamidomethyl [C1	Q9UHV7	Q9UHV7 [393-409]	Q9UHV7 1xCarbamidor	0,02	-0,39	0,13
[R],SRTSPAPWK,[R]	1xTMT6plex [K9];1xTM	Q9UQ35	Q9UQ35 [1854-1862]	Q9UQ35 1xTMT6plex [I	0,01	-0,39	0,12
[R],GHPHSASSPSER,[V]	1xTMT6plex [N-Term];1	Q15003	Q15003 [21-32]	Q15003 1xTMT6plex [N	0,02	-0,39	0,12
[R],LSSASTGKPPLSVEDDFEK	2xTMT6plex [K8; K19];1	O75152	O75152 [757-775]	O75152 2xTMT6plex [K	0,00	-0,39	0,11
[R],KGSSPSR,[S]	1xTMT6plex [K1];1xTM	Q8IYB3	Q8IYB3 [662-668]	Q8IYB3 1xTMT6plex [K	0,01	-0,39	0,12
[R],SPRPTGSDLLPGAPILSLR,[	1xTMT6plex [N-Term];1	Q8N1F8	Q8N1F8 [599-617]	Q8N1F8 1xTMT6plex [N	0,01	-0,39	0,12
[R],SPGLSPSSPSPEFLGLR,[S]	1xTMT6plex [N-Term];1	Q86X51	Q86X51 [443-459]	Q86X51 1xTMT6plex [N	0,00	-0,39	0,12
[K],DRQSPPPAK,[R]	1xTMT6plex [K9];1xTM	Q86VM9	Q86VM9 [839-847]	Q86VM9 1xTMT6plex [I	0,01	-0,39	0,12
[R],SRSSSSPPPKQK,[S]	2xTMT6plex [K10; K12]	Q9UQ35	Q9UQ35 [815-826]	Q9UQ35 2xTMT6plex [I	0,01	-0,39	0,12
[R],TPSSPHK,[K]	1xTMT6plex [K7];1xTM	Q9C0A6	Q9C0A6 [1122-1128]	Q9C0A6 1xTMT6plex [K	0,02	-0,39	0,13
[R],YSPSQNSPIHHIPSR,[R]	1xTMT6plex [N-Term];1	Q9NYF8	Q9NYF8 [284-298]	Q9NYF8 1xTMT6plex [N	0,00	-0,39	0,12
[R],DLSTPGLEK,[D]	1xTMT6plex [K9];1xTM	P46821	P46821 [1850-1858]	P46821 1xTMT6plex [K	0,04	-0,39	0,15
[R],KVSGDSSHTETTAEEVPED	2xTMT6plex [K1; K23];1	Q6W2J9	Q6W2J9 [1137-1159]	Q6W2J9 2xTMT6plex [I	0,01	-0,39	0,12

[R],RLSLPGLLSQVSPR,[L]	1xTMT6plex [N-Term];2	Q96Q42	Q96Q42 [481-494]	Q96Q42 1xTMT6plex [N	0,03	-0,39	0,14
[K],TAGYYPNPPLVLSSDETLIS	1xPhospho [T1];1xTMT	Q5TFE4	Q5TFE4 [435-455]	Q5TFE4 1xPhospho [T4	0,01	-0,39	0,12
[K],TAGYYPNPPLVLSSDETLIS	1xTMT6plex [K21];1xTM	Q5TFE4	Q5TFE4 [435-455]	Q5TFE4 1xTMT6plex [K	0,01	-0,39	0,12
[R],SVFWASSPYR,[R]	1xTMT6plex [N-Term];1	O15027	O15027 [23-32]	O15027 1xTMT6plex [N	0,03	-0,39	0,14
[R],LPNGEPSPDPGGKGTPR,[N	1xTMT6plex [K13];1xTM	Q9Y3Q8	Q9Y3Q8 [43-59]	Q9Y3Q8 1xTMT6plex [K	0,03	-0,39	0,14
[R],IDFTPVSPAPSPTR,[G]	1xTMT6plex [N-Term];2	Q7Z309	Q7Z309 [109-122]	Q7Z309 1xTMT6plex [N	0,02	-0,39	0,13
[R],QGSPVAAGAPAKQQQVDII	1xTMT6plex [K12];1xTM	Q9NZI8	Q9NZI8 [179-199]	Q9NZI8 1xTMT6plex [K	0,00	-0,39	0,12
[K],VGSTENIKHQPGGGR,[A]	1xTMT6plex [K8];1xTM	P27816	P27816 [939-953]	P27816 1xTMT6plex [K	0,00	-0,39	0,11
[R],AAVSPQK,[R]	1xTMT6plex [K7];1xTM	Q9HCU9	Q9HCU9 [234-240]	Q9HCU9 1xTMT6plex [	0,03	-0,39	0,14
[R],SLESVLSLGPRPTGGGSSF	1xTMT6plex [N-Term];1	Q13470	Q13470 [502-524]	Q13470 1xTMT6plex [N	0,04	-0,39	0,15
[K],TLVITSTPASPNR,[E]	1xTMT6plex [N-Term];1	Q8TBN0	Q8TBN0 [159-171]	Q8TBN0 1xTMT6plex [N	0,02	-0,39	0,13
[R],KLTPDHNK,[N]	2xTMT6plex [K1; K8];1x	Q8NDD1	Q8NDD1 [140-147]	Q8NDD1 2xTMT6plex [	0,04	-0,38	0,15
[K],WHASLYPASGR,[S]	1xTMT6plex [N-Term];1	Q9NRA8	Q9NRA8 [66-76]	Q9NRA8 1xTMT6plex [N	0,02	-0,38	0,12
[R],RDSDSFLNIFPEK,[Q]	1xTMT6plex [K13];1xTM	O94854	O94854 [664-676]	O94854 1xTMT6plex [K	0,01	-0,38	0,12
[R],KSREDLSAQPVQTK,[F]	2xTMT6plex [K1; K14];1	Q07157	Q07157 [616-629]	Q07157 2xTMT6plex [K	0,02	-0,38	0,13
[K],SAPATGGVK,[K]	1xTMT6plex [K9];1xTM	Q5TEC6;	Q5TEC6 [29-37]; Q16695 [29-37]	Q5TEC6 1xTMT6plex [K	0,01	-0,38	0,12
[R],VIEGSLSPK,[E]	1xTMT6plex [K9];1xTM	Q8IX01	Q8IX01 [597-605]	Q8IX01 1xTMT6plex [K	0,02	-0,38	0,12
[R],SSAIGIENIQEVQEK,[R]	1xTMT6plex [K15];1xTM	P47736	P47736 [498-512]	P47736 1xTMT6plex [K	0,05	-0,38	0,16
[R],SPCALPLAECK,[E]	2xCarbamidomethyl [C3	Q13615	Q13615 [913-923]	Q13615 2xCarbamidom	0,02	-0,38	0,12
[R],SPPEKPK,[Q]	1xMethyl [K5];1xTMT6p	Q68DK2	Q68DK2 [703-709]	Q68DK2 1xMethyl [K70	0,02	-0,38	0,12
[K],LGDVSPTQIDVSQFGSFKE	2xTMT6plex [K18; K22]	P46821	P46821 [1497-1518]	P46821 2xTMT6plex [K	0,04	-0,38	0,15
[R],LTAGVPDTPTR,[L]	1xTMT6plex [N-Term];1	P16144	P16144 [1523-1533]	P16144 1xTMT6plex [N	0,05	-0,38	0,16
[R],RKSLVGTPYWMAPELISR,[	1xTMT6plex [K2];1xTM	O96013	O96013 [472-489]	O96013 1xTMT6plex [K	0,02	-0,38	0,13
[R],TTSFFLNSPEK,[E]	1xTMT6plex [K11];1xTM	Q8WYP5	Q8WYP5 [1276-1286]	Q8WYP5 1xTMT6plex [	0,00	-0,38	0,11
[K],SPLAGNK,[D]	1xTMT6plex [K7];1xTM	O75151	O75151 [625-631]	O75151 1xTMT6plex [K	0,04	-0,38	0,15
[R],TQTPPLGQTPQLGLK,[T]	1xTMT6plex [K15];1xTM	P78344	P78344 [506-520]	P78344 1xTMT6plex [K	0,01	-0,38	0,12
[R],SSDKPIR,[T]	1xTMT6plex [K4];1xTM	Q99961	Q99961 [291-297]	Q99961 1xTMT6plex [K	0,03	-0,38	0,13
[R],GPSLNPVLDYDHGSRSQES	1xTMT6plex [N-Term];1	P43243	P43243 [193-216]	P43243 1xTMT6plex [N	0,00	-0,38	0,12
[R],TLEPVRPPVVPNDYVPSPT	1xTMT6plex [N-Term];1	Q9NYB9	Q9NYB9 [200-219]	Q9NYB9 1xTMT6plex [N	0,00	-0,38	0,12
[R],TASFVCR,[Q]	1xCarbamidomethyl [C6	Q12872	Q12872 [215-221]	Q12872 1xCarbamidom	0,00	-0,38	0,12
[R],LNCSTSPEIFR,[K]	1xCarbamidomethyl [C3	Q9BRS8	Q9BRS8 [404-414]	Q9BRS8 1xCarbamidon	0,03	-0,38	0,13
[R],QSHSSSSPHPK,[V]	1xTMT6plex [K11];1xTM	Q9UQ35	Q9UQ35 [833-843]	Q9UQ35 1xTMT6plex [	0,03	-0,38	0,14
[K],SHTPSPTSPK,[S]	1xTMT6plex [K10];1xTM	P40123	P40123 [305-314]	P40123 1xTMT6plex [K	0,03	-0,38	0,13
[R],SVPPATPATPTSPATVDAA	1xTMT6plex [K25];1xTM	Q6NYC8	Q6NYC8 [479-503]	Q6NYC8 1xTMT6plex [	0,01	-0,38	0,12
[R],QTQSASSTLQK,[H]	1xTMT6plex [K11];1xTM	O95819	O95819 [839-849]	O95819 1xTMT6plex [K	0,00	-0,38	0,11
[K],ASSVSTKESPAR,[K]	1xTMT6plex [K7];1xTM	Q13428	Q13428 [225-236]	Q13428 1xTMT6plex [K	0,01	-0,38	0,12
[R],LPNNSSRPSTPTINVLESK,[	1xTMT6plex [K19];1xTM	Q15910	Q15910 [358-376]	Q15910 1xTMT6plex [K	0,00	-0,38	0,12
[R],RYSPSPPPKR,[R]	1xTMT6plex [K9];1xTM	Q8IYB3	Q8IYB3 [603-612]	Q8IYB3 1xTMT6plex [K	0,00	-0,38	0,11
[R],GPSLNPVLDYDHGSR,[S]	1xTMT6plex [N-Term];1	P43243	P43243 [193-207]	P43243 1xTMT6plex [N	0,01	-0,38	0,12
[K],TAAELLQSQGSQAGGSQTI	1xTMT6plex [K20];1xTM	Q14141	Q14141 [401-420]	Q14141 1xTMT6plex [K	0,02	-0,38	0,13
[R],TVPSTPTLVVPHR,[T]	1xTMT6plex [N-Term];1	P12270	P12270 [2133-2145]	P12270 1xTMT6plex [N	0,02	-0,38	0,13
[R],KRESESESDETPPAAPQLI	2xTMT6plex [K1; K20];1	O60832	O60832 [448-467]	O60832 2xTMT6plex [K	0,00	-0,38	0,11
[R],GELSPSFLNPPLPPSIDDR,[	1xTMT6plex [N-Term];1	P78559	P78559 [2446-2464]	P78559 1xTMT6plex [N	0,02	-0,38	0,13
[K],ADTTTPTPTAILAPGSPASP	1xTMT6plex [K27];1xTM	P25440	P25440 [283-309]	P25440 1xTMT6plex [K	0,00	-0,38	0,11
[R],KEDKPEGQSPVK,[A]	3xTMT6plex [K1; K4; K	Q9C0C9	Q9C0C9 [888-899]	Q9C0C9 3xTMT6plex [K	0,01	-0,38	0,12
[R],TGPLSTSSPSR,[R]	1xTMT6plex [N-Term];1	Q8IY92	Q8IY92 [1446-1456]	Q8IY92 1xTMT6plex [N	0,03	-0,38	0,13
[K],KLSPIIEDSR,[E]	1xTMT6plex [K1];1xTM	O60566	O60566 [668-677]	O60566 1xTMT6plex [K	0,03	-0,38	0,14
[R],RGSSGSVDETLFALPAASE	1xTMT6plex [N-Term];1	Q9NQC3	Q9NQC3 [179-201]	Q9NQC3 1xTMT6plex [	0,04	-0,38	0,15
[K],SLPSSPSSPSSPASR,[K]	1xTMT6plex [N-Term];1	Q9Y6H5	Q9Y6H5 [556-570]	Q9Y6H5 1xTMT6plex [N	0,03	-0,38	0,13
[R],KRDSDAGSSTPTTSTR,[S]	1xTMT6plex [K1];1xTM	P51532	P51532 [1414-1429]	P51532 1xTMT6plex [K	0,02	-0,38	0,12
[R],TLDFDPLLSPASPK,[R]	1xTMT6plex [K14];1xTM	Q53H80	Q53H80 [10-23]	Q53H80 1xTMT6plex [K	0,04	-0,38	0,15
[R],LKLSPSPSSR,[V]	1xTMT6plex [K2];1xTM	P20700; Q	P20700 [388-397]; Q03252 [402-411]	P20700 1xTMT6plex [K	0,01	-0,38	0,12
[R],LLASITPSPSGQSIIR,[T]	1xTMT6plex [N-Term];1	O43610	O43610 [99-114]	O43610 1xTMT6plex [N	0,02	-0,38	0,13
[K],TGGKEAASGTTPQK,[S]	2xTMT6plex [K4; K14];1	Q13428	Q13428 [1260-1273]	Q13428 2xTMT6plex [K	0,02	-0,37	0,12
[K],IRDSAGQK,[G]	1xTMT6plex [K8];1xTM	P52209	P52209 [254-261]	P52209 1xTMT6plex [K	0,03	-0,37	0,14
[R],SRTSPAPWK,[R]	1xTMT6plex [K9];1xTM	Q9UQ35	Q9UQ35 [1854-1862]	Q9UQ35 1xTMT6plex [	0,01	-0,37	0,12
[K],RPNTSPDR,[G]	1xTMT6plex [N-Term];1	Q86VM9	Q86VM9 [848-855]	Q86VM9 1xTMT6plex [N	0,04	-0,37	0,15

[R],SHSLSGAR,[D]	1xTMT6plex [N-Term];1	Q9Y519	Q9Y519 [386-393]	Q9Y519 1xTMT6plex [N	0,02	-0,37	0,13
[R],SYSVVASEYDK,[Q]	1xTMT6plex [K11];1xTM	O75592	O75592 [3476-3486]	O75592 1xTMT6plex [K	0,02	-0,37	0,13
[RC],QVSASELHTSGILGPETLF	1xTMT6plex [N-Term];1	P58107	P58107 [2716-2734]; [3247-3265]; [3	P58107 1xTMT6plex [N	0,01	-0,37	0,12
[K],LPEGSPTWLGISCSSYER,[	1xCarbamidomethyl [C1	Q13506	Q13506 [99-116]	Q13506 1xCarbamidom	0,01	-0,37	0,12
[K],KSPEIHR,[R]	1xTMT6plex [K1];1xTM	Q9Y2W1	Q9Y2W1 [671-677]	Q9Y2W1 1xTMT6plex [	0,04	-0,37	0,15
[R],AASVSSLHER,[I]	1xTMT6plex [N-Term];1	Q12756	Q12756 [414-423]	Q12756 1xTMT6plex [N	0,02	-0,37	0,12
[K],AGMSSNQSISSPVLDAVPR	1xTMT6plex [N-Term];1	Q9UQ35	Q9UQ35 [1394-1412]	Q9UQ35 1xTMT6plex [	0,01	-0,37	0,12
[K],LSPPVASGGIPHQSPPTK,[	1xTMT6plex [K18];1xTM	Q96T58	Q96T58 [2480-2497]	Q96T58 1xTMT6plex [K	0,01	-0,37	0,12
[K],GPAAPLTPGPQSPPTPLAP	1xTMT6plex [K23];1xTM	O75398	O75398 [165-187]	O75398 1xTMT6plex [K	0,03	-0,37	0,14
[K],QAGSHSNSFR,[L]	1xTMT6plex [N-Term];1	P18433	P18433 [182-191]	P18433 1xTMT6plex [N	0,03	-0,37	0,14
[K],IIQADFTK,[D]	1xTMT6plex [K8];1xTM	P37058	P37058 [102-109]	P37058 1xTMT6plex [K	0,04	-0,37	0,16
[R],NALGPGLSPELGPLPALR,[	1xTMT6plex [N-Term];1	Q8N1G4	Q8N1G4 [85-102]	Q8N1G4 1xTMT6plex [	0,03	-0,37	0,14
[K],HAEPSSSPSK,[V]	1xTMT6plex [K10];1xTM	Q92738	Q92738 [589-598]	Q92738 1xTMT6plex [K	0,02	-0,37	0,12
[R],LSCSELSLYQPLQNSSK,[E]	1xCarbamidomethyl [C3	Q12923	Q12923 [935-951]	Q12923 1xCarbamidom	0,01	-0,37	0,12
[R],KDSSSQTMPVEDK,[S]	2xTMT6plex [K1; K13];1	Q9ULV3	Q9ULV3 [196-208]	Q9ULV3 2xTMT6plex [	0,01	-0,37	0,12
[K],IMVGSTDDPSVFSLPDSK,[	1xTMT6plex [K18];1xTM	O43741	O43741 [35-52]	O43741 1xTMT6plex [K	0,03	-0,37	0,14
[R],RGSHPYIDFR,[I]	1xTMT6plex [N-Term];1	Q13946	Q13946 [82-91]	Q13946 1xTMT6plex [N	0,03	-0,37	0,14
[K],RPSLPSSPSPGLPK,[A]	1xTMT6plex [K14];1xTM	O43294	O43294 [135-148]	O43294 1xTMT6plex [K	0,00	-0,37	0,12
[R],FGSPVLGTPK,[E]	1xTMT6plex [K10];1xTM	Q96K83	Q96K83 [544-553]	Q96K83 1xTMT6plex [K	0,05	-0,37	0,16
[K],TVDGPSGK,[L]	1xTMT6plex [K8];1xTM	P04406	P04406 [187-194]	P04406 1xTMT6plex [K	0,04	-0,37	0,15
[R],SGVTNMSSPHK,[N]	1xTMT6plex [K11];1xTM	Q9NVG8	Q9NVG8 [177-187]	Q9NVG8 1xTMT6plex [	0,04	-0,37	0,16
[M],ASPVAAQAGK,[L]	1xTMT6plex [K10];1xTM	Q96I99	Q96I99 [2-11]	Q96I99 1xTMT6plex [K1	0,03	-0,37	0,14
[R],AVVVSPKEENK,[A]	1xMethyl [K7];1xTMT6p	O75909	O75909 [336-346]	O75909 1xMethyl [K342	0,01	-0,37	0,12
[K],LNPYPTPSPPHPLYPGR,[R	1xTMT6plex [N-Term];1	Q5JTD0	Q5JTD0 [313-329]	Q5JTD0 1xTMT6plex [N	0,02	-0,37	0,13
[R],TDSTSDGRPAWMR,[T]	1xTMT6plex [N-Term];1	Q14204	Q14204 [4366-4378]	Q14204 1xTMT6plex [N	0,01	-0,37	0,12
[R],IHLGSSPK,[K]	1xTMT6plex [K8];1xTM	Q9H410	Q9H410 [53-60]	Q9H410 1xTMT6plex [K	0,04	-0,37	0,15
[R],VSHSPPPK,[Q]	1xTMT6plex [K8];1xTM	Q8IYB3	Q8IYB3 [635-642]	Q8IYB3 1xTMT6plex [K	0,00	-0,37	0,12
[R],KRSPSPSPTPEAK,[K]	2xTMT6plex [K1; K13];1	Q8TAQ2	Q8TAQ2 [300-312]	Q8TAQ2 2xTMT6plex [	0,01	-0,37	0,12
[R],VIEGSLSPK,[E]	1xTMT6plex [K9];1xTM	Q8IX01	Q8IX01 [597-605]	Q8IX01 1xTMT6plex [K	0,02	-0,37	0,13
[R],NLSFNELYPSGTLK,[L]	1xTMT6plex [K14];1xTM	Q8WYP5	Q8WYP5 [1539-1552]	Q8WYP5 1xTMT6plex [	0,02	-0,37	0,13
[R],GPPPSWGR,[R]	1xTMT6plex [N-Term];1	P84103	P84103 [91-98]	P84103 1xTMT6plex [N	0,00	-0,37	0,11
[R],LETAESK,[E]	1xTMT6plex [K7];1xTM	Q9UQ35	Q9UQ35 [1159-1165]	Q9UQ35 1xTMT6plex [	0,01	-0,37	0,12
[K],NSPEDLGLSLTGDSCK,[L]	1xCarbamidomethyl [C1	Q12888	Q12888 [499-514]	Q12888 1xCarbamidom	0,02	-0,37	0,13
[K],MTSSPVGPGTASAGPSLPG	1xTMT6plex [N-Term];1	Q14686	Q14686 [1889-1915]	Q14686 1xTMT6plex [N	0,03	-0,37	0,14
[K],ESEVCPVPTNSPSPPPLPP	1xCarbamidomethyl [C5	Q9ULJ3	Q9ULJ3 [971-995]	Q9ULJ3 1xCarbamidom	0,04	-0,37	0,16
[K],HIAFTPESQR,[R]	1xTMT6plex [N-Term];1	Q5H9R7	Q5H9R7 [627-636]	Q5H9R7 1xTMT6plex [N	0,02	-0,37	0,13
[R],SVSPLLSTHVLGK,[E]	1xTMT6plex [K13];1xTM	Q96H12	Q96H12 [96-108]	Q96H12 1xTMT6plex [K	0,04	-0,36	0,15
[R],GISHASSSIVSLAR,[S]	1xTMT6plex [N-Term];2	Q6GQQ9	Q6GQQ9 [98-111]	Q6GQQ9 1xTMT6plex [	0,01	-0,36	0,12
[K],IFPGLSPVR,[I]	1xTMT6plex [N-Term];1	Q5VZK9	Q5VZK9 [117-125]	Q5VZK9 1xTMT6plex [N	0,02	-0,36	0,13
[R],YSPSPPPK,[R]	1xTMT6plex [K8];1xTM	Q8IYB3	Q8IYB3 [604-611]	Q8IYB3 1xTMT6plex [K	0,02	-0,36	0,13
[R],KNSTGSGHSAQELPTIR,[T]	1xTMT6plex [K1];1xTM	O94763	O94763 [370-386]	O94763 1xTMT6plex [K	0,02	-0,36	0,12
[R],CLAHSLVGTPNYIAPEVLLR	1xCarbamidomethyl [C1	O95835; (	O95835 [905-924]; Q9NRM7 [868-88	O95835 1xCarbamidom	0,01	-0,36	0,12
[R],KDTDDIESPK,[R]	2xTMT6plex [K1; K10];1	Q9UPQ0	Q9UPQ0 [162-171]	Q9UPQ0 2xTMT6plex [	0,04	-0,36	0,16
[R],SSMGSTAVATDVK,[K]	1xTMT6plex [K13];1xTM	Q6UXY1	Q6UXY1 [477-489]	Q6UXY1 1xTMT6plex [	0,04	-0,36	0,15
[R],AALAPAKESPR,[K]	1xTMT6plex [K7];1xTM	Q13428	Q13428 [898-908]	Q13428 1xTMT6plex [K	0,02	-0,36	0,12
[R],RSPPADAIPK,[S]	1xTMT6plex [K10];1xTM	P18754	P18754 [10-19]	P18754 1xTMT6plex [K	0,01	-0,36	0,12
[R],GCSPGESASGGLSK,[K]	1xCarbamidomethyl [C2	Q9H165	Q9H165 [606-619]	Q9H165 1xCarbamidom	0,03	-0,36	0,13
[R],LTFNHDGSSR,[Y]	1xTMT6plex [N-Term];1	Q9C0C2	Q9C0C2 [187-196]	Q9C0C2 1xTMT6plex [N	0,03	-0,36	0,13
[R],SISANQK,[Q]	1xTMT6plex [K7];1xTM	Q9P0L2	Q9P0L2 [412-418]	Q9P0L2 1xTMT6plex [K	0,03	-0,36	0,14
[K],NKGTFDGDK,[L]	2xTMT6plex [K2; K9];1x	Q14671	Q14671 [257-265]	Q14671 2xTMT6plex [K	0,01	-0,36	0,12
[R],LAISEDHVASVK,[K]	1xTMT6plex [K12];1xTM	Q9HC35	Q9HC35 [52-63]	Q9HC35 1xTMT6plex [	0,02	-0,36	0,13
[R],GRPGRPPSTNK,[K]	1xTMT6plex [K11];1xTM	Q8WVM7	Q8WVM7 [38-48]	Q8WVM7 1xTMT6plex	0,03	-0,36	0,14
[K],IEPIPGESPK,[M]	1xTMT6plex [K10];1xTM	O60841	O60841 [1161-1170]	O60841 1xTMT6plex [K	0,05	-0,36	0,16
[R],SRASPVSR,[R]	1xTMT6plex [N-Term];1	Q9UQ35	Q9UQ35 [1913-1920]	Q9UQ35 1xTMT6plex [	0,02	-0,36	0,12
[R],SPALPPVWDVSASAK,[G]	1xTMT6plex [K15];1xTM	P56179	P56179 [130-144]	P56179 1xTMT6plex [K	0,04	-0,36	0,15
[R],LGSTGAK,[M]	1xTMT6plex [K7];1xTM	Q01826	Q01826 [45-51]	Q01826 1xTMT6plex [K	0,02	-0,36	0,12
[R],SSLGSLQTPEAVTTR,[K]	1xTMT6plex [N-Term];1	Q7Z2W4	Q7Z2W4 [386-400]	Q7Z2W4 1xTMT6plex [	0,01	-0,36	0,12

[R],VSGSFPEDSSK,[E]	1xTMT6plex [K11];1xTM	O15400	O15400 [128-138]	O15400 1xTMT6plex [K	0,00	-0,36	0,12
[R],APQTSSSPPPVR,[R]	1xTMT6plex [N-Term];1	Q8IYB3	Q8IYB3 [690-701]	Q8IYB3 1xTMT6plex [N	0,01	-0,36	0,12
[R],FFGNSWAETYR,[N]	1xTMT6plex [N-Term];1	O43488	O43488 [251-261]	O43488 1xTMT6plex [N	0,05	-0,36	0,16
[K],GKSDSEDGEK,[E]	2xTMT6plex [K2; K10];1	O00203	O00203 [748-757]	O00203 2xTMT6plex [K	0,03	-0,36	0,14
[R],VSSSGLTDSLFILK,[E]	1xTMT6plex [K14];1xTM	Q6P3S1	Q6P3S1 [651-664]	Q6P3S1 1xTMT6plex [K	0,03	-0,36	0,14
[K],EKTPSPKEEDEEPESPPEK	3xTMT6plex [K2; K7; K	Q9H1E3	Q9H1E3 [200-218]	Q9H1E3 3xTMT6plex [K	0,04	-0,36	0,15
[R],HSNLMLEDLDK,[S]	1xOxidation [M5];1xTMT	P11171	P11171 [663-673]	P11171 1xOxidation [M	0,05	-0,36	0,16
[R],QSHSGSISPYPK,[V]	1xTMT6plex [K12];1xTM	Q9UQ35	Q9UQ35 [987-998]	Q9UQ35 1xTMT6plex [I	0,01	-0,36	0,12
[R],LSPGSGGPEAQTAGPVTPA	1xTMT6plex [N-Term];1	O95613	O95613 [2351-2374]	O95613 1xTMT6plex [N	0,02	-0,36	0,12
[K],LTSSCPDLPSQTDK,[K]	1xCarbamidomethyl [C5	P56962	P56962 [286-299]	P56962 1xCarbamidom	0,04	-0,36	0,15
[R],LGAPENSGISTLER,[G]	1xTMT6plex [N-Term];1	Q9Y4F1	Q9Y4F1 [14-27]	Q9Y4F1 1xTMT6plex [N	0,03	-0,36	0,14
[R],ALTVPELTQQVFDAK,[N]	1xTMT6plex [K15];1xTM	P07437	P07437 [283-297]	P07437 1xTMT6plex [K	0,03	-0,36	0,14
[R],GDGGSTTGLSATPPASLPG	1xTMT6plex [K25];1xTM	P15056	P15056 [390-414]	P15056 1xTMT6plex [K	0,04	-0,36	0,15
[R],QSSTDSSGGHPGPR,[L]	1xTMT6plex [N-Term];1	Q9GZV5	Q9GZV5 [64-77]	Q9GZV5 1xTMT6plex [I	0,03	-0,36	0,14
[R],SSSSPPPK,[Q]	1xTMT6plex [K8];1xTM	Q9UQ35	Q9UQ35 [817-824]	Q9UQ35 1xTMT6plex [I	0,02	-0,36	0,13
[R],YGRPPDSHHSR,[R]	1xTMT6plex [N-Term];1	Q01130	Q01130 [92-102]	Q01130 1xTMT6plex [N	0,02	-0,36	0,13
[K],LYGSAGPPPTGEEDTAEKD	1xTMT6plex [K18];1xTM	P11021	P11021 [634-654]	P11021 1xTMT6plex [K	0,05	-0,36	0,16
[R],SSEDVSTHAATK,[A]	1xTMT6plex [K12];1xTM	O14492	O14492 [117-128]	O14492 1xTMT6plex [K	0,00	-0,36	0,12
[R],KPSGDSQPSSPR,[Y]	1xTMT6plex [K1];1xTM	Q9C0H5	Q9C0H5 [294-305]	Q9C0H5 1xTMT6plex [K	0,00	-0,36	0,12
[R],GAQAASPAK,[G]	1xTMT6plex [K9];1xTM	P18031	P18031 [381-389]	P18031 1xTMT6plex [K	0,04	-0,36	0,16
[K],RASFAEKTAQLER,[S]	1xTMT6plex [K7];1xTM	Q15149	Q15149 [1719-1731]	Q15149 1xTMT6plex [K	0,02	-0,36	0,13
[R],NFSFMNPGMER,[L]	2xOxidation [M5; M9];1	Q04759	Q04759 [693-703]	Q04759 2xOxidation [M	0,01	-0,36	0,12
[R],LSTHSPFR,[T]	1xTMT6plex [N-Term];1	P11171	P11171 [708-715]	P11171 1xTMT6plex [N	0,02	-0,36	0,13
[R],LSPPHSPR,[D]	1xTMT6plex [N-Term];1	Q92974	Q92974 [955-962]	Q92974 1xTMT6plex [N	0,03	-0,36	0,14
[R],LQQQHSEQPPLQPSPVMT	1xTMT6plex [N-Term];1	Q5JTV8	Q5JTV8 [130-148]	Q5JTV8 1xTMT6plex [N	0,00	-0,36	0,11
[R],AHPAPSMGSLR,[N]	1xOxidation [M7];1xTMT	Q9P2F8	Q9P2F8 [1533-1543]	Q9P2F8 1xOxidation [M	0,01	-0,36	0,12
[R],SSTVTEAPIAVVTSR,[T]	1xTMT6plex [N-Term];1	Q8TD19	Q8TD19 [331-345]	Q8TD19 1xTMT6plex [N	0,03	-0,36	0,14
[R],SSGSLGHRPSQEMDK,[M]	1xOxidation [M13];1xTM	Q5SW79	Q5SW79 [463-477]	Q5SW79 1xOxidation [I	0,01	-0,36	0,12
[R],LGGSTSDPPSSQSFSFHR,	1xTMT6plex [N-Term];1	Q13884	Q13884 [222-239]	Q13884 1xTMT6plex [N	0,02	-0,36	0,13
[K],GFQKAMVKTMSAALK,[I]	1xMethyl [K8];2xTMT6p	P11182	P11182 [258-272]	P11182 1xMethyl [K265	0,00	-0,36	0,12
[K],GFQKAMVKTMSAALK,[I]	1xMethyl [K4];2xTMT6p	P11182	P11182 [258-272]	P11182 1xMethyl [K261	0,00	-0,36	0,12
[R],SKLSPSPSLR,[K]	1xTMT6plex [K2];1xTM	Q5T200	Q5T200 [204-213]	Q5T200 1xTMT6plex [K	0,02	-0,35	0,12
[R],IESLSSQLSNLQK,[E]	1xTMT6plex [K13];1xTM	P20700	P20700 [300-312]	P20700 1xTMT6plex [K	0,03	-0,35	0,14
[R],EQDSPPMKPSALDAIR,[E]	1xTMT6plex [K8];1xTM	Q16799	Q16799 [484-499]	Q16799 1xTMT6plex [K	0,04	-0,35	0,15
[K],TPSKPPAQLSPSVPK,[R]	2xTMT6plex [K4; K15];1	P18887	P18887 [257-271]	P18887 2xTMT6plex [K	0,04	-0,35	0,15
[R],TGPLSTSSPSR,[R]	1xTMT6plex [N-Term];1	Q8IY92	Q8IY92 [1446-1456]	Q8IY92 1xTMT6plex [N	0,01	-0,35	0,12
[K],ASSWPHR,[R]	1xTMT6plex [N-Term];1	Q12774	Q12774 [1002-1008]	Q12774 1xTMT6plex [N	0,03	-0,35	0,14
[R],VEQALSEVASSLQSSAPK,[	1xTMT6plex [K18];1xTM	Q86TV6	Q86TV6 [668-685]	Q86TV6 1xTMT6plex [K	0,04	-0,35	0,16
[R],YSHSGSSSPDTK,[V]	1xTMT6plex [K12];1xTM	Q9UQ35	Q9UQ35 [967-978]	Q9UQ35 1xTMT6plex [I	0,05	-0,35	0,16
[R],RKSEPAVGPPR,[G]	1xTMT6plex [K2];1xTM	O94875	O94875 [257-267]	O94875 1xTMT6plex [K	0,03	-0,35	0,14
[R],AISTGSLASSTLNK,[L]	1xTMT6plex [K14];1xTM	Q12923	Q12923 [906-919]	Q12923 1xTMT6plex [K	0,01	-0,35	0,12
[K],LPSSETHPEESMYK,[R]	1xOxidation [M12];1xTM	Q8TBP0	Q8TBP0 [381-394]	Q8TBP0 1xOxidation [M	0,03	-0,35	0,14
[R],KSEDFVK,[D]	2xTMT6plex [K1; K7];1x	Q9H9J4	Q9H9J4 [1246-1252]	Q9H9J4 2xTMT6plex [K	0,01	-0,35	0,12
[R],GPKPEPPGSGSPAPPR,[R]	1xTMT6plex [K3];1xTM	Q9UKJ3	Q9UKJ3 [730-745]	Q9UKJ3 1xTMT6plex [K	0,04	-0,35	0,15
[K],SLTLTPTR,[S]	1xTMT6plex [N-Term];1	Q3V6T2	Q3V6T2 [1417-1424]	Q3V6T2 1xTMT6plex [N	0,00	-0,35	0,12
[R],MKESSPSGSK,[S]	2xTMT6plex [K2; K10];1	Q9NX63	Q9NX63 [36-45]	Q9NX63 2xTMT6plex [K	0,03	-0,35	0,14
[K],QEPAPPSLSPAVSVK,[S]	1xTMT6plex [K15];1xTM	Q9HCS4	Q9HCS4 [317-331]	Q9HCS4 1xTMT6plex [I	0,02	-0,35	0,13
[K],GKSLDSEPSVPSAAKPPSP	3xTMT6plex [K2; K15; K	Q7Z3K3	Q7Z3K3 [408-428]	Q7Z3K3 3xTMT6plex [K	0,00	-0,35	0,11
[R],RVSFADPIYQAGLADDIDR,	1xTMT6plex [N-Term];1	Q5UIP0	Q5UIP0 [2203-2221]	Q5UIP0 1xTMT6plex [N	0,03	-0,35	0,14
[R],SGSMDPSGAHPSVR,[Q]	1xTMT6plex [N-Term];1	Q07666	Q07666 [18-31]	Q07666 1xTMT6plex [N	0,02	-0,35	0,12
[K],LSGLSFK,[R]	1xTMT6plex [K7];1xTM	P49006	P49006 [100-106]	P49006 1xTMT6plex [K	0,05	-0,35	0,16
[R],RGSLSNAGDPEIVK,[S]	1xTMT6plex [K14];1xTM	O43847	O43847 [92-105]	O43847 1xTMT6plex [K	0,05	-0,35	0,17
[K],VSHVSTGGGASLELLEGK,[	1xTMT6plex [K18];1xTM	P00558	P00558 [389-406]	P00558 1xTMT6plex [K	0,04	-0,35	0,15
[R],IQPQPPDEDGDHSDKEDE(	2xTMT6plex [K15; K26]	Q96AT1	Q96AT1 [38-63]	Q96AT1 2xTMT6plex [K	0,02	-0,35	0,13
[R],GSGGLFSPSTAHVPDGALC	1xTMT6plex [N-Term];1	Q9C0C2	Q9C0C2 [1023-1043]	Q9C0C2 1xTMT6plex [N	0,00	-0,35	0,12
[R],LSSGLVSPAPSFYSK,[E]	1xTMT6plex [K15];1xTM	Q15911	Q15911 [2890-2904]	Q15911 1xTMT6plex [K	0,03	-0,35	0,13
[R],VGPGDDRHSTK,[S]	1xTMT6plex [K11];1xTM	Q9Y4F5	Q9Y4F5 [329-339]	Q9Y4F5 1xTMT6plex [K	0,02	-0,35	0,13

[K],TGMGSGSAGK,[E]	1xTMT6plex [K10];1xTM	Q86YS7	Q86YS7 [317-326]	Q86YS7 1xTMT6plex [K	0,04	-0,35	0,15
[R],TPSLPTPPTR,[E]	1xTMT6plex [N-Term];1	P10636	P10636 [529-538]	P10636 1xTMT6plex [N	0,02	-0,35	0,13
[R],AHSSLGPGRPR,[S]	1xTMT6plex [N-Term];1	Q9NRR6	Q9NRR6 [230-240]	Q9NRR6 1xTMT6plex [	0,01	-0,35	0,12
[R],NVSESPNRK,[N]	1xTMT6plex [K9];1xTM	Q13427	Q13427 [411-419]	Q13427 1xTMT6plex [K	0,02	-0,35	0,12
[R],APSAPVGK,[E]	1xTMT6plex [K8];1xTM	P43119	P43119 [335-342]	P43119 1xTMT6plex [K	0,03	-0,35	0,14
[K],LNGPQDHSHLLYSTIPR,[M]	1xTMT6plex [N-Term];1	O60716	O60716 [85-101]	O60716 1xTMT6plex [N	0,03	-0,35	0,14
[K],SQTPPRR,[S]	1xTMT6plex [N-Term];1	Q9UQ35	Q9UQ35 [790-796]	Q9UQ35 1xTMT6plex [I	0,03	-0,35	0,13
[R],ALASAAGQTPK,[V]	1xTMT6plex [K11];1xTM	Q14209	Q14209 [7-17]	Q14209 1xTMT6plex [K	0,05	-0,35	0,16
[R],SPQQTVPYVVPLSPK,[L]	1xTMT6plex [K15];1xTM	Q9UQR0	Q9UQR0 [499-513]	Q9UQR0 1xTMT6plex [	0,02	-0,35	0,13
[R],SPSPSPTPEAKK,[K]	2xTMT6plex [K11; K12]	Q8TAQ2	Q8TAQ2 [302-313]	Q8TAQ2 2xTMT6plex [I	0,00	-0,35	0,11
[K],TISTSDPAEVLVK,[N]	1xTMT6plex [K13];1xTM	Q86UE4	Q86UE4 [492-504]	Q86UE4 1xTMT6plex [K	0,00	-0,35	0,12
[R],SSSLIQLTSQNSSPNQQR,[	1xTMT6plex [N-Term];1	O95639	O95639 [200-217]	O95639 1xTMT6plex [N	0,03	-0,35	0,14
[R],LSDKSSTSETSLGEER,[A]	1xTMT6plex [K4];1xTM	Q562E7	Q562E7 [1108-1123]	Q562E7 1xTMT6plex [K	0,01	-0,35	0,12
[R],SSNASDKDSDLQK,[T]	2xTMT6plex [K7; K13];1	Q9UKA4	Q9UKA4 [284-296]	Q9UKA4 2xTMT6plex [I	0,01	-0,35	0,12
[R],RGSAVATSHFEVGNTCPSE	1xCarbamidomethyl [C1	Q92576	Q92576 [1756-1778]	Q92576 1xCarbamidom	0,02	-0,35	0,13
[R],TLTPASSPVSSPSK,[H]	1xTMT6plex [K14];1xTM	Q9UM11	Q9UM11 [30-43]	Q9UM11 1xTMT6plex [I	0,01	-0,35	0,12
[R],SPYSPVLR,[R]	1xTMT6plex [N-Term];1	Q14004	Q14004 [397-404]	Q14004 1xTMT6plex [N	0,00	-0,35	0,12
[K],AEPYVASEYKTVHEELTK,[S	2xTMT6plex [K10; K18]	Q9BZ29	Q9BZ29 [921-938]	Q9BZ29 2xTMT6plex [K	0,03	-0,35	0,14
[R],ERDQRPSSPIR,[H]	1xTMT6plex [N-Term];1	Q5T200	Q5T200 [636-646]	Q5T200 1xTMT6plex [N	0,01	-0,35	0,12
[R],LHPGTSCPR,[L]	1xCarbamidomethyl [C7	Q6ZN04	Q6ZN04 [432-440]	Q6ZN04 1xCarbamidom	0,04	-0,35	0,15
[R],KNNPSPPPDSDLER,[V]	1xTMT6plex [K1];1xTM	O95677	O95677 [357-370]	O95677 1xTMT6plex [K	0,02	-0,35	0,12
[R],TPSPPPPIPEDIALGK,[K]	1xTMT6plex [K16];1xTM	Q5T200	Q5T200 [263-278]	Q5T200 1xTMT6plex [K	0,01	-0,35	0,12
[R],EEASLLSHSPGTSNQSQPC	1xCarbamidomethyl [C1	Q9H4Z3	Q9H4Z3 [11-35]	Q9H4Z3 1xCarbamidom	0,00	-0,35	0,12
[R],THSTSSSLGSGESPFSR,[S	1xTMT6plex [N-Term];1	Q9UGV2	Q9UGV2 [329-345]	Q9UGV2 1xTMT6plex [	0,00	-0,35	0,12
[R],EATGLSPQAAQEKDGIVIVK	2xTMT6plex [K13; K20]	P17029	P17029 [8-27]	P17029 2xTMT6plex [K	0,03	-0,35	0,14
[K],CSPGSPTDPNATLSK,[D]	1xCarbamidomethyl [C1	Q9P0K8	Q9P0K8 [42-56]	Q9P0K8 1xCarbamidom	0,04	-0,35	0,15
[R],HSCSGSSPPR,[V]	1xCarbamidomethyl [C3	Q9UQ35	Q9UQ35 [889-898]	Q9UQ35 1xCarbamidom	0,02	-0,35	0,13
[K],DLSTSPKPSPIPSPVLGR,[K	1xTMT6plex [K7];1xTM	Q8NDI1	Q8NDI1 [424-441]	Q8NDI1 1xTMT6plex [K	0,01	-0,35	0,12
[K],GKPESEGEAK,[E]	2xTMT6plex [K2; K10];1	Q96EI5	Q96EI5 [63-72]	Q96EI5 2xTMT6plex [K	0,05	-0,35	0,16
[K],TGSGSPFAGNSPAR,[E]	1xTMT6plex [N-Term];1	Q9UPT8	Q9UPT8 [1265-1278]	Q9UPT8 1xTMT6plex [N	0,01	-0,35	0,12
[R],EVRPSPSK,[T]	1xTMT6plex [K8];1xTM	Q92833	Q92833 [327-334]	Q92833 1xTMT6plex [K	0,02	-0,35	0,13
[R],QLHLEGASLELSDDDTESK	1xTMT6plex [K19];1xTM	P35580	P35580 [1945-1963]	P35580 1xTMT6plex [K	0,02	-0,35	0,13
[R],LAGGQTSQPTTPLTSPQR,[	1xTMT6plex [N-Term];1	O95155	O95155 [17-34]	O95155 1xTMT6plex [N	0,04	-0,35	0,15
[K],ENKEPSPK,[A]	2xTMT6plex [K3; K8];1x	B2RTY4	B2RTY4 [1824-1831]	B2RTY4 2xTMT6plex [K	0,02	-0,35	0,12
[R],LFAPPSPETGEAK,[R]	1xTMT6plex [K13];1xTM	Q9NQV8	Q9NQV8 [370-382]	Q9NQV8 1xTMT6plex [	0,02	-0,35	0,12
[R],TSDVGGYYYEK,[I]	1xTMT6plex [K11];1xTM	P46821	P46821 [1898-1908]	P46821 1xTMT6plex [K	0,03	-0,35	0,13
[R],RDSAPYGEYGSWYK,[A]	1xTMT6plex [K14];1xTM	Q9H0B6	Q9H0B6 [426-439]	Q9H0B6 1xTMT6plex [K	0,01	-0,35	0,12
[K],VNEEKDGPISPK,[K]	2xTMT6plex [K5; K12];1	Q8WWQ0	Q8WWQ0 [902-913]	Q8WWQ0 2xTMT6plex	0,01	-0,35	0,12
[R],SVIEPLPVTPTR,[D]	1xTMT6plex [N-Term];1	Q13153	Q13153 [204-215]	Q13153 1xTMT6plex [N	0,03	-0,35	0,14
[K],LVLPSNTPNVR,[R]	1xTMT6plex [N-Term];1	Q03188	Q03188 [728-738]	Q03188 1xTMT6plex [N	0,03	-0,34	0,13
[R],TCPSLSPTSPLNNK,[G]	1xCarbamidomethyl [C2	O75925	O75925 [480-493]	O75925 1xCarbamidom	0,02	-0,34	0,12
[K],SMPVLGSVSSVTK,[T]	1xOxidation [M2];1xTM	A0MZ66	A0MZ66 [506-518]	A0MZ66 1xOxidation [M	0,01	-0,34	0,12
[K],APKPDGPGGGPGGSHMG(	1xOxidation [M16];1xTM	P35637	P35637 [449-472]	P35637 1xOxidation [M	0,01	-0,34	0,12
[R],WDQTADQTPGATPK,[K]	1xTMT6plex [K14];1xTM	O75533	O75533 [200-213]	O75533 1xTMT6plex [K	0,03	-0,34	0,13
[K],KPAPGPHSSPPEEK,[G]	2xTMT6plex [K1; K14];1	O75182	O75182 [732-745]	O75182 2xTMT6plex [K	0,03	-0,34	0,13
[R],VSVSPGRTSGK,[V]	1xTMT6plex [K11];1xTM	Q8IYB3	Q8IYB3 [428-438]	Q8IYB3 1xTMT6plex [K	0,01	-0,34	0,12
[K],AAQPSPSTVPK,[T]	1xTMT6plex [K11];1xTM	P11308	P11308 [272-282]	P11308 1xTMT6plex [K	0,01	-0,34	0,12
[R],KADSEEERDDVSTLGSMLF	2xTMT6plex [K1; K21];1	P78364	P78364 [642-662]	P78364 2xTMT6plex [K	0,04	-0,34	0,15
[R],QVVQTPNTVLSTPFR,[T]	1xTMT6plex [N-Term];1	Q99459	Q99459 [400-414]	Q99459 1xTMT6plex [N	0,05	-0,34	0,17
[R],KVNTSPR,[R]	1xTMT6plex [K1];1xTM	Q8WZ74	Q8WZ74 [1457-1463]	Q8WZ74 1xTMT6plex [	0,01	-0,34	0,12
[K],LMHSSSLTNSSIPR,[F]	1xTMT6plex [N-Term];1	Q08499	Q08499 [370-383]	Q08499 1xTMT6plex [N	0,01	-0,34	0,12
[R],SPGRPTQGALGEQQDLSN	1xTMT6plex [K22];1xTM	P10071	P10071 [664-685]	P10071 1xTMT6plex [K	0,04	-0,34	0,16
[R],IPVEGPLSPSR,[G]	1xTMT6plex [N-Term];1	O00512	O00512 [680-690]	O00512 1xTMT6plex [N	0,01	-0,34	0,12
[R],VDSPSHGLVTSSLCIPSPAF	1xCarbamidomethyl [C1	Q9UER7	Q9UER7 [686-705]	Q9UER7 1xCarbamidom	0,00	-0,34	0,11
[R],SPSWYGIPR,[L]	1xTMT6plex [N-Term];1	P78347	P78347 [515-523]	P78347 1xTMT6plex [N	0,02	-0,34	0,12
[K],SPSLSPSPPSPLEK,[T]	1xTMT6plex [K14];1xTM	P46821	P46821 [1256-1269]	P46821 1xTMT6plex [K	0,04	-0,34	0,16
[K],SPSLSPSPPSPLEK,[T]	1xTMT6plex [K14];1xTM	P46821	P46821 [1256-1269]	P46821 1xTMT6plex [K	0,04	-0,34	0,16

[R],EGSPIPHDPEFGSK,[L]	1xTMT6plex [K14];1xTM	P85037	P85037 [443-456]	P85037 1xTMT6plex [K	0,02	-0,34	0,12
[R],TPVKPSSVEEEDSFFR,[Q]	1xTMT6plex [K4];1xTM	Q9UHB7	Q9UHB7 [674-689]	Q9UHB7 1xTMT6plex [	0,02	-0,34	0,13
[R],LSQPESAEK,[H]	1xTMT6plex [K9];1xTM	Q9UKV3	Q9UKV3 [709-717]	Q9UKV3 1xTMT6plex [	0,05	-0,34	0,16
[R],WDETPASQMGGSTPVLTP	1xTMT6plex [K20];1xTM	O75533	O75533 [338-357]	O75533 1xTMT6plex [K	0,02	-0,34	0,13
[R],SPGLSPSSPSPEFLGLR,[S]	1xTMT6plex [N-Term];1	Q86X51	Q86X51 [443-459]	Q86X51 1xTMT6plex [N	0,03	-0,34	0,14
[R],ELGTGEQPLSPTTATSPYG	1xTMT6plex [K31];1xTM	Q96NU1	Q96NU1 [631-661]	Q96NU1 1xTMT6plex [	0,05	-0,34	0,16
[R],ELGTGEQPLSPTTATSPYG	1xPhospho [T12];1xTM	Q96NU1	Q96NU1 [631-661]	Q96NU1 1xPhospho [T	0,05	-0,34	0,16
[R],TLSFGSDLNYATR,[E]	1xTMT6plex [N-Term];1	Q14152	Q14152 [490-502]	Q14152 1xTMT6plex [N	0,03	-0,34	0,14
[K],LTASPSDPK,[V]	1xTMT6plex [K9];1xTM	Q76L83	Q76L83 [392-400]	Q76L83 1xTMT6plex [K	0,02	-0,34	0,13
[R],VSHSPPPK,[Q]	1xTMT6plex [K8];1xTM	Q8IYB3	Q8IYB3 [635-642]	Q8IYB3 1xTMT6plex [K	0,00	-0,34	0,12
[R],NLHQSNFSLSGAQIDDNNP	1xTMT6plex [N-Term];1	Q14194	Q14194 [533-552]	Q14194 1xTMT6plex [N	0,01	-0,34	0,12
[R],TLEPVRPPVVPNDYVPSPT	1xTMT6plex [N-Term];1	Q9NYB9	Q9NYB9 [200-219]	Q9NYB9 1xTMT6plex [	0,01	-0,34	0,12
[K],NIPCAAVSPNR,[S]	1xCarbamidomethyl [C4	Q13118	Q13118 [199-209]	Q13118 1xCarbamidom	0,00	-0,34	0,12
[R],DSITPDIATKPGQPLFLDSIS	2xTMT6plex [K10; K23]	Q2TB10	Q2TB10 [316-338]	Q2TB10 2xTMT6plex [K	0,03	-0,34	0,14
[R],GLPNGPTHAFSSPSESPDS	1xTMT6plex [N-Term];1	Q9ULJ7	Q9ULJ7 [1152-1174]	Q9ULJ7 1xTMT6plex [N	0,00	-0,34	0,11
[K],VPSFTFTPTVTYQR,[G]	1xTMT6plex [N-Term];1	Q9Y6N7	Q9Y6N7 [938-951]	Q9Y6N7 1xTMT6plex [N	0,03	-0,34	0,14
[K],ILGTQEITARDSGVR,[E]	1xTMT6plex [N-Term];1	Q8N3D4	Q8N3D4 [723-737]	Q8N3D4 1xTMT6plex [N	0,01	-0,34	0,12
[R],GTPKPPGPPAQPPGPPNA	1xTMT6plex [K4];1xTM	Q8N4C8	Q8N4C8 [714-738]	Q8N4C8 1xTMT6plex [	0,03	-0,34	0,14
[R],SSSASSPPKK,[E]	2xTMT6plex [K9; K10];1	P51608	P51608 [355-364]	P51608 2xTMT6plex [K	0,02	-0,34	0,13
[R],NQSFCPTVNLDK,[L]	1xCarbamidomethyl [C5	P46776	P46776 [66-77]	P46776 1xCarbamidom	0,05	-0,34	0,16
[R],CADTRPGSEQPPLGGAAS	1xCarbamidomethyl [C1	Q96RY5	Q96RY5 [579-605]	Q96RY5 1xCarbamidon	0,01	-0,34	0,12
[R],HIISATSLSTSPTELGSR,[N]	1xTMT6plex [N-Term];1	Q5JSZ5	Q5JSZ5 [216-233]	Q5JSZ5 1xTMT6plex [N	0,04	-0,34	0,15
[K],NKSPAAVTEPETNK,[F]	2xTMT6plex [K2; K14];1	O75449	O75449 [168-181]	O75449 2xTMT6plex [K	0,00	-0,34	0,11
[R],DTSPSSGSAVSSSK,[V]	1xTMT6plex [K14];1xTM	Q8NEY8	Q8NEY8 [203-216]	Q8NEY8 1xTMT6plex [	0,01	-0,34	0,12
[R],GSGVFCANCLTTK,[T]	2xCarbamidomethyl [C6	Q9UHF7	Q9UHF7 [891-903]	Q9UHF7 2xCarbamidor	0,05	-0,34	0,16
[R],ECLVCTGCQTPLAGQQFTS	3xCarbamidomethyl [C2	Q13643	Q13643 [184-203]	Q13643 3xCarbamidom	0,02	-0,34	0,13
[K],KGWDAEGSPFR,[G]	1xTMT6plex [K1];1xTM	Q8IVD9	Q8IVD9 [333-343]	Q8IVD9 1xTMT6plex [K	0,01	-0,34	0,12
[R],VPASETSPGPPPMGPPPPS	1xTMT6plex [K21];1xTM	Q9HD15	Q9HD15 [63-83]	Q9HD15 1xTMT6plex [	0,03	-0,34	0,14
[K],EKLIPGPLSPVAR,[G]	1xTMT6plex [K2];1xTM	P48634	P48634 [1211-1223]	P48634 1xTMT6plex [K	0,02	-0,34	0,12
[R],KRTPSPSYQR,[T]	1xTMT6plex [K1];1xTM	Q5T200	Q5T200 [352-361]	Q5T200 1xTMT6plex [K	0,02	-0,34	0,12
[K],TKKDDSHSAEDSEDEK,[E]	3xTMT6plex [K2; K3; K	Q9H1E3	Q9H1E3 [68-83]	Q9H1E3 3xTMT6plex [	0,02	-0,34	0,13
[K],SVSDPVEDKK,[E]	2xTMT6plex [K9; K10];1	Q8NFC6	Q8NFC6 [2973-2982]	Q8NFC6 2xTMT6plex [	0,04	-0,34	0,15
[R],SHQATTLK,[R]	1xTMT6plex [K8];1xTM	Q9H2E6	Q9H2E6 [928-935]	Q9H2E6 1xTMT6plex [	0,00	-0,34	0,12
[R],SSTPSPSPLNLSSSR,[N]	1xTMT6plex [N-Term];1	P37275	P37275 [700-714]	P37275 1xTMT6plex [N	0,00	-0,34	0,11
[R],TVSEPSLSGLLQIK,[K]	1xTMT6plex [K14];1xTM	Q6N021	Q6N021 [97-110]	Q6N021 1xTMT6plex [K	0,02	-0,34	0,13
[K],GSSTPVKN,[-]	1xTMT6plex [K7];1xTM	O00399	O00399 [183-190]	O00399 1xTMT6plex [K	0,04	-0,34	0,15
[R],INPPSSGGTSSSPIK,[A]	1xTMT6plex [K15];1xTM	P14859	P14859 [437-451]	P14859 1xTMT6plex [K	0,00	-0,34	0,12
[R],SHSGPSSLPEAPLKPPGPL	2xPhospho [S1; S3];2x	Q08378	Q08378 [16-42]	Q08378 2xPhospho [S1	0,03	-0,34	0,14
[R],SHSGPSSLPEAPLKPPGPL	2xTMT6plex [K14; K27]	Q08378	Q08378 [16-42]	Q08378 2xTMT6plex [K	0,03	-0,34	0,14
[R],KGTQTSPVPR,[K]	1xTMT6plex [K1];1xTM	Q2M1K9	Q2M1K9 [1155-1164]	Q2M1K9 1xTMT6plex [	0,01	-0,34	0,12
[R],NSLLAGGDDDTMSVISGIS	1xTMT6plex [N-Term];1	Q8N3U4	Q8N3U4 [1046-1066]	Q8N3U4 1xTMT6plex [N	0,01	-0,34	0,12
[K],VQTTPSKPGGDR,[Y]	1xTMT6plex [K7];1xTM	Q12834	Q12834 [67-78]	Q12834 1xTMT6plex [K	0,01	-0,34	0,12
[R],SSMEMEGIFNHK,[D]	1xTMT6plex [K12];1xTM	O75962	O75962 [1723-1734]	O75962 1xTMT6plex [K	0,05	-0,34	0,16
[R],SHNNFVAILDLPEGEHQYK	1xTMT6plex [K19];1xTM	Q9Y478	Q9Y478 [108-126]	Q9Y478 1xTMT6plex [K	0,03	-0,33	0,14
[K],TSPAKQQAPPVR,[N]	1xTMT6plex [K5];1xTM	Q16555	Q16555 [521-532]	Q16555 1xTMT6plex [K	0,01	-0,33	0,12
[K],RKHSPSPPPPTPTESR,[K]	1xTMT6plex [K2];1xTM	Q92922	Q92922 [325-340]	Q92922 1xTMT6plex [K	0,00	-0,33	0,12
[R],SHVATCSK,[Y]	1xCarbamidomethyl [C6	Q9Y508	Q9Y508 [105-112]	Q9Y508 1xCarbamidom	0,02	-0,33	0,12
[R],SPSKPTLAYPESNSR,[A]	1xTMT6plex [K4];1xTM	Q86XR8	Q86XR8 [53-67]	Q86XR8 1xTMT6plex [	0,02	-0,33	0,13
[R],SPSKPDFLYK,[K]	2xTMT6plex [K4; K10];1	Q9P203	Q9P203 [1119-1128]	Q9P203 2xTMT6plex [K	0,04	-0,33	0,16
[R],TSSSSSFSDK,[K]	1xTMT6plex [K10];1xTM	Q03111	Q03111 [318-327]	Q03111 1xTMT6plex [K	0,02	-0,33	0,13
[R],TSSSSSFSDK,[K]	1xTMT6plex [K10];1xTM	Q03111	Q03111 [318-327]	Q03111 1xTMT6plex [K	0,02	-0,33	0,13
[R],DRNTLPK,[K]	1xTMT6plex [K7];1xTM	Q8NEY1	Q8NEY1 [939-945]	Q8NEY1 1xTMT6plex [	0,03	-0,33	0,14
[R],ASAGTPSLSAGVSPK,[R]	1xTMT6plex [K15];1xTM	Q8TEK3	Q8TEK3 [1092-1106]	Q8TEK3 1xTMT6plex [	0,03	-0,33	0,13
[R],EFTGSPPSSATK,[K]	1xTMT6plex [K12];1xTM	Q92609	Q92609 [550-561]	Q92609 1xTMT6plex [K	0,00	-0,33	0,12
[R],SPPQSPGPGK,[A]	1xTMT6plex [K10];1xTM	Q86VQ6	Q86VQ6 [4-13]	Q86VQ6 1xTMT6plex [	0,01	-0,33	0,12
[R],KRSPSPSPTPEAK,[K]	2xTMT6plex [K1; K13];1	Q8TAQ2	Q8TAQ2 [300-312]	Q8TAQ2 2xTMT6plex [	0,00	-0,33	0,12
[K],VATPPNQNQK,[Q]	1xTMT6plex [K10];1xTM	Q6NUQ4	Q6NUQ4 [95-104]	Q6NUQ4 1xTMT6plex [	0,04	-0,33	0,15

[R],SIGASPNPFSVHTATAVPS(	1xTMT6plex [K21];1xTM	P09884	P09884 [186-206]	P09884 1xTMT6plex [K	0,02	-0,33	0,13
[R],TEAVSPTVSQLSAVFENTD	1xTMT6plex [K28];1xTM	Q9ULJ8	Q9ULJ8 [195-222]	Q9ULJ8 1xTMT6plex [K	0,01	-0,33	0,12
[K],LDFNSPGGSSPVENSDCST	1xCarbamidomethyl [C1	Q9Y2F5	Q9Y2F5 [935-956]	Q9Y2F5 1xCarbamidom	0,01	-0,33	0,12
[K],TKKDDSHSAEDSEDEK,[E]	3xTMT6plex [K2; K3; K	Q9H1E3	Q9H1E3 [68-83]	Q9H1E3 3xTMT6plex [K	0,02	-0,33	0,13
[R],RSSLSSR,[G]	1xTMT6plex [N-Term];1	Q8WUM0	Q8WUM0 [55-61]	Q8WUM0 1xTMT6plex	0,04	-0,33	0,15
[R],SSVHGVSFDISFDK,[E]	1xTMT6plex [K14];1xTM	Q08AD1	Q08AD1 [433-446]	Q08AD1 1xTMT6plex [K	0,01	-0,33	0,12
[R],KSSTSIDEDAGR,[R]	1xTMT6plex [K1];1xTM	Q9Y6V0	Q9Y6V0 [1614-1625]	Q9Y6V0 1xTMT6plex [K	0,04	-0,33	0,15
[R],SIDAALFR,[E]	1xTMT6plex [N-Term];1	Q5FWE3	Q5FWE3 [815-822]	Q5FWE3 1xTMT6plex [	0,02	-0,33	0,13
[R],SHSSPSQIPK,[K]	1xTMT6plex [K10];1xTM	Q9H792	Q9H792 [1033-1042]	Q9H792 1xTMT6plex [K	0,01	-0,33	0,12
[K],LATPAGLK,[K]	1xTMT6plex [K8];1xTM	Q15054	Q15054 [275-282]	Q15054 1xTMT6plex [K	0,03	-0,33	0,14
[R],VDSPSHGLVTSSLCIPSPAF	1xCarbamidomethyl [C1	Q9UER7	Q9UER7 [686-705]	Q9UER7 1xCarbamidor	0,02	-0,33	0,13
[R],SLSPLGGR,[D]	1xTMT6plex [N-Term];1	Q14004	Q14004 [315-322]	Q14004 1xTMT6plex [N	0,03	-0,33	0,14
[R],SVIEPLPVTPTR,[D]	1xTMT6plex [N-Term];1	Q13153	Q13153 [204-215]	Q13153 1xTMT6plex [N	0,04	-0,33	0,15
[R],RFSAGQWEAR,[R]	1xTMT6plex [N-Term];1	Q9Y4W2	Q9Y4W2 [439-448]	Q9Y4W2 1xTMT6plex [	0,01	-0,33	0,12
[R],HSSAPPK,[K]	1xTMT6plex [K7];1xTM	Q9Y5B9	Q9Y5B9 [1037-1043]	Q9Y5B9 1xTMT6plex [K	0,05	-0,33	0,16
[R],RGSQNSSEHRPPASSTSEI	1xTMT6plex [K21];1xTM	O15014	O15014 [411-431]	O15014 1xTMT6plex [K	0,03	-0,33	0,14
[R],GSSGCSEAGGAGHEEGRA	1xCarbamidomethyl [C5	Q9C0C9	Q9C0C9 [97-118]	Q9C0C9 1xCarbamidon	0,04	-0,33	0,16
[R],TPSPPPPIPEDIALGK,[K]	1xTMT6plex [K16];1xTM	Q5T200	Q5T200 [263-278]	Q5T200 1xTMT6plex [K	0,03	-0,33	0,14
[K],KSDGACDSPSSDK,[E]	1xCarbamidomethyl [C6	Q9UIG0	Q9UIG0 [151-163]	Q9UIG0 1xCarbamidom	0,04	-0,33	0,15
[K],QQLTGHSK,[R]	1xTMT6plex [K8];1xTM	Q9Y490	Q9Y490 [2267-2274]	Q9Y490 1xTMT6plex [K	0,02	-0,33	0,13
[R],GILLEDGSESPAK,[R]	1xTMT6plex [K13];1xTM	Q08999	Q08999 [1103-1115]	Q08999 1xTMT6plex [K	0,00	-0,33	0,11
[R],QLYVLGSPAMQR,[I]	1xTMT6plex [N-Term];1	P41226	P41226 [18-29]	P41226 1xTMT6plex [N	0,03	-0,33	0,13
[R],SFSSAHR,[H]	1xTMT6plex [N-Term];1	Q96RG2	Q96RG2 [40-46]	Q96RG2 1xTMT6plex [I	0,01	-0,33	0,12
[R],KSSTSIDEDAGR,[R]	1xTMT6plex [K1];1xTM	Q9Y6V0	Q9Y6V0 [1614-1625]	Q9Y6V0 1xTMT6plex [K	0,03	-0,33	0,14
[K],ALYSPLFPASECEECYTNAI	2xCarbamidomethyl [C1	Q92628	Q92628 [1291-1310]	Q92628 2xCarbamidom	0,01	-0,33	0,12
[K],KPQSSSTEPAR,[K]	1xTMT6plex [K1];1xTM	Q7Z4V5	Q7Z4V5 [415-425]	Q7Z4V5 1xTMT6plex [K	0,04	-0,33	0,15
[R],RLSEQLAHTPTAFK,[R]	1xTMT6plex [K14];1xTM	Q9UBF8	Q9UBF8 [509-522]	Q9UBF8 1xTMT6plex [K	0,02	-0,33	0,12
[R],QSHSGSISPYPK,[V]	1xTMT6plex [K12];1xTM	Q9UQ35	Q9UQ35 [987-998]	Q9UQ35 1xTMT6plex [I	0,05	-0,33	0,16
[K],ALLGPRDTR,[V]	1xTMT6plex [N-Term];1	Q9NYP7	Q9NYP7 [14-22]	Q9NYP7 1xTMT6plex [I	0,01	-0,33	0,12
[K],ALLQSSASR,[K]	1xTMT6plex [N-Term];1	Q9UQ80	Q9UQ80 [356-364]	Q9UQ80 1xTMT6plex [I	0,02	-0,33	0,13
[K],ALLQSSASR,[K]	1xTMT6plex [N-Term];1	Q9UQ80	Q9UQ80 [356-364]	Q9UQ80 1xTMT6plex [I	0,02	-0,33	0,13
[R],TGVLLPSSPEAEVPGKLSP	2xTMT6plex [K16; K20]	O60285	O60285 [438-457]	O60285 2xTMT6plex [K	0,01	-0,33	0,12
[K],RPASLSTAPSEK,[G]	1xTMT6plex [K12];1xTM	Q9UPN4	Q9UPN4 [111-122]	Q9UPN4 1xTMT6plex [I	0,02	-0,33	0,13
[R],FSALQPPAPSGSTPSTPVE	1xTMT6plex [N-Term];1	O43432	O43432 [1101-1123]	O43432 1xTMT6plex [N	0,00	-0,33	0,12
[R],SPGLSPSSPSPEFLGLR,[S]	1xTMT6plex [N-Term];1	Q86X51	Q86X51 [443-459]	Q86X51 1xTMT6plex [N	0,03	-0,33	0,13
[R],WTKPSSFSDSER,[-]	1xTMT6plex [K3];1xTM	Q8NC56	Q8NC56 [492-503]	Q8NC56 1xTMT6plex [K	0,04	-0,33	0,15
[K],IISPGGSSTPSSTR,[S]	1xTMT6plex [N-Term];1	Q5VT52	Q5VT52 [756-768]	Q5VT52 1xTMT6plex [N	0,04	-0,33	0,15
[R],RLSPPACTLR,[K]	1xCarbamidomethyl [C7	P28360	P28360 [158-167]	P28360 1xCarbamidom	0,01	-0,33	0,12
[R],KRSYSPDGK,[E]	2xTMT6plex [K1; K9];1x	P43243	P43243 [594-602]	P43243 2xTMT6plex [K	0,02	-0,33	0,13
[R],SSGHSSSELSPDAVEK,[A]	1xTMT6plex [K16];1xTM	Q9UQ35	Q9UQ35 [1378-1393]	Q9UQ35 1xTMT6plex [I	0,01	-0,33	0,12
[R],GLNSSFETSPK,[K]	1xTMT6plex [K11];1xTM	Q8IV63	Q8IV63 [51-61]	Q8IV63 1xTMT6plex [K	0,03	-0,33	0,14
[K],RKQSAEER,[A]	1xTMT6plex [K2];1xTM	O15062	O15062 [193-200]	O15062 1xTMT6plex [K	0,01	-0,33	0,12
[R],FCLSSPTEALK,[M]	1xCarbamidomethyl [C2	Q76L83	Q76L83 [1150-1160]	Q76L83 1xCarbamidom	0,05	-0,33	0,16
[R],LASEYLTPEEMVTFK,[K]	1xTMT6plex [K15];1xTM	O43290	O43290 [386-400]	O43290 1xTMT6plex [K	0,03	-0,33	0,14
[R],KLSAEELER,[K]	1xTMT6plex [K1];1xTM	Q9NXE8	Q9NXE8 [335-343]	Q9NXE8 1xTMT6plex [K	0,02	-0,33	0,12
[R],GHQEVLEGYPSSETELSLK	1xTMT6plex [K19];1xTM	Q9ULJ7	Q9ULJ7 [1389-1407]	Q9ULJ7 1xTMT6plex [K	0,01	-0,33	0,12
[K],LSPATPSR,[K]	1xTMT6plex [N-Term];1	Q9Y6X9	Q9Y6X9 [729-736]	Q9Y6X9 1xTMT6plex [N	0,03	-0,32	0,13
[R],FLEENSSDPTYTSSLGGK,[	1xTMT6plex [K18];1xTM	P54760	P54760 [764-781]	P54760 1xTMT6plex [K	0,03	-0,32	0,14
[R],FLEENSSDPTYTSSLGGK,[	1xTMT6plex [K18];1xTM	P54760	P54760 [764-781]	P54760 1xTMT6plex [K	0,03	-0,32	0,14
[R],ILGSASPEEEQEKPILDRPT	1xTMT6plex [K13];1xTM	Q7Z422	Q7Z422 [102-122]	Q7Z422 1xTMT6plex [K	0,03	-0,32	0,14
[R],GSPGGPEFLK,[T]	1xTMT6plex [K10];1xTM	Q4VC44	Q4VC44 [502-511]	Q4VC44 1xTMT6plex [K	0,02	-0,32	0,13
[R],RFSYSQSK,[S]	1xTMT6plex [K8];1xTM	Q8WXF0	Q8WXF0 [144-151]	Q8WXF0 1xTMT6plex [	0,01	-0,32	0,12
[R],FAIQDISVEETSAK,[E]	1xTMT6plex [K14];1xTM	P12814; F	P12814 [134-147]; P35609 [141-154]	P12814 1xTMT6plex [K	0,02	-0,32	0,13
[R],ESSPSHGLLK,[L]	1xTMT6plex [K10];1xTM	Q96JH7	Q96JH7 [996-1005]	Q96JH7 1xTMT6plex [K	0,04	-0,32	0,16
[R],LSTTPAHSPVLK,[H]	1xTMT6plex [K12];1xTM	Q8IY63	Q8IY63 [899-910]	Q8IY63 1xTMT6plex [K	0,05	-0,32	0,16
[R],KPSGDSQPSSPR,[Y]	1xTMT6plex [K1];1xTM	Q9C0H5	Q9C0H5 [294-305]	Q9C0H5 1xTMT6plex [K	0,04	-0,32	0,15
[K],TPTSSPASSPLVAK,[K]	1xTMT6plex [K14];1xTM	Q14684	Q14684 [728-741]	Q14684 1xTMT6plex [K	0,03	-0,32	0,15

[R],SRSPLGFYVHLK,[N]	1xTMT6plex [K12];1xTM	Q8IXT5	Q8IXT5 [278-289]	Q8IXT5 1xTMT6plex [K	0,01	-0,32	0,12
[R],TPHTPGTPKSAILVPSEK,[K	2xTMT6plex [K9; K18];1	P11137	P11137 [1625-1642]	P11137 2xTMT6plex [K	0,01	-0,32	0,12
[R],SITSPPSTSTTK,[R]	1xTMT6plex [K12];1xTM	Q14938	Q14938 [265-276]	Q14938 1xTMT6plex [K	0,02	-0,32	0,13
[K],SPQVLGSSLSVR,[S]	1xTMT6plex [N-Term];1	Q86UU0	Q86UU0 [975-986]	Q86UU0 1xTMT6plex [N	0,04	-0,32	0,15
[K],DLLVSSGSNNSLPCGSPK,[	1xCarbamidomethyl [C1	Q5THK1	Q5THK1 [1014-1031]	Q5THK1 1xCarbamidom	0,04	-0,32	0,16
[K],LENSPGHGK,[S]	1xTMT6plex [K9];1xTM	Q8IY63	Q8IY63 [921-929]	Q8IY63 1xTMT6plex [K	0,02	-0,32	0,12
[R],TPTHQPPKPGSPGRPACPI	1xCarbamidomethyl [C1	Q96F63	Q96F63 [202-231]	Q96F63 1xCarbamidom	0,05	-0,32	0,16
[K],EPLRYSYNPDQFHNMDLR,	1xTMT6plex [N-Term];1	Q76N89	Q76N89 [37-54]	Q76N89 1xTMT6plex [N	0,02	-0,32	0,12
[R],WDQTADQTPGATPK,[K]	1xTMT6plex [K14];1xTM	O75533	O75533 [200-213]	O75533 1xTMT6plex [K	0,04	-0,32	0,15
[K],STTPVSK,[S]	1xTMT6plex [K7];1xTM	Q04727	Q04727 [316-322]	Q04727 1xTMT6plex [K	0,05	-0,32	0,16
[K],SLTNLSFLTDSEK,[K]	1xTMT6plex [K13];1xTM	Q8NEY1	Q8NEY1 [90-102]	Q8NEY1 1xTMT6plex [	0,00	-0,32	0,11
[K],RPPSGTSTTSK,[S]	1xTMT6plex [K11];1xTM	Q68DC2	Q68DC2 [721-731]	Q68DC2 1xTMT6plex [K	0,00	-0,32	0,11
[K],YSPTSPTYSPTSPK,[YG]	1xTMT6plex [K14];1xTM	P24928	P24928 [1909-1922]; [1923-1936]	P24928 1xTMT6plex [K	0,00	-0,32	0,11
[K],LDSPDPFKLNDPFQPFPGN	2xTMT6plex [K8; K23];1	P42566	P42566 [794-816]	P42566 2xTMT6plex [K	0,02	-0,32	0,13
[K],RVPSPTPAPKEAVR,[E]	1xTMT6plex [K10];1xTM	Q9UQ35	Q9UQ35 [2578-2591]	Q9UQ35 1xTMT6plex [	0,01	-0,32	0,12
[R],GSPEEELPLPAFEK,[L]	1xTMT6plex [K14];1xTM	Q5JTD0	Q5JTD0 [299-312]	Q5JTD0 1xTMT6plex [K	0,04	-0,32	0,15
[R],YSPSQNSPIHHIPSR,[R]	1xTMT6plex [N-Term];1	Q9NYF8	Q9NYF8 [284-298]	Q9NYF8 1xTMT6plex [N	0,01	-0,32	0,12
[R],LLHQFSFSPER,[E]	1xTMT6plex [N-Term];1	Q9UJX6	Q9UJX6 [527-537]	Q9UJX6 1xTMT6plex [N	0,04	-0,32	0,15
[R],RGSMNNELLSPEFGPVRDI	1xTMT6plex [N-Term];1	O00562	O00562 [591-619]	O00562 1xTMT6plex [N	0,00	-0,32	0,12
[R],RPGSLPAPPPSQLPR,[N]	1xTMT6plex [N-Term];1	P39880	P39880 [422-436]	P39880 1xTMT6plex [N	0,02	-0,32	0,13
[K],ENLSPGFSHLLSK,[N]	1xTMT6plex [K13];1xTM	Q99550	Q99550 [991-1003]	Q99550 1xTMT6plex [K	0,03	-0,32	0,14
[R],ATSGSSLPVGINR,[Q]	1xTMT6plex [N-Term];1	Q13905	Q13905 [309-321]	Q13905 1xTMT6plex [N	0,01	-0,32	0,12
[R],AGSRLSAEER,[Q]	1xTMT6plex [N-Term];1	Q86Y82	Q86Y82 [137-146]	Q86Y82 1xTMT6plex [N	0,01	-0,32	0,12
[R],LAGSALTDKHSDKS,[-]	2xTMT6plex [K9; K13];1	Q9NZN5	Q9NZN5 [1531-1544]	Q9NZN5 2xTMT6plex [	0,01	-0,32	0,12
[R],SPPGPAGSSPK,[Q]	1xTMT6plex [K11];1xTM	Q9H9D4	Q9H9D4 [338-348]	Q9H9D4 1xTMT6plex [K	0,01	-0,32	0,12
[K],NVMSAFGLTDDQVSGPPSA	1xTMT6plex [N-Term];1	Q92734	Q92734 [180-203]	Q92734 1xTMT6plex [N	0,02	-0,32	0,12
[R],FGAPHASESCSK,[N]	1xCarbamidomethyl [C1	O75170	O75170 [656-667]	O75170 1xCarbamidom	0,02	-0,32	0,13
[R],RLSLDIQSPPNIGLR,[R]	1xTMT6plex [N-Term];1	Q9NSI6	Q9NSI6 [694-708]	Q9NSI6 1xTMT6plex [N	0,01	-0,32	0,12
[R],SGTNSPPPPFSDWGR,[L]	1xTMT6plex [N-Term];1	Q9UKT5	Q9UKT5 [8-22]	Q9UKT5 1xTMT6plex [N	0,01	-0,32	0,12
[K],LETGAPRPPATVTNAVSWR	1xTMT6plex [N-Term];1	Q9BXS5	Q9BXS5 [142-160]	Q9BXS5 1xTMT6plex [N	0,04	-0,32	0,15
[K],GSASSGSTTSPTCSPK,[H]	1xCarbamidomethyl [C1	Q8IZD0	Q8IZD0 [247-262]	Q8IZD0 1xCarbamidom	0,02	-0,32	0,12
[R],LQPGVGTSGR,[G]	1xTMT6plex [N-Term];1	Q8IVB5	Q8IVB5 [9-18]	Q8IVB5 1xTMT6plex [N	0,02	-0,32	0,13
[K],IEDVGSDEEDDSGKDKK,[K	3xTMT6plex [K14; K16;	P08238	P08238 [250-266]	P08238 3xTMT6plex [K	0,00	-0,32	0,12
[R],AKSPTPDGSER,[I]	1xTMT6plex [K2];1xTM	Q96MU7	Q96MU7 [144-154]	Q96MU7 1xTMT6plex [	0,02	-0,32	0,13
[R],LEAGGASPK,[L]	1xTMT6plex [K9];1xTM	O95873	O95873 [106-114]	O95873 1xTMT6plex [K	0,01	-0,32	0,12
[R],KPSDEEFASR,[K]	1xTMT6plex [K1];1xTM	Q14155	Q14155 [692-701]	Q14155 1xTMT6plex [K	0,03	-0,32	0,14
[K],AGTVSPGKEDPVSSR,[R]	1xTMT6plex [K8];1xTM	Q7Z2K8	Q7Z2K8 [448-462]	Q7Z2K8 1xTMT6plex [K	0,01	-0,32	0,12
[R],MGFTEVTPVTGASLR,[R]	1xTMT6plex [N-Term];1	P55265	P55265 [802-816]	P55265 1xTMT6plex [N	0,03	-0,31	0,13
[K],DLLPSPAGPVPSKDPK,[T]	2xTMT6plex [K13; K16]	Q9UHB7	Q9UHB7 [810-825]	Q9UHB7 2xTMT6plex [	0,00	-0,31	0,11
[R],VPSGAAGGK,[M]	1xTMT6plex [K9];1xTM	Q14160	Q14160 [1283-1291]	Q14160 1xTMT6plex [K	0,02	-0,31	0,13
[R],ISICSSDK,[R]	1xCarbamidomethyl [C4	Q13547	Q13547 [405-412]	Q13547 1xCarbamidom	0,04	-0,31	0,15
[R],SYSSSHSSPAK,[I]	1xTMT6plex [K11];1xTM	Q9H792	Q9H792 [723-733]	Q9H792 1xTMT6plex [K	0,05	-0,31	0,16
[R],SGHFDVTTVASIK,[E]	1xTMT6plex [K13];1xTM	Q8TDM6	Q8TDM6 [1795-1807]	Q8TDM6 1xTMT6plex [	0,03	-0,31	0,13
[R],VSPATPK,[A]	1xTMT6plex [K7];1xTM	Q9HAH7	Q9HAH7 [280-286]	Q9HAH7 1xTMT6plex [	0,00	-0,31	0,11
[K],HSTPSPTR,[Y]	1xTMT6plex [N-Term];1	P49792	P49792 [777-784]	P49792 1xTMT6plex [N	0,01	-0,31	0,12
[K],APVPGTPDSLSSGSSR,[D]	1xTMT6plex [N-Term];1	Q9UJZ1	Q9UJZ1 [322-337]	Q9UJZ1 1xTMT6plex [N	0,02	-0,31	0,13
[R],HKSDNETNLQQQVVWGNF	1xTMT6plex [K2];1xTM	Q7Z401	Q7Z401 [1013-1030]	Q7Z401 1xTMT6plex [K	0,01	-0,31	0,12
[R],LRSFSASQSTDR,[E]	1xTMT6plex [N-Term];1	Q9ULJ3	Q9ULJ3 [407-418]	Q9ULJ3 1xTMT6plex [N	0,02	-0,31	0,13
[R],VLSSTSEEDEPGVVK,[F]	1xTMT6plex [K15];1xTM	Q8NI08	Q8NI08 [206-220]	Q8NI08 1xTMT6plex [K	0,00	-0,31	0,12
[R],KSSTSPK,[W]	2xTMT6plex [K1; K7];1x	Q9Y2W1	Q9Y2W1 [915-921]	Q9Y2W1 2xTMT6plex [	0,01	-0,31	0,12
[K],ISHLSGSGSGDER,[V]	1xTMT6plex [N-Term];1	Q96T37	Q96T37 [204-216]	Q96T37 1xTMT6plex [N	0,01	-0,31	0,12
[K],EFVSPSLTPPPAIAHPALPT	1xTMT6plex [N-Term];1	O94763	O94763 [485-508]	O94763 1xTMT6plex [N	0,04	-0,31	0,15
[R],KPSEDEVLNK,[G]	2xTMT6plex [K1; K10];1	Q8NDI1	Q8NDI1 [1056-1065]	Q8NDI1 2xTMT6plex [K	0,00	-0,31	0,12
[R],ERHPSWR,[S]	1xTMT6plex [N-Term];1	P23588	P23588 [402-408]	P23588 1xTMT6plex [N	0,00	-0,31	0,11
[R],LTPVRPAAASPIVSGAR,[R]	1xTMT6plex [N-Term];2	Q9Y2K7	Q9Y2K7 [549-565]	Q9Y2K7 1xTMT6plex [N	0,01	-0,31	0,12
[K],NDKSEEEQSSSSVK,[K]	2xTMT6plex [K3; K14];1	P07910	P07910 [230-243]	P07910 2xTMT6plex [K	0,01	-0,31	0,12
[K],SVDWTHFGGSPPSDEIR,[T	1xTMT6plex [N-Term];1	Q92574	Q92574 [673-689]	Q92574 1xTMT6plex [N	0,03	-0,31	0,14

[R],GTTPPPPSWEPNSQTK,[R]	1xTMT6plex [K16];1xTM	O95835	O95835 [260-275]	O95835 1xTMT6plex [K	0,01	-0,31	0,12
[R],GGPISFSSSR,[S]	1xTMT6plex [N-Term];1	P35580	P35580 [1931-1940]	P35580 1xTMT6plex [N	0,01	-0,31	0,12
[R],REPVPFPSGSAGPGPR,[G]	1xTMT6plex [N-Term];1	Q9UBB5	Q9UBB5 [124-139]	Q9UBB5 1xTMT6plex [I	0,03	-0,31	0,14
[R],LGSSEHGQK,[S]	1xTMT6plex [K9];1xTM	Q9P267	Q9P267 [203-211]	Q9P267 1xTMT6plex [K	0,01	-0,31	0,12
[R],KSSLVTSKLAGGQVE,[-]	2xTMT6plex [K1; K8];1x	O43768	O43768 [107-121]	O43768 2xTMT6plex [K	0,02	-0,31	0,12
[K],HTVVEHLVSHHSPQR,[T]	1xTMT6plex [N-Term];1	Q5T0B9	Q5T0B9 [393-407]	Q5T0B9 1xTMT6plex [N	0,01	-0,31	0,12
[R],SEGPSLSPSGK,[R]	1xTMT6plex [K11];1xTM	A8MU93	A8MU93 [48-58]	A8MU93 1xTMT6plex [K	0,04	-0,31	0,15
[R],RSSSSGDQSSDSLNSPTLL	1xTMT6plex [N-Term];1	P15408	P15408 [306-326]	P15408 1xTMT6plex [N	0,03	-0,31	0,13
[K],RSPTPGK,[G]	1xTMT6plex [K7];1xTM	P78559	P78559 [2628-2634]	P78559 1xTMT6plex [K	0,00	-0,31	0,12
[R],STPLASPSPSPGR,[S]	1xTMT6plex [N-Term];1	Q8WYP5	Q8WYP5 [1209-1221]	Q8WYP5 1xTMT6plex [	0,01	-0,31	0,12
[R],VDSTTCLFPVEEK,[A]	1xCarbamidomethyl [C6	Q06210	Q06210 [259-271]	Q06210 1xCarbamidom	0,04	-0,31	0,15
[R],SGKSPSPSPTSPGSLR,[K]	1xTMT6plex [K3];1xTM	O15075	O15075 [327-342]	O15075 1xTMT6plex [K	0,00	-0,31	0,11
[K],KSDGACDSPSSDK,[E]	1xCarbamidomethyl [C6	Q9UIG0	Q9UIG0 [151-163]	Q9UIG0 1xCarbamidom	0,04	-0,31	0,15
[R],RDSPTYDPYKR,[S]	1xTMT6plex [K10];1xTM	Q8N2M8	Q8N2M8 [292-302]	Q8N2M8 1xTMT6plex [I	0,02	-0,31	0,12
[R],LRSPGSK,[G]	1xTMT6plex [K7];1xTM	Q8N9B5	Q8N9B5 [128-134]	Q8N9B5 1xTMT6plex [K	0,05	-0,31	0,16
[R],VSLSPHSPLLSPK,[V]	1xTMT6plex [K13];1xTM	O15069	O15069 [1297-1309]	O15069 1xTMT6plex [K	0,01	-0,31	0,12
[R],GSVHSLDAGLLLPSGDPFS	1xTMT6plex [K20];1xTM	Q15276	Q15276 [373-392]	Q15276 1xTMT6plex [K	0,03	-0,31	0,14
[R],REPESPPASAPIPLFGADTI	1xTMT6plex [N-Term];1	Q96AY4	Q96AY4 [24-46]	Q96AY4 1xTMT6plex [N	0,05	-0,31	0,16
[R],LGPGSSGGSAR,[E]	1xTMT6plex [N-Term];1	P17152	P17152 [9-19]	P17152 1xTMT6plex [N	0,05	-0,31	0,16
[R],LGPGSSGGSAR,[E]	1xTMT6plex [N-Term];1	P17152	P17152 [9-19]	P17152 1xTMT6plex [N	0,05	-0,31	0,16
[K],VVVSPTK,[K]	1xTMT6plex [K7];1xTM	P19338	P19338 [64-70]	P19338 1xTMT6plex [K	0,01	-0,31	0,12
[R],KSSTVESEIASEEK,[S]	2xTMT6plex [K1; K14];1	Q9Y2K1	Q9Y2K1 [303-316]	Q9Y2K1 2xTMT6plex [K	0,00	-0,31	0,11
[R],GNSLPCVLEQK,[I]	1xCarbamidomethyl [C6	Q08495	Q08495 [331-341]	Q08495 1xCarbamidom	0,02	-0,31	0,13
[R],TAAGISTPAPVAGLGPR,[A]	1xTMT6plex [N-Term];1	Q9UBU6	Q9UBU6 [189-205]	Q9UBU6 1xTMT6plex [I	0,02	-0,31	0,12
[K],MEVEDGLGSPKPEEIK,[D]	2xTMT6plex [K11; K16]	Q71F56	Q71F56 [915-930]	Q71F56 2xTMT6plex [K	0,04	-0,31	0,15
[R],LSPAYSLGSLTGASPCQSP	2xCarbamidomethyl [C1	Q86UU1	Q86UU1 [538-560]	Q86UU1 2xCarbamidon	0,04	-0,31	0,15
[K],ASLLHSMPTHSSPR,[S]	1xOxidation [M7];1xTM	O75122	O75122 [1002-1015]	O75122 1xOxidation [M	0,03	-0,31	0,14
[R],NFILDQTNVSAAAQR,[R]	1xTMT6plex [N-Term];1	Q00839	Q00839 [576-590]	Q00839 1xTMT6plex [N	0,00	-0,31	0,11
[K],DAGEGGLSLAIEGPSK,[A]	1xTMT6plex [K16];1xTM	P21333	P21333 [1892-1907]	P21333 1xTMT6plex [K	0,00	-0,31	0,11
[R],HLPSPPLTQEVDLHSIAGPF	1xTMT6plex [N-Term];1	Q07889	Q07889 [1272-1295]	Q07889 1xTMT6plex [N	0,05	-0,31	0,16
[R],TNKPSTPTTATR,[K]	1xTMT6plex [K3];1xTM	Q9UBP0	Q9UBP0 [298-309]	Q9UBP0 1xTMT6plex [I	0,00	-0,31	0,12
[K],QKLSECSLTK,[G]	1xCarbamidomethyl [C6	Q96PZ0	Q96PZ0 [33-42]	Q96PZ0 1xCarbamidom	0,00	-0,31	0,12
[R],VKSPGGPGSHVR,[Q]	1xTMT6plex [K2];1xTM	O75382	O75382 [435-446]	O75382 1xTMT6plex [K	0,05	-0,31	0,16
[R],VPGSSGHLHK,[T]	1xTMT6plex [K10];1xTM	Q9UEW8	Q9UEW8 [367-376]	Q9UEW8 1xTMT6plex [	0,02	-0,31	0,12
[K],TPSPKEEDEEPESPPEKK,[	3xTMT6plex [K5; K17; I	Q9H1E3	Q9H1E3 [202-219]	Q9H1E3 3xTMT6plex [K	0,01	-0,31	0,12
[R],ESPVSAPLEGLICR,[A]	1xCarbamidomethyl [C1	O75376	O75376 [1262-1275]	O75376 1xCarbamidom	0,04	-0,31	0,15
[R],SFELPPYTPPPILSPVR,[E]	1xTMT6plex [N-Term];2	Q6PJG2	Q6PJG2 [648-664]	Q6PJG2 1xTMT6plex [N	0,04	-0,30	0,15
[R],EAAVSASDILQESAIHSPGT	1xTMT6plex [K23];1xTM	Q96C57	Q96C57 [163-185]	Q96C57 1xTMT6plex [K	0,02	-0,30	0,13
[K],KPSPQPSSPR,[Q]	1xTMT6plex [K1];1xTM	O75909	O75909 [322-331]	O75909 1xTMT6plex [K	0,02	-0,30	0,13
[K],TLGTPTQPGSTPR,[I]	1xTMT6plex [N-Term];1	Q8NFH5	Q8NFH5 [270-282]	Q8NFH5 1xTMT6plex [I	0,01	-0,30	0,12
[R],SSLGSLQTPEAVTTR,[K]	1xTMT6plex [N-Term];1	Q7Z2W4	Q7Z2W4 [386-400]	Q7Z2W4 1xTMT6plex [	0,04	-0,30	0,15
[K],DAVPKPPLSPHK,[L]	2xTMT6plex [K5; K12];1	Q7L4P6	Q7L4P6 [353-364]	Q7L4P6 2xTMT6plex [K	0,00	-0,30	0,12
[R],SPVPSPGSSSPQLQVK,[S]	1xTMT6plex [K16];1xTM	Q8N3F8	Q8N3F8 [612-627]	Q8N3F8 1xTMT6plex [K	0,01	-0,30	0,12
[R],LTIHAPPQELGPPVQR,[I]	1xTMT6plex [N-Term];1	P12270	P12270 [2085-2100]	P12270 1xTMT6plex [N	0,01	-0,30	0,12
[R],QFLGLGIPSSLSATTK,[I]	1xTMT6plex [K16];1xTM	Q6ZS81	Q6ZS81 [924-939]	Q6ZS81 1xTMT6plex [K	0,01	-0,30	0,12
[R],SSRDRSPR,[D]	1xTMT6plex [N-Term];1	Q9Y383	Q9Y383 [353-360]	Q9Y383 1xTMT6plex [N	0,05	-0,30	0,16
[R],EVSSRPSTPGLSVVSGISA	1xTMT6plex [K27];1xTM	Q14C86	Q14C86 [755-781]	Q14C86 1xTMT6plex [K	0,04	-0,30	0,15
[R],VQNHLPASGPTQPPVVSST	1xTMT6plex [K32];1xTM	O43294	O43294 [170-201]	O43294 1xTMT6plex [K	0,04	-0,30	0,16
[R],TTSFFSR,[Q]	1xTMT6plex [N-Term];1	Q9BZ67	Q9BZ67 [437-443]	Q9BZ67 1xTMT6plex [N	0,03	-0,30	0,14
[R],SLGEQDQMTLRPPEK,[V]	1xTMT6plex [K15];1xTM	P48681	P48681 [768-782]	P48681 1xTMT6plex [K	0,01	-0,30	0,12
[K],ASTSDYQVISDRQTPK,[K]	1xTMT6plex [K16];1xTM	Q8NFH5	Q8NFH5 [295-310]	Q8NFH5 1xTMT6plex [I	0,02	-0,30	0,13
[R],SLPSTSSTSSTK,[R]	1xTMT6plex [K12];1xTM	Q12857	Q12857 [265-276]	Q12857 1xTMT6plex [K	0,00	-0,30	0,11
[K],AALPAATTPGPGLETAGPA	1xTMT6plex [N-Term];1	Q10586	Q10586 [56-88]	Q10586 1xTMT6plex [N	0,01	-0,30	0,12
[R],RPGQSFHVNSEVNSVLSPF	1xTMT6plex [N-Term];1	Q14671	Q14671 [193-211]	Q14671 1xTMT6plex [N	0,00	-0,30	0,12
[R],KRTPSPSYQR,[T]	1xTMT6plex [K1];1xTM	Q5T200	Q5T200 [352-361]	Q5T200 1xTMT6plex [K	0,02	-0,30	0,13
[K],FDSSLLSSDDETK,[C]	1xTMT6plex [K13];1xTM	P28715	P28715 [556-568]	P28715 1xTMT6plex [K	0,00	-0,30	0,11
[R],SYSPDHR,[Q]	1xTMT6plex [N-Term];1	Q14241	Q14241 [149-155]	Q14241 1xTMT6plex [N	0,01	-0,30	0,12

[K],TSGPLSPPTGPPGPAPAGF	1xTMT6plex [N-Term];1	P49815	P49815 [659-680]	P49815 1xTMT6plex [N	0,01	-0,30	0,12
[R],SPSFDTSTATSK,[L]	1xTMT6plex [K12];1xTN	A7MBM2	A7MBM2 [1173-1184]	A7MBM2 1xTMT6plex [	0,01	-0,30	0,12
[K],NMTVEQLLTGSPTSPTVEP	1xOxidation [M2];1xTM	Q9C0C9	Q9C0C9 [826-849]	Q9C0C9 1xOxidation [M	0,02	-0,30	0,12
[K],VSPSKSPSLSPSPPSPLEK,	2xTMT6plex [K5; K19];1	P46821	P46821 [1251-1269]	P46821 2xTMT6plex [K	0,01	-0,30	0,12
[R],RRSPPPR,[R]	1xTMT6plex [N-Term];1	P84103	P84103 [106-112]	P84103 1xTMT6plex [N	0,01	-0,30	0,12
[R],RQSPSPSTRPIR,[R]	1xTMT6plex [N-Term];1	Q8IYB3	Q8IYB3 [711-722]	Q8IYB3 1xTMT6plex [N	0,01	-0,30	0,12
[R],GSPTRPNPPVR,[N]	1xTMT6plex [N-Term];1	Q14195	Q14195 [521-531]	Q14195 1xTMT6plex [N	0,00	-0,30	0,11
[K],DASSSPASTASSASSTSLK,	1xTMT6plex [K19];1xTN	Q04724	Q04724 [282-300]	Q04724 1xTMT6plex [K	0,02	-0,30	0,13
[R],RLSGGSHSYGGESPR,[L]	1xTMT6plex [N-Term];1	Q13905	Q13905 [333-347]	Q13905 1xTMT6plex [N	0,04	-0,30	0,15
[R],ASPSPPPK,[R]	1xTMT6plex [K8];1xTM	Q8IYB3	Q8IYB3 [625-632]	Q8IYB3 1xTMT6plex [K	0,03	-0,30	0,14
[K],NASILLEELDLEK,[L]	1xTMT6plex [K13];1xTN	O75179	O75179 [1455-1467]	O75179 1xTMT6plex [K	0,01	-0,30	0,12
[R],VYSPYNHR,[H]	1xTMT6plex [N-Term];1	Q9NS56	Q9NS56 [583-590]	Q9NS56 1xTMT6plex [N	0,03	-0,30	0,14
[K],NQKNKPGPNIESGNEDDD/	3xTMT6plex [K3; K5; K	O60841	O60841 [203-224]	O60841 3xTMT6plex [K	0,01	-0,30	0,12
[R],RVSLTQR,[T]	1xTMT6plex [N-Term];1	Q96I34	Q96I34 [351-357]	Q96I34 1xTMT6plex [N-	0,03	-0,30	0,14
[K],KTESASVQGR,[N]	1xTMT6plex [K1];1xTM	Q9P2E9	Q9P2E9 [599-608]	Q9P2E9 1xTMT6plex [K	0,05	-0,30	0,16
[R],ALNIVDQEGSLLGK,[G]	1xTMT6plex [K14];1xTN	Q8IX01	Q8IX01 [215-228]	Q8IX01 1xTMT6plex [K	0,04	-0,30	0,16
[R],SFAPSTPLTGR,[R]	1xTMT6plex [N-Term];1	P28749	P28749 [364-374]	P28749 1xTMT6plex [N	0,02	-0,30	0,12
[R],SGDVLAK,[N]	1xTMT6plex [K7];1xTM	P85299	P85299 [240-246]	P85299 1xTMT6plex [K	0,02	-0,30	0,13
[K],KDEWGLVAPISPGPLTPMR	1xTMT6plex [K1];1xTM	P11137	P11137 [275-293]	P11137 1xTMT6plex [K	0,03	-0,30	0,14
[R],ELTPASPTCTNSVSK,[N]	1xCarbamidomethyl [C9	P28715	P28715 [521-535]	P28715 1xCarbamidom	0,00	-0,30	0,11
[R],QILIACSPVSSVR,[K]	1xCarbamidomethyl [C6	Q5QJE6	Q5QJE6 [111-123]	Q5QJE6 1xCarbamidon	0,03	-0,30	0,13
[R],APQTSSSPPPVR,[R]	1xTMT6plex [N-Term];1	Q8IYB3	Q8IYB3 [690-701]	Q8IYB3 1xTMT6plex [N	0,01	-0,30	0,12
[R],KRTEALEQGGLPK,[K]	2xTMT6plex [K1; K13];1	P50613	P50613 [330-342]	P50613 2xTMT6plex [K	0,01	-0,30	0,12
[R],THSLLYQPQK,[K]	1xTMT6plex [K10];1xTN	O95490	O95490 [1332-1341]	O95490 1xTMT6plex [K	0,03	-0,30	0,14
[R],IPSASPQTQR,[K]	1xTMT6plex [N-Term];1	Q13233	Q13233 [1014-1023]	Q13233 1xTMT6plex [N	0,01	-0,30	0,12
[R],GSGGGGGPQVPHQSPPK,	1xTMT6plex [K17];1xTN	Q9NYF3	Q9NYF3 [149-165]	Q9NYF3 1xTMT6plex [K	0,02	-0,30	0,13
[R],SNSISQIPSLEEMQQLR,[S]	1xTMT6plex [N-Term];1	Q9NYJ8	Q9NYJ8 [580-596]	Q9NYJ8 1xTMT6plex [N	0,02	-0,30	0,12
[K],ISEGLPTPTK,[M]	1xTMT6plex [K10];1xTN	P06400	P06400 [815-824]	P06400 1xTMT6plex [K	0,04	-0,30	0,15
[R],KQSAGPNSPTGGGGGGGS	1xTMT6plex [K1];1xTM	A7E2V4	A7E2V4 [46-67]	A7E2V4 1xTMT6plex [K	0,05	-0,30	0,16
[R],VSGEEELHTGPPAPQGPLS	1xTMT6plex [N-Term];1	Q9UPN7	Q9UPN7 [729-763]	Q9UPN7 1xTMT6plex [	0,03	-0,30	0,13
[R],SLHSAHSLASR,[R]	1xTMT6plex [N-Term];1	O15027	O15027 [1356-1366]	O15027 1xTMT6plex [N	0,00	-0,30	0,12
[R],ITHSNAGHLR,[G]	1xTMT6plex [N-Term];1	Q86YR5	Q86YR5 [578-587]	Q86YR5 1xTMT6plex [N	0,00	-0,30	0,12
[R],KRSPSPSPTPEAK,[K]	2xTMT6plex [K1; K13];1	Q8TAQ2	Q8TAQ2 [300-312]	Q8TAQ2 2xTMT6plex [I	0,02	-0,30	0,12
[K],KSEAPSGESR,[K]	1xTMT6plex [K1];1xTM	B5ME19	B5ME19 [200-209]	B5ME19 1xTMT6plex [K	0,03	-0,30	0,14
[R],RWSTELVPEGLQSPR,[G]	1xTMT6plex [N-Term];1	B2RTY4	B2RTY4 [1305-1319]	B2RTY4 1xTMT6plex [N	0,01	-0,30	0,12
[R],GSDSLIK,[G]	1xTMT6plex [K7];1xTM	Q99623	Q99623 [290-296]	Q99623 1xTMT6plex [K	0,01	-0,29	0,12
[R],KDSLESDSSTAIIPHELIR,[T	1xTMT6plex [K1];1xTM	Q9UMX1	Q9UMX1 [344-362]	Q9UMX1 1xTMT6plex [	0,02	-0,29	0,13
[R],FSTPPGELDGGISGR,[S]	1xTMT6plex [N-Term];1	Q9H165	Q9H165 [699-713]	Q9H165 1xTMT6plex [N	0,04	-0,29	0,15
[R],ARHTLDELNPQK,[S]	1xTMT6plex [K12];1xTN	Q13131	Q13131 [385-396]	Q13131 1xTMT6plex [K	0,01	-0,29	0,12
[R],LSPFHGSSPPQSTPLSPPP	1xTMT6plex [K23];1xTN	Q9H7D0	Q9H7D0 [1774-1796]	Q9H7D0 1xTMT6plex [K	0,05	-0,29	0,16
[R],IHAESLLLDSPAVAK,[S]	1xTMT6plex [K15];1xTN	Q9H4L5	Q9H4L5 [401-415]	Q9H4L5 1xTMT6plex [K	0,00	-0,29	0,11
[R],TLNAETPKSSPLPAK,[G]	2xTMT6plex [K8; K15];1	Q7L2J0	Q7L2J0 [208-222]	Q7L2J0 2xTMT6plex [K	0,01	-0,29	0,12
[K],IWDPTPSHTPAGAATPGR,[	1xTMT6plex [N-Term];1	O75533	O75533 [253-270]	O75533 1xTMT6plex [N	0,01	-0,29	0,12
[R],KPESNAVTK,[T]	2xTMT6plex [K1; K9];1x	P27816	P27816 [968-976]	P27816 2xTMT6plex [K	0,05	-0,29	0,16
[K],TPELPEPSVKVK,[E]	2xTMT6plex [K10; K12]	Q8IYB3	Q8IYB3 [220-231]	Q8IYB3 2xTMT6plex [K	0,00	-0,29	0,11
[R],QLSSTSPLAPYPTSQMVSS	1xOxidation [M16];1xTN	Q6UUV7	Q6UUV7 [408-428]	Q6UUV7 1xOxidation [M	0,04	-0,29	0,16
[R],KKSEPDDELLFDFLNSSQK	3xTMT6plex [K1; K2; K	Q8TBA6	Q8TBA6 [114-132]	Q8TBA6 3xTMT6plex [K	0,01	-0,29	0,12
[R],FDLSHGSPQMVR,[R]	1xTMT6plex [N-Term];1	Q8IVT5	Q8IVT5 [328-339]	Q8IVT5 1xTMT6plex [N	0,00	-0,29	0,12
[R],IDFIPVSPAPSPTR,[G]	1xTMT6plex [N-Term];1	Q96E09	Q96E09 [137-150]	Q96E09 1xTMT6plex [N	0,03	-0,29	0,13
[K],LLSPQMSGEEEDSDLAAK,[	1xOxidation [M6];1xTM	P42858	P42858 [1868-1885]	P42858 1xOxidation [M	0,00	-0,29	0,12
[K],VFLQDGPARPASPEAGNTL	1xTMT6plex [N-Term];1	Q9UJX6	Q9UJX6 [303-322]	Q9UJX6 1xTMT6plex [N	0,00	-0,29	0,12
[K],SLSPGVSR,[D]	1xTMT6plex [N-Term];1	Q9UKV3	Q9UKV3 [655-662]	Q9UKV3 1xTMT6plex [I	0,02	-0,29	0,12
[R],TLSDPPSPLPHGPPNK,[G]	1xTMT6plex [K16];1xTN	Q9ULH1	Q9ULH1 [837-852]	Q9ULH1 1xTMT6plex [K	0,02	-0,29	0,13
[R],ASSLLSR,[F]	1xTMT6plex [N-Term];1	P48634	P48634 [158-164]	P48634 1xTMT6plex [N	0,04	-0,29	0,15
[R],TNSMGSATGPLPGTK,[V]	1xTMT6plex [K15];1xTN	O15014	O15014 [465-479]	O15014 1xTMT6plex [K	0,03	-0,29	0,14
[R],HGSFHEDEDPIGSPR,[L]	1xTMT6plex [N-Term];1	Q96T58	Q96T58 [1266-1280]	Q96T58 1xTMT6plex [N	0,01	-0,29	0,12
[R],LTMQVSSLQR,[E]	1xOxidation [M3];1xTM	P35659	P35659 [66-75]	P35659 1xOxidation [M	0,01	-0,29	0,12

[K],VAQGATEKSPEDK,[V]	2xTMT6plex [K8; K13];1	P49321	P49321 [443-455]	P49321 2xTMT6plex [K	0,00	-0,29	0,11
[R],DTSPSSGSAVSSSK,[V]	1xTMT6plex [K14];1xTM	Q8NEY8	Q8NEY8 [203-216]	Q8NEY8 1xTMT6plex [I	0,02	-0,29	0,12
[K],SPISVPGGSALISNLGK,[V]	1xTMT6plex [K17];1xTM	P51610	P51610 [666-682]	P51610 1xTMT6plex [K	0,03	-0,29	0,15
[K],GSSTPTKGIENK,[A]	2xTMT6plex [K7; K12];1	O60271	O60271 [362-373]	O60271 2xTMT6plex [K	0,01	-0,29	0,12
[K],TRHSPTPQQSNR,[T]	1xTMT6plex [N-Term];2	Q8IYB3	Q8IYB3 [411-422]	Q8IYB3 1xTMT6plex [N	0,05	-0,29	0,16
[K],SPSLGMLSTATR,[T]	1xTMT6plex [N-Term];1	Q14687	Q14687 [10-21]	Q14687 1xTMT6plex [N	0,01	-0,29	0,12
[R],CASLEEILSQR,[D]	1xCarbamidomethyl [C1	Q53GL0	Q53GL0 [269-279]	Q53GL0 1xCarbamidom	0,03	-0,29	0,13
[K],GSGFSLDVIDGPISQR,[E]	1xTMT6plex [N-Term];1	O43166	O43166 [254-269]	O43166 1xTMT6plex [N	0,03	-0,29	0,14
[R],AHSSLGPGRPR,[S]	1xTMT6plex [N-Term];1	Q9NRR6	Q9NRR6 [230-240]	Q9NRR6 1xTMT6plex [	0,01	-0,29	0,12
[K],SPTSPTSPHMPALR,[I]	1xTMT6plex [N-Term];1	P35711	P35711 [408-421]	P35711 1xTMT6plex [N	0,00	-0,29	0,12
[R],KPSGDSQPSSPR,[Y]	1xTMT6plex [K1];1xTM	Q9C0H5	Q9C0H5 [294-305]	Q9C0H5 1xTMT6plex [I	0,02	-0,29	0,12
[R],LFVLLPEQSPVSYSK,[R]	1xTMT6plex [K15];1xTM	Q96K76	Q96K76 [824-838]	Q96K76 1xTMT6plex [K	0,01	-0,29	0,12
[R],SSSDPPAVHPPLPPLR,[V]	1xTMT6plex [N-Term];1	O43150	O43150 [820-835]	O43150 1xTMT6plex [N	0,03	-0,29	0,13
[K],TVSPPTVCTIPTVVGR,[N]	1xCarbamidomethyl [C8	Q9BWT3	Q9BWT3 [597-612]	Q9BWT3 1xCarbamido	0,01	-0,29	0,12
[R],SSFDWLTGSSTDPLVDHTS	1xTMT6plex [K30];1xTM	P78527	P78527 [2654-2683]	P78527 1xTMT6plex [K	0,05	-0,29	0,16
[R],SSFDWLTGSSTDPLVDHTS	1xTMT6plex [K30];1xTM	P78527	P78527 [2654-2683]	P78527 1xTMT6plex [K	0,05	-0,29	0,16
[K],KDITTSADQIAEVK,[T]	2xTMT6plex [K1; K14];1	Q76I76	Q76I76 [482-495]	Q76I76 2xTMT6plex [K	0,00	-0,29	0,12
[R],RAPAVPPARPGSR,[G]	1xTMT6plex [N-Term];1	Q05193	Q05193 [784-796]	Q05193 1xTMT6plex [N	0,02	-0,29	0,13
[K],DLSTSPKPSPIPSPVLGR,[K	1xTMT6plex [K7];1xTM	Q8NDI1	Q8NDI1 [424-441]	Q8NDI1 1xTMT6plex [K	0,01	-0,29	0,12
[R],KHSNLITVPIQDDSNSGAR,	1xTMT6plex [K1];1xTM	Q75N03	Q75N03 [288-306]	Q75N03 1xTMT6plex [K	0,03	-0,29	0,14
[R],NEFWFSDGSLSDK,[S]	1xTMT6plex [K13];1xTM	Q9P2F8	Q9P2F8 [1544-1556]	Q9P2F8 1xTMT6plex [K	0,01	-0,29	0,12
[R],SISPPSGLPQPHGGALR,[Q	1xTMT6plex [N-Term];1	Q9P202	Q9P202 [243-259]	Q9P202 1xTMT6plex [N	0,04	-0,29	0,15
[R],SPGIVSTNLPSYQK,[R]	1xTMT6plex [K14];1xTM	Q86WG5	Q86WG5 [1687-1700]	Q86WG5 1xTMT6plex [	0,00	-0,29	0,12
[R],LSGSDLGGHSSLLER,[L]	1xTMT6plex [N-Term];1	O95613	O95613 [2476-2490]	O95613 1xTMT6plex [N	0,01	-0,29	0,12
[K],RPPISDSEELSAK,[K]	1xTMT6plex [K13];1xTM	P42568	P42568 [284-296]	P42568 1xTMT6plex [K	0,04	-0,29	0,15
[K],IWDPTPSHTPAGAATPGR,[	1xTMT6plex [N-Term];1	O75533	O75533 [253-270]	O75533 1xTMT6plex [N	0,02	-0,29	0,12
[K],SLSDSESDDSKSK,[K]	2xTMT6plex [K11; K13]	Q13185	Q13185 [93-105]	Q13185 2xTMT6plex [K	0,01	-0,29	0,12
[R],LLHPSPDLVSQEATLSEAR,	1xTMT6plex [N-Term];1	Q8IY22	Q8IY22 [373-391]	Q8IY22 1xTMT6plex [N	0,05	-0,29	0,16
[K],TRHSPTPQQSNR,[T]	1xTMT6plex [N-Term];2	Q8IYB3	Q8IYB3 [411-422]	Q8IYB3 1xTMT6plex [N	0,04	-0,29	0,16
[R],MDRTPPPPTLSPAAITVGR,	1xTMT6plex [N-Term];2	Q8NDX5	Q8NDX5 [606-624]	Q8NDX5 1xTMT6plex [I	0,01	-0,29	0,12
[R],GQSQLSNPTDDSWK,[G]	1xTMT6plex [K14];1xTM	Q9Y6D5	Q9Y6D5 [1523-1536]	Q9Y6D5 1xTMT6plex [I	0,02	-0,29	0,13
[R],APSIVQDGPQPSSGLEGM\	1xTMT6plex [N-Term];1	O94887	O94887 [1031-1050]	O94887 1xTMT6plex [N	0,02	-0,29	0,12
[R],GDFHSPIVLGRPPNTEDRE	1xTMT6plex [K24];1xTM	Q02078	Q02078 [475-498]	Q02078 1xTMT6plex [K	0,04	-0,29	0,16
[K],LASFSPSPPSPYPTTVGPVI	1xTMT6plex [N-Term];1	Q96SK2	Q96SK2 [192-216]	Q96SK2 1xTMT6plex [N	0,01	-0,28	0,12
[K],YLPHSAGR,[Y]	1xTMT6plex [N-Term];1	P46782	P46782 [48-55]	P46782 1xTMT6plex [N	0,03	-0,28	0,14
[R],HNEFNPQHSLLVQFR,[N]	1xTMT6plex [N-Term];1	Q99717	Q99717 [144-158]	Q99717 1xTMT6plex [N	0,01	-0,28	0,12
[K],LSESPASLPSCPPVETALIN	1xCarbamidomethyl [C1	Q2TAZ0	Q2TAZ0 [1260-1281]	Q2TAZ0 1xCarbamidom	0,02	-0,28	0,12
[K],NLNNSNLFSPVNR,[D]	1xTMT6plex [N-Term];1	P52948	P52948 [604-616]	P52948 1xTMT6plex [N	0,01	-0,28	0,12
[K],DVQSPGFLNEPLSSK,[S]	1xTMT6plex [K15];1xTM	Q8NG31	Q8NG31 [1073-1087]	Q8NG31 1xTMT6plex [I	0,03	-0,28	0,14
[K],VYEDSGIPLPAESPK,[K]	1xTMT6plex [K15];1xTM	Q8IXM2	Q8IXM2 [84-98]	Q8IXM2 1xTMT6plex [K	0,01	-0,28	0,12
[R],KEIIDASDK,[E]	2xTMT6plex [K1; K9];1x	Q13523	Q13523 [81-89]	Q13523 2xTMT6plex [K	0,02	-0,28	0,12
[R],SSTVTEAPIAVVTSR,[T]	1xTMT6plex [N-Term];1	Q8TD19	Q8TD19 [331-345]	Q8TD19 1xTMT6plex [N	0,02	-0,28	0,13
[R],SFSPKSPLELGEK,[L]	2xTMT6plex [K5; K13];1	Q15742	Q15742 [157-169]	Q15742 2xTMT6plex [K	0,00	-0,28	0,11
[K],TSPVVAPTSEPSSPLHTQLI	1xTMT6plex [K21];1xTM	O75044	O75044 [982-1002]	O75044 1xTMT6plex [K	0,04	-0,28	0,16
[K],TLGTVTPR,[K]	1xTMT6plex [N-Term];1	Q9BW66	Q9BW66 [5-12]	Q9BW66 1xTMT6plex [	0,04	-0,28	0,15
[R],SSSSPLVVVSVK,[S]	1xTMT6plex [K12];1xTM	Q96B01	Q96B01 [315-326]	Q96B01 1xTMT6plex [K	0,02	-0,28	0,12
[K],QALGDIPQAPHDSPPVSPT	1xTMT6plex [K21];1xTM	Q86VW2	Q86VW2 [544-564]	Q86VW2 1xTMT6plex [	0,01	-0,28	0,12
[R],KPVTVSPTTPTSPTEGEAS	1xTMT6plex [K1];1xTM	Q9Y6G9	Q9Y6G9 [505-523]	Q9Y6G9 1xTMT6plex [I	0,01	-0,28	0,12
[R],AISAPTSPTR,[L]	1xTMT6plex [N-Term];1	Q9NQG6	Q9NQG6 [53-62]	Q9NQG6 1xTMT6plex [	0,00	-0,28	0,12
[R],AISAPTSPTR,[L]	1xTMT6plex [N-Term];1	Q9NQG6	Q9NQG6 [53-62]	Q9NQG6 1xTMT6plex [	0,00	-0,28	0,12
[R],SRTPPSAPSQSR,[M]	1xTMT6plex [N-Term];1	Q9UQ35	Q9UQ35 [2407-2418]	Q9UQ35 1xTMT6plex [I	0,01	-0,28	0,12
[K],TPPGLQHEYAAPADYFR,[I]	1xTMT6plex [N-Term];1	Q9BVL2	Q9BVL2 [331-347]	Q9BVL2 1xTMT6plex [N	0,04	-0,28	0,15
[K],VSSSPQK,[E]	1xTMT6plex [K7];1xTM	Q7Z5J4	Q7Z5J4 [1189-1195]	Q7Z5J4 1xTMT6plex [K	0,03	-0,28	0,13
[R],SPSTTYLHTPTPSEDAAIPS	1xTMT6plex [K21];1xTM	Q13111	Q13111 [775-795]	Q13111 1xTMT6plex [K	0,02	-0,28	0,12
[R],SSSPFLSK,[R]	1xTMT6plex [K8];1xTM	Q9NYV4	Q9NYV4 [332-339]	Q9NYV4 1xTMT6plex [I	0,03	-0,28	0,14
[R],KASQQSNQIQTQR,[T]	1xTMT6plex [K1];1xTM	Q05D32	Q05D32 [7-19]	Q05D32 1xTMT6plex [K	0,02	-0,28	0,13
[K],AQLSVQTSPVPTPDPK,[R]	1xTMT6plex [K16];1xTM	O43426	O43426 [1338-1353]	O43426 1xTMT6plex [K	0,02	-0,28	0,13

[K],SLIKTPER,[L]	1xTMT6plex [K4];1xTM	P35658	P35658 [412-419]	P35658 1xTMT6plex [K	0,02	-0,28	0,13
[K],LSSWDQAETPGHTPSLR,[V	1xTMT6plex [N-Term];1	O75533	O75533 [215-231]	O75533 1xTMT6plex [N	0,01	-0,28	0,12
[R],DTSLGSPK,[E]	1xTMT6plex [K8];1xTM	O75140	O75140 [440-447]	O75140 1xTMT6plex [K	0,02	-0,28	0,13
[K],TAESQTPTPSATSFFSGK,[S	1xTMT6plex [K18];1xTM	P55265	P55265 [596-613]	P55265 1xTMT6plex [K	0,00	-0,28	0,11
[R],KRESESESDETPPAAPQLIK	2xTMT6plex [K1; K20];1	O60832	O60832 [448-467]	O60832 2xTMT6plex [K	0,02	-0,28	0,12
[K],MAESPCSPSGQQPPSPPS	1xCarbamidomethyl [C6	Q14160	Q14160 [1292-1318]	Q14160 1xCarbamidom	0,04	-0,28	0,15
[R],YVASVLGLTPSPR,[Q]	1xTMT6plex [N-Term];1	Q99666	Q99666 [11-23]	Q99666 1xTMT6plex [N	0,04	-0,28	0,15
[K],ATPGAPALTSMTPTAVER,[	1xTMT6plex [N-Term];1	P54198	P54198 [575-592]	P54198 1xTMT6plex [N	0,05	-0,28	0,16
[R],LSSASTGKPPLSVEDDFEK	2xTMT6plex [K8; K19];1	O75152	O75152 [757-775]	O75152 2xTMT6plex [K	0,01	-0,28	0,12
[R],LSSASTGKPPLSVEDDFEK	2xTMT6plex [K8; K19];1	O75152	O75152 [757-775]	O75152 2xTMT6plex [K	0,01	-0,28	0,12
[K],VVVHHVTVSPLR,[T]	1xTMT6plex [N-Term];1	P15822	P15822 [563-574]	P15822 1xTMT6plex [N	0,01	-0,28	0,12
[K],SPAPPLLHVAALGQK,[Q]	1xTMT6plex [K15];1xTM	Q8N163	Q8N163 [124-138]	Q8N163 1xTMT6plex [K	0,05	-0,28	0,16
[R],MDRTPPPPTLSPAAITVGR,	1xOxidation [M1];1xTMT	Q8NDX5	Q8NDX5 [606-624]	Q8NDX5 1xOxidation [M	0,01	-0,28	0,12
[K],YEDKPEPEVDALGSPPALL	2xTMT6plex [K4; K20];1	Q68DQ2	Q68DQ2 [918-937]	Q68DQ2 2xTMT6plex [I	0,04	-0,28	0,15
[K],ILLTEPPMNPTK,[N]	1xOxidation [M8];1xTMT	P61160	P61160 [107-118]	P61160 1xOxidation [M	0,02	-0,28	0,13
[R],GSLDESSLGFGYPK,[F]	1xTMT6plex [K14];1xTM	Q9BUZ4	Q9BUZ4 [425-438]	Q9BUZ4 1xTMT6plex [K	0,05	-0,28	0,16
[R],KVSSAEGAAK,[E]	2xTMT6plex [K1; K10];1	P05114	P05114 [5-14]	P05114 2xTMT6plex [K	0,04	-0,28	0,15
[R],GIASSPK,[S]	1xTMT6plex [K7];1xTM	P0DMU9	P0DMU9 [111-117]	P0DMU9 1xTMT6plex [	0,05	-0,28	0,16
[K],KVPLPGPGSPEVK,[R]	2xTMT6plex [K1; K13];1	Q8WXD9	Q8WXD9 [1249-1261]	Q8WXD9 2xTMT6plex [	0,04	-0,28	0,15
[K],KDELSDYAEK,[S]	2xTMT6plex [K1; K10];1	P46100	P46100 [1057-1066]	P46100 2xTMT6plex [K	0,00	-0,28	0,11
[K],SMSTSGHPIK,[V]	1xTMT6plex [K10];1xTM	Q9P0L2	Q9P0L2 [554-563]	Q9P0L2 1xTMT6plex [K	0,01	-0,28	0,12
[R],ITVGNDHFCVSTPER,[R]	1xCarbamidomethyl [C9	Q5VUA4	Q5VUA4 [150-164]	Q5VUA4 1xCarbamidom	0,02	-0,28	0,13
[K],KAEGSPSEGKK,[A]	3xTMT6plex [K1; K10; K	Q9P2E9	Q9P2E9 [569-579]	Q9P2E9 3xTMT6plex [K	0,00	-0,28	0,12
[R],LPEASQSPLVLK,[Q]	1xTMT6plex [K12];1xTM	Q13330	Q13330 [516-527]	Q13330 1xTMT6plex [K	0,04	-0,28	0,15
[R],VGEQDSAPTQEKPTSPGK,	2xTMT6plex [K12; K18]	Q9BX66	Q9BX66 [336-353]	Q9BX66 2xTMT6plex [K	0,02	-0,28	0,13
[R],SPLLLGAQR,[R]	1xTMT6plex [N-Term];1	Q96DR7	Q96DR7 [80-88]	Q96DR7 1xTMT6plex [N	0,01	-0,28	0,12
[K],NSERAQKSEPR,[E]	1xTMT6plex [K7];1xTM	Q9Y388	Q9Y388 [209-219]	Q9Y388 1xTMT6plex [K	0,02	-0,28	0,13
[R],SMSGGHGLR,[V]	1xTMT6plex [N-Term];1	P49815	P49815 [1130-1138]	P49815 1xTMT6plex [N	0,05	-0,28	0,16
[K],SPPTTMLLPASPAK,[A]	1xOxidation [M6];1xTMT	Q9UGU5	Q9UGU5 [502-515]	Q9UGU5 1xOxidation [M	0,01	-0,28	0,12
[K],AVTTPGKK,[G]	2xTMT6plex [K7; K8];1x	P19338	P19338 [103-110]	P19338 2xTMT6plex [K	0,04	-0,28	0,15
[K],VSPASSVDSNIPSSQGYK,[I	1xTMT6plex [K18];1xTM	Q9UHB7	Q9UHB7 [486-503]	Q9UHB7 1xTMT6plex [I	0,02	-0,28	0,13
[K],VHAYFAPVTPPPSVGGSR,	1xTMT6plex [N-Term];2	Q96IG2	Q96IG2 [409-426]	Q96IG2 1xTMT6plex [N	0,01	-0,28	0,12
[R],HSCSGSSPPR,[V]	1xCarbamidomethyl [C3	Q9UQ35	Q9UQ35 [889-898]	Q9UQ35 1xCarbamidom	0,05	-0,28	0,16
[K],TTKPPYPGSPVK,[Y]	2xTMT6plex [K3; K12];1	Q96T17	Q96T17 [307-318]	Q96T17 2xTMT6plex [K	0,00	-0,28	0,12
[K],DKSPVREPIDNLTPEER,[D]	1xTMT6plex [K2];1xTM	Q14498	Q14498 [134-150]	Q14498 1xTMT6plex [K	0,01	-0,28	0,12
[R],SSSSDSIHSVR,[G]	1xTMT6plex [N-Term];1	Q8WYL5	Q8WYL5 [935-945]	Q8WYL5 1xTMT6plex [	0,00	-0,28	0,12
[R],ELSPQHK,[R]	1xTMT6plex [K7];1xTM	Q86V48	Q86V48 [392-398]	Q86V48 1xTMT6plex [K	0,01	-0,28	0,12
[R],ALSGGGSVASPPPSPAMPI	1xOxidation [M17];1xTM	O75420	O75420 [15-33]	O75420 1xOxidation [M	0,01	-0,27	0,12
[R],LEPVYSPPGSPPPGDPR,[I]	1xTMT6plex [N-Term];1	O75676	O75676 [338-354]	O75676 1xTMT6plex [N	0,03	-0,27	0,14
[K],TLPGGAGK,[G]	1xTMT6plex [K8];1xTM	Q9H9B1	Q9H9B1 [154-161]	Q9H9B1 1xTMT6plex [K	0,04	-0,27	0,16
[K],NYVPSYDPSSPQTSQSWY	1xPhospho [S9];1xTMT	O00712	O00712 [401-420]	O00712 1xPhospho [S4	0,01	-0,27	0,12
[R],GAAAAATGQPGTAPAGTP(	1xTMT6plex [K30];1xTM	Q13263	Q13263 [525-554]	Q13263 1xTMT6plex [K	0,01	-0,27	0,12
[K],SKPNLPSESRSR,[S]	1xTMT6plex [K2];1xTM	Q08170	Q08170 [450-461]	Q08170 1xTMT6plex [K	0,05	-0,27	0,16
[R],KYSDSSLPPSNSGK,[I]	2xTMT6plex [K1; K14];1	Q68CP9	Q68CP9 [1298-1311]	Q68CP9 2xTMT6plex [K	0,00	-0,27	0,11
[K],DSSSVVEWTQAPK,[E]	1xTMT6plex [K13];1xTM	Q8TC07	Q8TC07 [69-81]	Q8TC07 1xTMT6plex [K	0,03	-0,27	0,14
[R],AMSLNTLNVDAPR,[A]	1xOxidation [M2];1xTMT	Q6P597	Q6P597 [464-476]	Q6P597 1xOxidation [M	0,00	-0,27	0,11
[K],SVLGSSEDEK,[T]	1xTMT6plex [K10];1xTM	Q9NPC8	Q9NPC8 [207-216]	Q9NPC8 1xTMT6plex [I	0,02	-0,27	0,12
[K],LGYLVSPPQQIR,[R]	1xTMT6plex [N-Term];1	Q7Z6E9	Q7Z6E9 [511-522]	Q7Z6E9 1xTMT6plex [N	0,01	-0,27	0,12
[R],VSSSPGVK,[G]	1xTMT6plex [K8];1xTM	P32519	P32519 [331-338]	P32519 1xTMT6plex [K	0,04	-0,27	0,16
[R],TPGHPPPPEIPSELGACDFI	1xCarbamidomethyl [C1	Q92538	Q92538 [1750-1775]	Q92538 1xCarbamidom	0,01	-0,27	0,12
[K],AGPSAQEPGSQTPLK,[S]	1xTMT6plex [K15];1xTM	O60271	O60271 [1253-1267]	O60271 1xTMT6plex [K	0,01	-0,27	0,12
[K],AILLPDLSEPNNEPLFSPAS	1xTMT6plex [N-Term];1	Q8WYP5	Q8WYP5 [2104-2127]	Q8WYP5 1xTMT6plex [	0,04	-0,27	0,16
[K],IISPGSSTPSSTRSPPPGR,[	1xTMT6plex [N-Term];2	Q5VT52	Q5VT52 [756-774]	Q5VT52 1xTMT6plex [N	0,01	-0,27	0,12
[K],VVVTTPLHR,[D]	1xTMT6plex [N-Term];1	P10244	P10244 [483-491]	P10244 1xTMT6plex [N	0,00	-0,27	0,12
[R],QLSMTLR,[G]	1xOxidation [M4];1xTMT	Q00536	Q00536 [10-16]	Q00536 1xOxidation [M	0,04	-0,27	0,15
[R],IPSPLQPEMQGTPDDEPSE	1xTMT6plex [N-Term];1	Q96GY3	Q96GY3 [180-209]	Q96GY3 1xTMT6plex [N	0,04	-0,27	0,15
[K],VASEAPLEHKPQVEASSPR	1xTMT6plex [K10];1xTM	Q8TD19	Q8TD19 [853-871]	Q8TD19 1xTMT6plex [K	0,03	-0,27	0,14

[R],KSFSQMISEK,[Q]	2xTMT6plex [K1; K10];1	Q13459	Q13459 [1042-1051]	Q13459 2xTMT6plex [K	0,04	-0,27	0,15
[R],RHSSDINHLVTQGRESPEG	1xTMT6plex [N-Term];2	Q5T0N5	Q5T0N5 [486-515]	Q5T0N5 1xTMT6plex [N	0,01	-0,27	0,12
[R],VDSTTCLFPVEEK,[A]	1xCarbamidomethyl [C6	Q06210	Q06210 [259-271]	Q06210 1xCarbamidom	0,04	-0,27	0,16
[R],SPGSGSQSSGWHEVEPGM	1xTMT6plex [K25];1xTM	Q12857	Q12857 [300-324]	Q12857 1xTMT6plex [K	0,00	-0,27	0,11
[R],FSVSPSSPSSQQTPPPVTP	1xTMT6plex [N-Term];1	Q14185	Q14185 [1755-1774]	Q14185 1xTMT6plex [N	0,03	-0,27	0,14
[R],GSITQGIPR,[S]	1xTMT6plex [N-Term];1	Q9Y618	Q9Y618 [1353-1361]	Q9Y618 1xTMT6plex [N	0,02	-0,27	0,13
[R],ALSPLPTR,[T]	1xTMT6plex [N-Term];1	Q86UU1	Q86UU1 [468-475]	Q86UU1 1xTMT6plex [N	0,04	-0,27	0,16
[R],KPLPTAAAQCSFEDPDSAV	1xCarbamidomethyl [C1	O14795	O14795 [166-192]	O14795 1xCarbamidom	0,05	-0,27	0,16
[K],SLPDSLAEGMTK,[M]	1xOxidation [M10];1xTM	Q9Y5W8	Q9Y5W8 [720-731]	Q9Y5W8 1xOxidation [M	0,00	-0,27	0,11
[R],LGTPTEPTTPK,[A]	1xTMT6plex [K11];1xTM	O14559	O14559 [556-566]	O14559 1xTMT6plex [K	0,02	-0,27	0,13
[K],EKSPPDQPAVPHPPPSTPII	2xTMT6plex [K2; K20];1	P35611	P35611 [598-617]	P35611 2xTMT6plex [K	0,03	-0,27	0,14
[R],ASLTGTPPR,[A]	1xTMT6plex [N-Term];1	Q9UF83	Q9UF83 [370-378]	Q9UF83 1xTMT6plex [N	0,00	-0,27	0,12
[R],SGSGISVISSTSVDQR,[L]	1xTMT6plex [N-Term];1	Q15811	Q15811 [313-328]	Q15811 1xTMT6plex [N	0,03	-0,27	0,13
[R],RKDSEEEAASPEGK,[R]	2xTMT6plex [K2; K14];1	Q9HB96	Q9HB96 [201-214]	Q9HB96 2xTMT6plex [K	0,02	-0,27	0,13
[R],SPGLVPPSPEFAPR,[S]	1xTMT6plex [N-Term];1	Q9H5H4	Q9H5H4 [90-103]	Q9H5H4 1xTMT6plex [N	0,01	-0,27	0,12
[R],EYSPPYAPSHQQYPPSHAT	1xTMT6plex [K23];1xTM	Q9UPT8	Q9UPT8 [157-179]	Q9UPT8 1xTMT6plex [K	0,03	-0,27	0,14
[K],SVDIHDSIQPR,[S]	1xTMT6plex [N-Term];1	Q9Y6D6	Q9Y6D6 [1569-1579]	Q9Y6D6 1xTMT6plex [N	0,00	-0,27	0,12
[K],VVGSMPTAGSAGSVPENLN	1xOxidation [M5];1xTMT	Q8WU79	Q8WU79 [228-254]	Q8WU79 1xOxidation [I	0,01	-0,27	0,12
[K],SAQGTGFELGQLQSIR,[S]	1xTMT6plex [N-Term];1	Q9C0B5	Q9C0B5 [432-447]	Q9C0B5 1xTMT6plex [N	0,03	-0,27	0,15
[R],GMYDGPVFDLTTTPKGGTP	1xTMT6plex [K15];1xTM	Q14195	Q14195 [497-520]	Q14195 1xTMT6plex [K	0,03	-0,27	0,14
[R],SPSPAHLSNK,[V]	1xTMT6plex [K10];1xTM	Q9HCS4	Q9HCS4 [169-178]	Q9HCS4 1xTMT6plex [I	0,04	-0,27	0,15
[R],AMTMPPVSPVGAEGPVVLI	1xOxidation [M4];1xTMT	O15020	O15020 [2352-2370]	O15020 1xOxidation [M	0,04	-0,27	0,15
[R],STSTPNVHMVSTTLPVDSR	1xOxidation [M9];1xTMT	P04049	P04049 [257-275]	P04049 1xOxidation [M	0,00	-0,27	0,11
[K],ALLSSPEGEEK,[V]	1xTMT6plex [K11];1xTM	O75592	O75592 [3928-3938]	O75592 1xTMT6plex [K	0,04	-0,27	0,15
[R],EISSSPTSK,[N]	1xTMT6plex [K9];1xTM	Q9UQ35	Q9UQ35 [452-460]	Q9UQ35 1xTMT6plex [I	0,02	-0,27	0,12
[K],TIGGGDDSFNTFFSETGAG	1xTMT6plex [K20];1xTM	Q71U36	Q71U36 [41-60]	Q71U36 1xTMT6plex [K	0,02	-0,27	0,12
[R],GGSDTNLNFDVPDGILDFH	1xTMT6plex [K20];1xTM	Q9ULS5	Q9ULS5 [44-63]	Q9ULS5 1xTMT6plex [K	0,05	-0,27	0,16
[R],QILQETIHNFHSSFESSASN	1xTMT6plex [N-Term];1	Q68DC2	Q68DC2 [842-863]	Q68DC2 1xTMT6plex [N	0,01	-0,27	0,12
[R],LSGSKNLEKAIQIMYQNLQ	2xMethyl [K5; K9];1xTM	Q9Y646	Q9Y646 [78-102]	Q9Y646 2xMethyl [K82;	0,01	-0,27	0,12
[R],ASPAPGSGHPEGPGAHLD	1xOxidation [M19];1xTM	O94826	O94826 [90-113]	O94826 1xOxidation [M	0,01	-0,27	0,12
[K],SLSSSPDKR,[E]	1xTMT6plex [K8];1xTM	Q9BVI0	Q9BVI0 [155-163]	Q9BVI0 1xTMT6plex [K	0,03	-0,27	0,14
[R],TNKPSTPTTATR,[K]	1xTMT6plex [K3];1xTM	Q9UBP0	Q9UBP0 [298-309]	Q9UBP0 1xTMT6plex [I	0,04	-0,27	0,16
[R],GLNSSFETSPK,[K]	1xTMT6plex [K11];1xTM	Q8IV63	Q8IV63 [51-61]	Q8IV63 1xTMT6plex [K	0,03	-0,27	0,14
[K],SSSPLPTVQLHPQSPTAGK	1xTMT6plex [K19];1xTM	O60245	O60245 [998-1016]	O60245 1xTMT6plex [K	0,01	-0,27	0,12
[K],SPCGLTEQYLHK,[D]	1xCarbamidomethyl [C3	P78559	P78559 [1172-1183]	P78559 1xCarbamidom	0,02	-0,27	0,13
[R],KPSGDSQPSSPR,[Y]	1xTMT6plex [K1];1xTM	Q9C0H5	Q9C0H5 [294-305]	Q9C0H5 1xTMT6plex [K	0,00	-0,27	0,12
[R],IPGGNIYISPLK,[S]	1xTMT6plex [K12];1xTM	P06400	P06400 [799-810]	P06400 1xTMT6plex [K	0,03	-0,27	0,13
[K],RSSGFISELPSEEGKK,[L]	2xTMT6plex [K15; K16]	Q5VZK9	Q5VZK9 [966-981]	Q5VZK9 2xTMT6plex [K	0,02	-0,27	0,13
[K],ATPKLDSSPSVSSTLAAK,[D	2xTMT6plex [K4; K18];1	Q13428	Q13428 [1221-1238]	Q13428 2xTMT6plex [K	0,02	-0,27	0,13
[R],GPPNSPDLK,[L]	1xTMT6plex [K9];1xTM	Q6ZSZ5	Q6ZSZ5 [944-952]	Q6ZSZ5 1xTMT6plex [K	0,00	-0,26	0,12
[R],SWHEESPFEK,[Q]	1xTMT6plex [K10];1xTM	Q9BY84	Q9BY84 [612-621]	Q9BY84 1xTMT6plex [K	0,02	-0,26	0,12
[R],DPLPSAPLPDPPAPAESPK	1xTMT6plex [K19];1xTM	A0A0U1R	A0A0U1RRI6 [89-107]	A0A0U1RRI6 1xTMT6p	0,04	-0,26	0,16
[R],DEPTGEVLSLVGK,[L]	1xTMT6plex [K13];1xTM	O75643	O75643 [34-46]	O75643 1xTMT6plex [K	0,05	-0,26	0,16
[K],IDHLSSSAPGSPPDLLESVP	1xTMT6plex [K21];1xTM	Q5TC82	Q5TC82 [525-545]	Q5TC82 1xTMT6plex [K	0,02	-0,26	0,13
[R],RPSSTSVPLGDK,[G]	1xTMT6plex [K12];1xTM	Q9NXR1	Q9NXR1 [304-315]	Q9NXR1 1xTMT6plex [I	0,02	-0,26	0,13
[R],RVSFNEAALFEQSR,[K]	1xTMT6plex [N-Term];1	Q8N350	Q8N350 [131-144]	Q8N350 1xTMT6plex [N	0,01	-0,26	0,12
[R],ASPAPGSGHPEGPGAHLD	1xTMT6plex [N-Term];1	O94826	O94826 [90-113]	O94826 1xTMT6plex [N	0,04	-0,26	0,16
[R],FQDVGPQAPVGSVYQK,[T]	1xTMT6plex [K16];1xTM	Q9UJU6	Q9UJU6 [149-164]	Q9UJU6 1xTMT6plex [K	0,01	-0,26	0,12
[R],IPNYQLSPTK,[L]	1xTMT6plex [K10];1xTM	O60934	O60934 [426-435]	O60934 1xTMT6plex [K	0,05	-0,26	0,16
[R],LMSGSSR,[S]	1xTMT6plex [N-Term];1	Q7Z6J6	Q7Z6J6 [5-11]	Q7Z6J6 1xTMT6plex [N	0,02	-0,26	0,13
[R],SSPPLQPPPVNSLTTENR,[	1xTMT6plex [N-Term];1	Q9NQC7	Q9NQC7 [398-415]	Q9NQC7 1xTMT6plex [	0,04	-0,26	0,15
[R],FQSVESGANNVVFIR,[T]	1xTMT6plex [N-Term];1	Q9NXG2	Q9NXG2 [117-131]	Q9NXG2 1xTMT6plex [	0,03	-0,26	0,14
[R],RLAAAASPHSGGR,[A]	1xTMT6plex [N-Term];1	O95613	O95613 [3268-3280]	O95613 1xTMT6plex [N	0,01	-0,26	0,12
[R],SRSFSSSAEEHS,[-]	1xTMT6plex [N-Term];1	Q96EQ0	Q96EQ0 [293-304]	Q96EQ0 1xTMT6plex [N	0,01	-0,26	0,12
[R],GESPPTPPGQPPISPK,[K]	1xTMT6plex [K16];1xTM	Q92835	Q92835 [958-973]	Q92835 1xTMT6plex [K	0,04	-0,26	0,16
[R],SLSPNHNTLQTLK,[S]	1xTMT6plex [K13];1xTM	O75592	O75592 [2787-2799]	O75592 1xTMT6plex [K	0,03	-0,26	0,14
[K],ISHLSGSGSGDER,[V]	1xTMT6plex [N-Term];1	Q96T37	Q96T37 [204-216]	Q96T37 1xTMT6plex [N	0,01	-0,26	0,12

[R],YLSVPPSPNISTSESR,[S]	1xTMT6plex [N-Term];1	Q9BTC0	Q9BTC0 [1024-1039]	Q9BTC0 1xTMT6plex [N	0,02	-0,26	0,13
[R],RGSISSMSSVSSVLDEK,[D]	1xTMT6plex [K17];1xTM	Q9H1K0	Q9H1K0 [228-244]	Q9H1K0 1xTMT6plex [K	0,00	-0,26	0,11
[R],IKTEPSSPLSDPSDIIR,[V]	1xTMT6plex [K2];1xTM	Q9ULJ3	Q9ULJ3 [429-445]	Q9ULJ3 1xTMT6plex [K	0,01	-0,26	0,12
[R],IVAHAVEVPAVQSPR,[R]	1xTMT6plex [N-Term];1	Q96FF9	Q96FF9 [63-77]	Q96FF9 1xTMT6plex [N	0,00	-0,26	0,12
[R],YAALSLSETSLTEK,[G]	1xTMT6plex [K14];1xTM	C9J069	C9J069 [503-516]	C9J069 1xTMT6plex [K	0,01	-0,26	0,12
[R],SKSPVGNPQLIQFSR,[E]	1xTMT6plex [K2];1xTM	Q9Y4F3	Q9Y4F3 [1091-1105]	Q9Y4F3 1xTMT6plex [K	0,00	-0,26	0,12
[R],RFSLSPSLGPQASR,[F]	1xTMT6plex [N-Term];2	Q9NYF3	Q9NYF3 [230-243]	Q9NYF3 1xTMT6plex [N	0,04	-0,26	0,15
[R],IFSQDSLCSQENYIIDK,[R]	1xCarbamidomethyl [C8	Q15032	Q15032 [297-313]	Q15032 1xCarbamidom	0,03	-0,26	0,13
[K],DVPPLSETEATPVPIK,[D]	1xTMT6plex [K16];1xTM	P27816	P27816 [575-590]	P27816 1xTMT6plex [K	0,00	-0,26	0,12
[K],ALVATPGK,[K]	1xTMT6plex [K8];1xTM	P19338	P19338 [117-124]	P19338 1xTMT6plex [K	0,04	-0,26	0,15
[R],APSPGDYK,[S]	1xTMT6plex [K8];1xTM	P55198	P55198 [421-428]	P55198 1xTMT6plex [K	0,00	-0,26	0,12
[R],STSPAGQHHSPISSR,[H]	1xTMT6plex [N-Term];1	Q5T200	Q5T200 [316-330]	Q5T200 1xTMT6plex [N	0,03	-0,26	0,14
[K],DLTGQVPTPVVK,[Q]	1xTMT6plex [K12];1xTM	P46821	P46821 [520-531]	P46821 1xTMT6plex [K	0,00	-0,26	0,12
[K],ELLHSVHPESPNLK,[T]	1xTMT6plex [K14];1xTM	Q9Y217	Q9Y217 [552-565]	Q9Y217 1xTMT6plex [K	0,00	-0,26	0,11
[K],TKESLDVSR,[L]	1xTMT6plex [K2];1xTM	Q32P28	Q32P28 [436-444]	Q32P28 1xTMT6plex [K	0,04	-0,26	0,16
[K],ALVSVVNSFHEK,[I]	1xMethyl [K12];1xTMT6	Q8NF91	Q8NF91 [5147-5158]	Q8NF91 1xMethyl [K51	0,04	-0,26	0,15
[R],SPSPQFAPQK,[L]	1xTMT6plex [K10];1xTM	Q13796	Q13796 [1171-1180]	Q13796 1xTMT6plex [K	0,02	-0,26	0,13
[R],VYENVGLMQQQK,[S]	1xTMT6plex [K12];1xTM	Q06124	Q06124 [579-590]	Q06124 1xTMT6plex [K	0,00	-0,26	0,11
[R],ASSPSPPGR,[F]	1xTMT6plex [N-Term];1	Q86X51	Q86X51 [408-416]	Q86X51 1xTMT6plex [N	0,03	-0,26	0,13
[K],NSQEDSEDSEDKDVKTK,[K	3xTMT6plex [K12; K15;	Q9H1E3	Q9H1E3 [53-69]	Q9H1E3 3xTMT6plex [K	0,02	-0,26	0,13
[K],NSQEDSEDSEDKDVKTK,[K	3xTMT6plex [K12; K15;	Q9H1E3	Q9H1E3 [53-69]	Q9H1E3 3xTMT6plex [K	0,02	-0,26	0,13
[R],KTEIIVPEAEK,[E]	2xTMT6plex [K1; K11];1	Q8N3D4	Q8N3D4 [868-878]	Q8N3D4 2xTMT6plex [K	0,04	-0,26	0,16
[R],LAQTTPVDSALGSSR,[H]	1xTMT6plex [N-Term];1	Q2KHR2	Q2KHR2 [887-901]	Q2KHR2 1xTMT6plex [N	0,01	-0,26	0,12
[K],QKSPDGVHR,[V]	1xTMT6plex [K2];1xTM	Q9UMN6	Q9UMN6 [1162-1170]	Q9UMN6 1xTMT6plex [	0,01	-0,26	0,12
[R],QASTDAGTAGALTPQHVR,	1xTMT6plex [N-Term];1	P46937	P46937 [107-124]	P46937 1xTMT6plex [N	0,01	-0,26	0,12
[R],LLPPASP,[-]	1xTMT6plex [N-Term];1	Q9Y4R8	Q9Y4R8 [831-837]	Q9Y4R8 1xTMT6plex [N	0,01	-0,26	0,12
[R],TMTTNSSDPFLNSGTYHSR	1xTMT6plex [N-Term];1	P46937	P46937 [376-394]	P46937 1xTMT6plex [N	0,02	-0,26	0,13
[R],TMTTNSSDPFLNSGTYHSR	1xTMT6plex [N-Term];1	P46937	P46937 [376-394]	P46937 1xTMT6plex [N	0,02	-0,26	0,13
[K],YLCIPAADSPSQNLTR,[H]	1xCarbamidomethyl [C3	Q9NRW4	Q9NRW4 [50-65]	Q9NRW4 1xCarbamido	0,02	-0,26	0,13
[R],SSTSSIDSNISSK,[S]	1xTMT6plex [K13];1xTM	Q8IVL1	Q8IVL1 [1232-1244]	Q8IVL1 1xTMT6plex [K	0,02	-0,26	0,13
[R],GPASCSSLSK,[T]	1xCarbamidomethyl [C5	Q15052	Q15052 [549-558]	Q15052 1xCarbamidom	0,04	-0,26	0,15
[R],SASWGSTDQLK,[E]	1xTMT6plex [K11];1xTM	Q6P1L5	Q6P1L5 [271-281]	Q6P1L5 1xTMT6plex [K	0,03	-0,26	0,14
[K],AEDGATPSPSNETPK,[K]	1xTMT6plex [K15];1xTM	P29966	P29966 [138-152]	P29966 1xTMT6plex [K	0,02	-0,26	0,13
[K],SPPLSPVGTTPVK,[L]	1xTMT6plex [K13];1xTM	Q9BVC5	Q9BVC5 [189-201]	Q9BVC5 1xTMT6plex [	0,03	-0,25	0,14
[K],DAHLSPK,[R]	1xTMT6plex [K7];1xTM	O60503	O60503 [1303-1309]	O60503 1xTMT6plex [K	0,03	-0,25	0,14
[R],AISTPEK,[S]	1xTMT6plex [K7];1xTM	P42695	P42695 [1412-1418]	P42695 1xTMT6plex [K	0,04	-0,25	0,15
[R],ADSGPTQPPLSLSPAPETK	1xTMT6plex [K19];1xTM	O15027	O15027 [2071-2089]	O15027 1xTMT6plex [K	0,01	-0,25	0,12
[R],NHGYASPLK,[G]	1xTMT6plex [K9];1xTM	Q9Y5R6	Q9Y5R6 [81-89]	Q9Y5R6 1xTMT6plex [K	0,04	-0,25	0,15
[R],SQSSSSVLINK,[S]	1xTMT6plex [K11];1xTM	Q9ULL1	Q9ULL1 [1181-1191]	Q9ULL1 1xTMT6plex [K	0,01	-0,25	0,12
[R],LHSSNPNLSTLDFGEEK,[N]	1xTMT6plex [K17];1xTM	Q9H4L5	Q9H4L5 [301-317]	Q9H4L5 1xTMT6plex [K	0,00	-0,25	0,12
[K],IKPTGLLTIPSPQI,[-]	1xTMT6plex [K2];1xTM	Q8TEA7	Q8TEA7 [880-893]	Q8TEA7 1xTMT6plex [K	0,04	-0,25	0,15
[K],SLVMHTPPVLK,[K]	1xTMT6plex [K11];1xTM	P46013	P46013 [538-548]	P46013 1xTMT6plex [K	0,04	-0,25	0,15
[K],SSPSVKPAVDPAAAK,[L]	2xTMT6plex [K6; K15];1	Q6FI81	Q6FI81 [182-196]	Q6FI81 2xTMT6plex [K	0,01	-0,25	0,12
[K],HHLDPVPPAGNSSPTEALK	1xTMT6plex [K19];1xTM	P52746	P52746 [999-1017]	P52746 1xTMT6plex [K	0,02	-0,25	0,12
[R],YVGFGNTPPPQKK,[E]	2xTMT6plex [K12; K13]	Q8N6T3	Q8N6T3 [183-195]	Q8N6T3 2xTMT6plex [K	0,04	-0,25	0,15
[K],VQPPTPLLPSVK,[V]	1xTMT6plex [K12];1xTM	O60885	O60885 [938-949]	O60885 1xTMT6plex [K	0,03	-0,25	0,14
[K],IATQSQITPPGTPSSALSSG	1xTMT6plex [N-Term];1	Q8N0Z3	Q8N0Z3 [228-250]	Q8N0Z3 1xTMT6plex [N	0,01	-0,25	0,12
[R],SSTPLHSPSPIR,[V]	1xTMT6plex [N-Term];1	O95817	O95817 [283-294]	O95817 1xTMT6plex [N	0,03	-0,25	0,14
[R],RQSPSPSTRPIR,[R]	1xTMT6plex [N-Term];2	Q8IYB3	Q8IYB3 [711-722]	Q8IYB3 1xTMT6plex [N	0,03	-0,25	0,14
[K],FSPPSPLPQAVFSTSSR,[F]	1xTMT6plex [N-Term];1	Q99594	Q99594 [144-160]	Q99594 1xTMT6plex [N	0,02	-0,25	0,13
[K],SLKESEQESEEEILAQK,[K]	2xTMT6plex [K3; K17];1	Q9BW71	Q9BW71 [219-235]	Q9BW71 2xTMT6plex [	0,02	-0,25	0,13
[R],SDVFPGPSFR,[S]	1xTMT6plex [N-Term];1	Q8IX01	Q8IX01 [82-91]	Q8IX01 1xTMT6plex [N	0,02	-0,25	0,13
[R],RPHFPQFSYSASGRE,[-]	1xTMT6plex [N-Term];1	Q9Y243	Q9Y243 [465-479]	Q9Y243 1xTMT6plex [N	0,00	-0,25	0,11
[K],IGSPSPSPSSLSDSSSSFGK	1xTMT6plex [K20];1xTM	Q8IXZ3	Q8IXZ3 [9-28]	Q8IXZ3 1xTMT6plex [K	0,02	-0,25	0,13
[K],GEHPGLSIGDVAK,[K]	1xTMT6plex [K13];1xTM	P09429	P09429 [115-127]	P09429 1xTMT6plex [K	0,02	-0,25	0,13
[R],DPEDRIPSPLGYAAIPIK,[F]	1xTMT6plex [K18];1xTM	Q9Y3A4	Q9Y3A4 [12-29]	Q9Y3A4 1xTMT6plex [K	0,01	-0,25	0,12
[R],FSCPPNFTAKPPASESPR,[	1xCarbamidomethyl [C3	O15446	O15446 [12-29]	O15446 1xCarbamidom	0,04	-0,25	0,15

[K],AYSPTCSPTLGFK,[E]	1xCarbamidomethyl [C6	Q5TGY3	Q5TGY3 [1397-1409]	Q5TGY3 1xCarbamidom	0,02	-0,25	0,13
[R],TVIADGSLSHPK,[E]	1xTMT6plex [K12];1xTM	Q8IXS6	Q8IXS6 [222-233]	Q8IXS6 1xTMT6plex [K	0,01	-0,25	0,12
[R],AGAPHALAPHPDRFSHDR,	1xTMT6plex [N-Term];1	Q9H9J4	Q9H9J4 [1119-1136]	Q9H9J4 1xTMT6plex [N	0,01	-0,25	0,12
[R],ASLGAGDPLSPLHPARPLQ	1xTMT6plex [N-Term];1	P61371	P61371 [139-166]	P61371 1xTMT6plex [N	0,03	-0,25	0,14
[K],STVLQQQYNR,[V]	1xTMT6plex [N-Term];1	Q9Y490	Q9Y490 [429-438]	Q9Y490 1xTMT6plex [N	0,02	-0,25	0,13
[R],RNSSEASSGDFLDLK,[G]	1xTMT6plex [K15];1xTM	Q9UK76	Q9UK76 [85-99]	Q9UK76 1xTMT6plex [K	0,00	-0,25	0,12
[K],RLSTIDELDELFPSR,[D]	1xTMT6plex [N-Term];1	Q8IW52	Q8IW52 [739-753]	Q8IW52 1xTMT6plex [N	0,04	-0,25	0,15
[K],AVASSPAATNSEVK,[M]	1xTMT6plex [K14];1xTM	Q9P1Y5	Q9P1Y5 [550-563]	Q9P1Y5 1xTMT6plex [K	0,00	-0,25	0,12
[K],AVASSPAATNSEVK,[M]	1xTMT6plex [K14];1xTM	Q9P1Y5	Q9P1Y5 [550-563]	Q9P1Y5 1xTMT6plex [K	0,00	-0,25	0,12
[R],THFPQFSYSASIRE,[-]	1xTMT6plex [N-Term];1	P31751	P31751 [468-481]	P31751 1xTMT6plex [N	0,01	-0,25	0,12
[K],ATDIPASASPPPVAGVPFFK	1xTMT6plex [K20];1xTM	Q8N1G0	Q8N1G0 [245-264]	Q8N1G0 1xTMT6plex [	0,05	-0,25	0,16
[K],AKSPTPSPSPPR,[N]	1xTMT6plex [K2];1xTM	Q8IYB3	Q8IYB3 [789-800]	Q8IYB3 1xTMT6plex [K	0,03	-0,25	0,14
[R],YHGHSMSDPGVSYR,[T]	1xOxidation [M6];1xTM	P08559	P08559 [289-302]	P08559 1xOxidation [M	0,02	-0,25	0,13
[K],KKPGDASSLPDAGLSPGSQ	3xTMT6plex [K1; K2; K	Q13459	Q13459 [1391-1413]	Q13459 3xTMT6plex [K	0,04	-0,25	0,15
[K],VAQRSPVDSGTILR,[E]	1xTMT6plex [N-Term];1	P49454	P49454 [3050-3063]	P49454 1xTMT6plex [N	0,01	-0,25	0,12
[R],GHVSPAEDQTIQAPPVSVS	1xTMT6plex [N-Term];1	Q92622	Q92622 [263-285]	Q92622 1xTMT6plex [N	0,04	-0,25	0,15
[R],ASPLSSDSPVK,[T]	1xTMT6plex [K11];1xTM	Q9Y426	Q9Y426 [434-444]	Q9Y426 1xTMT6plex [K	0,00	-0,25	0,11
[R],ASPLSSDSPVK,[T]	1xTMT6plex [K11];1xTM	Q9Y426	Q9Y426 [434-444]	Q9Y426 1xTMT6plex [K	0,00	-0,25	0,11
[R],KVPDFLGSPGAEGK,[D]	2xTMT6plex [K1; K14];1	Q96RY7	Q96RY7 [353-366]	Q96RY7 2xTMT6plex [K	0,01	-0,25	0,12
[R],SLTAHSLLPLAEK,[Q]	1xTMT6plex [K13];1xTM	Q86VI3	Q86VI3 [1424-1436]	Q86VI3 1xTMT6plex [K	0,05	-0,25	0,16
[K],APVHFVEPLSPTGVAGHR,[	1xTMT6plex [N-Term];1	Q5T5Y3	Q5T5Y3 [1071-1088]	Q5T5Y3 1xTMT6plex [N	0,02	-0,25	0,13
[K],GIGTPPNTTPIK,[N]	1xTMT6plex [K12];1xTM	O96028	O96028 [107-118]	O96028 1xTMT6plex [K	0,04	-0,25	0,16
[K],SQSLTNAFNLPEPAPPRPS	1xTMT6plex [K26];1xTM	P17600	P17600 [663-688]	P17600 1xTMT6plex [K	0,00	-0,25	0,12
[K],RQKETSPR,[G]	1xTMT6plex [K3];1xTM	Q8IYB3	Q8IYB3 [536-543]	Q8IYB3 1xTMT6plex [K	0,02	-0,25	0,13
[R],TGSSPTQGIVNK,[A]	1xTMT6plex [K12];1xTM	Q9UPT6	Q9UPT6 [362-373]	Q9UPT6 1xTMT6plex [K	0,03	-0,25	0,14
[K],KDNQESSDAELSSSEYIK,[T	2xTMT6plex [K1; K18];1	Q96JM3	Q96JM3 [621-638]	Q96JM3 2xTMT6plex [K	0,01	-0,24	0,12
[K],GLGGSGGAGGPPGTPYEL	1xTMT6plex [K20];1xTM	Q9ULL5	Q9ULL5 [673-692]	Q9ULL5 1xTMT6plex [K	0,04	-0,24	0,15
[R],VLHSPPAVR,[R]	1xTMT6plex [N-Term];1	Q9Y4B5	Q9Y4B5 [1511-1519]	Q9Y4B5 1xTMT6plex [N	0,01	-0,24	0,12
[K],VSEPVEIGIQVTPDEDDHLL	1xTMT6plex [K24];1xTM	Q9C0K0	Q9C0K0 [109-132]	Q9C0K0 1xTMT6plex [K	0,02	-0,24	0,13
[R],TLSSSSMDLSR,[R]	1xTMT6plex [N-Term];1	Q9H0B6	Q9H0B6 [606-616]	Q9H0B6 1xTMT6plex [N	0,03	-0,24	0,14
[K],SPGSTPTTPTSSQAPQK,[L	1xTMT6plex [K17];1xTM	P35658	P35658 [430-446]	P35658 1xTMT6plex [K	0,03	-0,24	0,14
[K],ATSSSNPSSPAPDWYK,[D]	1xTMT6plex [K16];1xTM	P46100	P46100 [1988-2003]	P46100 1xTMT6plex [K	0,01	-0,24	0,12
[R],APSGHLAPSPPAFDGELDL	1xTMT6plex [N-Term];1	Q9P2Y4	Q9P2Y4 [8-28]	Q9P2Y4 1xTMT6plex [N	0,02	-0,24	0,12
[K],KNSETSILQAMSR,[G]	1xTMT6plex [K1];1xTM	Q659C4	Q659C4 [346-358]	Q659C4 1xTMT6plex [K	0,02	-0,24	0,13
[K],VPAQANGTPTTK,[S]	1xTMT6plex [K12];1xTM	Q9BQG0	Q9BQG0 [1220-1231]	Q9BQG0 1xTMT6plex [	0,02	-0,24	0,13
[K],RKSLSDSESDDSK,[S]	2xTMT6plex [K2; K13];1	Q13185	Q13185 [91-103]	Q13185 2xTMT6plex [K	0,01	-0,24	0,12
[K],TDLPEEKPDATPQNPFLK,[S	2xTMT6plex [K7; K18];1	A1X283	A1X283 [620-637]	A1X283 2xTMT6plex [K	0,03	-0,24	0,13
[R],DLSSSPPGPYGQEMYAFR,	1xOxidation [M14];1xTM	O43741	O43741 [180-197]	O43741 1xOxidation [M	0,04	-0,24	0,15
[R],DLSSSPPGPYGQEMYAFR,	1xOxidation [M14];1xTM	O43741	O43741 [180-197]	O43741 1xOxidation [M	0,04	-0,24	0,15
[R],RKSPLQDPFPEEDYSSTEG	1xTMT6plex [K2];1xTM	P48551	P48551 [398-420]	P48551 1xTMT6plex [K	0,00	-0,24	0,12
[K],RKEDNLSLSCVIGSSGSFSG	1xCarbamidomethyl [C1	Q6ZNL6	Q6ZNL6 [578-598]	Q6ZNL6 1xCarbamidom	0,03	-0,24	0,13
[K],SHSLDLNISEK,[L]	1xTMT6plex [K11];1xTM	Q86T96	Q86T96 [228-238]	Q86T96 1xTMT6plex [K	0,02	-0,24	0,12
[R],CSTPLLHQQYTSR,[T]	1xCarbamidomethyl [C1	Q9HCK8	Q9HCK8 [1980-1992]	Q9HCK8 1xCarbamidom	0,02	-0,24	0,13
[K],SGPISPGIHSCDLTVEGLAT	1xCarbamidomethyl [C1	Q9BUB4	Q9BUB4 [223-245]	Q9BUB4 1xCarbamidom	0,01	-0,24	0,12
[K],ATDGDLAQEPGPGLTFEDS	2xTMT6plex [K23; K27]	O15417	O15417 [2520-2546]	O15417 2xTMT6plex [K	0,03	-0,24	0,14
[K],EISPSVTK,[K]	1xTMT6plex [K8];1xTM	Q8N488	Q8N488 [97-104]	Q8N488 1xTMT6plex [K	0,04	-0,24	0,15
[R],LNQPGTPTR,[T]	1xTMT6plex [N-Term];1	P36507	P36507 [389-397]	P36507 1xTMT6plex [N	0,01	-0,24	0,12
[K],SPSLSPSPPSPLEK,[T]	1xTMT6plex [K14];1xTM	P46821	P46821 [1256-1269]	P46821 1xTMT6plex [K	0,01	-0,24	0,12
[R],STSPAGQHHSPISSR,[H]	1xTMT6plex [N-Term];2	Q5T200	Q5T200 [316-330]	Q5T200 1xTMT6plex [N	0,01	-0,24	0,12
[K],FLQEHGSDSFLAEHK,[L]	1xTMT6plex [K15];1xTM	Q00688	Q00688 [28-42]	Q00688 1xTMT6plex [K	0,03	-0,24	0,14
[K],LTHSLSTSDITAIPEK,[E]	1xTMT6plex [K16];1xTM	O15151	O15151 [337-352]	O15151 1xTMT6plex [K	0,03	-0,24	0,13
[R],AMLDSGIYPPGSPGK,[-]	1xOxidation [M2];1xTM	O95295	O95295 [122-136]	O95295 1xOxidation [M	0,01	-0,24	0,12
[R],RNSSEASSGDFLDLK,[G]	1xTMT6plex [K15];1xTM	Q9UK76	Q9UK76 [85-99]	Q9UK76 1xTMT6plex [K	0,00	-0,24	0,12
[K],DVNAAIATIK,[T]	1xTMT6plex [K10];1xTM	Q71U36	Q71U36 [327-336]	Q71U36 1xTMT6plex [K	0,01	-0,24	0,12
[R],TLSLTQR,[G]	1xTMT6plex [N-Term];1	Q8IVF5	Q8IVF5 [751-757]	Q8IVF5 1xTMT6plex [N	0,02	-0,24	0,13
[R],ATTPPNQGRPDSPVYANLQ	1xTMT6plex [K22];1xTM	Q8IWW6	Q8IWW6 [229-250]	Q8IWW6 1xTMT6plex [	0,01	-0,24	0,12
[K],GLGHPPSSPLLK,[K]	1xTMT6plex [K12];1xTM	O95785	O95785 [976-987]	O95785 1xTMT6plex [K	0,00	-0,24	0,12

[R],VTQGAASPGHGIQEK,[L]	1xTMT6plex [K15];1xTM	Q9HB58	Q9HB58 [374-388]	Q9HB58 1xTMT6plex [K	0,00	-0,24	0,11
[R],REHASIDAQSGAGVPNPST	1xTMT6plex [K24];1xTM	Q8IXJ6	Q8IXJ6 [347-370]	Q8IXJ6 1xTMT6plex [K	0,02	-0,24	0,12
[R],STSTPNVHMVSTTLPVDSR	1xTMT6plex [N-Term];1	P04049	P04049 [257-275]	P04049 1xTMT6plex [N	0,01	-0,24	0,12
[R],EQYGLGPYEAVTPLTK,[A]	1xTMT6plex [K16];1xTM	Q12888	Q12888 [1598-1613]	Q12888 1xTMT6plex [K	0,02	-0,24	0,13
[R],ESGSPYSVLVDTK,[M]	1xTMT6plex [K13];1xTM	Q9Y2D9	Q9Y2D9 [54-66]	Q9Y2D9 1xTMT6plex [K	0,01	-0,24	0,12
[R],LQLTTER,[M]	1xTMT6plex [N-Term];1	Q93034	Q93034 [692-698]	Q93034 1xTMT6plex [N	0,02	-0,24	0,12
[K],LKEDSPRK,[T]	2xTMT6plex [K2; K8];1x	Q9ULD9	Q9ULD9 [1266-1273]	Q9ULD9 2xTMT6plex [K	0,03	-0,24	0,13
[R],RGEGDAPFSEPGTTSTQRI	1xTMT6plex [K27];1xTM	Q9UQ35	Q9UQ35 [303-329]	Q9UQ35 1xTMT6plex [I	0,02	-0,24	0,13
[R],SSLSASHPMVGK,[W]	1xTMT6plex [K12];1xTM	Q8TEW0	Q8TEW0 [220-231]	Q8TEW0 1xTMT6plex [	0,01	-0,24	0,12
[K],SSSGLGSSGSLSQK,[T]	1xTMT6plex [K14];1xTM	Q15648	Q15648 [1245-1258]	Q15648 1xTMT6plex [K	0,05	-0,24	0,16
[R],LPHSSSSENIK,[S]	1xTMT6plex [K11];1xTM	Q76I76	Q76I76 [1212-1222]	Q76I76 1xTMT6plex [K	0,04	-0,24	0,15
[K],SPTSPTSPHMPALR,[I]	1xTMT6plex [N-Term];1	P35711	P35711 [408-421]	P35711 1xTMT6plex [N	0,04	-0,24	0,15
[K],KDSSQLGTDATK,[E]	2xTMT6plex [K1; K12];1	O43491	O43491 [13-24]	O43491 2xTMT6plex [K	0,02	-0,24	0,12
[K],LGHPEALSAGTGSPQPPSF	1xTMT6plex [N-Term];1	Q15942	Q15942 [296-320]	Q15942 1xTMT6plex [N	0,03	-0,24	0,14
[R],AYQDQISPVPGAPK,[A]	1xTMT6plex [K14];1xTM	Q969V6	Q969V6 [379-392]	Q969V6 1xTMT6plex [K	0,04	-0,24	0,15
[K],KLPSDSGDLEALEGK,[D]	2xTMT6plex [K1; K15];1	O60524	O60524 [828-842]	O60524 2xTMT6plex [K	0,02	-0,24	0,12
[K],VYTFTDNAPSPSIGSASR,[L	1xTMT6plex [N-Term];1	Q9ULD9	Q9ULD9 [886-903]	Q9ULD9 1xTMT6plex [N	0,02	-0,24	0,13
[K],RPSLPSSPSPGLPK,[A]	1xTMT6plex [K14];1xTM	O43294	O43294 [135-148]	O43294 1xTMT6plex [K	0,04	-0,24	0,16
[R],STPLPGPPGSPR,[S]	1xTMT6plex [N-Term];1	Q8TDC3	Q8TDC3 [499-510]	Q8TDC3 1xTMT6plex [I	0,01	-0,24	0,12
[R],RSLLQSEQQPSWTDDLPL(	1xCarbamidomethyl [C1	Q15750	Q15750 [6-35]	Q15750 1xCarbamidom	0,02	-0,24	0,13
[K],QEPAPPSLSPAVSVK,[S]	1xTMT6plex [K15];1xTM	Q9HCS4	Q9HCS4 [317-331]	Q9HCS4 1xTMT6plex [I	0,01	-0,24	0,12
[K],GGLNTPLHESDFSGVTPQF	1xTMT6plex [N-Term];2	Q99459	Q99459 [381-399]	Q99459 1xTMT6plex [N	0,01	-0,24	0,12
[K],SPSPVQGK,[K]	1xTMT6plex [K8];1xTM	Q96JP5	Q96JP5 [101-108]	Q96JP5 1xTMT6plex [K	0,01	-0,24	0,12
[K],ATSSHPNSTSLK,[A]	1xTMT6plex [K12];1xTM	Q14258	Q14258 [426-437]	Q14258 1xTMT6plex [K	0,04	-0,24	0,15
[R],LQQQHSEQPPLQPSPVMT	1xTMT6plex [N-Term];1	Q5JTV8	Q5JTV8 [130-148]	Q5JTV8 1xTMT6plex [N	0,01	-0,24	0,12
[R],GPSLNPVLDYDHGSRSQES	1xTMT6plex [N-Term];1	P43243	P43243 [193-216]	P43243 1xTMT6plex [N	0,02	-0,24	0,13
[R],SRPYGSSHSPR,[H]	1xTMT6plex [N-Term];1	Q8TAP9	Q8TAP9 [58-68]	Q8TAP9 1xTMT6plex [N	0,03	-0,24	0,14
[R],SGGSIDNVLSQIAAQR,[K]	1xTMT6plex [N-Term];1	Q68DC2	Q68DC2 [657-672]	Q68DC2 1xTMT6plex [N	0,02	-0,24	0,13
[R],VLLSPSIPSVGQDQTLSPGS	1xTMT6plex [K21];1xTM	Q13625	Q13625 [553-573]	Q13625 1xTMT6plex [K	0,04	-0,24	0,16
[R],GLPSTASGR,[L]	1xTMT6plex [N-Term];1	P48730	P48730 [325-333]	P48730 1xTMT6plex [N	0,03	-0,24	0,13
[R],LALSPEDKPIR,[L]	1xTMT6plex [K8];1xTM	O15417	O15417 [1124-1134]	O15417 1xTMT6plex [K	0,03	-0,24	0,13
[R],GKEPKVSAGEPGSHPSPAI	2xTMT6plex [K2; K5];1x	Q9Y4F1	Q9Y4F1 [412-431]	Q9Y4F1 2xTMT6plex [K	0,02	-0,24	0,13
[R],GALQNIIPASTGAAK,[A]	1xTMT6plex [K15];1xTM	P04406	P04406 [201-215]	P04406 1xTMT6plex [K	0,03	-0,24	0,14
[K],LGDAFRPASR,[S]	1xTMT6plex [N-Term];1	Q2M3G4	Q2M3G4 [294-303]	Q2M3G4 1xTMT6plex [	0,03	-0,23	0,14
[K],LGSTTVGSK,[S]	1xTMT6plex [K9];1xTM	Q9P0L2	Q9P0L2 [461-469]	Q9P0L2 1xTMT6plex [K	0,01	-0,23	0,12
[R],LKEEHGIELSSPR,[H]	1xTMT6plex [K2];1xTM	O14647	O14647 [1355-1367]	O14647 1xTMT6plex [K	0,02	-0,23	0,12
[R],SSSSLLASPGHISVK,[E]	1xTMT6plex [K15];1xTM	A0FGR8	A0FGR8 [736-750]	A0FGR8 1xTMT6plex [K	0,02	-0,23	0,13
[R],RPHFPQFSYSASGTA,[-]	1xTMT6plex [N-Term];1	P31749	P31749 [466-480]	P31749 1xTMT6plex [N	0,02	-0,23	0,13
[K],YELISETGGSHDK,[R]	1xTMT6plex [K13];1xTM	Q12906	Q12906 [541-553]	Q12906 1xTMT6plex [K	0,03	-0,23	0,14
[K],LRPYFEGLSHSSSQTEIGSI	1xTMT6plex [N-Term];1	Q86VP3	Q86VP3 [319-342]	Q86VP3 1xTMT6plex [N	0,03	-0,23	0,14
[R],APASVLPAATPR,[Q]	1xTMT6plex [N-Term];1	P13861	P13861 [45-56]	P13861 1xTMT6plex [N	0,01	-0,23	0,12
[K],HTGPNSPDTANDGFVR,[L]	1xTMT6plex [N-Term];1	P31943	P31943 [99-114]	P31943 1xTMT6plex [N	0,02	-0,23	0,13
[K],NNCPFSADENYRPLAK,[T]	1xCarbamidomethyl [C3	Q15154	Q15154 [955-970]	Q15154 1xCarbamidom	0,00	-0,23	0,12
[R],RPEGPGAQAPSSPR,[V]	1xTMT6plex [N-Term];1	P40222	P40222 [504-517]	P40222 1xTMT6plex [N	0,02	-0,23	0,13
[R],SSQHGGSSTSLASTK,[V]	1xTMT6plex [K15];1xTM	O15075	O15075 [346-360]	O15075 1xTMT6plex [K	0,00	-0,23	0,11
[R],FLTTPGCNPQLTYTATLPEI	1xCarbamidomethyl [C7	Q70EL1	Q70EL1 [1556-1575]	Q70EL1 1xCarbamidom	0,01	-0,23	0,12
[K],EKPKEVAENQQNQSSDPEI	3xTMT6plex [K2; K4; K	O43491	O43491 [25-45]	O43491 3xTMT6plex [K	0,04	-0,23	0,15
[K],GSLENVLDSK,[D]	1xTMT6plex [K10];1xTM	P40818	P40818 [152-161]	P40818 1xTMT6plex [K	0,04	-0,23	0,15
[K],TEPACVSPIQNSAPVSDLTK	1xCarbamidomethyl [C5	Q6ZN30	Q6ZN30 [397-416]	Q6ZN30 1xCarbamidom	0,01	-0,23	0,12
[K],LPTPTSSVPAQK,[T]	1xTMT6plex [K12];1xTM	Q9BTA9	Q9BTA9 [291-302]	Q9BTA9 1xTMT6plex [K	0,00	-0,23	0,11
[R],ASSPSPPGR,[F]	1xTMT6plex [N-Term];1	Q86X51	Q86X51 [408-416]	Q86X51 1xTMT6plex [N	0,01	-0,23	0,12
[R],ERGSDASGQLFHGR,[A]	1xTMT6plex [N-Term];1	P19174	P19174 [1230-1243]	P19174 1xTMT6plex [N	0,00	-0,23	0,12
[R],TTTSPFFSEDTFR,[Q]	1xTMT6plex [N-Term];1	Q17R98	Q17R98 [757-769]	Q17R98 1xTMT6plex [N	0,00	-0,23	0,11
[R],LGSVMRPTEDITAR,[E]	1xOxidation [M5];1xTM	Q9P2H5	Q9P2H5 [611-624]	Q9P2H5 1xOxidation [M	0,02	-0,23	0,12
[K],GGGPGKSPTQGST,[-]	1xTMT6plex [K6];1xTM	Q6P582	Q6P582 [146-158]	Q6P582 1xTMT6plex [K	0,02	-0,23	0,12
[R],KASGPPVSELITK,[A]	2xTMT6plex [K1; K13];1	P16402; F	P16402 [35-47]; P10412 [34-46]; P16	P16402 2xTMT6plex [K	0,03	-0,23	0,13
[K],SPSLSPSPPSPLEK,[T]	1xTMT6plex [K14];1xTM	P46821	P46821 [1256-1269]	P46821 1xTMT6plex [K	0,01	-0,23	0,12

[K],RELPSLSPAPDTGLSPSK,[I	1xTMT6plex [K18];1xTM	Q9UBF8	Q9UBF8 [252-269]	Q9UBF8 1xTMT6plex [K	0,04	-0,23	0,16
[K],VHAYFAPVTPPPSVGGSR,[	1xTMT6plex [N-Term];1	Q96IG2	Q96IG2 [409-426]	Q96IG2 1xTMT6plex [N	0,00	-0,23	0,11
[R],LEAHSEMGSTEILEK,[E]	1xTMT6plex [K15];1xTM	Q9NYI0	Q9NYI0 [554-568]	Q9NYI0 1xTMT6plex [K	0,02	-0,23	0,13
[R],SFSSAHR,[H]	1xTMT6plex [N-Term];1	Q96RG2	Q96RG2 [40-46]	Q96RG2 1xTMT6plex [I	0,01	-0,23	0,12
[R],ADKASDTSSETVFGK,[R]	2xTMT6plex [K3; K15];1	Q86V48	Q86V48 [527-541]	Q86V48 2xTMT6plex [K	0,00	-0,23	0,12
[R],SAPASPNHAGVLSAHSSGA	1xTMT6plex [N-Term];2	Q01167	Q01167 [369-395]	Q01167 1xTMT6plex [N	0,03	-0,23	0,14
[R],VSSSPGVK,[G]	1xTMT6plex [K8];1xTM	P32519	P32519 [331-338]	P32519 1xTMT6plex [K	0,03	-0,23	0,13
[R],MSPSQFAR,[V]	1xTMT6plex [N-Term];1	Q86TB9	Q86TB9 [277-284]	Q86TB9 1xTMT6plex [N	0,02	-0,23	0,13
[R],SKFDSDEEEEDTENVEAAS	2xTMT6plex [K2; K22];1	Q8TF01	Q8TF01 [286-307]	Q8TF01 2xTMT6plex [K	0,05	-0,23	0,16
[K],SSKASLGSLEGEAEAEASS	2xTMT6plex [K3; K21];1	Q09666	Q09666 [5745-5765]	Q09666 2xTMT6plex [K	0,03	-0,23	0,14
[R],SAEPAEALVLACK,[R]	1xCarbamidomethyl [C1	Q96CW6	Q96CW6 [16-28]	Q96CW6 1xCarbamido	0,02	-0,23	0,12
[R],VPAASPSAHSISTATPDR,[T	1xTMT6plex [N-Term];1	Q9P0L2	Q9P0L2 [584-601]	Q9P0L2 1xTMT6plex [N	0,03	-0,23	0,14
[K],ATSSSNPSSPAPDWYK,[D]	1xTMT6plex [K16];1xTM	P46100	P46100 [1988-2003]	P46100 1xTMT6plex [K	0,01	-0,23	0,12
[K],KDEESGGGSNPFQHLEK,[S	2xTMT6plex [K1; K17];1	Q9Y678	Q9Y678 [8-24]	Q9Y678 2xTMT6plex [K	0,01	-0,23	0,12
[K],SPPPPTHSTQLGAPSR,[K]	1xTMT6plex [N-Term];1	Q14814	Q14814 [251-266]	Q14814 1xTMT6plex [N	0,00	-0,23	0,12
[R],SLMSSPEDLTK,[D]	1xTMT6plex [K11];1xTM	P46821	P46821 [828-838]	P46821 1xTMT6plex [K	0,02	-0,23	0,12
[R],LVDVSPDRGSPPSR,[V]	1xTMT6plex [N-Term];1	Q9NYZ3	Q9NYZ3 [571-584]	Q9NYZ3 1xTMT6plex [N	0,02	-0,23	0,13
[K],VVEAVNSDSDSEFGIPK,[K]	1xTMT6plex [K17];1xTM	Q02880	Q02880 [1516-1532]	Q02880 1xTMT6plex [K	0,03	-0,23	0,14
[R],INVYYNEATGNK,[Y]	1xTMT6plex [K12];1xTM	Q9BVA1	Q9BVA1 [47-58]	Q9BVA1 1xTMT6plex [K	0,04	-0,23	0,16
[R],QGLAETASPVAVSLR,[S]	1xTMT6plex [N-Term];1	Q15154	Q15154 [854-868]	Q15154 1xTMT6plex [N	0,03	-0,23	0,14
[R],TFSLTEVR,[G]	1xTMT6plex [N-Term];1	O95239	O95239 [799-806]	O95239 1xTMT6plex [N	0,04	-0,23	0,15
[K],LTVSDGESGEEK,[K]	1xTMT6plex [K12];1xTM	P46100	P46100 [1345-1356]	P46100 1xTMT6plex [K	0,02	-0,23	0,13
[K],FLQEHGSDSFLAEHK,[L]	1xTMT6plex [K15];1xTM	Q00688	Q00688 [28-42]	Q00688 1xTMT6plex [K	0,02	-0,23	0,12
[K],THTTALAGRSPSPASGR,[R	1xTMT6plex [N-Term];2	Q9UQ35	Q9UQ35 [286-302]	Q9UQ35 1xTMT6plex [I	0,00	-0,23	0,12
[R],KPVTVSPTTPTSPTEGEAS	1xTMT6plex [K1];1xTM	Q9Y6G9	Q9Y6G9 [505-523]	Q9Y6G9 1xTMT6plex [K	0,00	-0,23	0,11
[K],QGPVSPGPAPPPSFIMSYK	1xOxidation [M16];1xTM	P29375	P29375 [1662-1680]	P29375 1xOxidation [M	0,03	-0,23	0,14
[K],KTSATVGPK,[A]	2xTMT6plex [K1; K9];1x	P16104	P16104 [120-128]	P16104 2xTMT6plex [K	0,01	-0,23	0,12
[R],QLHNSLDPSELPGK,[Q]	1xTMT6plex [K14];1xTM	Q86V48	Q86V48 [1038-1051]	Q86V48 1xTMT6plex [K	0,01	-0,23	0,12
[R],SSPLSWR,[Y]	1xTMT6plex [N-Term];1	Q8NEY1	Q8NEY1 [311-317]	Q8NEY1 1xTMT6plex [I	0,03	-0,22	0,13
[R],NFSFMNPGMER,[L]	1xOxidation [M9];1xTM	Q04759	Q04759 [693-703]	Q04759 1xOxidation [M	0,02	-0,22	0,13
[K],TKESLDVSR,[L]	1xTMT6plex [K2];1xTM	Q32P28	Q32P28 [436-444]	Q32P28 1xTMT6plex [K	0,02	-0,22	0,13
[K],SPGSTPTTPTSSQAPQK,[L	1xTMT6plex [K17];1xTM	P35658	P35658 [430-446]	P35658 1xTMT6plex [K	0,03	-0,22	0,14
[R],RPTLQAVR,[E]	1xTMT6plex [N-Term];1	Q8IWW6	Q8IWW6 [637-644]	Q8IWW6 1xTMT6plex [	0,02	-0,22	0,13
[R],FYSDSHHLK,[R]	1xTMT6plex [K9];1xTM	Q92576	Q92576 [1923-1931]	Q92576 1xTMT6plex [K	0,00	-0,22	0,11
[K],GAASPVLQEDHCDSLPSVL	1xCarbamidomethyl [C1	Q8N9B5	Q8N9B5 [710-733]	Q8N9B5 1xCarbamidon	0,01	-0,22	0,12
[K],YPESNRTPVKPSSVEEEDS	1xTMT6plex [K10];1xTM	Q9UHB7	Q9UHB7 [668-689]	Q9UHB7 1xTMT6plex [I	0,01	-0,22	0,12
[R],FKSDSGSLGDAK,[N]	2xTMT6plex [K2; K12];1	Q5T5U3	Q5T5U3 [1711-1722]	Q5T5U3 2xTMT6plex [K	0,01	-0,22	0,12
[K],VKPPPQISPSK,[S]	2xTMT6plex [K2; K11];1	Q9H6U6	Q9H6U6 [563-573]	Q9H6U6 2xTMT6plex [K	0,01	-0,22	0,12
[K],TKDSGLPSQGLNFK,[F]	2xTMT6plex [K2; K14];1	Q12959	Q12959 [595-608]	Q12959 2xTMT6plex [K	0,04	-0,22	0,15
[K],VNFSEEGETEEDDQDSSHS	1xTMT6plex [K25];1xTM	Q5JTV8	Q5JTV8 [212-236]	Q5JTV8 1xTMT6plex [K	0,03	-0,22	0,14
[K],THTTALAGRSPSPASGR,[R	1xTMT6plex [N-Term];2	Q9UQ35	Q9UQ35 [286-302]	Q9UQ35 1xTMT6plex [I	0,00	-0,22	0,11
[K],SHHANSPTAGAAK,[S]	1xTMT6plex [K13];1xTM	Q15555	Q15555 [195-207]	Q15555 1xTMT6plex [K	0,02	-0,22	0,13
[R],SSAPFSPPSGPPEK,[-]	1xTMT6plex [K14];1xTM	Q5TZA2	Q5TZA2 [2004-2017]	Q5TZA2 1xTMT6plex [K	0,05	-0,22	0,17
[K],DSDFSLPPGSASGPTGSPV	1xTMT6plex [K21];1xTM	Q5JSZ5	Q5JSZ5 [1792-1812]	Q5JSZ5 1xTMT6plex [K	0,04	-0,22	0,16
[R],SDKGSPGEDGFVPSALGTF	1xTMT6plex [K3];1xTM	Q5JPI9	Q5JPI9 [17-35]	Q5JPI9 1xTMT6plex [K:	0,04	-0,22	0,16
[R],RGSAPWHSFSR,[F]	1xTMT6plex [N-Term];1	Q68DQ2	Q68DQ2 [7-17]	Q68DQ2 1xTMT6plex [I	0,04	-0,22	0,15
[K],RPYLGPALLLTPR,[D]	1xTMT6plex [N-Term];1	Q7Z5J4	Q7Z5J4 [1466-1478]	Q7Z5J4 1xTMT6plex [N	0,03	-0,22	0,13
[R],LGGLRPESPESLTSVSR,[T]	1xTMT6plex [N-Term];1	Q9H6F5	Q9H6F5 [11-27]	Q9H6F5 1xTMT6plex [N	0,02	-0,22	0,13
[R],KASPEPEGEAAGK,[M]	2xTMT6plex [K1; K13];1	Q8IU81	Q8IU81 [382-394]	Q8IU81 2xTMT6plex [K	0,01	-0,22	0,12
[R],TSPGAPPPASKPK,[L]	2xTMT6plex [K11; K13]	Q9BZI1	Q9BZI1 [316-328]	Q9BZI1 2xTMT6plex [K	0,02	-0,22	0,12
[R],LSDSPSMK,[K]	1xTMT6plex [K8];1xTM	Q9BY77	Q9BY77 [365-372]	Q9BY77 1xTMT6plex [K	0,04	-0,22	0,16
[K],FGSTGSTPPVSPTPSER,[S	1xTMT6plex [N-Term];1	Q969V6	Q969V6 [444-460]	Q969V6 1xTMT6plex [N	0,00	-0,22	0,11
[R],SRSPHEAGFCVYLK,[G]	1xCarbamidomethyl [C1	Q9NTZ6	Q9NTZ6 [422-435]	Q9NTZ6 1xCarbamidon	0,01	-0,22	0,12
[R],TWSAPYDGERPSPEPSPF	1xTMT6plex [N-Term];1	Q8N612	Q8N612 [571-591]	Q8N612 1xTMT6plex [N	0,01	-0,22	0,12
[K],LNHVAAGLVSPSLKSDTSSI	2xTMT6plex [K14; K20]	Q05519	Q05519 [198-217]	Q05519 2xTMT6plex [K	0,02	-0,22	0,13
[R],GPSVPSAGK,[V]	1xTMT6plex [K9];1xTM	Q8IUG5	Q8IUG5 [615-623]	Q8IUG5 1xTMT6plex [K	0,01	-0,22	0,12
[R],TPEQYYQTFSPSSSHSPAF	1xTMT6plex [N-Term];1	Q7Z5J4	Q7Z5J4 [330-348]	Q7Z5J4 1xTMT6plex [N	0,01	-0,22	0,12

[K],KAESTESIGSSEK,[T]	2xTMT6plex [K1; K13];1	Q9BZI7	Q9BZI7 [406-418]	Q9BZI7 2xTMT6plex [K	0,04	-0,22	0,15
[R],VSGSFPEDSSK,[E]	1xTMT6plex [K11];1xTM	O15400	O15400 [128-138]	O15400 1xTMT6plex [K	0,01	-0,22	0,12
[K],TLVLSSSPTSPTQEPLPGGI	1xTMT6plex [K20];1xTM	Q96QF0	Q96QF0 [257-276]	Q96QF0 1xTMT6plex [K	0,02	-0,22	0,13
[K],FTPPAESGSPSK,[E]	1xTMT6plex [K12];1xTM	Q70E73	Q70E73 [1004-1015]	Q70E73 1xTMT6plex [K	0,01	-0,22	0,12
[R],GGTSQVGSPTLLSPAVPSK	1xTMT6plex [K19];1xTM	Q8IY92	Q8IY92 [1063-1081]	Q8IY92 1xTMT6plex [K	0,02	-0,22	0,13
[R],DNTFFRESPVGR,[K]	1xTMT6plex [N-Term];1	Q8NEY8	Q8NEY8 [126-137]	Q8NEY8 1xTMT6plex [I	0,03	-0,22	0,14
[K],TPTPPAPTLLK,[M]	1xTMT6plex [K11];1xTM	Q14686	Q14686 [1878-1888]	Q14686 1xTMT6plex [K	0,03	-0,22	0,14
[K],AGGSPAPGPETPAISPSK,[I	1xTMT6plex [K18];1xTM	P33316	P33316 [85-102]	P33316 1xTMT6plex [K	0,03	-0,22	0,13
[R],RATPHPSELK,[V]	1xTMT6plex [K10];1xTM	Q14160	Q14160 [473-482]	Q14160 1xTMT6plex [K	0,02	-0,22	0,12
[R],RDSFDDRGPSLNPVLDYDH	1xTMT6plex [N-Term];1	P43243	P43243 [186-207]	P43243 1xTMT6plex [N	0,03	-0,22	0,14
[R],SQSSDTEQQSPTSGGGK,[	1xTMT6plex [K17];1xTM	P23588	P23588 [495-511]	P23588 1xTMT6plex [K	0,02	-0,22	0,13
[K],LGSSSTSIPHLSSPPTK,[F]	1xTMT6plex [K17];1xTM	Q96QT4	Q96QT4 [1384-1400]	Q96QT4 1xTMT6plex [K	0,01	-0,22	0,12
[R],SVSVVTGSEQK,[R]	1xTMT6plex [K11];1xTM	Q9UPN4	Q9UPN4 [45-55]	Q9UPN4 1xTMT6plex [I	0,03	-0,22	0,13
[K],RPATADSPKPSAK,[K]	2xTMT6plex [K9; K13];1	Q03111	Q03111 [286-298]	Q03111 2xTMT6plex [K	0,02	-0,22	0,13
[R],AFHGISPGLLASEK,[T]	1xTMT6plex [K14];1xTM	Q9P1Z0	Q9P1Z0 [386-399]	Q9P1Z0 1xTMT6plex [K	0,02	-0,22	0,13
[K],IHVQSSSDSSDEPAEK,[R]	1xTMT6plex [K16];1xTM	Q8WUF8	Q8WUF8 [211-226]	Q8WUF8 1xTMT6plex [	0,03	-0,22	0,14
[R],TYSQDCSFK,[N]	1xCarbamidomethyl [C6	Q86U86	Q86U86 [946-954]	Q86U86 1xCarbamidom	0,04	-0,22	0,16
[R],RNSSEASSGDFLDLK,[G]	1xTMT6plex [K15];1xTM	Q9UK76	Q9UK76 [85-99]	Q9UK76 1xTMT6plex [K	0,00	-0,22	0,12
[R],RPSVYLPTR,[E]	1xTMT6plex [N-Term];1	Q9BW61	Q9BW61 [31-39]	Q9BW61 1xTMT6plex [	0,01	-0,22	0,12
[R],AGSSGNSCITYQPSVSGEH	1xCarbamidomethyl [C8	Q99755	Q99755 [473-492]	Q99755 1xCarbamidom	0,04	-0,22	0,15
[R],SRESLNVDVVK,[Y]	1xTMT6plex [K11];1xTM	Q07866	Q07866 [521-531]	Q07866 1xTMT6plex [K	0,03	-0,22	0,13
[K],IQPLEPDSPTGLSENPTPAT	1xTMT6plex [K22];1xTM	Q9ULJ3	Q9ULJ3 [996-1017]	Q9ULJ3 1xTMT6plex [K	0,00	-0,22	0,11
[K],LGSLTPEIVSTPSSPEEEDK	1xTMT6plex [K20];1xTM	Q14CS0	Q14CS0 [222-241]	Q14CS0 1xTMT6plex [K	0,04	-0,22	0,15
[K],LISHCYIPQSPEPDLHK,[E]	1xCarbamidomethyl [C5	A2A3K4	A2A3K4 [429-445]	A2A3K4 1xCarbamidom	0,01	-0,22	0,12
[R],GNIETTSEDGQVFSPK,[K]	1xTMT6plex [K16];1xTM	Q5T200	Q5T200 [980-995]	Q5T200 1xTMT6plex [K	0,01	-0,22	0,12
[R],FSEGVLQSPSQDQEK,[L]	1xTMT6plex [K15];1xTM	Q9C0C2	Q9C0C2 [428-442]	Q9C0C2 1xTMT6plex [K	0,02	-0,22	0,13
[K],THSPLKPYGCEECGK,[S]	2xCarbamidomethyl [C1	Q13105	Q13105 [355-369]	Q13105 2xCarbamidom	0,01	-0,22	0,12
[R],RNSRDGDPLPSSLSCK,[V]	1xCarbamidomethyl [C1	P53602	P53602 [94-109]	P53602 1xCarbamidom	0,00	-0,22	0,12
[K],VKEPSVQEATSTSDILK,[V]	2xTMT6plex [K2; K17];1	Q8IYB3	Q8IYB3 [230-246]	Q8IYB3 2xTMT6plex [K	0,02	-0,22	0,13
[K],TNDASSESIASFSK,[Q]	1xTMT6plex [K14];1xTM	Q7RTN6	Q7RTN6 [42-55]	Q7RTN6 1xTMT6plex [I	0,01	-0,21	0,12
[R],NFGYPLGEMSQPTPSPAPA	1xOxidation [M9];1xTMT	Q15750	Q15750 [364-386]	Q15750 1xOxidation [M	0,01	-0,21	0,12
[K],FLWNPSSLPSP,[-]	1xTMT6plex [N-Term];1	Q8NHY6	Q8NHY6 [858-868]	Q8NHY6 1xTMT6plex [I	0,05	-0,21	0,16
[R],TGSSPTQGIVNK,[A]	1xTMT6plex [K12];1xTM	Q9UPT6	Q9UPT6 [362-373]	Q9UPT6 1xTMT6plex [K	0,01	-0,21	0,12
[R],EGGPGSSLTLPK,[V]	1xTMT6plex [K12];1xTM	Q9ULM0	Q9ULM0 [303-314]	Q9ULM0 1xTMT6plex [I	0,01	-0,21	0,12
[K],FLDTSHYSTAGSSSVR,[E]	1xTMT6plex [N-Term];1	Q96A65	Q96A65 [241-256]	Q96A65 1xTMT6plex [N	0,00	-0,21	0,11
[K],SLYESFVSSSDR,[L]	1xTMT6plex [N-Term];1	P18615	P18615 [131-142]	P18615 1xTMT6plex [N	0,03	-0,21	0,14
[K],YSPTSPTYSPTSPVYTPTSI	1xTMT6plex [K21];1xTM	P24928	P24928 [1888-1908]	P24928 1xTMT6plex [K	0,05	-0,21	0,16
[R],SSLSGDEEDELFKGATLK,[A	2xTMT6plex [K13; K18]	Q5T1M5	Q5T1M5 [1161-1178]	Q5T1M5 2xTMT6plex [K	0,05	-0,21	0,16
[R],EAALPPVSPLK,[A]	1xTMT6plex [K11];1xTM	Q04637	Q04637 [1224-1234]	Q04637 1xTMT6plex [K	0,03	-0,21	0,14
[R],YFCHCCSVEIVPR,[L]	3xCarbamidomethyl [C3	Q9BV68	Q9BV68 [11-23]	Q9BV68 3xCarbamidom	0,02	-0,21	0,13
[R],TVSVLDPYYRPR,[D]	1xTMT6plex [N-Term];1	Q96S53	Q96S53 [379-390]	Q96S53 1xTMT6plex [N	0,03	-0,21	0,13
[R],LLDHMAPPPVADQASPR,[A	1xTMT6plex [N-Term];1	Q9C004	Q9C004 [111-127]	Q9C004 1xTMT6plex [N	0,04	-0,21	0,16
[K],QSPGHQSPLASPK,[V]	1xTMT6plex [K13];1xTM	Q8N1G0	Q8N1G0 [265-277]	Q8N1G0 1xTMT6plex [I	0,03	-0,21	0,13
[R],IPRPSVSQGCSR,[E]	1xCarbamidomethyl [C1	O75122	O75122 [519-530]	O75122 1xCarbamidom	0,00	-0,21	0,12
[R],NVIPDTPPSTPLVPSR,[A]	1xTMT6plex [N-Term];1	Q9UKA4	Q9UKA4 [1095-1110]	Q9UKA4 1xTMT6plex [I	0,04	-0,21	0,15
[K],IISTTASK,[T]	1xTMT6plex [K8];1xTM	O95292	O95292 [140-147]	O95292 1xTMT6plex [K	0,01	-0,21	0,12
[R],AGGASPAASSTAQPPTQHI	1xTMT6plex [N-Term];1	Q8IU81	Q8IU81 [449-467]	Q8IU81 1xTMT6plex [N	0,01	-0,21	0,12
[R],SQSVSPTSFLTISNEGSEES	1xCarbamidomethyl [C2	Q6P1L5	Q6P1L5 [410-441]	Q6P1L5 1xCarbamidom	0,03	-0,21	0,13
[R],GIQLSPGVGSSAGPPGDLE	1xTMT6plex [K25];1xTM	O75420	O75420 [402-426]	O75420 1xTMT6plex [K	0,01	-0,21	0,12
[K],QSPGHQSPLASPK,[V]	1xTMT6plex [K13];1xTM	Q8N1G0	Q8N1G0 [265-277]	Q8N1G0 1xTMT6plex [I	0,00	-0,21	0,12
[R],NNRPAFFSPSLK,[R]	1xTMT6plex [K12];1xTM	Q6ICG6	Q6ICG6 [297-308]	Q6ICG6 1xTMT6plex [K	0,00	-0,21	0,12
[R],AGSPFSPPPSSSSLTGEAA	1xTMT6plex [N-Term];1	Q96JY6	Q96JY6 [132-153]	Q96JY6 1xTMT6plex [N	0,03	-0,21	0,14
[R],TLHSPPLQLQQR,[S]	1xTMT6plex [N-Term];1	Q6PFW1	Q6PFW1 [1149-1160]	Q6PFW1 1xTMT6plex [	0,01	-0,21	0,12
[K],AAFHHLSAPALLSAQTR,[G]	1xTMT6plex [N-Term];1	Q8IVE3	Q8IVE3 [1452-1468]	Q8IVE3 1xTMT6plex [N	0,00	-0,21	0,12
[K],EEALQMSSPGRK,[E]	1xOxidation [M6];1xTMT	Q76L83	Q76L83 [433-444]	Q76L83 1xOxidation [M	0,03	-0,21	0,13
[K],SREDLSAQPVQTK,[F]	1xTMT6plex [K13];1xTM	Q07157	Q07157 [617-629]	Q07157 1xTMT6plex [K	0,03	-0,21	0,14
[R],LNQQPSQGLGPR,[G]	1xTMT6plex [N-Term];1	P36915	P36915 [63-74]	P36915 1xTMT6plex [N	0,03	-0,21	0,14

[K],FLQEHGSDSFLAEHK,[L]	1xTMT6plex [K15];1xTM	Q00688	Q00688 [28-42]	Q00688 1xTMT6plex [K	0,00	-0,21	0,11
[R],GGPPSPTK,[T]	1xTMT6plex [K8];1xTM	Q5TCQ9	Q5TCQ9 [655-662]	Q5TCQ9 1xTMT6plex [	0,00	-0,21	0,12
[K],SLNANTDITSLAR,[K]	1xTMT6plex [N-Term];1	Q92845	Q92845 [60-72]	Q92845 1xTMT6plex [N	0,03	-0,21	0,14
[R],RLSSTSLASGHSVR,[L]	1xTMT6plex [N-Term];1	Q9BZL6	Q9BZL6 [195-208]	Q9BZL6 1xTMT6plex [N	0,00	-0,21	0,12
[R],IGELGAPEVWGLSPK,[N]	1xTMT6plex [K15];1xTM	Q8N5F7	Q8N5F7 [137-151]	Q8N5F7 1xTMT6plex [K	0,03	-0,21	0,13
[R],SQSFSHQQPSR,[S]	1xTMT6plex [N-Term];1	Q8WVR3	Q8WVR3 [515-525]	Q8WVR3 1xTMT6plex [	0,01	-0,21	0,12
[R],SASEPSLHR,[AT]	1xTMT6plex [N-Term];1	P04049; F	P04049 [619-627]; P10398 [580-588]	P04049 1xTMT6plex [N	0,03	-0,21	0,13
[R],NLQSPTQFQTPR,[S]	1xTMT6plex [N-Term];1	O75385	O75385 [447-458]	O75385 1xTMT6plex [N	0,02	-0,21	0,13
[R],RPSHEGYLAAPGLEGPGPI	1xOxidation [M24];1xTM	O94910	O94910 [1445-1474]	O94910 1xOxidation [M	0,04	-0,21	0,16
[K],SLPLPTPEEK,[M]	1xTMT6plex [K10];1xTM	Q5SYE7	Q5SYE7 [198-207]	Q5SYE7 1xTMT6plex [K	0,04	-0,21	0,15
[K],TSTSPPPEK,[S]	1xTMT6plex [K9];1xTM	Q9H1E3	Q9H1E3 [220-228]	Q9H1E3 1xTMT6plex [K	0,04	-0,21	0,15
[R],EAGRPGVLQAPPGSPRPLI	1xTMT6plex [N-Term];1	O15417	O15417 [403-427]	O15417 1xTMT6plex [N	0,02	-0,21	0,12
[R],INGIHGAVFGKGTYFAR,[D]	1xMethyl [K11];1xTMT6	Q9NR21	Q9NR21 [223-239]	Q9NR21 1xMethyl [K23	0,04	-0,21	0,15
[K],APELPNTSSSPSLK,[M]	1xTMT6plex [K14];1xTM	Q9UN79	Q9UN79 [301-314]	Q9UN79 1xTMT6plex [K	0,03	-0,21	0,13
[K],TPPTPPSSIVAK,[V]	1xTMT6plex [K12];1xTM	Q96QT6	Q96QT6 [877-888]	Q96QT6 1xTMT6plex [K	0,01	-0,21	0,12
[R],SLSGSADENPSCGTGSER,	1xCarbamidomethyl [C1	Q86X51	Q86X51 [361-378]	Q86X51 1xCarbamidom	0,02	-0,21	0,13
[R],ASPSPQPSSQPLQIHR,[Q]	1xTMT6plex [N-Term];1	Q9HC35	Q9HC35 [143-158]	Q9HC35 1xTMT6plex [N	0,00	-0,21	0,12
[K],TPPVVIK,[S]	1xTMT6plex [K7];1xTM	Q96A49	Q96A49 [248-254]	Q96A49 1xTMT6plex [K	0,01	-0,21	0,12
[R],RFSNVGLVHTSER,[R]	1xTMT6plex [N-Term];1	Q9C0H9	Q9C0H9 [43-55]	Q9C0H9 1xTMT6plex [N	0,04	-0,21	0,15
[R],KVSPESSPDQEETEINFTQI	2xTMT6plex [K1; K20];1	Q8IYW5	Q8IYW5 [409-428]	Q8IYW5 2xTMT6plex [K	0,04	-0,21	0,15
[R],VALVVHPGTAR,[L]	1xTMT6plex [N-Term];1	Q12846	Q12846 [23-33]	Q12846 1xTMT6plex [N	0,04	-0,21	0,16
[K],SPTATFEK,[H]	1xTMT6plex [K8];1xTM	Q9HC44	Q9HC44 [21-28]	Q9HC44 1xTMT6plex [K	0,02	-0,21	0,13
[R],AKTPEPGAQQSGFPTLSR,	1xTMT6plex [K2];1xTM	Q9H9D4	Q9H9D4 [320-337]	Q9H9D4 1xTMT6plex [K	0,02	-0,21	0,13
[R],ALQSPALGLR,[G]	1xTMT6plex [N-Term];1	P46940	P46940 [327-336]	P46940 1xTMT6plex [N	0,02	-0,21	0,12
[K],GSRPPLILQSQSLPCSSPR,	1xCarbamidomethyl [C1	Q6KC79	Q6KC79 [290-308]	Q6KC79 1xCarbamidom	0,01	-0,21	0,12
[K],SPSLESLSRPPSLGFGDTR	1xTMT6plex [N-Term];1	Q5M775	Q5M775 [912-930]	Q5M775 1xTMT6plex [N	0,01	-0,21	0,12
[R],LSSPVLHR,[L]	1xTMT6plex [N-Term];1	Q16643	Q16643 [140-147]	Q16643 1xTMT6plex [N	0,04	-0,21	0,15
[R],SGGGSGGSGGQVSLK,[K]	1xTMT6plex [K15];1xTM	Q9H7N4	Q9H7N4 [929-943]	Q9H7N4 1xTMT6plex [K	0,04	-0,21	0,15
[R],SPSKPTLAYPESNSR,[A]	1xTMT6plex [K4];1xTM	Q86XR8	Q86XR8 [53-67]	Q86XR8 1xTMT6plex [K	0,02	-0,21	0,12
[R],SNSAPLIHGLSDTSPVFQAE	1xTMT6plex [N-Term];1	Q96E09	Q96E09 [35-59]	Q96E09 1xTMT6plex [N	0,02	-0,21	0,13
[R],VTSFSTPPTPER,[N]	1xTMT6plex [N-Term];1	Q6ICG6	Q6ICG6 [285-296]	Q6ICG6 1xTMT6plex [N	0,03	-0,21	0,13
[K],LGSDISK,[H]	1xTMT6plex [K7];1xTM	Q9HCE1	Q9HCE1 [95-101]	Q9HCE1 1xTMT6plex [	0,03	-0,21	0,14
[K],TKKDDSHSAEDSEDEK,[E]	3xTMT6plex [K2; K3; K	Q9H1E3	Q9H1E3 [68-83]	Q9H1E3 3xTMT6plex [K	0,00	-0,21	0,11
[R],GPGAPGLAHLQESQAGSD	1xTMT6plex [K25];1xTM	Q14676	Q14676 [360-384]	Q14676 1xTMT6plex [K	0,04	-0,21	0,15
[R],VYENVGLMQQQK,[S]	1xOxidation [M8];1xTM	Q06124	Q06124 [579-590]	Q06124 1xOxidation [M	0,03	-0,20	0,14
[R],NQSLGQSAPSLTAGLK,[E]	1xTMT6plex [K16];1xTM	Q6P0Q8	Q6P0Q8 [142-157]	Q6P0Q8 1xTMT6plex [K	0,05	-0,20	0,16
[R],HPASLTSSGSSGSPSSSIK,	1xTMT6plex [K19];1xTM	Q9C0D5	Q9C0D5 [1656-1674]	Q9C0D5 1xTMT6plex [K	0,02	-0,20	0,13
[R],STSTPNVHMVSTTLPVDSR	1xOxidation [M9];1xTM	P04049	P04049 [257-275]	P04049 1xOxidation [M	0,02	-0,20	0,12
[K],IGTPTRPCPLPPGK,[R]	1xCarbamidomethyl [C8	P42566	P42566 [775-788]	P42566 1xCarbamidom	0,04	-0,20	0,16
[R],LTTGTPASPR,[R]	1xTMT6plex [N-Term];1	Q99501	Q99501 [387-396]	Q99501 1xTMT6plex [N	0,01	-0,20	0,12
[R],KPDSMVTNSSTENEA,[-]	1xOxidation [M5];1xTM	O43237	O43237 [478-492]	O43237 1xOxidation [M	0,01	-0,20	0,12
[R],SRTPLLPR,[K]	1xTMT6plex [N-Term];1	Q9UQ35	Q9UQ35 [2032-2039]	Q9UQ35 1xTMT6plex [N	0,00	-0,20	0,11
[R],SRTPLLPR,[K]	1xTMT6plex [N-Term];1	Q9UQ35	Q9UQ35 [2032-2039]	Q9UQ35 1xTMT6plex [N	0,00	-0,20	0,11
[R],SLAALDALNTDDENDEEEY	1xTMT6plex [K23];1xTM	P55081	P55081 [258-280]	P55081 1xTMT6plex [K	0,01	-0,20	0,12
[R],AAAAAAAATMALAAPSSPT	1xTMT6plex [K28];1xTM	Q9NQS7	Q9NQS7 [127-154]	Q9NQS7 1xTMT6plex [	0,04	-0,20	0,15
[K],TTKTPEDGDYSYEIIEK,[T]	2xTMT6plex [K3; K17];1	P46821	P46821 [1929-1945]	P46821 2xTMT6plex [K	0,03	-0,20	0,14
[K],TTKTPEDGDYSYEIIEK,[T]	2xTMT6plex [K3; K17];1	P46821	P46821 [1929-1945]	P46821 2xTMT6plex [K	0,03	-0,20	0,14
[R],GGSLDPSHSSGR,[G]	1xTMT6plex [N-Term];1	Q96JQ0	Q96JQ0 [3022-3033]	Q96JQ0 1xTMT6plex [N	0,05	-0,20	0,16
[R],RQGLGPIHSR,[T]	1xTMT6plex [N-Term];1	Q99835	Q99835 [763-772]	Q99835 1xTMT6plex [N	0,00	-0,20	0,12
[R],LPHSSSSENIK,[S]	1xTMT6plex [K11];1xTM	Q76I76	Q76I76 [1212-1222]	Q76I76 1xTMT6plex [K1	0,02	-0,20	0,13
[R],NSPFQIPPPSPDSK,[K]	1xTMT6plex [K14];1xTM	Q86W92	Q86W92 [570-583]	Q86W92 1xTMT6plex [	0,03	-0,20	0,13
[K],IAELEEEQSQGSTTNSDWM	1xTMT6plex [K20];1xTM	P46821	P46821 [328-347]	P46821 1xTMT6plex [K	0,05	-0,20	0,16
[K],RASLPAGLVGTPPESPSEP	1xTMT6plex [N-Term];1	A6NDB9	A6NDB9 [141-160]	A6NDB9 1xTMT6plex [N	0,01	-0,20	0,12
[R],IDSPGFKPASQQK,[A]	2xTMT6plex [K7; K13];1	Q07157	Q07157 [910-922]	Q07157 2xTMT6plex [K	0,04	-0,20	0,15
[R],KRESESESDETPPAAPQLIK	2xTMT6plex [K1; K20];1	O60832	O60832 [448-467]	O60832 2xTMT6plex [K	0,00	-0,20	0,11
[R],THFPQFSYSASIRE,[-]	1xTMT6plex [N-Term];1	P31751	P31751 [468-481]	P31751 1xTMT6plex [N	0,00	-0,20	0,12
[K],ASAQASLASK,[D]	1xTMT6plex [K10];1xTM	Q15154	Q15154 [1282-1291]	Q15154 1xTMT6plex [K	0,01	-0,20	0,12

[R],CPSLDNLAVPESPGVGGGI	1xCarbamidomethyl [C1	O14686	O14686 [2249-2267]	O14686 1xCarbamidom	0,01	-0,20	0,12
[K],KGPQASSPR,[R]	1xTMT6plex [K1];1xTM	Q8N1G1	Q8N1G1 [416-424]	Q8N1G1 1xTMT6plex [	0,02	-0,20	0,13
[K],NSSLALSQSSPSSISSPGHS	1xTMT6plex [N-Term];1	Q9ULK2	Q9ULK2 [828-848]	Q9ULK2 1xTMT6plex [N	0,02	-0,20	0,12
[R],AGSGLVLSGSEIPK,[E]	1xTMT6plex [K14];1xTM	Q6W2J9	Q6W2J9 [471-484]	Q6W2J9 1xTMT6plex [	0,04	-0,20	0,15
[R],DALGDSLQVPVSPSSTTSS	1xTMT6plex [N-Term];1	Q9Y2D5	Q9Y2D5 [141-160]	Q9Y2D5 1xTMT6plex [N	0,04	-0,20	0,16
[R],QPSLNAYNSLTR,[S]	1xTMT6plex [N-Term];1	Q9H2E6	Q9H2E6 [986-997]	Q9H2E6 1xTMT6plex [N	0,05	-0,20	0,16
[R],APSPASFQR,[S]	1xTMT6plex [N-Term];1	Q8NFD5	Q8NFD5 [1540-1548]	Q8NFD5 1xTMT6plex [	0,01	-0,20	0,12
[R],GAPSSPATGVLPSPQGK,[S	1xTMT6plex [K17];1xTM	Q5SRE5	Q5SRE5 [1705-1721]	Q5SRE5 1xTMT6plex [	0,01	-0,20	0,12
[R],RASLLAFAEDAPPSR,[A]	1xTMT6plex [N-Term];1	Q6NV75	Q6NV75 [437-451]	Q6NV75 1xTMT6plex [N	0,04	-0,20	0,15
[R],ITVGNDHFCVSTPER,[R]	1xCarbamidomethyl [C9	Q5VUA4	Q5VUA4 [150-164]	Q5VUA4 1xCarbamidor	0,03	-0,20	0,13
[R],TASPLPLRPDAPVEKSPEET	2xPhospho [T1; S3];2xT	Q9HCK8	Q9HCK8 [1993-2025]	Q9HCK8 2xPhospho [T	0,03	-0,20	0,13
[R],TASPLPLRPDAPVEKSPEET	2xTMT6plex [K15; K33]	Q9HCK8	Q9HCK8 [1993-2025]	Q9HCK8 2xTMT6plex [	0,03	-0,20	0,13
[R],TASPLPLRPDAPVEKSPEET	2xPhospho [T1; T20];2x	Q9HCK8	Q9HCK8 [1993-2025]	Q9HCK8 2xPhospho [T	0,03	-0,20	0,13
[R],TASPLPLRPDAPVEKSPEET	2xPhospho [T1; T22];2x	Q9HCK8	Q9HCK8 [1993-2025]	Q9HCK8 2xPhospho [T	0,03	-0,20	0,13
[R],TASPLPLRPDAPVEKSPEET	2xPhospho [T1; S16];2x	Q9HCK8	Q9HCK8 [1993-2025]	Q9HCK8 2xPhospho [T	0,03	-0,20	0,13
[R],LGGSTSDPPSSQSFSFHR,	1xTMT6plex [N-Term];1	Q13884	Q13884 [222-239]	Q13884 1xTMT6plex [N	0,02	-0,20	0,13
[R],SPLSPTETFSWPDVR,[E]	1xTMT6plex [N-Term];1	A1L390	A1L390 [1037-1051]	A1L390 1xTMT6plex [N	0,04	-0,20	0,15
[K],RTSSIADEGTYTLDSILR,[Q]	1xTMT6plex [N-Term];1	Q9Y4I1	Q9Y4I1 [1649-1666]	Q9Y4I1 1xTMT6plex [N	0,02	-0,20	0,13
[K],GESQNTDLSPKPLISEQTVI	2xTMT6plex [K11; K23]	P15622	P15622 [126-148]	P15622 2xTMT6plex [K	0,05	-0,20	0,16
[R],VLTLSERPLDFLDLERPPTT	1xTMT6plex [N-Term];1	Q9GZY8	Q9GZY8 [119-145]	Q9GZY8 1xTMT6plex [	0,01	-0,20	0,12
[K],RRSPSLSSK,[H]	1xTMT6plex [K9];1xTM	Q8IYB3	Q8IYB3 [651-659]	Q8IYB3 1xTMT6plex [K	0,00	-0,20	0,11
[K],DKEPFTFSSPASGR,[S]	1xTMT6plex [K2];1xTM	Q6P1X5	Q6P1X5 [1177-1190]	Q6P1X5 1xTMT6plex [K	0,00	-0,20	0,11
[R],TVAISDAAQLPHDYCTTPG	1xCarbamidomethyl [C1	Q13542	Q13542 [21-51]	Q13542 1xCarbamidom	0,04	-0,20	0,15
[K],KAPSSPPPPPPLR,[S]	1xTMT6plex [K1];1xTM	Q13796	Q13796 [309-322]	Q13796 1xTMT6plex [K	0,04	-0,20	0,16
[K],SFPSSPSK,[G]	1xTMT6plex [K8];1xTM	Q96KQ7	Q96KQ7 [115-122]	Q96KQ7 1xTMT6plex [	0,02	-0,20	0,13
[R],VASVASSLAAR,[G]	1xTMT6plex [N-Term];1	Q96JQ0	Q96JQ0 [3053-3063]	Q96JQ0 1xTMT6plex [N	0,01	-0,20	0,12
[K],LGVSVSPSR,[A]	1xTMT6plex [N-Term];1	Q86VM9	Q86VM9 [529-537]	Q86VM9 1xTMT6plex [	0,02	-0,20	0,13
[K],ERPISLGIFPLPAGDGLLTPI	1xTMT6plex [K24];1xTM	O60271	O60271 [199-222]	O60271 1xTMT6plex [K	0,01	-0,20	0,12
[R],LDNVPHTPSSYIETLPK,[A]	1xTMT6plex [K17];1xTM	Q99733	Q99733 [45-61]	Q99733 1xTMT6plex [K	0,04	-0,20	0,15
[R],VGAGDSQLK,[G]	1xTMT6plex [K9];1xTM	P43246	P43246 [712-720]	P43246 1xTMT6plex [K	0,05	-0,20	0,16
[R],IPASQTSVPFDHLGK,[-]	1xTMT6plex [K15];1xTM	P49674	P49674 [402-416]	P49674 1xTMT6plex [K	0,02	-0,20	0,12
[R],LYSGSPTR,[S]	1xTMT6plex [N-Term];1	Q6PJI9	Q6PJI9 [599-606]	Q6PJI9 1xTMT6plex [N	0,03	-0,20	0,14
[K],APASPGAGNPPGTPK,[G]	1xTMT6plex [K15];1xTM	Q9ULD9	Q9ULD9 [624-638]	Q9ULD9 1xTMT6plex [	0,04	-0,19	0,15
[R],SSSAPNVHINTIEPVNIDDLI	1xTMT6plex [N-Term];1	P15056	P15056 [363-384]	P15056 1xTMT6plex [N	0,03	-0,19	0,14
[R],MDRTPPPPTLSPAAITVGR,	1xTMT6plex [N-Term];1	Q8NDX5	Q8NDX5 [606-624]	Q8NDX5 1xTMT6plex [	0,01	-0,19	0,12
[K],LSSWDQAETPGHTPSLR,[V	1xTMT6plex [N-Term];1	O75533	O75533 [215-231]	O75533 1xTMT6plex [N	0,02	-0,19	0,13
[K],GLQSLPTHDPSPLQR,[Y]	1xTMT6plex [N-Term];1	P04626	P04626 [1097-1111]	P04626 1xTMT6plex [N	0,00	-0,19	0,12
[R],KMEESDEEAVQAK,[V]	1xOxidation [M2];2xTM	Q9Y2K7	Q9Y2K7 [688-700]	Q9Y2K7 1xOxidation [M	0,01	-0,19	0,12
[K],YSPTSPTYSPTSPK,[YG]	1xTMT6plex [K14];1xTM	P24928	P24928 [1909-1922]; [1923-1936]	P24928 1xTMT6plex [K	0,01	-0,19	0,12
[R],SSVSSHRPK,[D]	1xTMT6plex [K9];1xTM	Q5VUA4	Q5VUA4 [8-16]	Q5VUA4 1xTMT6plex [	0,03	-0,19	0,14
[R],LLSGDSTEK,[R]	1xTMT6plex [K9];1xTM	Q6P0Q8	Q6P0Q8 [1030-1038]	Q6P0Q8 1xTMT6plex [	0,05	-0,19	0,16
[R],NSFTPLSSSNTIR,[R]	1xTMT6plex [N-Term];1	O60825	O60825 [465-477]	O60825 1xTMT6plex [N	0,04	-0,19	0,15
[R],ETEAAPTSPPIVPLK,[S]	1xTMT6plex [K15];1xTM	Q7Z3B3	Q7Z3B3 [1075-1089]	Q7Z3B3 1xTMT6plex [K	0,00	-0,19	0,12
[K],QQQEPTGEPSPK,[R]	1xTMT6plex [K12];1xTM	P52926	P52926 [35-46]	P52926 1xTMT6plex [K	0,01	-0,19	0,12
[R],SQSSHSYDDSTLPLIDR,[N]	1xTMT6plex [N-Term];1	O60716	O60716 [859-875]	O60716 1xTMT6plex [N	0,01	-0,19	0,12
[R],RFTPPSTALSPGK,[M]	1xTMT6plex [K13];1xTM	Q01196	Q01196 [12-24]	Q01196 1xTMT6plex [K	0,02	-0,19	0,13
[K],DADLSPVNASIIK,[E]	1xTMT6plex [K13];1xTM	O75970	O75970 [479-491]	O75970 1xTMT6plex [K	0,03	-0,19	0,14
[R],IDFIPVSPAPSPTR,[G]	1xTMT6plex [N-Term];1	Q96E09	Q96E09 [137-150]	Q96E09 1xTMT6plex [N	0,04	-0,19	0,15
[K],GHSPAQKPK,[T]	2xTMT6plex [K7; K9];1x	Q14966	Q14966 [603-611]	Q14966 2xTMT6plex [K	0,01	-0,19	0,12
[K],TPSPSQPK,[K]	1xTMT6plex [K8];1xTM	Q9NTI5	Q9NTI5 [1381-1388]	Q9NTI5 1xTMT6plex [K	0,00	-0,19	0,10
[R],SPYSGPK,[F]	1xTMT6plex [K7];1xTM	Q14498	Q14498 [97-103]	Q14498 1xTMT6plex [K	0,05	-0,19	0,16
[R],RATISSPLELEGTVSR,[H]	1xTMT6plex [N-Term];1	Q96GS4	Q96GS4 [194-209]	Q96GS4 1xTMT6plex [	0,01	-0,19	0,12
[K],FANLTPSR,[T]	1xTMT6plex [N-Term];1	O15027	O15027 [2050-2057]	O15027 1xTMT6plex [N	0,01	-0,19	0,12
[K],GPGEPDSPTPLHPPTPPILS	1xTMT6plex [N-Term];1	Q03164	Q03164 [1831-1853]	Q03164 1xTMT6plex [N	0,03	-0,19	0,14
[R],EVPHSSPPYYEFLWGPR,[A	1xTMT6plex [N-Term];1	Q9UBF1	Q9UBF1 [288-304]	Q9UBF1 1xTMT6plex [	0,02	-0,19	0,13
[R],GDPQQQSITHIAIPQEAYNA	1xTMT6plex [K34];1xTM	Q2TAL8	Q2TAL8 [320-353]	Q2TAL8 1xTMT6plex [K	0,01	-0,19	0,12
[K],APGSAGHYELPWVEK,[Y]	1xTMT6plex [K15];1xTM	P35250	P35250 [27-41]	P35250 1xTMT6plex [K	0,01	-0,19	0,12

[R],SIGSAVDQGNESIVAK,[T]	1xTMT6plex [K16];1xTM	Q9H0H5	Q9H0H5 [203-218]	Q9H0H5 1xTMT6plex [K	0,05	-0,19	0,16
[R],RGSGDTSISIDTEASIR,[E]	1xTMT6plex [N-Term];1	Q32MZ4	Q32MZ4 [118-134]	Q32MZ4 1xTMT6plex [N	0,05	-0,19	0,16
[K],IISNASCTTNCLAPLAK,[V]	2xCarbamidomethyl [C7	P04406	P04406 [146-162]	P04406 2xCarbamidom	0,02	-0,19	0,13
[K],NQLTSNPENTVFDAK,[R]	1xTMT6plex [K15];1xTM	P11021	P11021 [82-96]	P11021 1xTMT6plex [K	0,04	-0,19	0,15
[R],QLHLEGASLELSDDDTESK	1xTMT6plex [K19];1xTM	P35580	P35580 [1945-1963]	P35580 1xTMT6plex [K	0,04	-0,19	0,15
[R],FSEGVLQSPSQDQEK,[L]	1xTMT6plex [K15];1xTM	Q9C0C2	Q9C0C2 [428-442]	Q9C0C2 1xTMT6plex [K	0,02	-0,19	0,12
[K],VLSANHGDPSIQTSGSEQT	1xTMT6plex [K22];1xTM	Q9H694	Q9H694 [593-614]	Q9H694 1xTMT6plex [K	0,01	-0,19	0,12
[R],ALPAAHIPAPPHEGSPR,[D]	1xTMT6plex [N-Term];1	P17029	P17029 [194-210]	P17029 1xTMT6plex [N	0,02	-0,19	0,13
[K],SRTPPRR,[SR]	1xTMT6plex [N-Term];1	Q9UQ35	Q9UQ35 [808-814]; [1682-1688]	Q9UQ35 1xTMT6plex [N	0,01	-0,19	0,12
[K],TGSPGPELLFHEGQQK,[R]	1xTMT6plex [K16];1xTM	Q14202	Q14202 [462-477]	Q14202 1xTMT6plex [K	0,00	-0,19	0,11
[K],ATSSSNPSSPAPDWYK,[D]	1xTMT6plex [K16];1xTM	P46100	P46100 [1988-2003]	P46100 1xTMT6plex [K	0,01	-0,19	0,12
[K],VAAETQSPSLFGSTK,[L]	1xTMT6plex [K15];1xTM	Q9UKX7	Q9UKX7 [215-229]	Q9UKX7 1xTMT6plex [K	0,01	-0,19	0,12
[K],RALASNTSFFSGCSPIEEEA	1xCarbamidomethyl [C1	O95239	O95239 [1212-1232]	O95239 1xCarbamidom	0,01	-0,19	0,12
[R],SSPPAPPLPPGSGSPGTPQ	1xTMT6plex [N-Term];1	Q9Y3L3	Q9Y3L3 [585-607]	Q9Y3L3 1xTMT6plex [N	0,04	-0,19	0,15
[R],TTSISPALAR,[K]	1xTMT6plex [N-Term];1	Q9UKE5	Q9UKE5 [676-685]	Q9UKE5 1xTMT6plex [N	0,03	-0,19	0,13
[R],AGAPGALSPSYDGGLHGLQ	1xTMT6plex [K21];1xTM	P15923	P15923 [372-392]	P15923 1xTMT6plex [K	0,01	-0,19	0,12
[K],TTTPGPSLSQGVSVDEK,[L]	1xTMT6plex [K17];1xTM	O60934	O60934 [335-351]	O60934 1xTMT6plex [K	0,02	-0,19	0,12
[K],ITESSLVEITEHK,[D]	1xTMT6plex [K13];1xTM	Q99590	Q99590 [584-596]	Q99590 1xTMT6plex [K	0,04	-0,19	0,15
[K],AYHEQLSVAEITNACFEPAN	1xCarbamidomethyl [C1	Q71U36; P	Q71U36 [281-304]; P68366 [281-304	Q71U36 1xCarbamidom	0,01	-0,19	0,12
[R],RISFSASHR,[L]	1xTMT6plex [N-Term];1	Q03393	Q03393 [17-25]	Q03393 1xTMT6plex [N	0,01	-0,19	0,12
[R],KASSPSPLTIGTPESQR,[K]	1xTMT6plex [K1];1xTM	Q9NPI6	Q9NPI6 [520-536]	Q9NPI6 1xTMT6plex [K	0,01	-0,19	0,12
[K],SEWLDPSQKSPLHVGETR,	1xTMT6plex [K9];1xTM	O15164	O15164 [802-819]	O15164 1xTMT6plex [K	0,04	-0,19	0,15
[R],SLSPILPGR,[H]	1xTMT6plex [N-Term];1	Q6ZSZ5	Q6ZSZ5 [1289-1297]	Q6ZSZ5 1xTMT6plex [N	0,01	-0,19	0,12
[R],KDSLTQAQEQGNLLN,[-]	1xTMT6plex [K1];1xTM	Q5JTD0	Q5JTD0 [543-557]	Q5JTD0 1xTMT6plex [K	0,00	-0,19	0,11
[K],SPSPVQGK,[K]	1xTMT6plex [K8];1xTM	Q96JP5	Q96JP5 [101-108]	Q96JP5 1xTMT6plex [K	0,04	-0,18	0,15
[K],AHQSESYLPIGCK,[L]	1xCarbamidomethyl [C1	O75815	O75815 [335-347]	O75815 1xCarbamidom	0,02	-0,18	0,12
[K],QSSVTQVTEQSPK,[V]	1xTMT6plex [K13];1xTM	Q14966	Q14966 [118-130]	Q14966 1xTMT6plex [K	0,04	-0,18	0,16
[R],AFSFPVSPER,[G]	1xTMT6plex [N-Term];1	Q96B18	Q96B18 [4-13]	Q96B18 1xTMT6plex [N	0,00	-0,18	0,11
[R],RVSHSPPPK,[Q]	1xTMT6plex [K9];1xTM	Q8IYB3	Q8IYB3 [634-642]	Q8IYB3 1xTMT6plex [K	0,05	-0,18	0,16
[R],AHTGDGAKPDSPQK,[E]	2xTMT6plex [K8; K14];1	O15069	O15069 [1058-1071]	O15069 2xTMT6plex [K	0,02	-0,18	0,13
[R],VGTNYQAVIPECKPESPAR	1xCarbamidomethyl [C1	Q8IZ40	Q8IZ40 [48-66]	Q8IZ40 1xCarbamidome	0,01	-0,18	0,12
[R],QSSIFGGADVGFSGGIPSP	1xTMT6plex [K21];1xTM	Q9P2Y5	Q9P2Y5 [481-501]	Q9P2Y5 1xTMT6plex [K	0,02	-0,18	0,13
[R],ISSIYAR,[E]	1xTMT6plex [N-Term];1	Q14683	Q14683 [969-975]	Q14683 1xTMT6plex [N	0,04	-0,18	0,15
[R],TPSPAPK,[R]	1xTMT6plex [K7];1xTM	Q63ZY6	Q63ZY6 [258-264]	Q63ZY6 1xTMT6plex [K	0,02	-0,18	0,13
[K],LTSPVTSISPIQASEK,[T]	1xTMT6plex [K16];1xTM	Q7Z589	Q7Z589 [1128-1143]	Q7Z589 1xTMT6plex [K	0,02	-0,18	0,12
[R],SLAFEEGSQSTTISSLSEK,[	1xTMT6plex [K19];1xTM	Q13315	Q13315 [1974-1992]	Q13315 1xTMT6plex [K	0,03	-0,18	0,14
[K],AEEDEILNRSPR,[N]	1xTMT6plex [N-Term];1	P27824	P27824 [574-585]	P27824 1xTMT6plex [N	0,00	-0,18	0,12
[R],RSSDSWEVWGSASTNR,[N	1xTMT6plex [N-Term];1	Q8N6T3	Q8N6T3 [359-374]	Q8N6T3 1xTMT6plex [N	0,03	-0,18	0,14
[K],TEVVMNSQQTPVGTPK,[D]	1xTMT6plex [K16];1xTM	O75781	O75781 [132-147]	O75781 1xTMT6plex [K	0,02	-0,18	0,13
[R],SYSLSELSVLQAK,[S]	1xTMT6plex [K13];1xTM	Q9P0V3	Q9P0V3 [244-256]	Q9P0V3 1xTMT6plex [K	0,04	-0,18	0,15
[R],VMTIPYQPMPASSPVICAG	1xCarbamidomethyl [C1	Q15365	Q15365 [178-200]	Q15365 1xCarbamidom	0,04	-0,18	0,15
[R],DFTNEAPPAPLPDASASPLS	1xTMT6plex [N-Term];1	Q6WCQ1	Q6WCQ1 [346-369]	Q6WCQ1 1xTMT6plex	0,02	-0,18	0,13
[R],ATLSSIR,[H]	1xTMT6plex [N-Term];1	P46779	P46779 [88-94]	P46779 1xTMT6plex [N	0,03	-0,18	0,14
[K],QRSSPVTK,[R]	1xTMT6plex [K8];1xTM	Q8IYB3	Q8IYB3 [643-650]	Q8IYB3 1xTMT6plex [K	0,03	-0,18	0,14
[K],DIPRTPSR,[G]	1xTMT6plex [N-Term];1	Q9UQ35	Q9UQ35 [1468-1475]	Q9UQ35 1xTMT6plex [N	0,04	-0,18	0,16
[K],SKSPPKVPIVIQDDSLPAGP	2xTMT6plex [K2; K6];1x	Q7Z422	Q7Z422 [37-61]	Q7Z422 2xTMT6plex [K	0,02	-0,18	0,13
[R],SLLLSGLR,[A]	1xTMT6plex [N-Term];1	Q96JJ6	Q96JJ6 [222-229]	Q96JJ6 1xTMT6plex [N	0,05	-0,18	0,16
[K],LTGSTSSLNK,[L]	1xTMT6plex [K10];1xTM	Q9NV70	Q9NV70 [467-476]	Q9NV70 1xTMT6plex [K	0,00	-0,18	0,11
[R],IGSPLSPK,[K]	1xTMT6plex [K8];1xTM	Q659C4	Q659C4 [338-345]	Q659C4 1xTMT6plex [K	0,02	-0,18	0,13
[K],GILSLPHQASPVSR,[T]	1xTMT6plex [N-Term];1	O75925	O75925 [494-507]	O75925 1xTMT6plex [N	0,01	-0,18	0,12
[R],QRPVPQPSSASLDEYTLMR	1xTMT6plex [N-Term];1	Q9Y4H2	Q9Y4H2 [584-602]	Q9Y4H2 1xTMT6plex [N	0,02	-0,18	0,13
[R],QRPVPQPSSASLDEYTLMR	1xTMT6plex [N-Term];1	Q9Y4H2	Q9Y4H2 [584-602]	Q9Y4H2 1xTMT6plex [N	0,02	-0,18	0,13
[K],AALPSLTNPPSLSPIR,[R]	1xTMT6plex [N-Term];1	P42858	P42858 [1167-1182]	P42858 1xTMT6plex [N	0,03	-0,18	0,14
[K],SPSLSPSPPSPLEK,[T]	1xTMT6plex [K14];1xTM	P46821	P46821 [1256-1269]	P46821 1xTMT6plex [K	0,01	-0,18	0,12
[K],SERPPTILMTEEPSSPK,[G]	1xTMT6plex [K17];1xTM	Q5VZK9	Q5VZK9 [1080-1096]	Q5VZK9 1xTMT6plex [K	0,00	-0,18	0,11
[R],NFSFMNPGMER,[L]	1xOxidation [M5];1xTMT	Q04759	Q04759 [693-703]	Q04759 1xOxidation [M	0,04	-0,18	0,15
[R],IQHLSTIDYVEDGK,[G]	1xTMT6plex [K14];1xTM	Q9BZ67	Q9BZ67 [414-427]	Q9BZ67 1xTMT6plex [K	0,04	-0,18	0,15

[K],TAVSVTQGGHSR,[T]	1xTMT6plex [N-Term];1	P40425	P40425 [320-331]	P40425 1xTMT6plex [N	0,01	-0,18	0,12
[R],ISHEGSPVKPVAIR,[E]	1xTMT6plex [K9];1xTM	Q96II8	Q96II8 [414-427]	Q96II8 1xTMT6plex [K4	0,04	-0,18	0,15
[R],KRSWGHESPEER,[H]	1xTMT6plex [K1];1xTM	Q9UKJ3	Q9UKJ3 [1007-1018]	Q9UKJ3 1xTMT6plex [K	0,03	-0,18	0,14
[R],GVLHTFSPSPK,[L]	1xTMT6plex [K11];1xTM	Q86YP4	Q86YP4 [540-550]	Q86YP4 1xTMT6plex [K	0,03	-0,18	0,14
[R],GGPASVPSSSPGTSVK,[K]	1xTMT6plex [K16];1xTM	O43237	O43237 [398-413]	O43237 1xTMT6plex [K	0,04	-0,18	0,15
[K],SKPIPIMPASPQK,[G]	1xOxidation [M7];2xTMT	O00429	O00429 [607-619]	O00429 1xOxidation [M	0,01	-0,18	0,12
[R],TLLTGDGGGEATGSPLAQ(	1xTMT6plex [K20];1xTM	Q6WCQ1	Q6WCQ1 [878-897]	Q6WCQ1 1xTMT6plex	0,02	-0,18	0,13
[R],SLMSSPEDLTK,[D]	1xOxidation [M3];1xTMT	P46821	P46821 [828-838]	P46821 1xOxidation [M:	0,05	-0,18	0,16
[K],AVDKPPSPSPIEMK,[K]	1xOxidation [M13];2xTM	Q9H165	Q9H165 [80-93]	Q9H165 1xOxidation [M	0,04	-0,18	0,16
[K],GIGTPPNTTPIK,[N]	1xTMT6plex [K12];1xTM	O96028	O96028 [107-118]	O96028 1xTMT6plex [K	0,03	-0,18	0,14
[R],GICDYFPSPSK,[T]	1xCarbamidomethyl [C3	Q86XL3	Q86XL3 [252-262]	Q86XL3 1xCarbamidom	0,04	-0,18	0,15
[R],IGSVTSR,[Q]	1xTMT6plex [N-Term];1	Q99569	Q99569 [288-294]	Q99569 1xTMT6plex [N	0,01	-0,18	0,12
[R],YFQSPSRSR,[S]	1xTMT6plex [N-Term];2	Q16629	Q16629 [189-197]	Q16629 1xTMT6plex [N	0,02	-0,18	0,13
[R],SFSADNFIGIQR,[S]	1xTMT6plex [N-Term];1	Q8N7R7	Q8N7R7 [342-353]	Q8N7R7 1xTMT6plex [N	0,00	-0,18	0,12
[K],NSSPAPPQPAPGK,[V]	1xTMT6plex [K13];1xTM	Q9NYV4	Q9NYV4 [1081-1093]	Q9NYV4 1xTMT6plex [I	0,02	-0,18	0,12
[R],RNSVTPLASPEPTK,[K]	1xTMT6plex [K14];1xTM	Q16875	Q16875 [459-472]	Q16875 1xTMT6plex [K	0,01	-0,18	0,12
[K],EISVIQHTSSFEK,[S]	1xTMT6plex [K13];1xTM	Q5T1R4	Q5T1R4 [796-808]	Q5T1R4 1xTMT6plex [K	0,03	-0,17	0,14
[K],IEIPVTPTGQSVPSSPSIPG1	1xTMT6plex [K25];1xTM	Q9H4Z3	Q9H4Z3 [130-154]	Q9H4Z3 1xTMT6plex [K	0,02	-0,17	0,12
[R],FHFPPLDTHSPTNDVQPGF	1xTMT6plex [N-Term];1	P41162	P41162 [150-168]	P41162 1xTMT6plex [N	0,04	-0,17	0,15
[R],GPSPSLLNTISLDTMPK,[F]	1xTMT6plex [K17];1xTM	O60307	O60307 [772-788]	O60307 1xTMT6plex [K	0,04	-0,17	0,15
[K],IVSGSPISTPSPSPLPR,[T]	1xTMT6plex [N-Term];2	Q9ULM3	Q9ULM3 [461-477]	Q9ULM3 1xTMT6plex [I	0,03	-0,17	0,13
[R],EGTGALEKPDPVAAGSPGI	2xTMT6plex [K8; K20];1	Q96H86	Q96H86 [116-135]	Q96H86 2xTMT6plex [K	0,02	-0,17	0,12
[K],IAAPELHKGDSDSEEDEPTI	2xTMT6plex [K8; K20];1	Q92733	Q92733 [147-166]	Q92733 2xTMT6plex [K	0,02	-0,17	0,13
[K],IAAPELHKGDSDSEEDEPTI	2xTMT6plex [K8; K20];1	Q92733	Q92733 [147-166]	Q92733 2xTMT6plex [K	0,02	-0,17	0,13
[R],AIGSTSKPQESPK,[G]	2xTMT6plex [K7; K13];1	P18887	P18887 [231-243]	P18887 2xTMT6plex [K	0,03	-0,17	0,14
[K],SLAPDRSDDEHDPLDNTSR	1xTMT6plex [N-Term];1	Q92614	Q92614 [2014-2034]	Q92614 1xTMT6plex [N	0,00	-0,17	0,12
[R],GSVHSLDAGLLLPSGDPFS	1xTMT6plex [K20];1xTM	Q15276	Q15276 [373-392]	Q15276 1xTMT6plex [K	0,01	-0,17	0,12
[R],MVIQLRPGRSPEPDGPAPA	1xOxidation [M1];1xTMT	Q9Y4F5	Q9Y4F5 [844-865]	Q9Y4F5 1xOxidation [M	0,02	-0,17	0,12
[R],HSSISPSTLTLK,[S]	1xTMT6plex [K12];1xTM	Q14004	Q14004 [435-446]	Q14004 1xTMT6plex [K	0,03	-0,17	0,15
[R],VASVASSLAAR,[G]	1xTMT6plex [N-Term];1	Q96JQ0	Q96JQ0 [3053-3063]	Q96JQ0 1xTMT6plex [N	0,00	-0,17	0,11
[R],SSAQTSAAGATATTSTSST	1xTMT6plex [K35];1xTM	Q9ULU4	Q9ULU4 [766-800]	Q9ULU4 1xTMT6plex [K	0,05	-0,17	0,16
[R],QLPSPEPCEIQK,[N]	1xCarbamidomethyl [C8	Q5VVJ2	Q5VVJ2 [337-348]	Q5VVJ2 1xCarbamidom	0,04	-0,17	0,16
[R],SQPAPVSPQQK,[S]	1xTMT6plex [K11];1xTM	Q13118	Q13118 [243-253]	Q13118 1xTMT6plex [K	0,03	-0,17	0,14
[R],ANSDIISLNFR,[S]	1xTMT6plex [N-Term];1	Q8TC76	Q8TC76 [299-309]	Q8TC76 1xTMT6plex [N	0,03	-0,17	0,14
[K],SSFLSSPER,[A]	1xTMT6plex [N-Term];1	Q4AC94	Q4AC94 [2127-2135]	Q4AC94 1xTMT6plex [N	0,00	-0,17	0,12
[R],KDGSSPPLLEK,[Q]	2xTMT6plex [K1; K11];1	Q6W2J9	Q6W2J9 [419-429]	Q6W2J9 2xTMT6plex [I	0,01	-0,17	0,12
[K],EQDRVHSPCPTSGSEK,[K]	1xCarbamidomethyl [C9	Q96LT9	Q96LT9 [102-117]	Q96LT9 1xCarbamidom	0,02	-0,17	0,12
[K],IQHVTWP,[-]	1xTMT6plex [N-Term];1	Q9BVC5	Q9BVC5 [226-232]	Q9BVC5 1xTMT6plex [I	0,00	-0,17	0,11
[K],TKGDDTDTRDDISILATGCK	1xCarbamidomethyl [C1	Q12888	Q12888 [586-605]	Q12888 1xCarbamidom	0,00	-0,17	0,11
[K],SPFLPLAPQTETQK,[N]	1xTMT6plex [K14];1xTM	Q96L73	Q96L73 [224-237]	Q96L73 1xTMT6plex [K	0,04	-0,17	0,15
[R],AFSYYGPLR,[T]	1xTMT6plex [N-Term];1	Q16629	Q16629 [30-38]	Q16629 1xTMT6plex [N	0,04	-0,17	0,15
[K],DSPGIPPSAGAHQLFR,[G]	1xTMT6plex [N-Term];1	Q15418	Q15418 [362-377]	Q15418 1xTMT6plex [N	0,00	-0,17	0,12
[K],VASEAPLEHKPQVEASSPR	1xTMT6plex [K10];1xTM	Q8TD19	Q8TD19 [853-871]	Q8TD19 1xTMT6plex [K	0,02	-0,17	0,13
[R],RTPSDDEEDNLFAPPK,[L]	1xTMT6plex [K16];1xTM	Q9Y4E1; (	Q9Y4E1 [330-345]; Q641Q2 [330-34	Q9Y4E1 1xTMT6plex [K	0,01	-0,17	0,12
[R],ASPSPQPSSQPLQIHR,[Q]	1xTMT6plex [N-Term];1	Q9HC35	Q9HC35 [143-158]	Q9HC35 1xTMT6plex [N	0,01	-0,17	0,12
[R],TASPLPLRPDAPVEK,[S]	1xTMT6plex [K15];1xTM	Q9HCK8	Q9HCK8 [1993-2007]	Q9HCK8 1xTMT6plex [I	0,05	-0,17	0,16
[K],VTENGGSPQGIK,[S]	1xTMT6plex [K12];1xTM	Q92766	Q92766 [30-41]	Q92766 1xTMT6plex [K	0,02	-0,17	0,12
[K],SVIDTSTIVR,[K]	1xTMT6plex [N-Term];1	O43815	O43815 [259-268]	O43815 1xTMT6plex [N	0,01	-0,17	0,12
[R],VPLTPTR,[G]	1xTMT6plex [N-Term];1	Q68CZ2	Q68CZ2 [629-635]	Q68CZ2 1xTMT6plex [N	0,03	-0,17	0,13
[R],APSIQAK,[L]	1xTMT6plex [K7];1xTM	Q5XKK7	Q5XKK7 [64-70]	Q5XKK7 1xTMT6plex [K	0,02	-0,17	0,13
[R],QQSTPQAANSPPNLGAK,[I	1xTMT6plex [K17];1xTM	Q9Y4C1	Q9Y4C1 [316-332]	Q9Y4C1 1xTMT6plex [K	0,01	-0,17	0,12
[K],NYVPSYDPSSPQTSQSWY	1xTMT6plex [N-Term];1	O00712	O00712 [401-420]	O00712 1xTMT6plex [N	0,02	-0,17	0,13
[K],SYSSSETLK,[A]	1xTMT6plex [K9];1xTM	Q9P273; (	Q9P273 [40-48]; Q6N022 [38-46]	Q9P273 1xTMT6plex [K	0,03	-0,17	0,14
[K],NKPGPNIESGNEDDDASFK	2xTMT6plex [K2; K19];1	O60841	O60841 [206-224]	O60841 2xTMT6plex [K	0,04	-0,17	0,15
[K],ELSPPPGLPSK,[I]	1xTMT6plex [K11];1xTM	Q7Z4S6	Q7Z4S6 [1210-1220]	Q7Z4S6 1xTMT6plex [K	0,05	-0,17	0,17
[R],LLNLQDSDSEECTSR,[K]	1xCarbamidomethyl [C1	Q52LR7	Q52LR7 [532-546]	Q52LR7 1xCarbamidom	0,02	-0,17	0,13
[K],GGLLTSEEDSGFSTSPKDN	2xTMT6plex [K17; K23]	Q9UQR1	Q9UQR1 [292-314]	Q9UQR1 2xTMT6plex [	0,04	-0,17	0,15

[K],GGLLTSEEDSGFSTSPKDN	2xTMT6plex [K17; K23]	Q9UQR1	Q9UQR1 [292-314]	Q9UQR1 2xTMT6plex [	0,04	-0,17	0,15
[K],SPPLSPVGTTPVK,[L]	1xTMT6plex [K13];1xTM	Q9BVC5	Q9BVC5 [189-201]	Q9BVC5 1xTMT6plex [I	0,03	-0,16	0,14
[K],NRPCLSPK,[H]	1xCarbamidomethyl [C4	P10636	P10636 [406-413]	P10636 1xCarbamidom	0,05	-0,16	0,16
[R],LNFSHGTHEYHAETIK,[N]	1xTMT6plex [K16];1xTM	P14618	P14618 [74-89]	P14618 1xTMT6plex [K	0,01	-0,16	0,12
[R],TRDPTPRPPGLPEEATALA	1xTMT6plex [N-Term];1	Q96GP6	Q96GP6 [721-745]	Q96GP6 1xTMT6plex [N	0,01	-0,16	0,12
[R],SCSASCVPHGAK,[D]	2xCarbamidomethyl [C2	P00519	P00519 [716-727]	P00519 2xCarbamidom	0,02	-0,16	0,12
[K],GLGHPPSSPLLK,[K]	1xTMT6plex [K12];1xTM	O95785	O95785 [976-987]	O95785 1xTMT6plex [K	0,02	-0,16	0,13
[K],AEVPGATGGDSPHLQPAEI	1xTMT6plex [N-Term];1	Q9P2K5	Q9P2K5 [7-30]	Q9P2K5 1xTMT6plex [N	0,03	-0,16	0,13
[K],APSTPVPPSPAPAPGLTK,[I	1xTMT6plex [K18];1xTM	Q96EZ8	Q96EZ8 [100-117]	Q96EZ8 1xTMT6plex [K	0,04	-0,16	0,15
[R],VPVNLLNSPDCDVK,[T]	1xCarbamidomethyl [C1	P33981	P33981 [274-287]	P33981 1xCarbamidom	0,04	-0,16	0,15
[K],SPTLSQVHSPLVTSPSANLI	2xPhospho [S1; T13];1x	Q86UU0	Q86UU0 [934-953]	Q86UU0 2xPhospho [S9	0,04	-0,16	0,15
[K],SPTLSQVHSPLVTSPSANLI	2xPhospho [T3; T13];1x	Q86UU0	Q86UU0 [934-953]	Q86UU0 2xPhospho [T9	0,04	-0,16	0,15
[K],SPTLSQVHSPLVTSPSANLI	2xPhospho [S1; S14];1x	Q86UU0	Q86UU0 [934-953]	Q86UU0 2xPhospho [S9	0,04	-0,16	0,15
[K],LEGQGDVPTPK,[Q]	1xTMT6plex [K11];1xTM	Q9Y2Z0	Q9Y2Z0 [257-267]	Q9Y2Z0 1xTMT6plex [K	0,03	-0,16	0,14
[K],YGLQDSDEEEEEHPSK,[T]	1xTMT6plex [K16];1xTM	P52948	P52948 [883-898]	P52948 1xTMT6plex [K	0,01	-0,16	0,12
[K],QSSLAEPVSPSK,[K]	1xTMT6plex [K12];1xTM	O43572	O43572 [179-190]	O43572 1xTMT6plex [K	0,04	-0,16	0,16
[K],GNVFSSPTAAGTPNK,[E]	1xTMT6plex [K15];1xTM	Q05682	Q05682 [719-733]	Q05682 1xTMT6plex [K	0,02	-0,16	0,12
[R],FKPESSSPVK,[K]	2xTMT6plex [K2; K10];1	Q5TCQ9	Q5TCQ9 [1444-1453]	Q5TCQ9 2xTMT6plex [	0,05	-0,16	0,16
[K],ASIGQSPGLPSTTFK,[L]	1xTMT6plex [K15];1xTM	Q5VT52	Q5VT52 [609-623]	Q5VT52 1xTMT6plex [K	0,03	-0,16	0,14
[R],IVEYSPPSAPR,[R]	1xTMT6plex [N-Term];1	O95696	O95696 [124-134]	O95696 1xTMT6plex [N	0,02	-0,16	0,13
[R],SSPQLAK,[L]	1xTMT6plex [K7];1xTM	Q13370	Q13370 [980-986]	Q13370 1xTMT6plex [K	0,04	-0,16	0,15
[K],RASGQAFELILSPR,[S]	1xTMT6plex [N-Term];2	P16949	P16949 [14-27]	P16949 1xTMT6plex [N	0,03	-0,16	0,13
[R],SPAAGSSQSSGWPNDVDA	1xTMT6plex [K24];1xTM	Q14938	Q14938 [301-324]	Q14938 1xTMT6plex [K	0,04	-0,16	0,15
[R],GVISYQTLPR,[N]	1xTMT6plex [N-Term];1	Q9HAU0	Q9HAU0 [432-441]	Q9HAU0 1xTMT6plex [I	0,02	-0,16	0,13
[K],DRSLWNLHQGK,[Q]	1xTMT6plex [K11];1xTM	O94964	O94964 [1219-1229]	O94964 1xTMT6plex [K	0,01	-0,16	0,12
[R],LGSATPK,[I]	1xTMT6plex [K7];1xTM	P68543	P68543 [140-146]	P68543 1xTMT6plex [K	0,03	-0,16	0,14
[R],LGSATPK,[I]	1xTMT6plex [K7];1xTM	P68543	P68543 [140-146]	P68543 1xTMT6plex [K	0,03	-0,16	0,14
[K],SSVLPSPSGR,[S]	1xTMT6plex [N-Term];1	P35658	P35658 [641-650]	P35658 1xTMT6plex [N	0,01	-0,16	0,12
[R],TPAAAAAMNLASPR,[T]	1xOxidation [M8];1xTMT	Q9UQ35	Q9UQ35 [2261-2274]	Q9UQ35 1xOxidation [M	0,04	-0,16	0,16
[K],GPSPPGAK,[R]	1xTMT6plex [K8];1xTM	Q96S55	Q96S55 [63-70]	Q96S55 1xTMT6plex [K	0,04	-0,16	0,15
[R],AHASPFSGALTPSAPPGPE	1xTMT6plex [N-Term];1	O15027	O15027 [119-140]	O15027 1xTMT6plex [N	0,03	-0,16	0,14
[R],DLEPHSFGGLLEGIR,[G]	1xTMT6plex [N-Term];1	Q9Y3Q8	Q9Y3Q8 [99-113]	Q9Y3Q8 1xTMT6plex [N	0,03	-0,16	0,14
[K],FLQSYPGRGSGSDDTELR,	1xTMT6plex [N-Term];1	Q13948	Q13948 [553-570]	Q13948 1xTMT6plex [N	0,01	-0,16	0,12
[R],RRSDSTLFSTVDTDEIPAK,[	1xTMT6plex [K19];1xTM	Q9HC62	Q9HC62 [30-48]	Q9HC62 1xTMT6plex [K	0,04	-0,16	0,15
[R],RGEGDAPFSEPGTTSTQRI	1xTMT6plex [K27];1xTM	Q9UQ35	Q9UQ35 [303-329]	Q9UQ35 1xTMT6plex [I	0,00	-0,16	0,12
[K],YTPTSPSYSPSSPEYTPTSI	1xTMT6plex [K21];1xTM	P24928	P24928 [1839-1859]	P24928 1xTMT6plex [K	0,01	-0,15	0,12
[K],VVESLPSSSSEQSPLQK,[Q	1xTMT6plex [K17];1xTM	Q6PJQ5	Q6PJQ5 [127-143]	Q6PJQ5 1xTMT6plex [K	0,04	-0,15	0,15
[R],SRSPPRPK,[W]	1xTMT6plex [K8];1xTM	Q8WUQ7	Q8WUQ7 [71-78]	Q8WUQ7 1xTMT6plex	0,01	-0,15	0,12
[R],TTSISPALAR,[K]	1xTMT6plex [N-Term];1	Q9UKE5	Q9UKE5 [676-685]	Q9UKE5 1xTMT6plex [I	0,04	-0,15	0,15
[R],ALSSGGSITSPPLSPALPK,[	1xTMT6plex [K19];1xTM	Q6Y7W6	Q6Y7W6 [17-35]	Q6Y7W6 1xTMT6plex [	0,05	-0,15	0,16
[K],QNSPVAPTAQPK,[A]	1xTMT6plex [K12];1xTM	Q6P1N0	Q6P1N0 [453-464]	Q6P1N0 1xTMT6plex [K	0,03	-0,15	0,14
[R],ADGAPSAAPPDGLLASPDL	1xTMT6plex [K23];1xTM	P17535	P17535 [75-97]	P17535 1xTMT6plex [K	0,00	-0,15	0,11
[K],KPGDGEVSPSTEDAPFQHS	2xTMT6plex [K1; K23];1	O60318	O60318 [520-542]	O60318 2xTMT6plex [K	0,01	-0,15	0,12
[R],GPGAPGLAHLQESQAGSD	1xTMT6plex [K25];1xTM	Q14676	Q14676 [360-384]	Q14676 1xTMT6plex [K	0,02	-0,15	0,13
[K],ELPPSPEKK,[T]	2xTMT6plex [K8; K9];1x	P27816	P27816 [692-700]	P27816 2xTMT6plex [K	0,05	-0,15	0,16
[K],TVFPGAVPVLPASPPPK,[D]	1xTMT6plex [K17];1xTM	Q9UBC2	Q9UBC2 [217-233]	Q9UBC2 1xTMT6plex [I	0,03	-0,15	0,14
[R],RRSPSPYYSR,[YG]	1xTMT6plex [N-Term];2	Q13595; P	Q13595 [258-267]; P62995 [262-271]	Q13595 1xTMT6plex [N	0,01	-0,15	0,12
[R],KSPLVPR,[S]	1xTMT6plex [K1];1xTM	P29375	P29375 [1422-1428]	P29375 1xTMT6plex [K	0,03	-0,15	0,14
[K],AEAAPGPMSQAAPLASDSL	1xTMT6plex [K21];1xTM	Q96JQ0	Q96JQ0 [2968-2988]	Q96JQ0 1xTMT6plex [K	0,03	-0,15	0,13
[R],RLSTSPDVIQGHQPR,[D]	1xTMT6plex [N-Term];1	Q9Y385	Q9Y385 [264-278]	Q9Y385 1xTMT6plex [N	0,03	-0,15	0,14
[K],SLGSASPGPGQPPLSSPTR	1xTMT6plex [N-Term];1	Q9C0B5	Q9C0B5 [679-697]	Q9C0B5 1xTMT6plex [N	0,01	-0,15	0,12
[K],DSLITPHVSR,[S]	1xTMT6plex [N-Term];1	P49792; Q	P49792 [2446-2455]; Q99666 [1470-1	P49792 1xTMT6plex [N	0,02	-0,15	0,13
[R],GPSPAPASSPK,[R]	1xTMT6plex [K11];1xTM	Q9H7N4	Q9H7N4 [717-727]	Q9H7N4 1xTMT6plex [K	0,02	-0,15	0,13
[K],SLVGTPYWMAPELISR,[L]	1xOxidation [M9];1xTMT	O96013	O96013 [474-489]	O96013 1xOxidation [M	0,01	-0,15	0,12
[K],SPTPPNLPSDK,[I]	1xTMT6plex [K11];1xTM	A7KAX9	A7KAX9 [1234-1244]	A7KAX9 1xTMT6plex [K	0,03	-0,15	0,14
[R],LSLSPLR,[G]	1xTMT6plex [N-Term];1	Q66K74	Q66K74 [654-660]	Q66K74 1xTMT6plex [N	0,04	-0,15	0,16
[R],RSTVLGLPQHVQK,[E]	1xTMT6plex [K13];1xTM	Q9P206	Q9P206 [160-172]	Q9P206 1xTMT6plex [K	0,03	-0,15	0,14

[K],ATLTASPLGAS,[-]	1xTMT6plex [N-Term];1	Q13111	Q13111 [946-956]	Q13111 1xTMT6plex [N	0,00	-0,15	0,12
[R],SAPASPTHPGLMSPR,[S]	1xOxidation [M12];1xTM	P85037	P85037 [416-430]	P85037 1xOxidation [M	0,01	-0,15	0,12
[R],QSHSESPSLQSK,[S]	1xTMT6plex [K12];1xTM	Q9UQ35	Q9UQ35 [1078-1089]	Q9UQ35 1xTMT6plex [	0,04	-0,15	0,15
[R],SYSVEQLQPAPPGLTSQSF	1xTMT6plex [N-Term];1	Q5VZ46	Q5VZ46 [1026-1044]	Q5VZ46 1xTMT6plex [N	0,05	-0,15	0,16
[R],ASQGPVYK,[G]	1xTMT6plex [K8];1xTM	Q9Y2V2	Q9Y2V2 [57-64]	Q9Y2V2 1xTMT6plex [K	0,02	-0,15	0,13
[K],MAESPCSPSGQQPPSPPS	1xCarbamidomethyl [C6	Q14160	Q14160 [1292-1318]	Q14160 1xCarbamidom	0,03	-0,15	0,13
[R],ATAPQTQHVSPMR,[Q]	1xTMT6plex [N-Term];1	P29692	P29692 [124-136]	P29692 1xTMT6plex [N	0,03	-0,15	0,14
[K],LSEAVWQPEEHYSSSPEK,	1xTMT6plex [K18];1xTM	Q9Y485	Q9Y485 [904-921]	Q9Y485 1xTMT6plex [K	0,04	-0,15	0,16
[R],RLQDSFASETNLDFR,[S]	1xTMT6plex [N-Term];1	Q92621	Q92621 [1935-1949]	Q92621 1xTMT6plex [N	0,04	-0,15	0,15
[R],APTVPPPLPPTPPQPAR,[R	1xTMT6plex [N-Term];1	Q9Y3L3	Q9Y3L3 [616-632]	Q9Y3L3 1xTMT6plex [N	0,04	-0,15	0,15
[K],VFVGGLSPDTSEEQIK,[E]	1xTMT6plex [K16];1xTM	O14979	O14979 [235-250]	O14979 1xTMT6plex [K	0,03	-0,15	0,14
[R],STSMLISSGHNK,[S]	1xTMT6plex [K12];1xTM	Q9Y485	Q9Y485 [572-583]	Q9Y485 1xTMT6plex [K	0,00	-0,15	0,12
[R],RFTPPSPAFPCGGGGGK,[	1xCarbamidomethyl [C1	Q13761	Q13761 [12-28]	Q13761 1xCarbamidom	0,04	-0,15	0,15
[R],SPSPTLGESLAPHK,[G]	1xTMT6plex [K14];1xTM	Q86UU1	Q86UU1 [518-531]	Q86UU1 1xTMT6plex [K	0,03	-0,15	0,13
[K],ESHSPFGLDSFNSTAK,[V]	1xTMT6plex [K16];1xTM	Q7LBC6	Q7LBC6 [1250-1265]	Q7LBC6 1xTMT6plex [K	0,03	-0,14	0,14
[K],AASSPSNPAALPVASDSSP	1xTMT6plex [K23];1xTM	Q7Z5J4	Q7Z5J4 [1105-1127]	Q7Z5J4 1xTMT6plex [K	0,02	-0,14	0,13
[R],TASPPPPPK,[R]	1xTMT6plex [K9];1xTM	Q8IYB3	Q8IYB3 [614-622]	Q8IYB3 1xTMT6plex [K	0,02	-0,14	0,13
[K],MNIASPGTVHK,[R]	1xOxidation [M1];1xTM	Q9NRH2	Q9NRH2 [514-524]	Q9NRH2 1xOxidation [M	0,00	-0,14	0,12
[K],LFEDDDSNEK,[L]	1xTMT6plex [K10];1xTM	O43719	O43719 [696-705]	O43719 1xTMT6plex [K	0,01	-0,14	0,12
[K],GSQPPPAAESQSSLRR,[Q]	1xTMT6plex [N-Term];1	O43491	O43491 [46-61]	O43491 1xTMT6plex [N	0,03	-0,14	0,14
[R],SSSFSLPSR,[A]	1xTMT6plex [N-Term];1	Q14153	Q14153 [165-173]	Q14153 1xTMT6plex [N	0,02	-0,14	0,13
[R],SGAHSSASPPR,[S]	1xTMT6plex [N-Term];1	P18615	P18615 [174-184]	P18615 1xTMT6plex [N	0,00	-0,14	0,12
[K],RQSQQLEALQQQVK,[Q]	1xTMT6plex [K14];1xTM	Q9P0K7	Q9P0K7 [913-926]	Q9P0K7 1xTMT6plex [K	0,00	-0,14	0,12
[K],EKTPELPEPSVK,[V]	2xTMT6plex [K2; K12];1	Q8IYB3	Q8IYB3 [218-229]	Q8IYB3 2xTMT6plex [K	0,01	-0,14	0,12
[R],SRSDIDVNAAASAK,[S]	1xTMT6plex [K14];1xTM	Q7Z460	Q7Z460 [598-611]	Q7Z460 1xTMT6plex [K	0,02	-0,14	0,12
[R],SMGASPK,[L]	1xTMT6plex [K7];1xTM	Q9P2P5	Q9P2P5 [440-446]	Q9P2P5 1xTMT6plex [K	0,03	-0,14	0,14
[R],LPSAESLSR,[R]	1xTMT6plex [N-Term];1	O43281	O43281 [296-304]	O43281 1xTMT6plex [N	0,01	-0,14	0,12
[K],NLEQILNGGESPK,[Q]	1xTMT6plex [K13];1xTM	Q13033	Q13033 [219-231]	Q13033 1xTMT6plex [K	0,03	-0,14	0,14
[R],CRSPGMLEPLGSSR,[T]	1xCarbamidomethyl [C1	Q9UQ35	Q9UQ35 [2130-2143]	Q9UQ35 1xCarbamidon	0,01	-0,14	0,12
[R],CRSPGMLEPLGSSR,[T]	1xCarbamidomethyl [C1	Q9UQ35	Q9UQ35 [2130-2143]	Q9UQ35 1xCarbamidon	0,04	-0,14	0,16
[K],STSTAPDGAALTPPSPSFAT	1xTMT6plex [N-Term];1	Q14938	Q14938 [452-479]	Q14938 1xTMT6plex [N	0,05	-0,14	0,16
[K],AGMSSNQSISSPVLDAVPR	1xTMT6plex [N-Term];1	Q9UQ35	Q9UQ35 [1394-1416]	Q9UQ35 1xTMT6plex [	0,05	-0,14	0,16
[M],SSSPVNVK,[K]	1xTMT6plex [K8];1xTM	Q00839	Q00839 [2-9]	Q00839 1xTMT6plex [K	0,04	-0,14	0,16
[K],SSPMTVK,[S]	1xTMT6plex [K7];1xTM	Q9H165	Q9H165 [431-437]	Q9H165 1xTMT6plex [K	0,03	-0,14	0,13
[R],TRLSPPR,[A]	1xTMT6plex [N-Term];1	Q96PK6	Q96PK6 [579-585]	Q96PK6 1xTMT6plex [N	0,01	-0,14	0,12
[K],TPTSSPASSPLVAK,[K]	1xTMT6plex [K14];1xTM	Q14684	Q14684 [728-741]	Q14684 1xTMT6plex [K	0,03	-0,14	0,14
[R],KPSVSEEVQATPNK,[A]	2xTMT6plex [K1; K14];1	Q9UKJ3	Q9UKJ3 [1105-1118]	Q9UKJ3 2xTMT6plex [K	0,02	-0,14	0,13
[R],HISENSFGPSGGLR,[E]	1xTMT6plex [N-Term];1	Q12923	Q12923 [1227-1240]	Q12923 1xTMT6plex [N	0,00	-0,14	0,12
[K],VSDSPSHIATK,[T]	1xTMT6plex [K11];1xTM	Q9P2D3	Q9P2D3 [1734-1744]	Q9P2D3 1xTMT6plex [K	0,04	-0,14	0,16
[K],LPQSSSSESSPPSPQPTK,[	1xTMT6plex [K18];1xTM	Q9UQ35	Q9UQ35 [412-429]	Q9UQ35 1xTMT6plex [	0,05	-0,14	0,16
[R],HLLTHQGQSPR,[N]	1xTMT6plex [N-Term];1	Q96N77	Q96N77 [418-428]	Q96N77 1xTMT6plex [N	0,00	-0,14	0,11
[R],ALSLDDK,[G]	1xTMT6plex [K7];1xTM	Q9NRR6	Q9NRR6 [83-89]	Q9NRR6 1xTMT6plex [	0,01	-0,14	0,12
[K],STMPTSLPNLAK,[E]	1xTMT6plex [K12];1xTM	Q04721	Q04721 [2110-2121]	Q04721 1xTMT6plex [K	0,01	-0,14	0,12
[K],SLPAPVAQRPDSPGGGLQ/	1xTMT6plex [K23];1xTM	Q5JVS0	Q5JVS0 [97-119]	Q5JVS0 1xTMT6plex [K	0,01	-0,14	0,12
[R],VSPLNLSSVTP,[-]	1xTMT6plex [N-Term];1	Q9UJX2	Q9UJX2 [587-597]	Q9UJX2 1xTMT6plex [N	0,01	-0,14	0,12
[R],VPCNVEGISPELEK,[V]	1xCarbamidomethyl [C3	Q86VQ1	Q86VQ1 [295-308]	Q86VQ1 1xCarbamidon	0,03	-0,14	0,14
[R],GGSPLAAPQGGSPTK,[L]	1xTMT6plex [K15];1xTM	Q9UQB3	Q9UQB3 [265-279]	Q9UQB3 1xTMT6plex [	0,02	-0,14	0,13
[K],LEPQELSPLSATVFPK,[V]	1xTMT6plex [K16];1xTM	Q3T8J9	Q3T8J9 [1249-1264]	Q3T8J9 1xTMT6plex [K	0,03	-0,14	0,13
[K],VTAEADSSSPTGILATSESK	1xTMT6plex [K20];1xTM	A0MZ66	A0MZ66 [486-505]	A0MZ66 1xTMT6plex [K	0,04	-0,14	0,15
[R],RGSSPGSLEIPK,[D]	1xTMT6plex [K12];1xTM	Q6GYQ0	Q6GYQ0 [858-869]	Q6GYQ0 1xTMT6plex [	0,03	-0,14	0,14
[K],LMLPDSPLVEEGR,[R]	1xOxidation [M2];1xTM	Q9P2F8	Q9P2F8 [1456-1468]	Q9P2F8 1xOxidation [M	0,03	-0,13	0,14
[R],ALSPLSPVAIEQTSLK,[M]	1xTMT6plex [K16];1xTM	Q96K83	Q96K83 [603-618]	Q96K83 1xTMT6plex [K	0,03	-0,13	0,14
[R],SGVGTGPPSPIALPPLR,[A]	1xTMT6plex [N-Term];1	Q92630	Q92630 [40-56]	Q92630 1xTMT6plex [N	0,05	-0,13	0,16
[R],DRTPPLLYR,[D]	1xTMT6plex [N-Term];1	Q96T37	Q96T37 [566-574]	Q96T37 1xTMT6plex [N	0,00	-0,13	0,11
[R],KASSPSPLTIGTPESQR,[K]	1xTMT6plex [K1];1xTM	Q9NPI6	Q9NPI6 [520-536]	Q9NPI6 1xTMT6plex [K	0,04	-0,13	0,16
[R],GLSDHVSLDGQELGTR,[S]	1xTMT6plex [N-Term];1	Q7L8J4	Q7L8J4 [356-371]	Q7L8J4 1xTMT6plex [N	0,04	-0,13	0,15
[R],NLDNVSPK,[D]	1xTMT6plex [K8];1xTM	P78563	P78563 [21-28]	P78563 1xTMT6plex [K	0,04	-0,13	0,15

[K],VGSLTPPSSPK,[T]	1xTMT6plex [K11];1xTM	Q2M2I8	Q2M2I8 [616-626]	Q2M2I8 1xTMT6plex [K	0,01	-0,13	0,12
[R],TPSSPLQSCTPSLSPR,[S]	1xCarbamidomethyl [C	Q96AP0	Q96AP0 [336-351]	Q96AP0 1xCarbamidom	0,00	-0,13	0,12
[R],TSSLTQFPPSQSEER,[S]	1xTMT6plex [N-Term];1	Q9H6Z4	Q9H6Z4 [124-138]	Q9H6Z4 1xTMT6plex [N	0,00	-0,13	0,11
[R],RNPSPDHSYK,[R]	1xTMT6plex [K10];1xTM	Q15696	Q15696 [381-390]	Q15696 1xTMT6plex [K	0,05	-0,13	0,16
[R],AGAPGALSPSYDGGLHGL	1xTMT6plex [K21];1xTM	P15923	P15923 [372-392]	P15923 1xTMT6plex [K	0,01	-0,13	0,12
[R],GYTSDSEVYTDHGRPGK,[	1xTMT6plex [K17];1xTM	Q92538	Q92538 [1315-1331]	Q92538 1xTMT6plex [K	0,04	-0,13	0,16
[R],AILGSYDSELTPAEYSPQLT	1xTMT6plex [N-Term];1	Q9Y6D9	Q9Y6D9 [413-433]	Q9Y6D9 1xTMT6plex [N	0,03	-0,13	0,14
[K],RSLPALR,[Q]	1xTMT6plex [N-Term];1	O00268	O00268 [659-665]	O00268 1xTMT6plex [N	0,01	-0,13	0,12
[R],TSSLTQFPPSQSEER,[S]	1xTMT6plex [N-Term];1	Q9H6Z4	Q9H6Z4 [124-138]	Q9H6Z4 1xTMT6plex [N	0,00	-0,13	0,11
[R],SGSISVK,[I]	1xTMT6plex [K7];1xTM	Q8TF01	Q8TF01 [724-730]	Q8TF01 1xTMT6plex [K	0,04	-0,13	0,15
[R],LILTDSGSAPTFCSDDEDV	1xCarbamidomethyl [C1	P78312	P78312 [280-304]	P78312 1xCarbamidom	0,02	-0,13	0,13
[K],LAGSPIR,[T]	1xTMT6plex [N-Term];1	Q8WXG6	Q8WXG6 [1267-1273]	Q8WXG6 1xTMT6plex	0,05	-0,13	0,17
[K],NLYPSSSPYTR,[N]	1xTMT6plex [N-Term];1	Q9Y2Z0	Q9Y2Z0 [275-285]	Q9Y2Z0 1xTMT6plex [N	0,02	-0,13	0,13
[K],LTSSCPDLPSQTDKK,[C]	1xCarbamidomethyl [C	P56962	P56962 [286-300]	P56962 1xCarbamidom	0,03	-0,13	0,14
[K],LTSSCPDLPSQTDKK,[C]	1xCarbamidomethyl [C	P56962	P56962 [286-300]	P56962 1xCarbamidom	0,03	-0,13	0,14
[K],GHTGGHHSPVK,[G]	1xTMT6plex [K11];1xTM	Q96I15	Q96I15 [122-132]	Q96I15 1xTMT6plex [K	0,01	-0,13	0,12
[R],KDGSSPPLLEK,[Q]	2xTMT6plex [K1; K11];	Q6W2J9	Q6W2J9 [419-429]	Q6W2J9 2xTMT6plex [	0,03	-0,13	0,14
[R],SISLSQSAENVPASK,[F]	1xTMT6plex [K15];1xTM	Q4ADV7	Q4ADV7 [1015-1029]	Q4ADV7 1xTMT6plex [	0,00	-0,12	0,12
[K],SILPYPVSPK,[Q]	1xTMT6plex [K10];1xTM	Q8IWI9	Q8IWI9 [917-926]	Q8IWI9 1xTMT6plex [K	0,00	-0,12	0,12
[R],AQSTDSLGTSGSLQSK,[A]	1xTMT6plex [K16];1xTM	Q15276	Q15276 [405-420]	Q15276 1xTMT6plex [K	0,03	-0,12	0,13
[K],GKQSPPGPGK,[A]	2xTMT6plex [K2; K10];	O75362	O75362 [792-801]	O75362 2xTMT6plex [K	0,03	-0,12	0,14
[K],LAPVPSPEPQKPAPVSPES	2xTMT6plex [K11; K21]	Q96JM3	Q96JM3 [199-219]	Q96JM3 2xTMT6plex [K	0,02	-0,12	0,13
[R],KPLSLAGDEETECQSSPK,[	1xCarbamidomethyl [C1	Q86TN4	Q86TN4 [225-242]	Q86TN4 1xCarbamidom	0,04	-0,12	0,16
[K],RLLPSVWSESEDGAGSPP	1xTMT6plex [N-Term];1	Q9UIS9	Q9UIS9 [384-404]	Q9UIS9 1xTMT6plex [N	0,04	-0,12	0,15
[R],RDSSGGTKDGDRPK,[I]	2xTMT6plex [K8; K14];	Q9Y478	Q9Y478 [22-35]	Q9Y478 2xTMT6plex [K	0,02	-0,12	0,12
[R],DLVQPDKPASPK,[F]	2xTMT6plex [K7; K12];	Q6PJT7	Q6PJT7 [506-517]	Q6PJT7 2xTMT6plex [K	0,04	-0,12	0,16
[K],FNEEHIPDSPFVVPVASPS	1xTMT6plex [N-Term];1	P21333	P21333 [2311-2333]	P21333 1xTMT6plex [N	0,01	-0,12	0,12
[R],GGSPDLWK,[S]	1xTMT6plex [K8];1xTM	Q96JM3	Q96JM3 [474-481]	Q96JM3 1xTMT6plex [K	0,04	-0,12	0,15
[R],SVSSFPVPQDNVDTHPGS	1xTMT6plex [K20];1xTM	Q676U5	Q676U5 [287-306]	Q676U5 1xTMT6plex [K	0,04	-0,12	0,15
[K],FANLTPSR,[T]	1xTMT6plex [N-Term];1	O15027	O15027 [2050-2057]	O15027 1xTMT6plex [N	0,00	-0,12	0,12
[K],AVSPPHLDGPPSPR,[S]	1xTMT6plex [N-Term];1	P29590	P29590 [516-529]	P29590 1xTMT6plex [N	0,01	-0,11	0,12
[R],ALPSLNTGSSSPR,[G]	1xTMT6plex [N-Term];1	O14545	O14545 [317-329]	O14545 1xTMT6plex [N	0,01	-0,11	0,12
[R],YIASVQGSTPSPR,[Q]	1xTMT6plex [N-Term];1	P49792	P49792 [11-23]	P49792 1xTMT6plex [N	0,05	-0,11	0,16
[R],TRSPSPTLGESLAPHK,[G]	1xTMT6plex [K16];1xTM	Q86UU1	Q86UU1 [516-531]	Q86UU1 1xTMT6plex [	0,01	-0,11	0,12
[K],ESVSTEDLSPPSPPLPK,[E]	1xTMT6plex [K17];1xTM	Q13127	Q13127 [853-869]	Q13127 1xTMT6plex [K	0,02	-0,11	0,13
[R],SGAHSSASPPR,[S]	1xTMT6plex [N-Term];1	P18615	P18615 [174-184]	P18615 1xTMT6plex [N	0,03	-0,11	0,14
[R],IHIDPEIQDGSPTTSR,[R]	1xTMT6plex [N-Term];1	P50479	P50479 [102-117]	P50479 1xTMT6plex [N	0,03	-0,11	0,14
[R],SPSILAAK,[L]	1xTMT6plex [K8];1xTM	Q569K4	Q569K4 [394-401]	Q569K4 1xTMT6plex [K	0,02	-0,11	0,13
[R],LVGATATSSPPPK,[A]	1xTMT6plex [K13];1xTM	Q96QD9	Q96QD9 [8-20]	Q96QD9 1xTMT6plex [	0,03	-0,10	0,14
[R],KLSGDQITLPTTVDYSSVP	2xTMT6plex [K1; K20];	O00559	O00559 [34-53]	O00559 2xTMT6plex [K	0,05	-0,10	0,16
[R],AAVVTSPPPTTAPHK,[E]	1xTMT6plex [K15];1xTM	P35611	P35611 [7-21]	P35611 1xTMT6plex [K	0,03	-0,10	0,14
[R],GQGSSPVAMQK,[A]	1xOxidation [M9];1xTM	P18206	P18206 [342-352]	P18206 1xOxidation [M	0,03	-0,10	0,13
[R],HSWGPGK,[N]	1xTMT6plex [K7];1xTM	Q12802	Q12802 [1564-1570]	Q12802 1xTMT6plex [K	0,03	-0,10	0,14
[R],LTQYHGGSLPNVSQLR,[S]	1xTMT6plex [N-Term];1	Q6UUV7	Q6UUV7 [55-70]	Q6UUV7 1xTMT6plex [	0,03	-0,10	0,14
[R],EALAEAALESPRPALVR,[S]	1xTMT6plex [N-Term];1	O14745	O14745 [271-287]	O14745 1xTMT6plex [N	0,03	-0,10	0,14
[R],GNLLHFPSSQGEEEK,[E]	1xTMT6plex [K15];1xTM	Q12888	Q12888 [1060-1074]	Q12888 1xTMT6plex [K	0,04	-0,10	0,15
[K],SLDSEPSVPSAAKPPSPEK	2xTMT6plex [K13; K19]	Q7Z3K3	Q7Z3K3 [410-428]	Q7Z3K3 2xTMT6plex [K	0,05	-0,09	0,16
[R],NLHQSGFSLSGTQVDEGV	1xTMT6plex [N-Term];1	Q14195	Q14195 [532-550]	Q14195 1xTMT6plex [N	0,01	-0,09	0,12
[K],IQSSLSSASPSK,[A]	1xTMT6plex [K12];1xTM	Q5W0B1	Q5W0B1 [711-722]	Q5W0B1 1xTMT6plex [	0,04	-0,09	0,15
[K],LDSSPSVSSTLAAK,[D]	1xTMT6plex [K14];1xTM	Q13428	Q13428 [1225-1238]	Q13428 1xTMT6plex [K	0,03	-0,08	0,13
[K],LIPGPLSPVAR,[G]	1xTMT6plex [N-Term];1	P48634	P48634 [1213-1223]	P48634 1xTMT6plex [N	0,04	-0,08	0,15
[R],LGTLSPAFSTR,[V]	1xTMT6plex [N-Term];1	Q7KZF4	Q7KZF4 [777-787]	Q7KZF4 1xTMT6plex [N	0,04	-0,08	0,15
[R],STSTPNVHMVSTTLPVDSR	1xTMT6plex [N-Term];1	P04049	P04049 [257-275]	P04049 1xTMT6plex [N	0,02	-0,08	0,13
[R],TASPPALPK,[I]	1xTMT6plex [K9];1xTM	Q13029	Q13029 [641-649]	Q13029 1xTMT6plex [K	0,03	-0,08	0,14
[R],ALPSLNTGSSSPR,[G]	1xTMT6plex [N-Term];1	O14545	O14545 [317-329]	O14545 1xTMT6plex [N	0,02	-0,08	0,12
[K],VPSTPPPK,[A]	1xTMT6plex [K8];1xTM	Q9H7N4	Q9H7N4 [973-980]	Q9H7N4 1xTMT6plex [	0,01	-0,07	0,12
[K],LQPSSSPENSLDPFPPR,[I]	1xTMT6plex [N-Term];1	Q8WWM	Q8WWM7 [554-570]	Q8WWM7 1xTMT6plex	0,03	-0,06	0,14
