# Supplementary material for: PP2A‐based triple‐strike therapy overcomes mitochondrial apoptosis resistance in brain cancer cells
Source: Mol Oncol. 2023 Jul 26;17(9):1803–20. doi: 10.1002/1878-0261.13488 (PMC10483611; doi:10.1002/1878-0261.13488)
Supplement: Supplementary file 3 — Table S2. Enriched Reactome pathways based on phosphopeptides regulation. [file MOL2-17-1803-s003.pdf]

Table S2. Enriched Reactome pathways based on phosphopeptide regulation

The following table shows the 25 most relevant pathways sorted by p-value.

Pathway name	Entities				Reactions	
	found	ratio	p-value	FDR*	found	ratio
Signaling by Rho GTPases	225 / 678	0.06	5.20e-14	9.41e-11	129 / 203	0.015
Signaling by Rho GTPases, Miro GTPases and RHOBTB3	226 / 694	0.062	2.87e-13	2.60e-10	130 / 212	0.016
RHO GTPase cycle	162 / 452	0.04	7.92e-13	4.78e-10	53 / 91	0.007
Processing of Capped Intron-Containing Pre-mRNA	97 / 245	0.022	2.77e-10	1.25e-07	32 / 32	0.002
CDC42 GTPase cycle	70 / 155	0.014	5.03e-10	1.82e-07	4 / 6	4.42e-04
RAC1 GTPase cycle	78 / 185	0.016	1.09e-09	3.30e-07	4 / 6	4.42e-04
Transport of Mature mRNA derived from an Intron-Containing Transcript	40 / 78	0.007	1.07e-07	2.77e-05	4 / 4	2.95e-04
mRNA Splicing - Major Pathway	70 / 180	0.016	1.57e-07	3.54e-05	9 / 9	6.63e-04
mRNA Splicing	72 / 188	0.017	1.84e-07	3.70e-05	14 / 14	0.001
Transport of Mature Transcript to Cytoplasm	42 / 87	0.008	2.54e-07	4.59e-05	13 / 13	9.58e-04
SUMO E3 ligases SUMOylate target proteins	67 / 174	0.015	4.02e-07	6.60e-05	85 / 131	0.01
SUMOylation	68 / 180	0.016	6.37e-07	9.62e-05	88 / 140	0.01
RHOA GTPase cycle	59 / 150	0.013	1.01e-06	1.40e-04	4 / 6	4.42e-04
Cell Cycle	187 / 670	0.06	1.90e-06	2.46e-04	366 / 449	0.033
Apoptotic execution phase	28 / 52	0.005	3.34e-06	4.01e-04	31 / 57	0.004
Chromatin organization	80 / 240	0.021	7.32e-06	7.76e-04	63 / 85	0.006
Chromatin modifying enzymes	80 / 240	0.021	7.32e-06	7.76e-04	63 / 85	0.006
Cell Cycle, Mitotic	152 / 537	0.048	8.89e-06	8.89e-04	283 / 350	0.026
RHOB GTPase cycle	33 / 71	0.006	9.87e-06	9.32e-04	3 / 6	4.42e-04
SUMOylation of DNA damage response and repair proteins	36 / 81	0.007	1.04e-05	9.32e-04	16 / 24	0.002
RHOC GTPase cycle	34 / 75	0.007	1.21e-05	0.001	4 / 6	4.42e-04
Signaling by ALK fusions and activated point mutants	28 / 57	0.005	1.73e-05	0.001	27 / 29	0.002
Signaling by ALK in cancer	28 / 57	0.005	1.73e-05	0.001	28 / 37	0.003
Apoptotic cleavage of cellular proteins	21 / 38	0.003	3.67e-05	0.003	23 / 38	0.003
mRNA 3'-end processing	27 / 57	0.005	4.42e-05	0.003	3 / 3	2.21e-04

* False Discovery Rate
